# Synthesis of a Novel Type of 2,3′‐BIMs via Platinum‐Catalysed Reaction of Indolylallenes with Indoles

**DOI:** 10.1002/chem.201705417

**Published:** 2018-03-05

**Authors:** Lisa Cooper, José Miguel Alonso, Louise Eagling, Helen Newson, Sachini Herath, Christopher Thomson, Andrew Lister, Catherine Howsham, Brian Cox, María Paz Muñoz

**Affiliations:** ^1^ School of Chemistry University of East Anglia Earlham Road Norwich NR4 7TJ UK; ^2^ Novartis Pharmaceuticals (UK) Limited RH12 5AB, Horsham West Sussex UK

**Keywords:** allenes, heterocycles, indoles, reaction mechanisms, platinum

## Abstract

Optimisation, scope and mechanism of the platinum‐catalysed addition of indoles to indolylallenes is reported here to give 2,3′‐BIMs with a novel core structure very relevant for pharmaceutical industry. The reaction is modulated by the electronic properties of the substituents on both indoles, with the 2,3′‐BIMs favoured when electron donating groups are present. Although simple at first, a complex mechanism has been uncovered that explains the different behaviour of these systems with platinum when compared with other metals (e.g. gold). Detailed labelling studies have shown Pt‐catalysed 6‐*endo‐trig* cyclisation of the indollylallene as the first step of the reaction and the involvement of two cyclic vinyl‐platinum intermediates in equilibrium through a platinum carbene, as the key intermediates of the catalytic cycle towards the second nucleophilic attack and formation of the BIMs.

## Introduction

The indole moiety is regarded as one of the most ubiquitous compound constituents in nature, for example, as part of the essential amino acid tryptophan. Consequently, indoles have become a significant class of heterocycles in biomedical research, and a very important scaffold in organic chemistry.^1^

**Figure 1:**
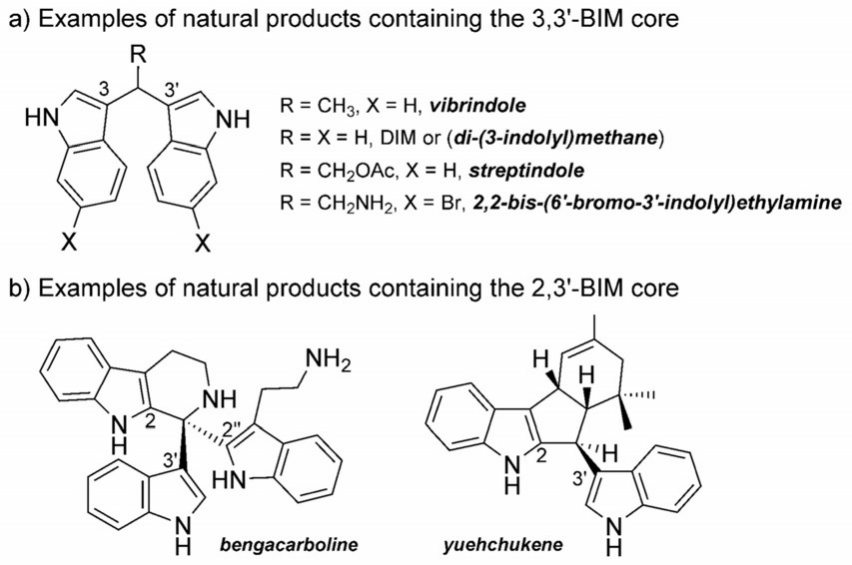
Examples of natural products containing the 3,3′‐ and 2,3′‐BIM framework.

Bisindolylmethanes (BIMs) are molecules that contain two indole units bonded to the same carbon.^2^ Natural products containing the BIM framework have been found from marine and terrestrial sources (Figure 1 a).^3^ Natural and synthetic BIMs have shown very important biological activities, including antiviral, antitumor, antibacterial, antimicrobial, anti‐inflammatory, antibiotic, genotoxicity and DNA‐damaging properties.^4^

There are different classes of BIMs according to the connectivity of the two indoles to the carbon. The most common structures are the 3,3′‐BIMs, where the indoles are connected through their most reactive position, *C*3 (Figure 1 a).

**Scheme 1:**
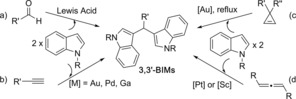
Different approaches to the synthesis of 3,3′‐BIMs.

3,3′‐BIMs are usually synthesised by condensation of indoles and aldehydes in the presence of Lewis acids (Scheme 1 a).^5^ When indoles are substituted in *C*3 position, 2,2′‐BIMS can be obtained using similar methods. More recently, new methodologies employing different metal catalysts in the reaction of indoles and unsaturated precursors have been reported.^6^ The most studied reaction is the two‐fold addition of indoles to alkynes catalysed by Au, Ga, Pd, Ru or Cu (Scheme 1 b),6d–6m but the Au‐catalysed reaction of indoles with cyclopropenes for the synthesis of 3,3′‐BIMs has also been reported (Scheme 1 c).6c Metal‐catalysed reactions of indoles with allenes has been widely explored in recent years.^7^ The reactivity encountered in the presence of gold or palladium follows the trend of the intermolecular reaction of allenes with other nucleophiles, normally giving addition of one indole to the less substituted carbon of the allene with formation of *E*‐allylated indoles.8c–8e However, reactions with platinum6b, 8a and scandium6a have shown to switch this reactivity, appearing as new methods for the synthesis of 3,3′‐BIMs (Scheme 1 d).

Due to the high reactivity of indole through the *C*3 position, the synthesis of 2,3′‐BIMs is fundamentally a more challenging target for chemists.^9^ However, this framework appears in very interesting alkaloid natural products such as bengacarboline10a or yuehchukene (Figure 1 b),10b and some efforts have been made towards the synthesis of fused cyclopenta[b]indoles containing the 2,3′‐BIM scaffold using mainly acid catalysis.^11^

**Scheme 2:**
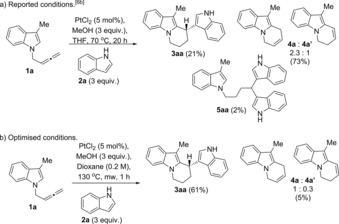
Platinum‐catalysed reaction of 3‐methyl‐*N‐*(2,3‐butadienyl)indole with an external indole: using reported conditions for bisindolylation of allenes (a)6b and optimised conditions (b).

In our previous work, we reported the Pt‐catalysed reaction of allenes with indoles to give 3,3′‐BIMs (Scheme 1 d).6b An example of 2,2′‐BIM and a 3,3′‐BPM (bispyrrolylmethane)^12^ were also reported although in lower yields. Using this method, we reported the first example of platinum‐catalysed reaction of a *N*‐allenylindole (**1 a**)^13^ in the presence of an external indole (**2 a**) to give a tetrahydropyrido[1,2a]indole (**3 aa**) by addition of the internal and the external indoles to the allene, albeit in low yield and cycles from the single intramolecular addition as the main products in the reaction (Scheme 2 a). This unique reactivity observed under platinum catalysis makes this reaction a potentially powerful new method for the synthesis of substituted tetrahydropyrido[1,2a]indoles: an original type of 2,3′‐BIM with a framework so far not observed in nature and of interest to the pharmaceutical industry.

Herein, we report our results in the optimisation and scope of this reaction and a mechanistic study, which emphasises the importance of this new reaction in the context of the divergent reactivity encountered with platinum when compared with other metals (e.g. gold).

## Results and Discussion

### Optimisation of reaction conditions

Reaction of 3‐methyl‐*N‐(*2,3‐butadienyl)indole (**1 a**) in the presence of external indole (**2 a**) under the reported conditions for the intermolecular bisindolylation of allenes (70 °C in THF over 20 hours, in the presence of 3 equivalents of MeOH) gave tetrahydropyrido[1,2a]indole (**3 aa**) in low yield (21 %) (Scheme 2 a).6b The reaction was initially limited to indolylallenes bearing a methyl group in the *C*3 position (such as **1 a**). Unsubstituted *N‐(*2,3‐butadienyl)indole gave the 6‐*endo*‐cyclisation product (analogous to **4 a**) in low yield with many impurities, and the *C*2‐methyl analogue gave mainly the double intermolecular bisindolylation on the terminal carbon of the allene (analogous to **5 aa**).

In order to optimise the reaction conditions towards selective formation of the desired 2,3′‐BIM, we carried out a screening of the reaction of 3‐methyl‐*N‐(*2,3‐butadienyl)indole (**1 a**) in the presence of external indole (**2 a**), changing all possible variables: solvent, temperature, method of heating, concentration, time, equivalents of PtCl_2_, indole and MeOH (see Supporting Information for details). Remarkably, the reaction time was decreased to 1 hour, and the isolated yield of compound **3 aa** was increased to 71 % (measured by LCMS; 61 % isolated yield) when using dry dioxane as solvent, 3 equivalents of methanol,^14^ 3 equivalents of indole at 130 °C under microwave heating (0.2 m), as the optimal conditions (Scheme 2 b).

### Scope of the reaction

With the optimised conditions for the intra‐intermolecular bisindolylation reaction established, the substitution in the internal and external indole and the chain length between the indole and the allene were changed to study the scope and limitations of the reaction. Substituted indole frameworks are widely present in natural products^15^ used in pharmaceutical industry. This makes them ideal candidates for expanding the scope of this reaction as substituted 2,3′‐BIMs could have enhanced potential for biological activity. Additionally, the effect that substituents with different electronic properties may have on the reactivity would be a valuable tool to understand the mechanism of this new transformation.

With the exception of commercially available 5‐bromo‐3‐methyl indole, all of the other 5‐substituted‐3‐methylindoles were synthesised from their corresponding 5‐substituted indole by regioselective methylation of the *C*3‐position using reported procedures,^16^ followed by *N*‐propargylation and Crabbé homologation under microwave conditions^17^ to form the corresponding 5‐substituted‐3‐methylindolyl allenes **1 x**.

The scope of the reaction under the optimised platinum‐catalysed conditions is shown in Table 1. Results in Table 1 highlight an interesting trend for the selective formation of **4 x:4 x′** or **3 xy** depending on the nucleophilicity of the indolylallene and the external indole.^18^ The desired 2,3′‐BIM **3 xy** was formed preferentially when position 5 was substituted with an electron‐donating group (entries 2 and 3, Table 1), while formation of the 6‐*endo* cyclised products **4 x:4 x′**, in ≈1:1 in all cases (entries 4–6, Table 1), was favoured when position 5 contained an electron‐withdrawing group.

**Table 1:** Reaction of substituted‐3‐methylindolylallenes **1 x** with substituted indoles **2 y**.

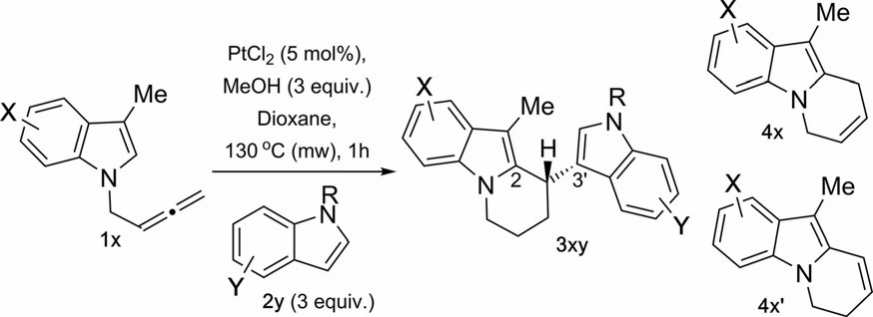				
Entry	**1 x**, X	**2 y**, Y	**3 xy** [%]^[a]^	**4 x:4 x′** [%],^[a]^ ratio
1	**1 a**, X=H	**2 a**, Y=H, R=H	**3 aa**, 61^[b]^	**4 a:4 a′**, 5 (1:0.3)
2^[c]^	**1 b**, X=5‐OMe	**2 a**, Y=H, R=H	**3 ba**, 48	**4 b:4 b′**, 0
3	**1 c**, X=5‐Me	**2 a**, Y=H, R=H	**3 ca**, 21	**4 c:4 c′**, 0
4	**1 d**, X=5‐Br	**2 a**, Y=H, R=H	**3 da**, 20	**4 d:4 d′**, 53, (1:0.95)
5	**1 e**, X=5‐CN	**2 a**, Y=H, R=H	**3 ea**, 0	**4 e:4 e′**, 47, (0.78:1)
6	**1 f**, X=5‐Cl	**2 a**, Y=H, R=H	**3 fa**, 0	**4 f:4 f′**, 35, (0.8:1)
7^[d]^	**1 a**, X=H	**2 b**, Y=5‐OMe, R=H	**3 ab**, 58	**4 a:4 a′**, 0
8^[e]^	**1 a**, X=H	**2 c**, Y=2‐Me, R=H	**3 ac**, 57	**4 a:4 a′**, 0
9^[f]^	**1 a**, X=H	**2 d**, Y=2‐Ph, R=H	**3 ad**, 35	**4 a:4 a′**, traces
10^[f]^	**1 a**, X=H	**2 e**, Y=5‐CN, R=H	**3 ae**, traces	**4 a′**, 74
11^[g]^	**1 a**, X=H	**2 f**, Y=5‐CN, R=Me	**3 af**, 0	**4 a′**, 71
12^[f]^	**1 a**, X=H	**2 g**, Y=5‐Cl, R=H	**3 ag**, traces	**4 a′**, 74
13	**1 a**, X=H	**2 h**, Y=5‐Br, R=H	**3 ah**, traces	**4 a′**, 33
14^[h]^	**1 a**, X=H	**2 i**, Y=H, R=Me	**3 ai**, 61	**4 a:4 a′**, 0
15^[i]^	**1 a**, X=H	**2 j**, Y=4‐OMe, R=H	**3 aj**, 62	**4 a:4a′**, 0
16^[i]^	**1 a**, X=H	**2 k**, Y=5‐OMe, R=Me	**3 ak**, 59	**4 a:4 a′**, 0
17^[i]^	**1 a**, X=H	**2 l**, Y=4‐OMe, R=Me	**3 al**, 60	**4 a:4 a′**, 0
18^[i]^	**1 a**, X=H	**2 m**, Y=H, R=Et	**3 am**, 63	**4 a:4 a′**, traces
19	**1 b**, X=5‐OMe	**2 b**, Y=5‐OMe, R=H	**3 bb**, 40	**4 b:4 b′** traces

[a] Isolated Yields. [b] 71 % by LCMS ELSD. [c] 6 % of **3 ba** and 51 % of **4 a** with wet MeOH. [d] Reaction carried out during 3 h; at 1 h: **3 ab**, 37 %, **4 a:4 a′**, 17 % (0.35:1), 3–10 % of dimer **7 a**. [e] Reaction carried out during 3 h; at 1 h: **3 ac**, 46 %, **4 a:4 a′**, 19 % (0.2:1). [f] 3–10 % of dimer **7 a**. [g] Reaction carried out during 3 h; at 1 h: **4 a**, 8 %, **4 a′** 67 %. [h] Reaction carried out during 3 h; at 1 h: **3 ai**, 22 %, **4 a′**, 44 %. [i] Reaction carried out during 3 h.

A similar trend was observed for the reaction with substituted external indoles, where more nucleophilic indoles bearing an electron‐donating group favour the formation of the desired 2,3‐BIM **3 xy**, whereas electron‐withdrawing groups favour the formation of the 6‐*endo* cycle product **4 a′** (entries 7–19, Table 1).

Interestingly, carrying out the reaction at longer times improved yields of the 2,3′‐BIMs in cases where electron‐donating groups were used in either component (entries 7, 8, 14–18, Table 1).

To further understand the effect of substituents in the indolylallene on the selectivity of the reaction, investigations were carried out with various substituents in position 3 of the indolylallene (Table 2). The starting indoles, either commercially available or synthesised using known procedures,^19^ were propargylated and homologated to the corresponding allenes.^17^ The subsequent reactions were carried out using the optimised platinum conditions in the presence of indole **2 a**. Substitution and steric bulk in this position had a dramatic effect on the reaction. Table 2 shows the results from the reaction with alkyl groups of different sizes (entries 1–5), where formation of both the conjugated 6‐*endo* cycle **4 x:4 x′** and the 2,3‐BIM **3 xa** is observed. However, the selectivity for **4 x** vs. **4 x′** is dependent on the size of the substituent in position 3. If position 3 bears a methyl group, formation of **3 aa** is favoured (entry 1, Table 2), whereas when the *i*Pr group is present **4 h′** is preferentially formed (entry 3, Table 2). It is worth noting that substitution at *C*3‐position is essential to obtain the 2,3′‐BIMs. Unsubstituted *N‐(*2,3‐butadienyl)indole (R=H) decomposed under the optimised conditions using microwave heating and gave cycles **4:4′** (R=H) as 1:1.6 mixture in 17 % as the only products of the reaction under the original reaction conditions (THF 70 °C, 20 h).6b

**Table 2:** Reaction of 3‐substituted indolylallenes **1 x** with indole **2 a**.

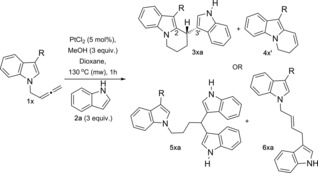					
Entry	**1 x**, R in *C*3	**3 xa** [%]^[a]^	**4 x:4 x′** [%]^[a]^	**5 xa** [%]^[a]^	**6 xa** [%]^[a]^
1	**1 a**, R=Me	**3 aa**, 61	**4 a**:**4 a′** 5 (1:0.3)	0	0
2^[b]^	**1 g**, R=Et	**3 ga**, 41	**4 g′** traces	0	0
3	**1 h**, R=*i*Pr	**3 ha**, 7	**4 h′** 27	0	0
4	**1 i**, R=*t*Bu	**3 ia**, 19	**4 i′** 25	0	0
5	**1 j**, R=Ph	**3 ja**, 11	**4 j:4 j′** traces (1:1)	0	0
6	**1 k**, R=CN	0	0	**5 ka**, 41	**6 ka**, 15
7	**1 l**, R=CO_2_Me	0	0	**5 la**, 32	**6 la**, 13
8	**1 m**, R=CHO	0	0	**5 ma**, 5	**6 ma**, 5
9	**1 n**, R=COMe	0	0	**5 na**, 23	**6 na**, 11

[a] LCMS ratio, full conversion observed. [b] 7% of dimer **7g**.

Interestingly we observed a switch in reactivity with formation of tris‐indole **5 xa** and allyl indole **6 xa** derivatives as the only products of the reaction with allenylindoles bearing electron‐withdrawing groups in position 3 (entries 6–9). These two compounds arise from the intermolecular addition of the external indole to the terminal carbon of the allene. Removal of electron density from position 2 of the indolylallene encourages the nucleophilic addition of the indole to the terminal carbon of the allene instead of carbocyclisation, as in the previously reported reactions with platinum and gold. The greater the electron‐withdrawing properties of the substituent, the better the selectivity for the tris‐indole product **5 xa**, for example, CN versus CHO (entries 6 and 8, Table 2).

**Scheme 3:**
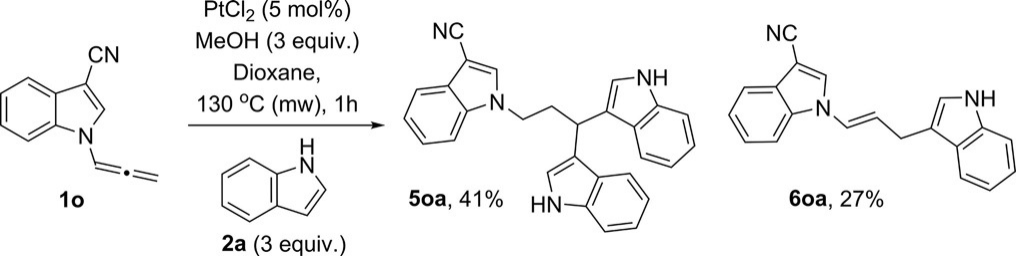
Reaction of indolylallene **1 o** with a shorter chain.

Similar reactivity was observed in the reaction of the indolylallene with a shorter chain (*n*=0) and a CN‐group in *C*3 position **1 o** that only gave tris‐indole **5 oa** and allyl indole derivative **6 oa** under the same conditions (Scheme 3).

**Scheme 4:**
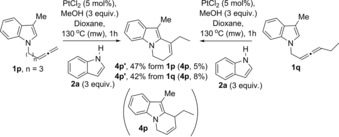
Reaction of indolylallenes **1 p** and **1 q** under optimised conditions.

Attempts to synthesise the 3‐methyl indolylallenes with other chain lengths (e.g. *n*=0 or *n*=2) failed.^20^ The reaction of the 3‐methyl indolylallene **1 p**, with *n*=3, under optimised platinum‐catalysis gave only the product from the 6‐*exo‐trig* cyclisation **4 p′**, by reaction with the internal carbon of the allene and double bond isomerisation (Scheme 4). The same product **4 p′** was obtained in similar yield when the reaction was carried out with 1,3‐disubstituted allene **1 q** (by *6‐endo‐trig* cyclisation and isomerisation of the double bond), which suggests that substitution at that position hinders the second nucleophilic attack. Traces of isomer heterocycle **4 p** were obtained in both cases. Similar results were obtained in the reactions of these derivatives when no external indole was present, with only cyclisation products observed as expected.

**Scheme 5:**
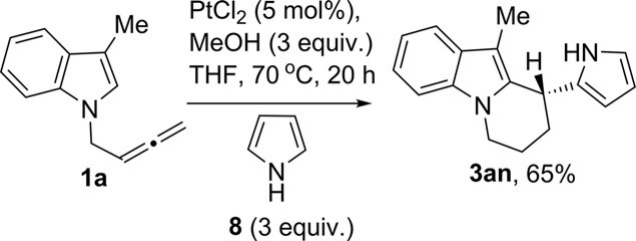
Further scope with pyrrole.

To further show the scope and expand the structural diversity of the products obtained in this reaction, we carried out the reaction of indolylallene **1 a** in the presence of pyrrole **8** as the external nucleophile (Scheme 5).^21^ Remarkably, the reaction gives only the product of the intra‐intermolecular attack **3 an** in 65 % yield, emphasising the importance of this reaction as a new method to obtain novel polyheterocyclic structures not tested so far as potential drugs.

### Mechanistic investigations

A preliminary mechanism for the intermolecular platinum‐catalysed bis‐indolylation6b and the analogous dihydroalkoxylation^22^ of allenes proposed that coordination of the platinum to the terminal carbon of the allene favours the first nucleophilic attack of one of the indoles to give a vinyl‐platinum intermediate, as proposed in analogous gold‐catalysed reactions. The divergent reactivity of platinum versus gold and other metals arises from the possibility of protonation of the internal carbon instead of the most common protonolysis of the C−metal bond, to form a platinum carbene that evolves by 1,2‐H shift and second nucleophilic attack.^23^ Within the context of the synthesis of BIMs, we speculated that the reaction could follow a similar pathway, where the first nucleophilic attack could occur intermolecularly with the external indole and the intramolecular reaction would happen in the final step. Alternatively, the cyclisation would occur first and the external indole would be added in the final step.^24^ Here we report our efforts to understand this mechanism and the implications on the reaction.

### 1 ‐*endo* Cyclisation products 4 x:4 x′ as intermediates

In order to clarify the involvement of cycles **4 x** and **4 x′** as intermediates in the formation of the 2,3′‐BIMs and to get more evidence on the pathways involved in the reaction, we synthesised the non‐conjugated cycle **4 a** via gold‐catalysed cyclisation of 3‐methyl‐*N‐*(2,3‐butadienyl)indole **1 a** as reported by Barluenga et al.13a This molecule was submitted to platinum catalysis in the absence and presence of external indole.^25^, ^26^

**Scheme 6:**
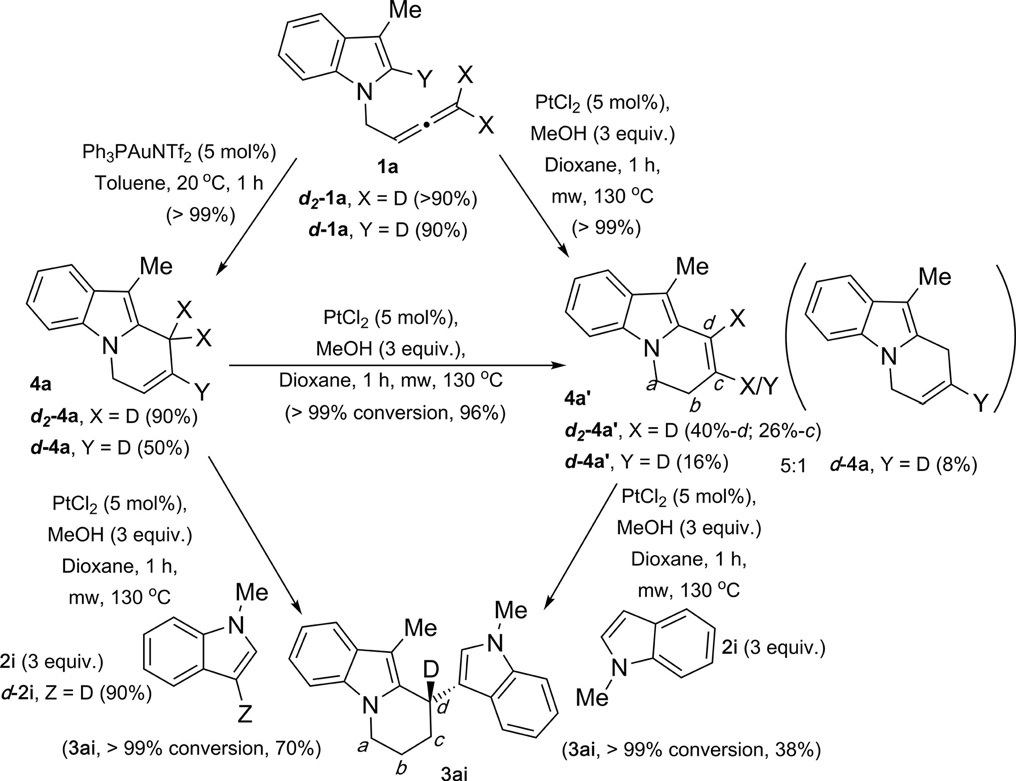
Reactions with cycles **4 a**/**4 a′** as intermediates.

The reaction of **4 a** in the absence of indole confirmed full isomerisation to **4 a′** with an isolated yield of 96 % (Scheme 6). Interestingly, this isomerisation in the absence of methanol does not go to completion, and a mixture of the two isomers **4 a:4 a′** with a ratio of 0.7:1 was observed. The reaction of **4 a** under optimised conditions for platinum catalysis with the external indole **2 i** gave the 2,3′‐BIM **3 ai** with >99 % conversion and 70 % yield (Scheme 5).

Finally, the conjugated cycle **4 a′** was reacted under optimised platinum catalysis with the external indole **2 i**, giving >99 % conversion to the desired 2,3′‐BIM **3 ai** (Scheme 6). It is worth emphasising here that gold was neither able to promote isomerisation of **4 a** to **4 a′** nor catalyse the addition of indole to any of the cycles. Monitoring the reaction of isolated **4 a** by ^1^H NMR over time, under optimised platinum catalysis, using dimethyl sulfone as internal reference and *C*3‐*d*‐indole ***d*** **‐2 i** (90 % D), showed that the concentration of **4 a′** increases during the first 20 minutes, followed by a first order decay, with formation of the 2,3′‐BIM **3 ai** (see Supporting Information for full details). Loss of deuterium in *C*3 of the external indole was also observed from early on in the reaction, with an overall loss of 41 % D. Analysis of the ^1^H NMR spectra showed deuterium incorporation at position *d* of cycle **4 a′** and position *d* of **3 ai** within the first 10 minutes of the reaction, with 31 % D incorporated in the final compound **3 ai**.

Furthermore, similar reactions were carried out with *d*_2_‐3‐methyl‐*N‐(*2,3‐butadien‐1‐yl) indole ***d***_**2**_ **‐1 a**, synthesised via Crabbé homologation of 3‐methyl‐1‐(2‐propyn‐1‐yl)‐1*H*‐indole with deuterated paraformaldehyde, and with *d*‐3‐methyl‐*N‐(*2,3‐butadienyl)indole ***d*** **‐1 a**, bearing deuterium in the *C*2 position of the indole, synthesised following reported procedures.^27^ The deuterated indolylallenes were reacted independently under gold catalysis to form the non‐conjugated cycles ***d***_**2**_‐**4 a** and ***d*** **‐4 a**, respectively. These were reacted separately under platinum conditions in the absence of indole to study the deuterium transfer in the isomerisation to ***d*** **‐4 a′**, and in the presence of indole to investigate the deuteration pattern in the 2,3′‐BIM **3 ai** (see the Supporting Information for full details). All of these experiments support a mechanism where cyclisation occurs first, followed by isomerisation of **4 a** to **4 a′**, with subsequent intermolecular addition of the external indole. However, the loss of deuterium when ***d*** **‐2 i** was used, the inefficient deuterium transfers and the deuterium incorporation patterns observed in all these experiments suggest a complex exchange of protons in the reaction media, which prompted us to perform further labelling studies.

### Further labelling experiments

**Scheme 7:**
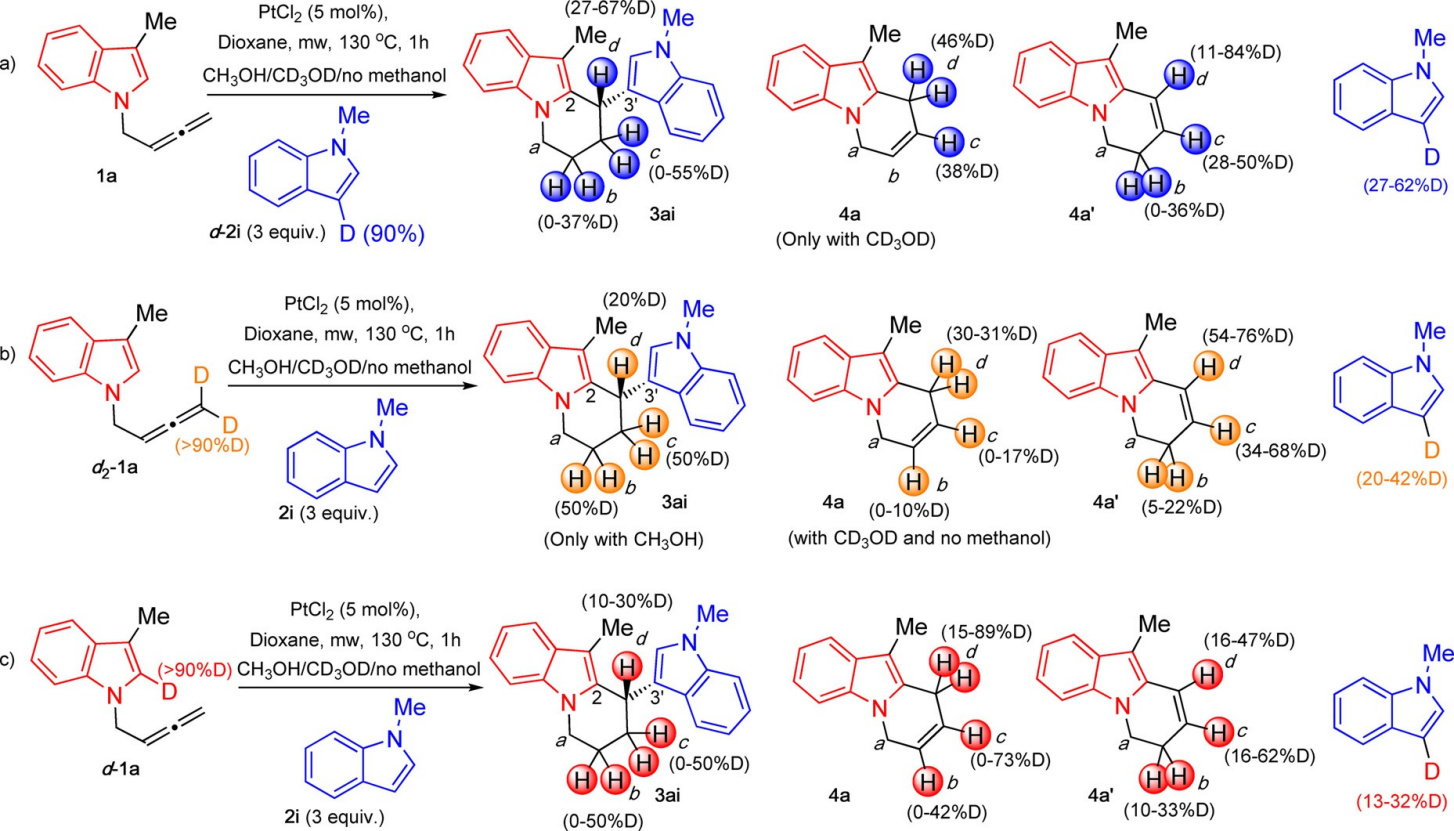
Deuteration experiments.

Reactions were carried out using deuterated and non‐deuterated starting materials (**1 a**, ***d***_**2**_ **‐1 a**, ***d*** **‐1 a**, **2 i**, ***d*** **‐2 i**) or a combination of them in the absence and presence of methanol, either CH_3_OH or CD_3_OD. The crude reaction mixtures were analysed by ^1^H NMR and the purified products were analysed when possible by ^1^H NMR and HSQC to confirm deuterium incorporation (see the Supporting Information for full details). Scheme 7 summarises the deuterium incorporation patterns observed in the experiments carried out.

From the experiments shown in Scheme 7 it can be seen that deuterium is scrambled from any position in the starting materials to all positions *b*, *c* and *d* in the three products in all conditions tested. When deuterated ***d***_**2**_ **‐1 a**, ***d*** **‐1 a** or ***d*** **‐2 i** were used, the yields of BIM were similar to the obtained in the reaction with the non‐deuterated analogues, as long as MeOH was used as additive in the reaction. This suggests that there is no KIE in the steps involving the *C*2‐position of the indole in the indolylallene (6‐*endo*‐cyclisation, **A** to **D**, Scheme 9 vide infra), the terminal position of the allene (1,2‐H‐shift, **E** to **H**, Scheme 9 vide infra) or the *C*3‐position of the external indole (intermolecular nucleophilic attack, **H** to **I**, Scheme 9 vide infra).

In all cases, formation of the BIM was less favoured in the absence of methanol or when CD_3_OD was used in the process, suggesting that the methanol is somehow involved in the rate‐limiting step of the reaction. Interestingly, when ***d***_**2**_ **‐1 a** was used in combination with ***d*** **‐2 i** no BIM was formed even in the presence of methanol. This could be explained by the increased deuterium scramble with the methanol.

Deuterium incorporation was in general slightly higher when CD_3_OD was used as additive, in the cases when the BIM was formed. Nevertheless, the deuterium incorporation in the three products is not maintained. Loss of deuterium is observed when ***d***_**2**_ **‐1 a**, with deuterium in the terminal position of the allene, is used. Similarly, deuterium loss was observed in the recovered excess indole ***d*** **‐2 i** in the reaction in equation a, Scheme 7. Deuterium gain in *C*3 of the non‐labelled indole **2 i** was observed in the reaction where deuterated indolylallenes were used, even in the absence of methanol (equations b and c, Scheme 7).

**Figure 2:**
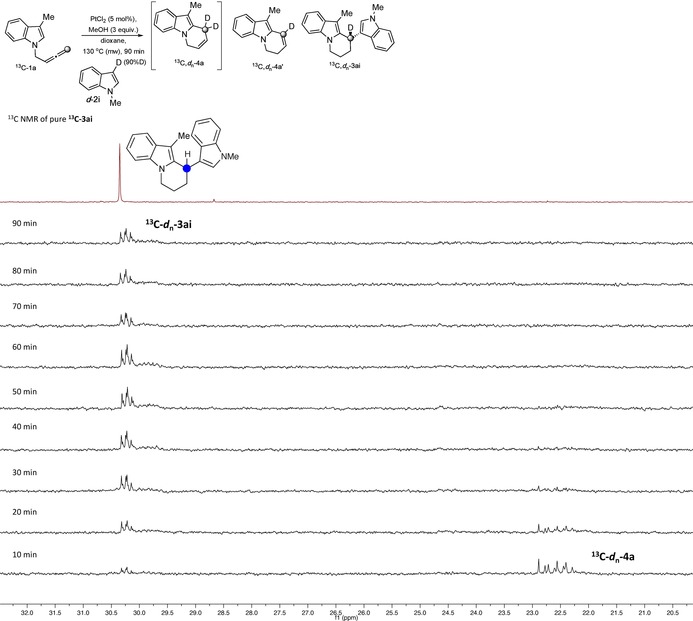
Expansion of the ^13^C NMR of the reaction of ^**13**^ **C‐1 a** and ***d*** **‐2 i** over time showing deuterium incorporation in the labelled carbon in **3 ai** and **4 a**. See Supporting Information for the signal of labelled carbon in **4 a′**.

All these results suggest that the protons at the deuterated positions in the different compounds can be washed out into the solution and exchanged with methanol protons to reprotonate the three compounds at positions *b*, *c* and *d*. Deuterium incorporation in position *d* in BIM **3 ai** was in all cases higher than expected, and also different before and after purification by column chromatography. This suggests further exchange of those protons in an acid or platinum catalysed out‐of‐cycle equilibrium. To support this, the isolated non‐labelled 2,3′‐BIM **3 ai** was reacted under platinum conditions in the presence of deuterated methanol. After 1 hour, we observed 42 % deuterium incorporation only at position *d* in **3 ai**, confirming our hypothesis. To gain more evidence, we performed an experiment with ^**13**^ **C‐1 a** in the presence of ***d*** **‐2 i**, monitoring the reaction by ^1^H and ^13^C NMR under the optimised conditions using dimethyl sulfone as the internal standard (Figure 2). Interestingly, high deuterium incorporation was observed in position *d* from the beginning of the reaction, suggesting that although further proton exchange in that position is possible, it is not the main pathway for deuterium incorporation (see full details in the Supporting Information).

### Reaction with TEMPO

**Scheme 8:**
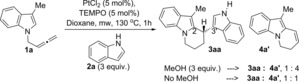
Experiments in the presence of TEMPO.

Platinum hydrides (Pt‐H) have been reported in reactions with platinum complexes and alcohols.^28^ This could have implications in the role of methanol in the reaction as Pt‐H species could be involved in several steps of the catalytic cycle. This was investigated by adding TEMPO as a Pt‐H trap^29^ to the reaction under optimised conditions in the presence and absence of methanol (Scheme 8). Analysis of these two reactions showed product **4 a′** as the major product, with compounds **4 a′** and **3 aa** present in a 4:1 ratio, in the reaction containing methanol. Formation of products **4 a′** and **3 aa** in a 1:1 ratio was observed in the reaction with no methanol present (Scheme 8). The different results obtained with and without methanol in the presence of TEMPO and in comparison, with the model reaction suggest that indeed Pt‐H might be involved in the catalytic cycle. The fact that formation of the 2,3′‐BIM **3 aa** is more inhibited in the presence of methanol with TEMPO present could point to Pt‐H species involved in the final demetalation step (vide infra).

### Proposed catalytic cycle

**Scheme 9:**
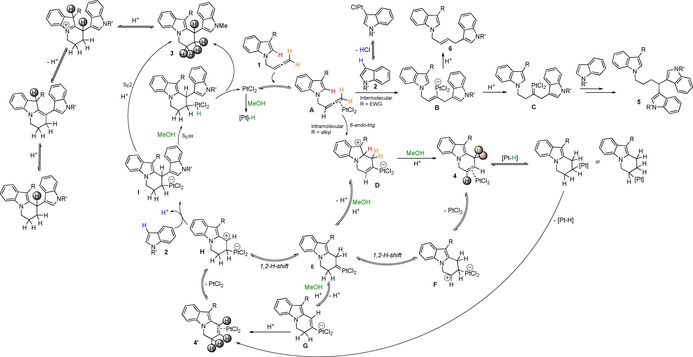
Mechanistic proposal highlighting the observed deuteration patterns.

Our proposal starts with η^2^‐coordination of platinum to the terminal double bond of indolylallene **1** to give complex **A** (Scheme 9).^30^ At this stage, the electronic properties of the group present in position 3 of the internal indole play an important role as to which pathway is taken. If position 3 of the indolylallene **1** contains an electron‐withdrawing group, intermolecular addition of the external indole to the terminal carbon of the activated allene would be the preferred pathway. This would form a vinyl‐platinum intermediate **B**, from which protodemetalation would give allyl indoles **6**. Alternatively, protonation on the internal carbon of the allene forms a platinum carbene intermediate **C**, which undergoes 1,2‐H shift and subsequent attack of the second external indole. Subsequent protodemetalation of the Pt−C_sp3_ bond (via Pt‐H or directly) would result in the tris‐indole product **5**.6b

However, when position 3 of indolylallene **1** contains an alkyl mild electron‐donating group, the complex undergoes 6‐*endo‐trig* cyclisation with formation of the cyclic vinyl‐platinum intermediate **D**. Protodemetalation of these species would explain the formation of the non‐conjugated cyclic allyl indole **4**, analogous to that proposed by Barluenga et al.13a in the gold‐catalysed cyclisation. The proton used for this step could be that lost from position 2 of the indolylallene to regain aromaticity (red proton in Scheme 9) or from the MeOH present (green proton in Scheme 9). Platinum‐catalysed C−H activation of position 3 of the external indole would give Pt^II^‐indole intermediates, unproductive compounds in our catalytic cycle, and HCl (blue proton in Scheme 9), which could also be involved in the protodemetalation of **D** to form **4** and would explain the loss or gain of deuterium in position *C*3 of the external indole during the reaction.

The isomerisation of **4** to **4′** could be explained by protonation of the internal carbon of the vinyl‐platinum intermediate **D**, aided by the methanol acting as a proton shuttle, to form a platinum–carbene complex **E**—a key intermediate in the reaction with platinum but not observed with other metals in similar systems. From this platinum‐carbene **E**, the 1,2‐H shift of the protons in position *b* would generate cationic intermediates **F** that after loss of PtCl_2_, would generate cycle **4** with the observed deuteration pattern and scramble of deuterium.

Alternatively, loss of one of the protons in position *d* (orange protons in Scheme 9) on the platinum‐carbene **E** would form the isomer cyclic vinyl‐platinum intermediate **G**, which can undergo protodemetalation (with any proton source in the media) to form the conjugated cycle **4′**. Our studies involving the cycles as intermediates in the reaction have highlighted that the non‐conjugated cycle **4** can return to the reaction without the external indole with successful isomerisation to **4′**.^31^ Therefore it is likely that platinum can re‐coordinate and add to the cycle to form intermediate **F** that can regenerate the cyclic vinyl–platinum intermediates **D** and **G**. These are in equilibrium with the platinum‐carbene **E**, which is further supported by the deuterium scramble in position *d*. Involvement of Pt‐H in this isomerisation can not be completely ruled out. However, in the experiments with TEMPO in the presence of MeOH, complete isomerisation of **4** to **4′** was observed, supporting the equilibrium via the platinum–carbene shown in Scheme 9 as the main pathway for the isomerisation.

Coordination and insertion of platinum to cycle **4′** would give a cationic intermediate **H**, which can also come from 1,2‐H shift in the platinum‐carbene. These Pt‐coordinated **4′** and cationic intermediate **H** could be seen as resonance structures that are ready to undergo the intermolecular nucleophilic addition by the external indole to form the platinum complex **I** that already possess the BIM structure. The electronic properties of the internal and external indoles are also crucial in this step. EDG‐substituted indoles would stabilise the positive charge in intermediate **H**, favouring the second nucleophilic attack of the external indole.^32^ Similarly, external indoles with EDGs would be better nucleophiles favouring the formation of BIMs.^19^ However, when bulky groups are present in position *C*3 of the indole or EWGs are present in either of the indole moieties, the intermolecular attack is less favoured, explaining the formation of the cycles as the major products of the reaction.

It has been reported that 3,3′‐BIMS can be obtained as by‐products in the reversible acid‐catalysed reaction of indoles with aldehydes, where 3‐vinyl indoles are the major products at low acid‐catalyst loading and at low temperatures.^33^ Similarly, gold‐catalysed/acid‐assisted addition of indoles to 3‐vinyl indoles also gives BIMs.6c In order to investigate the reversibility of the nucleophilic addition of the external indole to the cycle 2‐vinyl indole **4′**, the isolated BIM **3 ai** was reacted under platinum conditions with methanol and the external indole **2 a**. Indole exchange on position *d* of the BIM would imply that the BIM is able to go back into the catalytic cycle to reform the platinum‐coordinated vinyl indole **4′** or intermediate **H**, and a mixture of two different BIMs with different external indoles would be expected. However, after 1 hour of microwave irradiation, only starting material **3 ai** and the excess indole **2 a** were observed, with no traces or formation of the other 2,3′‐BIM **3 aa**. This therefore supports that the second nucleophilic attack is not reversible.

The final demetalation step is proposed to occur via two pathways depending on the presence or absence of methanol.^34^ In the presence of methanol, the protodemetalation could occur via S_E_Ox mechanism, by protonation of the platinum centre with formation of Pt^IV^−H species that can be trapped by TEMPO, explaining the poor conversion to the BIM. Reductive elimination would form BIMs **3**. In contrast, when no methanol is present, generation of Pt‐H species would be less favoured and conventional protodemetalation could occur directly in an S_e_2 mechanism with other protons present in the media.

Finally, platinum‐ or acid‐catalysed out‐of‐cycle equilibrium would explain further deuterium scramble into position *d* of the 2,3′‐BIMs **3**.

## Conclusion

In summary, we have optimised the platinum‐catalysed reaction of indolylallenes with external indoles to give substituted 2,3′‐BIMs with a novel tetrahydropyrido[1,2a]indole framework that has strong potential for biological activity.

The reaction is unique to platinum catalysis and has shown to be very sensitive to the nucleophilicity of both indoles, giving divergent reactivity depending on the ability of the indolylallene to undergo 6‐*endo‐trig* cyclisation as the preferred pathway in the first step of the reaction.

Detailed labelling and other mechanistic studies have allowed us to propose a complex mechanism in which proton scramble from all positions would account for the deuterium patterns observed. The reaction seems to proceed through cyclic vinyl‐platinum intermediates (**D** and **G** in Scheme 9) that are in equilibrium through a platinum carbene (**E** in Scheme 9) a key intermediate of the catalytic cycle and main difference in the reactivity with other metals in which the reaction stops at the cyclic allyl indole after cyclisation.

The significance of the new methodology and the use of the new structures obtained in pharmaceutical industry are highlighted by the constant need of novel chemical scaffolds for testing to fight resistance and new diseases.

Further investigations into expanding the scope of the reaction to other nucleophiles and other allenic systems using this methodology are under investigation and will be reported in due course.

## Experimental Section

**General procedure for platinum‐catalysed reaction of indolylallenes with external nucleophile under optimised conditions**: PtCl_2_ (5 mol %) and the appropriate nucleophile (3 equiv) were added to a microwave vial, capped and flashed with N_2_ atmosphere. The solids were dissolved in a small amount of dry 1,4‐dioxane and the appropriate indolylallene (1 equiv) dissolved in dry 1,4‐dioxane (0.2 m) was added. Dry methanol (3 equiv) was added and the vial was heated under microwave irradiation at 130 °C for 1 hour. The resulting reaction mixture was filtered through Celite and washed with DCM and compounds purified via column chromatography in silica gel with Pet/EtOAc.

## Conflict of interest

The authors declare no conflict of interest.

## Supporting information

As a service to our authors and readers, this journal provides supporting information supplied by the authors. Such materials are peer reviewed and may be re‐organized for online delivery, but are not copy‐edited or typeset. Technical support issues arising from supporting information (other than missing files) should be addressed to the authors.

Supplementary
