# Supplementary material for: Synthesis of a Novel Type of 2,3′‐BIMs via Platinum‐Catalysed Reaction of Indolylallenes with Indoles
Source: Chemistry. 2018 Mar 5;24(23):6105–14. doi: 10.1002/chem.201705417 (PMC5947743; doi:10.1002/chem.201705417)

CHEMISTRY
A European Journal

Supporting Information

Synthesis of a Novel Type of 2,3′-BIMs via Platinum-Catalysed Reaction of Indolylallenes with Indoles

Lisa Cooper,[a] José Miguel Alonso,[a] Louise Eagling,[a, b] Helen Newson,[a, b] Sachini Herath,[a] Christopher Thomson,[b] Andrew Lister,[b] Catherine Howsham,[b] Brian Cox,[b] and María Paz Muñoz*[a]

chem_201705417_sm_miscellaneous_information.pdf

Table of contents

Table of contents	Pages
**1. General experimental details**	1
**2. Optimisation**	2-4
**3. General procedure for platinum-catalysed reaction of indolyl allenes with external nucleophiles under optimal conditions**	4
**4. Characterisation of products**	5-22
**5. 6-Endo cyclisation products as intermediates in the formation of 2,3'-BIMs and deuterium labeling studies.**	22-32
**5.1. Synthesis of 4a with gold catalysis**	22
**5.2. Synthesis of deuterated starting materials**	23-27
**5.3. Reaction profile of 1a with *d*-2i monitored by ^{1}H NMR**	28-29
**5.4. Experiments with deuterated intermediates**	29-32
**6. Further labeling experiments**	32-40
**6.1. Deuteration experiments**	32-36
**6.2. ^{13}C-labeling experiment**	37-39
**6.3. Reaction of 3ai in the presence of $PtCl_2$ and CD_3OD**	40
**7. NMR spectra**	41-110

1. General experiment details

All reagents were purchased from commercial sources and used without further purification, unless stated otherwise. All reactions were carried out under nitrogen atmosphere and in the absence of moisture, unless stated otherwise. Reactions using microwave irradiation were carried out in Biotage Initiator+ Microwave system. Reactions were monitored using Thin Layer Chromatography (TLC) using 0.2 mm thick silica gel plates 60F-254 (5735 Merck) with a mobile phase of hexane and ethyl acetate, with visualization by illumination by uv light λ = 254 nm or staining with either potassium permanganate or phosphomolybdic acid solution. ^{1}H NMR and ^{13}C NMR spectra were recorded on a Bruker (500 MHz) spectrometer with $CDCl_3$ solvent. Chemical shifts (δ) are given in parts per million (ppm) and coupling constants values (J) are given in Hertz (Hz), and are approximated to the nearest 0.1 Hz. ^{13}C NMR was recorded using broad-band proton decoupling. Abbreviations used in NMR analyses are as follows: s = singlet, d = doublet, t = triplet, q = quartet, p = pentet, dd = doublet of doublets, dt = doublet of triplets, td = triplet of doublets, ddd = doublet of doublet of doublets, dq = doublet of quartets, qd = quartet of doublets and m = multiplet. HRMS were performed by EPSRC National Mass Spectrometry Service Centre, Swansea.

2. Optimisation

Screening of solvent, time, temperature, concentration and equivalents of platinum, indole and methanol were carried out for the reaction of 3-methyl-*N*-(2,3-butadienyl)indole **1a** with external indole **2a** to optimise the conditions for selective formation of BIM 3aa. These reactions were carried out at the Novartis laboratories using LCMS ELSD (Evaporative Light Scattering Detector) traces for detecting the different components of the reactions.

Reported reactions by Muñoz *et al.*[1] were carried out at 70°C using thermal heating in an oil bath. However, it was established that using microwave irradiation decreased the reaction time dramatically from 20 hours to 1-2 hours as well as increasing both the selectivity and yield of product **3aa**. Microwave irradiation was therefore used for all further screening and extensive screening of solvent and temperature was carried out with THF, 1,4-Dioxane, CPME (cyclopentylmethyl ether) and 2-methyl THF (**Table 2.1**). We found that 1,4-dioxane at 130°C under microwave irradiation for 1 hour gave 71 % yield (61% isolated) to desired compound **3aa**. For practical experimental reasons, concentration of 0.2M was preferred due to the limitation of minimum solvent required for microwave experiments.

Table 2.1. Screening of conditions for the intra-intermolecular reaction of indole **2a** and 3-methyl-*N*-(2,3-butadienyl)indole **1a**.

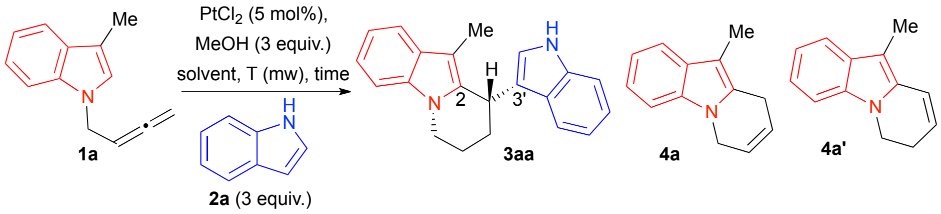

Entry	Solvent	Concentration	Temp (°C)	Time (min)	% **4a/4a'**[a]	% **3aa**[a]
1	THF	0.2M	100	180	0	33
2	THF	0.2M	130	30	0	37
3	1,4-Dioxane	0.2M	130	60	5 (0.3:1)	61
4	1,4-Dioxane	0.1M	120	60	12 (1:1.3)	58
5	1,4-Dioxane	0.4M	120	60	0	68
6	1,4-Dioxane	0.2M	150	15	0	47
7	CPME	0.2M	180	20	5	39
8	2-Methyl THF	0.2M	150	15	0	5

a) Isolated product.

1 M. P. Muñoz, M. de la Torre and M. A. Sierra, *Chem. Eur. J.* **2012**, *18*, 4499-4504.

Variations in equivalents of platinum, indole and methanol were then explored. We found that although results were slightly better with $PtCl_2$ 10 mol %, similar reactivity was achieved when 5 mol % was used, so this was chosen as the optimized conditions. **Table 2.2** shows the results when different amounts of methanol were used, with the best results achieved with 3 or more equivalents as already reported (entries 3-5).

Table 2.2. Screening eqs of MeOH for the intra-intermolecular reaction of indole **2a** and 3-methyl-N-(2,3-butadienyl)indole **1a**.

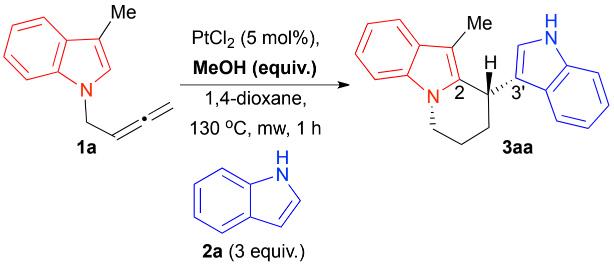

Entry	Equivalents of MeOH	% conversion to **3aa**[a]
1	1	92
2	2.5	95
3	3	100
4	3.5	100
5	4	100

a) Measured by LCMS ELDS.

The equivalent of indole to allene was also investigated and **Table 2.3** shows that using 3 or 4 equivalents gives the best conversion to product **3aa**.

Table 2.3. Screening eqs of indole **2a** for the intra-intermolecular reaction of indole **2a** and 3-methyl-N-(2,3-butadienyl)indole **1a**.

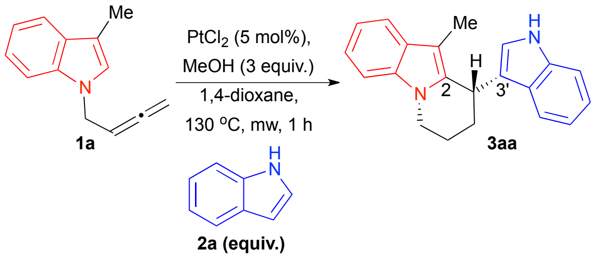

Entry	Equivalents of indole **2a**	% conversion to **3aa**[a]
1	1	100[b]
2	1.5	91
3	2	91
4	2 .5	90

5	3	100
6	4	100
7	4.5	96
8	5	85

a) Measured by LCMS ELDS. b) Results with one equivalent were less reproducible.

After this screening we chose the optimised conditions as: microwave irradiation at 130°C for 1 hour with 5 mol % $PtCl_2$, 3 eqs indole, 3 eqs of methanol and 1,4-dioxane (0.2 M). These newly optimised conditions were able to give an isolated yield of 61 % for compound **3aa**. It is important to state that the reaction requires dry 1,4-dioxane as the solvent as reactions carried out with wet 1,4-dioxane under optimised conditions resulted mainly in the formation of the cyclised products **4a/4a’** (**Table 2.4**).

Table 2.4. Effect of solvent moisture on the selectivity of the reaction.

Entry	Dioxane	% yield of **4a/4a’**	% yield of **3aa**
1	Wet	58 % (not isolated)	0
2	Dry	5	61

3. General procedure for platinum-catalysed reaction of indolyl allene with external nucleophile under optimised conditions

$PtCl_2$ (5 mol %) and the appropriate nucleophile (3 eqs) were added to a microwave vial, capped and flashed with N_2 atmosphere. The solids were dissolved in dry 1,4-dioxane and the appropriate indolylallene (1 eq) dissolved in dry 1,4-dioxane (0.2 M) was added. Dry methanol (3 eqs) was added and the vial was heated under microwave irradiation at 130°C for 1 hour, or the appropriated time indicated for each particular compound. The resulting reaction mixture was filtered through Celite and washed with DCM and compounds purified via column chromatography in silica gel with Pet/EtOAc.

4. Characterisation of products

2,3'-BIM **3aa** and cycles **4a/4a'**:

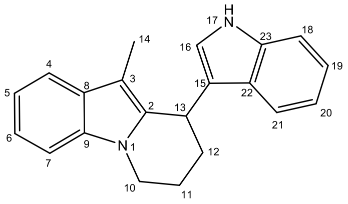

Cycle **4a** Cycle **4a'**

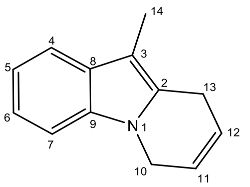

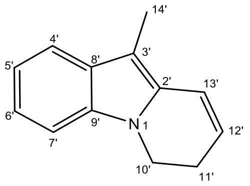

Synthesized using the general procedure from 1-(2, 3-butadien-1-yl)-3-methyl-1H-indole (75 mg, 0.41 mmol), dry methanol (50 µl, 1.23 mmol), indole (144 mg, 1.23 mmol), $PtCl_2$ (5.45 mg, 0.02 mmol) in dry 1,4-dioxane (2 mL, 0.2 M). Products obtained by column chromatography, Pet/EtOAc, (50:1): 4 mg, 0.02 mmol, 5 % of compound **4a** and **4a'** (1:0.3, inseparable mixture of isomers) as an orange/yellow solid; (20:1), 76 mg, 0.25 mmol, 61 % of compound **3aa** as a green/yellow oil.

Cycles 4a and 4a': ^{1}**H NMR** (500 MHz, $CDCl_3$) δ 7.48 – 7.42 (m, 2H, H-***7+7'***), 7.21– 6.92 (m, 6H, H-***4-6*** and ***4'-6'***), 6.59 (dt, *J* = 9.9, 1.8 Hz, 1H, H-***13'***), 6.04 – 5.93 (m, 2H, H-***11*** and H-***12***), 5.92 – 5.86 (m, 1H, H-***12'***), 4.53 (ddd, *J* = 6.7, 4.4, 2.7 Hz, 2H, H-***10***), 3.98 (t, *J* = 6.9 Hz, 2H, H-***10'***), 3.47 – 3.41 (m, 2H, H-***13***), 2.58 – 2.52 (m, 2H, H-***11'***) 2.24 (s, 3H, H-***14'***), 2.19 (s, 3H, H-***14***). ^{13}**C NMR** (126 MHz, $CDCl_3$) δ 136.74 (C-**9'**), 135.3 (C-**9**), 131.6 (C-**2'**), 129.2 (C-**2**), 129.2 (C-**8'**), 128.5 (C-**8**), 122.9 (CH, C-***12'***), 122.1 (CH, C-***11*** or ***12***), 121.9 (CH, C-***4-6*** or ***4'-6'***), 120.5 (CH, C-***11*** or ***12***), 120.3 (CH, C-***4-6*** or C-***4'-6'***), 119.2 (CH, C-***4-6*** or C-***4'-6'***), 118.9 (CH, C-***4-6*** or C-***4'-6'***), 118.8 (CH, C-***7*** or C-***7'***), 118.5 (CH, C-***13'***), 117.8 (CH, C-***7*** or C-***7'***), 108.5 (CH, C-***4-6*** and C-***4'-6'***), 108.4 (CH, C-***4-6*** and C-***4'-6'***), 107.5 (C-***3'***), 104.5 (C-***3***) 41.8 (CH_2, C-***10***), 39.8 (CH_2, C-***10'***), 24.4 (CH_2, C-***11'***), 22.8 (CH_2, C-***13***), 8.3 (CH_3, C-***14***), 8.1 (CH_3, C-***14'***). Spectra consistent with previously published data.[1]

2,3'-BIM 3aa: ^{1}**H NMR** (500 MHz, $CDCl_3$) δ 7.81 (s, 1H, H-***17***), 7.66 (d, *J* = 7.9 Hz, 1H, H-***4***), 7.57 (d, *J* = 7.8 Hz, 1H, H-***7***), 7.36 (t, *J* = 7.4 Hz, 2H, H-***5*** + H-***6***), 7.23 (m, 2H, H-***19***+ H-***20***), 7.16 (t, *J* = 7.5 Hz, 2H, H-***18*** + H-***21***), 6.52 (s, 1H, H-***16***), 4.81 (t, *J* = 4.3 Hz, 1H, H-***13***), 4.34 – 4.25 (m, 1H, H-***10***), 3.94 (td, *J* = 11.1, 4.8 Hz, 1H, H-***10***), 2.44 – 2.35 (m, 1H, H-***12***), 2.24 – 2.15 (m, 1H, H-***12***), 2.15 – 2.06 (m, 1H, H-***11***), 2.03 (s, 3H, H-***14***), 1.97 – 1.88 (m, 1H, H-***11***). ^{13}**C NMR** (126 MHz, $CDCl_3$) δ 136.70 (C-***2***), 135.88 (C-***9***), 134.87 (C-***23***), 128.74 (C-***8***), 126.22 (C-***22***), 123.28 (CH, C-***16***), 121.94 (CH, C-***19/20***), 120.29 (CH, C-***19/20***), 119.34 (CH, C-***18/21***), 118.98 (CH, C-***4***), 118.78 (C-***3***), 118.05 (CH, C-***7***), 111.36 (CH, C-***5/6***), 108.65 (CH, C-***5/6***), 105.96 (C-***15***), 42.58 (CH_2, C-***10***), 30.42 (CH, C-***13***), 28.17 (CH_2, C-***12***), 19.56 (CH_2, C-***11***), 8.36 (CH, CH_3, C-***14***). Spectra consistent with previously published data.[1] **M.P.** 151-158°C.

2,3'-BIM **3ba**

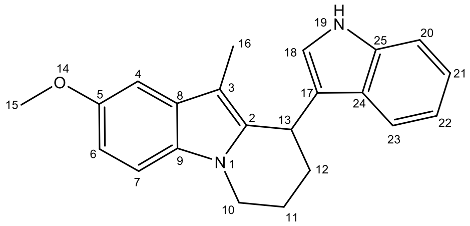

Synthesised using the general procedure from 5-methoxy-1-(2,3-butadien-1-yl)-3-methyl-1H-indole (60 mg, 0.28 mmol), dry methanol (34 µl, 0.84 mmol), indole (98 mg, 0.84 mmol), $PtCl_2$ (3.7 mg, 0.014 mmol) in 1.4 mL of dry 1,4-dioxane. Obtained by column chromatography, Pet/EtOAc, (20:1), 44.1 mg, 0.13 mmol, 48 % of compound **3ba** as a fluorescent yellow solid.

^{1}H NMR (500 MHz, $CDCl_3$) δ 7.88 (s, 1H, H-***19***), 7.63 (d, *J* = 8.2 Hz, 1H, H-***7***), 7.37 (d, *J* = 8.2 Hz, 1H, H-***20***), 7.23-7.19 (m, 2H, H-***21+22***), 7.16-7.11 (m, 1H, H-***6***), 6.98 (d, *J* = 2.4 Hz, 1H, H-***4***), 6.85 (dd, *J* = 8.2, 2.4 Hz, 1H, H-***23***), 6.56 (d, *J* = 1.6 Hz, 1H, H-***18***), 4.77 (t, *J* = 4.3 Hz, 1H, H-***13***), 4.26-4.2 (m, 1H, H-***10***), 3.91-3.86 (m, 1H, H-***10***), 3.88 (s, 3H, H-***15***), 2.35 – 2.32 (m, 1H, H-***12***), 2.20 – 2.12 (m, 1H, H-***12***), 2.11-2.05 (m, 1H, H-***11***), 1.97 (s, 3H, H-***16***), 1.93-1.86 (m, 1H, H-***11***). **^{13}C NMR** (126 MHz, $CDCl_3$) δ 153.96 (C-***5***), 136.69 (C-***2***), 135.63 (C-***9***), 131.27 (C-***8***), 128.93 (C-***25***), 126.22 (C-***24***), 123.23 (CH, C-***18***), 121.94 (CH, C-***21/22***), 119.33 (CH, C-***6***), 118.97 (CH, C-***7***), 118.85 (C-***3***), 111.31 (CH, C-***20***), 110.10 (CH, C-***23***), 109.26 (CH, C-***21/22***), 105.58 (C-***17***), 100.31 (CH, C-***4***), 56.08 (CH_3, OMe, C-***15***), 42.61 (CH_2, C-***10***), 30.40 (CH, C-***13***), 28.07 (CH_2, C-***12***), 19.55 (CH_2, C-***11***), 8.38 (CH_3 – C-***16***). **ν_{max}/cm^{-1}**: 3410 (s, NH), 3053, 2942 – 2830 (m, C-H), 1618 – 1578 (w, C=C), 1484 (s, C-H methyl), 1456 (m, C-C in ring), 1227 (s, C-N). **HRMS** (FTMS + p NSI) ((DCM) / MeOH + NH_4OAc): Calc. for $C_{22}H_{22}N_2O$ $[M+H]^+$: 331.1805: Found: 331.1805. **M.P.** 189-192°C.

2,3'-BIM **3ca**

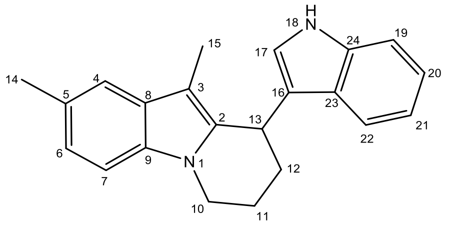

Synthesised using the general procedure from 5-methyl-1-(2,3-butadien-1-yl)-3-methyl-1H-indole (45 mg, 0.23 mmol), dry methanol (3.1 µl, 0.68 mmol), indole (80 mg, 0.68 mmol), $PtCl_2$ (3 mg, 0.011 mmol) in 1.14 mL of dry 1,4-dioxane (0.2 M). Obtained by column chromatography, Pet/Et_2O (20:1) 15.1 mg, 0.05 mmol, 21 % of compound **3ca** as a yellow oil.

^{1}H NMR (500 MHz, $CDCl_3$) δ 7.85 (s, 1H, H-***18***), 7.64 (d, *J* = 8.1 Hz, 1H, H-***Ar***), 7.36 (d, *J* = 8.1 Hz, 1H, H-***Ar***), 7.32 (d, *J* = 0.7 Hz, 1H, H-***Ar***), 7.24 – 7.18 (m, 2H, H-***Ar***), 7.16 – 7.11 (m, 1H, H-***Ar***), 7.02 (dd, *J* = 8.1, 1.4 Hz, 1H, H-***Ar***), 6.53 (dd, *J* = 2.3, 0.7 Hz, 1H, H-***17***), 4.78 (t, *J* = 4.3 Hz, 1H, H-***13***), 4.25 (ddd, *J* = 11.2, 4.9, 3.5 Hz, 1H, H-***10***), 3.89 (td, *J* = 11.2, 4.9 Hz, 1H, H-***10***), 2.49 (s, 3H, H-***14***), 2.39 – 2.33 (m, 1H, H-***12***), 2.21 – 2.12 (m, 1H, H-***12***), 2.11 – 2.01 (m, 1H, H-***11***), 1.98 (s, 3H, H-***15***), 1.92 – 1.85 (m, 1H, H-***11***). **^{13}C NMR** (126 MHz, $CDCl_3$) δ 136.69 (C-***2***), 134.9 (C-***9***), 134.3 (C-***24***), 128.9 (C-***5***), 128.1 (C-***8***), 126.2 (C-***23***), 123.3 (CH, C-***17***), 121.9 (CH Ar) 121.8 (CH Ar), 119.8 (C-***3***), 119.3 (CH Ar), 118.9 (CH Ar), 117.8 (CH Ar), 111.3 (CH Ar), 108.3 (CH Ar), 105.4 (C-***16***), 42.6 (CH_2, C-***10***), 30.4 (CH, C-***13***), 28.1 (CH_2, C-***12***), 21.6 (CH_3, C-***14***), 19.6 (CH_2, C-***11***), 8.3 (CH_3, C-***15***). **HRMS** (FTMS + p NSI) ((DCM) / MeOH + NH_4OAc): Calculated for $C_{22}H_{22}N$ $[M^+ +H]$: 315.1856 found: 315.1858.

2,3’-BIM **3da** and cycles **4d:4d’**

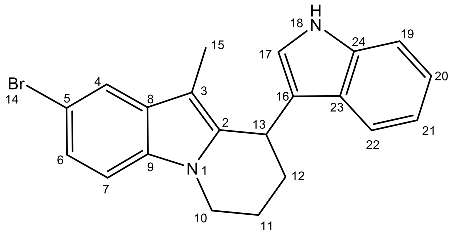

Cycle 4d

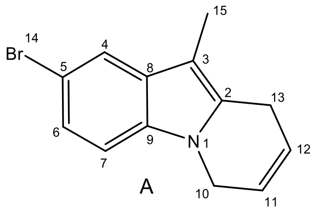

Cycle 4d’

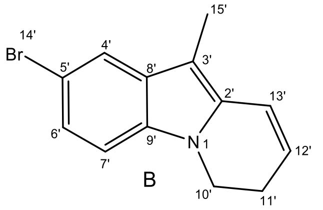

Synthesised using the general procedure from 5-bromo-1-(2,3-butadien-1-yl)-3-methyl-1H-indole (100 mg, 0.38 mmol), $PtCl_2$ (5 mg, 0.02 mmol), indole (133 mg, 1.14 mmol) and dry methanol (50 µl, 1.14 mmol) in 1.9 mL of dry 1,4-dioxane. Obtained by column chromatography, Pet:EtOAc, (15:1), 30 mg, 0.08 mmol, 20 % of compound **3da** as a brown oil and 53 mg, 0.2 mmol, 53 % of compounds **4d:4d’** as an inseparable mixture (1:0.95).

Cycles 4d:4d’: **^{1}H NMR** (500 MHz, $CDCl_3$) δ 7.56 (d, *J* = 1.7 Hz, 1H, H-***4***), 7.53 (d, *J* = 1.8 Hz, 1H, H-***4’***), 7.21 – 7.11 (m, 2H, H-***6*** and H-***7***), 7.00 (m, 2H, H-***6’*** and H-***7’***), 6.56 (d, *J* = 9.9 Hz, 1H, H-***12***), 6.02 – 5.95 (m, 1H, H-***11***), 5.92 (m, 2H, H-***13’*** and H-***12’***), 4.46 (s, 2H, H-***11’***), 3.97 – 3.88 (m, 2H, H-***13***), 3.40 (s, 2H, H-***10’***), 2.59 – 2.47 (m, 2H, H-***10***), 2.17 (s, 3H, H-***15***), 2.12 (s, 3H, H-***15’***). **^{13}C NMR** (126 MHz, $CDCl_3$) δ 135.36 (C-***9*** or ***9’***), 133.99 (C-***9*** or ***9’***), 132.57 (C-***2*** or ***2’***), 130.85 (C-***2*** or ***2’***), 130.66 (C – ***8*** or ***8’***), 130.18 (C – ***8*** or ***8’***), 124.57 (CH, C-***4***), 123.76 (CH, C-***4’***), 122.89 (CH, C-***6***), 121.90 (CH, C-***7***), 121.36 (CH, C-***6’***), 120.39 (CH, C-***7’***), 120.29 (CH, C-***12***), 118.24 (CH, C-***11***), 112.52 (C-***5*** or ***5’***), 112.00 (C-***5*** or ***5’***), 109.89 (CH, C-***13’***), 107.02 (C-***3*** or ***3’***), 104.31 (C-***3*** or ***3’***), 41.83 (CH_2, C-***11’***), 39.86 (CH_2, C-***13***), 30.95 (CH, C-***12’***), 24.31 (CH_2, C-***10’***), 22.80 (CH_2, C-***10***), 8.19 (CH_3, H-***15***), 8.04 (CH_3, H-***15’***). **ν_{max}/cm^{-1}:** 2967 (m, =C-H), 2924 (s, C-H), 2857 (m, C-H), 1699 (m, C=C), 1459 (m, C-C in ring) 1351 (w, Ar-H), 1204 (w, C-N).

2,3’-BIM 3da: **^{1}H NMR** (500 MHz, $CDCl_3$) δ 7.97 (s, 1H; H-***18***), 7.65 - 7.60 (d, *J* = 1.8 Hz, 1H, H-***7***), 7.57 (d, *J* = 8.0 Hz, 1H, H-***4***), 7.39 - 7.32 (m, 1H, H-***6***), 7.23 - 7.14 (m, 4H, H-***22***, H-***20***, H-***19*** and H-***21***), 6.51 - 6.48 (m, 1H, H-***17***), 4.75 (t, *J* = 4.4 Hz, 1H, H-***13***), 4.26 - 4.17 (m, 1H, H-***10***), 3.88 (td, *J* = 11.1, 4.9 Hz, 1H, H-***10***), 2.38 - 2.31 (m, 2H; H-***11*** and H-***12***), 2.03 (s, 3H,H-***15***), 1.92 (d, *J* = 9.4 Hz, 2H, H-***12*** and H-***11***). **^{13}C NMR** (126 MHz, $CDCl_3$) δ 136.70 (C-***9***), 136.21 (C-***24***), 134.48 (C-***2***), 130.41 (C-***8***), 126.12 (C-***23***), 123.00 (CH, C-***17***), 122.92 (CH, C-***22/21/20/19***), 122.06 (CH, C-***22/21/20/19***), 120.63 (CH, C-***4***), 119.43 (CH, C-***7***), 118.91 (CH, C-***22/21/20/19***), 118.45 (C-***5***), 112.31 (C-***3***), 111.38 (CH, C-***6***), 110.04 (CH, C-***22/21/20/19***), 105.81 (C-***16***), 42.65 (CH_2, C-*10*), 30.45 (CH, C-***13***), 28.00 (CH_2, C-***11***), 19.45 (CH_2, C-***12***), 8.24 (CH_3, C-***15***). **ν_{max}/cm^{-1}:** 3383 (m, br, N-H), 2957 (m, =C-H), 2924 (s, C-H), 2854 (m, C-H), 1713 (s, C=C), 1461 (s, Ar-H), 1366 (m, C-N). **HRMS** (FTMS + p NSI) ((MeOH)/ MeOH + NH_4OAc): Calc. for $C_{21}H_{19}{}^{79}BrN_2$ (M^{+}+H): 379.0804. Found: 379.0807. Calc. for $C_{21}H_{19}{}^{81}BrN_2$ $[M+H]^{+}$: 381.0784. Found: 381.0786.

Cycles **4e:4e'**:

Cycle 4e **Cycle 4e'**

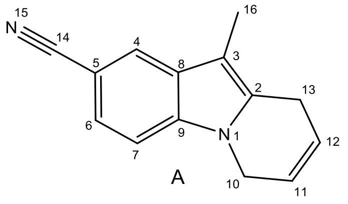
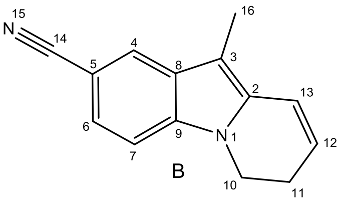

Synthesised using the general procedure from 5-cyano-1-(2,3-butadien-1-yl)-3-methyl-1H-indole (70 mg, 0.34 mmol), dry methanol (42 µl, 1.02 mmol), indole (119 mg, 1.02 mmol), $PtCl_2$ (4.5 mg, 0.02 mmol) in 2.5 mL of dry 1,4-dioxane (0.14 M). Obtained by column chromatography, Pet/EtOAc, (10:1), 32.9 mg, 0.16 mmol, 47 % of compounds **4e:4e'** as an inseparable mixture (0.78:1) as a brown oil.

^{1}H NMR (500 MHz, $CDCl_3$) δ 7.92-7.85 (m, 2H; H-***4*** and H-***4'***), 7.46-7.41 (m, 2H; H-***6*** and H-***6'***), 7.33 (d, *J* = 8.5 Hz, 1H; H-***7*** and ***7'***), 7.27 (d, *J* = 8.5 Hz, 1H, H-***7*** and ***7'***), 6.73-6.69 (m, 1H; H-***13'***), 6.13-6.09 (m, 1H, H-***11+12*** and H-***12'***), 4.69-4.64 (m, 2H, H-***10***), 4.13 (t, *J* = 7.0 Hz, 2H, H-***10'***), 3.56 (m, 2H; H-***13***), 2.70 (ddd, *J* = 9.7, 6.7, 3.3 Hz, 2H, H-***11'***), 2.34 (s, 3H, H-***16'***), 2.29 (s, 3H, H-***16***). **^{13}C NMR** (126 MHz, $CDCl_3$) δ 124.95 (CH, C-***6/6'***), 124.73 (CH, CH=C, C-***11/12/12'***), 124.19 (CH, C-***4/4'***), 123.38 (CH, C-***6/6'***), 123.33 (CH, C-***4/4'***), 121.92 (CH, CH=C, C-***11/12/12'***), 120.01 (CH, CH=C, C-***11/12/12'***), 118.08 (CH, CH=C, C-***13'***), 109.20(CH, C-***7/7'***), 109.01 (CH, C-***7/7'***), 41.96 (CH_2, C-***10***), 39.89 (CH_2, C-***10'***), 24.16 (CH_2, C-***11'***), 22.78 (CH_2, C-***13***), 14.22, 8.14 (CH_3, C-***16***), 7.97 (CH_3, C-***16'***). Some quaternary carbons not detected. **HRMS** (ASAP) Solid: Calculated for $C_{14}H_{12}N_2$ $[M+H]^+$: 209.1082. Found: 209.1079.

Cycles **4f:4f'**:

Cycle 4f **Cycle 4f'**

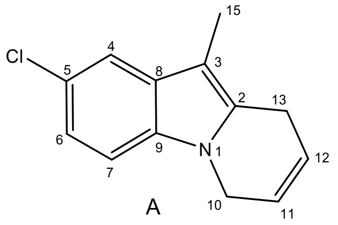
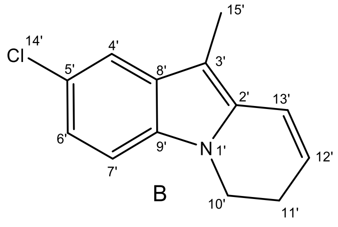

Synthesised using the general procedure from 5-chloro-1-(2,3-butadien-1-yl)-3-methyl-1H-indole (100 mg, 0.46 mmol), dry methanol (56 µl, 1.38 mmol), indole (161 mg, 1.38 mmol), $PtCl_2$ (6.1 mg, 0.023 mmol) in 3 mL of dry 1,4-dioxane (0.15 M). Obtained by column chromatography, Pet/EtOAc (20:1) 35.2 mg, 0.16 mmol, 35 % compounds **4f:4f'** as an inseparable mixture (0.8:1) as a brown oil.

^{1}H NMR (500 MHz, $CDCl_3$) δ 7.42 (d, *J* = 2.0 Hz, 2H, H-***7*** and ***7'***), 7.39 (t, *J* = 1.3 Hz, 2H, H-***4*** and ***4'***), 7.04 (d, *J* = 1.3 Hz, 2H, H-***6*** and ***6'***), 6.59 – 6.56 (m, 1H, H-***13'***), 6.04 – 5.90 (m, 3H, H-**11** and **12** and H-***12'***), 4.53 – 4.48 (m, 2H, H-***10***), 3.96 (t, *J* = 6.9 Hz, 2H, H-***10'***),3.45 – 3.41 (m, 2H, H-***13***), 2.59 – 2.54 (m, 2H, H-***11'***), 2.19 (s, 3H, H-***15'***), 2.14 (s, 3H, H-***15***). **^{13}C NMR** (126 MHz, $CDCl_3$) δ 135.1 (C-***9*** or ***9'***), 132.7 (C-***2*** or ***2'***), 130.8 (C-***5*** or ***5'***), 130.2 (C-***5*** or ***5'***), 124.9 (C-***8*** or ***8'***), 124.5 (C-***8*** or ***8'***), 123.69 (CH, C-***12'***), 122.03 (CH, C-***6*** or ***6'***), 121.89 (CH, C-***11*** or ***12***), 120.41 (CH, C-***11*** or ***12***), 118.37 (CH, C-***13'*** and ***7*** *or* ***7'***), 117.35 (CH, C-***4*** or ***4'***), 109.41 (CH, C-***7*** or ***7'***), 109.33 (CH, C-***6*** or ***6'***), 107.10 (C-***3 or 3'***), 41.85 (CH_2, C-***10***), 39.89 (CH_2, C-***10'***), 24.33 (CH_2, C-***11'***), 22.83 (CH_2, C-***13***), 8.19 (CH_3, C-***15***), 8.04 (CH_3, C-***15'***). **ν_{max}/cm^{-1}:** 2967 (m, =C-H), 2924 (s, C-H), 2857 (m, C-H), 1699 (m, C=C), 1459 (m, C-C in ring) 1351 (w, Ar-H), 1204 (w, C-N). **HRMS** (ASAP) Solid**:** Calculated for $C_{13}H_{12}{}^{35}ClN$ $[M+H]^+$: 218.0739. Found: 218.0737.

2,3'-BIM **3ab**

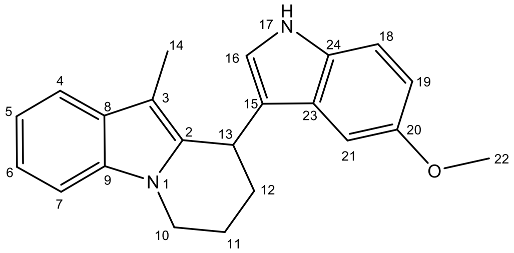

Synthesised following a modification of the general procedure: from indole **1a** (40 mg, 0.21 mmol), dry methanol (17 µl, 0.65 mmol), 5-methoxy indole (96 mg, 0.65 mmol), $PtCl_2$ (5.8 mg, 0.02 mmol) in 0.5 mL of dry 1,4-dioxane. Obtained after 3h of irradiation under microwave conditions at 130 °C (**3ab**, 58%).

2,3'-BIM 3ab: ^{1}**H NMR** (500 MHz, $CDCl_3$) δ 7.58 (s, 1H; H-***17***), 7.45 (d, *J* = 7.9 Hz, 1H; H-***7***), 7.22 (d, *J* = 7.9 Hz, 1H; H-***4***), 7.13 – 7.08 (m, 2H; H-***5*** & H-***18***), 7.07 – 7.01 (m, 1H; H-***6***), 6.89 (d, *J* = 2.4 Hz, 1H; H-***21***), 6.76 (dd, *J* = 8.6, 2.4 Hz, 1H; H-***19***), 6.35 (d, *J* = 2.3 Hz, 1H; H-***16***), 4.67 – 4.61 (m, 1H; H-***13***), 4.16 (ddd, *J* = 11.2, 5.0, 3.2 Hz, 1H; H-***10***), 3.79 (td, *J* = 11.2, 5.0 Hz, 1H; H-***10***), 3.72 (s, 3H; H-***22***), 2.27-1.95 (m, 3H; 2H-***12*** & H-***11***), 1.92 (s, 3H; H-***14***), 1.85 – 1.73 (m, 1H; H-***11***). ^{13}**C NMR** (126 MHz, $CDCl_3$) δ 154.19 (C-***20***), 136.19 (C-***9***), 135.20 (C-***24***), 132.13 (C), 129.05 (C-***8***), 126.93 (C-***23***), 124.42 (CH, C-***16***), 120.63 (CH, C-***5***), 119.30 (CH, C-***6***), 118.71 (C-***3***), 118.37 (CH, H-***7***), 112.36 (CH, C-***18***), 112.18 (CH, C-***19***), 108.98 (CH, C-***4***), 106.28 (C-***15***), 101.31 (CH, C-***21***), 56.29 (CH_3, C-***22***), 42.88 (N-CH_2, C-***10***), 30.62 (CH, C-***13***), 28.38 (CH_2, C-***12***), 19.80 (CH_2, C-***11***), 8.69 (CH_3, C-***14***). **HRMS** (FTMS + p NSI) (DCM/ MeOH + NH_4OAc): Calc. for $C_{22}H_{22}N_2O$ $[M+H]^+$: 331.1805: Found: 331.1805.

2,3'-BIM **3ac**

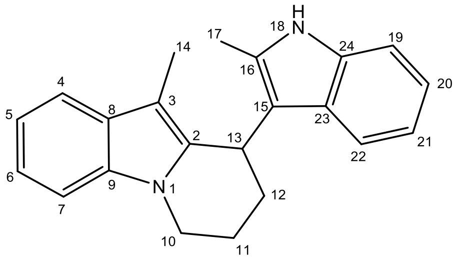

Synthesised following a modification of the general procedure: from indole **1a** (40 mg, 0.21 mmol), dry methanol (17 µl, 0.65 mmol), 2-methylindole (86 mg, 0.65 mmol), $PtCl_2$ (5.8 mg, 0.02 mmol) in 0.5 mL of dry 1,4-dioxane. Obtained after 3h of irradiation under microwave conditions at 130 °C, (**3ac**, 57%).

2,3'-BIM 3ac: ^{1}**H NMR** (500 MHz, $CDCl_3$) δ 7.69 (s, 1H; H-***18***), 7.49 (d, *J* = 8.0 Hz, 1H; H-***7***), 7.36 (d, *J* = 8.0 Hz, 1H; H-***4***), 7.27 (d, *J* = 8.0 Hz, 1H; H-***19***), 7.20 (ddd, *J* = 8.0, 7.1, 1.2 Hz, 1H; H-***5***), 7.17 – 7.06 (m, 3H; H-***22***, H-***6***, H-***20***), 6.95 (t, *J* = 7.4 Hz, 1H; H-***21***), 4.67 (t, *J* = 5.8 Hz, 1H; H-***13***), 4.28 – 4.11 (m, 2H; H-***10***), 2.23 – 1.95 (m, 7H; H-***14***, H-***12***, H-***11***), 1.83 (s, 3H, H-***17***). ^{13}**C NMR** (126 MHz, $CDCl_3$) δ 135.49 (C-***16***), 135.08 (C-***2***), 134.12 (C-***8***), 131.06 (C-***24***), 128.77 (C-***15***), 127.97 (C-***8***), 120.88 (C-***23***), 120.21 (CH, C-***5***), 119.19 (CH, C-***21***), 118.83 (CH, C-***6***), 118.33 (CH, C-***22***), 117.99 (CH, C-***7***), 113.70 (C-***3***), 110.13 (CH, C-***19***), 108.52 (CH, C-***4***), 42.57 (N-CH_2, C-***10***), 30.83 (CH, C-***13***), 29.71 (CH_2, C-***11***), 21.61 (CH_2, C-***12***), 11.77 (CH_3, C-***14***), 8.16 (CH_3, C-***17***). **HRMS** (FTMS + p NSI) (DCM/ MeOH + NH_4OAc): Calc. for $C_{22}H_{22}N_2$ $[M+H]^+$: 315.1783. Found: 315.1856.

2,3'-BIM **3ad**

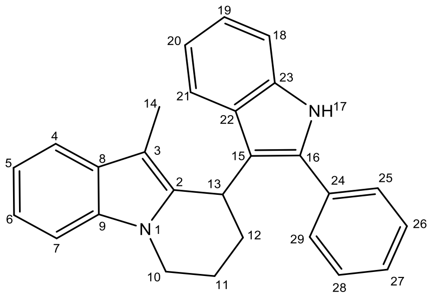

Synthesised using the general procedure from 1-(2,3-butadien-1-yl)-3-methyl-1H-indole (100 mg, 0.55 mmol), $PtCl_2$ (7.2 mg, 0.028 mmol), 2-phenyl-1H-indole (319 mg, 1.65 mmol), dry methanol (67 µL, 1.65 mmol) and 2.75 mL (0.2 M) of dry 1,4-dioxane. Obtained by column chromatography, Pet:EtOAc, (20:1), 77 mg, 0.21 mmol, 35 % of compound **3ad** as a red/brown oil.

^{1}H NMR (500 MHz, $CDCl_3$) δ 7.84 (s, 1H, H-***17***), 7.45 (d, *J* = 7.9 Hz, 2H, Ar), 7.37 (d, *J* = 7.3 Hz, 2H, Ar), 7.13 (d, *J* = 7.3 Hz, 2H, Ar), 7.06 (s, 1H, Ar), 7.03 – 7.00 (m, 2H, Ar), 6.90 – 6.86 (m, 1H, Ar), 6.81 (d, *J* = 7.8 Hz, 1H, Ar), 6.70 – 6.65 (m, 1H, Ar), 4.54 (dd, *J* = 9.2, 5.8 Hz, 1H, H-***13***), 4.11 (dd, *J* = 10.2, 5.3 Hz, 1H, H-***10***), 3.91 (td, *J* = 11.0, 4.3 Hz, 1H, H-***10***), 2.13 – 1.99 (m, 3H, H-***12*** + H-***11***), 1.91 – 1.80 (m, 1H, H-***11***), 1.50 (s, 3H, H-***14***). **^{13}C NMR** (126 MHz, $CDCl_3$) δ 137.91, 136.85, 135.52, 135.48, 134.49, 129.29, 129.06, 128.91, 127.74, 125.19, 122.38, 122.11, 120.69, 120.30, 119.57, 118.71, 115.62, 110.93, 108.42, 106.78, 42.52 (CH_2, C-***10***), 32.22 (CH, C-***13***), 30.54 (CH_2, C-***11/12***), 23.09 (CH_2, C-***11/12***), 8.36 (CH_3, C-***14***). **HRMS** (FTMS) Calculated for $C_{27}H_{24}N_2H$ $[M+1]^+$: 377.2012. Found: 377.2011.

2,3'-BIM **3ai**

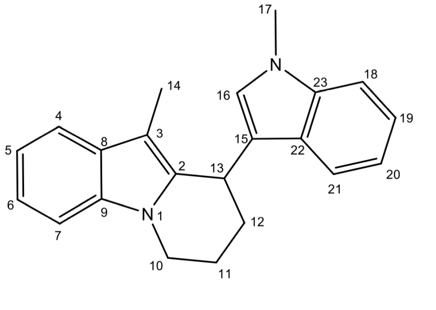

Synthesised following a modification of the general procedure: from indole **1a** (40 mg, 0.21 mmol), dry methanol (17 µl, 0.65 mmol), N-methylindole (86 mg, 0.65 mmol), $PtCl_2$ (5.8 mg, 0.02 mmol) in 0.5 mL of dry 1,4-dioxane. Obtained after 3h of irradiation under microwave conditions at 130 °C, (**3ai**, 61%).

<u>2,3'BIM 3ah</u>: **^{1}H NMR** (500 MHz, $CDCl_3$) δ 7.56 (d, *J* = 7.9 Hz, 1H, H-***4***), 7.47 (d, *J* = 7.7 Hz, 1H, H-***18***), 7.24 (dd, *J* = 14.9, 8.1 Hz, 2H, H-***7***+H-***21***), 7.17 – 7.09 (m, 2H, H-***5***+H-***19***), 7.08 – 7.03 (m, 2H, H-***6***+H-***20***), 6.30 (s, 1H, H-***16***), 4.72 (t, *J* = 4.1 Hz, 1H, H-***13***), 4.21 (ddd, *J* = 11.2, 4.9, 3.1 Hz, 1H, H-***10***), 3.82 (td, *J* = 11.2, 4.8 Hz, 1H, H-***10***), 3.56 (s, 3H, H-***17***), 2.32 – 2.26 (m, 1H, H-***12***), 2.12 – 2.06 (m, 1H, H-***12***), 2.06 – 1.98 (m, 1H, H-***11***), 1.95 (s, 3H, H-***14***), 1.84 – 1.78 (m, 1H, H-***11***). **^{13}C NMR** (126 MHz, $CDCl_3$) δ 137.4 (C-***2***), 135.9 (C-***9***), 135.07 (C-***23***), 128.7 (C-***8***), 128.12 (CH, C-***16***), 126.6 (C-***22***), 121.4 (CH, C-***6/20***), 120.2 (CH, C-***6/20***), 119.0 (CH, C-***4***), 118.9 (CH, C-***5/19***), 118.7 (CH, C-***5/19***), 118.0 (CH, C-***18***), 117.1 (C-***3***), 109.4 (CH, C-***7/21***), 108.7 (CH, C-***7/21***), 105.9 (C-***15***), 42.6 (CH_2, C-***10***), 32.6 (CH_3, C-***17***), 30.2 (CH, C-***13***), 28.2 (CH_2, C-***12***), 19.4 (CH_2, C-***11***), 8.4 (CH_3, C-***14***). **v_{max}/cm^{-1}**: 3410 (s, NH), 3053, 2942 – 2830 (m, C-H), 1618 – 1578

(w, C=C), 1484 (s, C-H methyl), 1456 (m, C-C in ring), 1227 (s, C-N). **HRMS** (FTMS + p NSI) ((DCM) / MeOH + NH_4OAc): Calc. for $C_{22}H_{22}N_2$ $[M+H]^+$: 315.4320: Found: 315.4220.

2,3'-BIM **3aj**

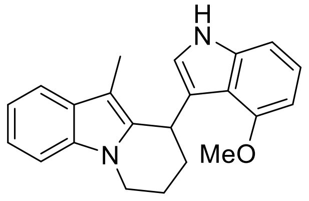

Synthesised following a modification of the general procedure: from indole **1a** (70 mg, 0.38 mmol), dry methanol (48 µl, 1.14 mmol), 4-methoxyindole (168 mg, 1.14 mmol), $PtCl_2$ (10.2 mg, 0.04 mmol) in 1 mL of dry 1,4-dioxane. Obtained after 3h of irradiation under microwave conditions at 130 °C, and isolated by column chromatography, Pet/EtOAc/DCM, (8:1:1), 78 mg, 62 % of compound **3aj** as a pale green thick oil.

^{1}H NMR (500 MHz, $CDCl_3$) δ 7.80 (s, 1H, NH), 7.54 (d, *J* = 7.7 Hz, 1H, Ar), 7.31 (d, *J* = 7.3 Hz, 1H, Ar), 7.17 (m, 1H, Ar), 7.11 (m, 2H, Ar), 6.96 (d, J= 6.9 Hz, 1H, Ar), 6.56 (d, *J* = 7.3 Hz, 1H, Ar), 6.27 (s, 1H, Ar), 5.06 (m, 1H), 4.27 (m, 1H), 3.91 (s, 3H, OMe), 3.87 (m, 1H), 2.40 (m, 1H), 2.18 (m, 2H), 2.05 (s, 3H, Me), 1.85 (m. 1H). **^{13}C NMR** (126 MHz, $CDCl_3$) δ 154.8,138.3 (1C, Ar), 136.2 (1C, Ar), 135.8 (1C, Ar), 128.8 (1C, Ar), 122.7 (1CH, Ar), 122.1 (1CH, Ar), 119.9 (1CH, Ar), 119.7 (1C, Ar), 118.8 (1CH, Ar), 117.9 (1CH, Ar), 116.5 (1C, Ar), 106.6 (1CH, Ar), 105.5 (1CH, Ar), 104.5 (1CH, Ar), 99.5 (1CH, Ar), 55.2 (1C, OMe), 42.7 (1C, CHH), 31.0 (1C, CH), 28.8 (1C, CHH), 16.7 (1C, CHH), 6.42 (1C, Me). **HRMS** (FTMS) Calculated for $C_{22}H_{23}N_2O$ $[M]^+$: 331.1805. Found: 331.1801.

2,3'-BIM **3ak**

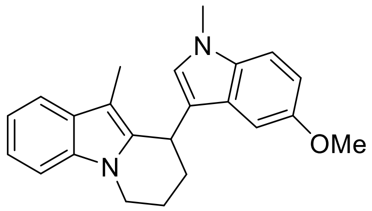

Synthesised following a modification of the general procedure: from indole **1a** (70 mg, 0.38 mmol), dry methanol (48 µl, 1.14 mmol), *N*-methyl-5-methoxyindole (161 mg, 1.14 mmol), $PtCl_2$ (10.2 mg, 0.04 mmol) in 1 mL of dry 1,4-dioxane. Obtained after 3h of irradiation under microwave conditions at 130 °C, and isolated by column chromatography, Pet/EtOAc/DCM, (8:1:1), 106 mg as a non-separable mixture of compound **3ak** (59%) and indole **2j** (starting material recovered 28 mg, 15%) as an orange thick oil.

^{1}H NMR (500 MHz, $CDCl_3$) δ 7.51 (d, *J* = 7.5 Hz, 1H, Ar), 7.46 (d, *J* = 7.5 Hz, 1H, Ar), 7.25 (m, 2H, Ar), 7.05 (m, 2H, Ar), 6.88 (s, 1H, Ar), 6.27 (s, 1H, Ar), 4.76 (m, 1H), 4.31 (m, 1H), 3.91 (m, 1H), 3.84 (s, 3H. OMe), 3.61 (s, 3H, Me), 2.35 (m, 1H), 2.34 (s, 3H, Me), 2.41 (m, 1H), 1.90 (m. 1H). **^{13}C NMR** (126 MHz, $CDCl_3$) δ 153.6 (1C, Ar), 135.8 (1C, Ar), 135.1 (1C, Ar), 132.8 (1C, Ar), 126.8 (1C, Ar), 121.8 (1CH, Ar), 120.3 (1CH, Ar), 118.9 (1CH, Ar), 118.0 (1CH, Ar), 116.5 (1C, Ar), 111.4 (1CH, Ar), 110.9 (1C, Ar), 110.1 (1CH, Ar), 106.5 (1CH, Ar), 105.9 (1C, Ar), 101.1 (1CH, Ar), 56.0 (1C, OMe), 42.6 (1C, CHH), 30.2 (1C, CH), 28.1 (1C, CHH), 19.3 (1C, CHH), 8.3 (1C, Me). **HRMS** (FTMS) Calculated for $C_{23}H_{25}N_2O$ $[M]^+$: 345.1961. Found: 345.1959.

2,3'-BIM **3al**

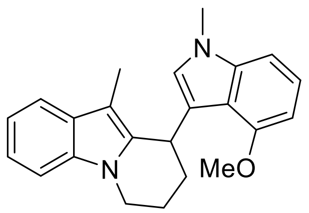

Synthesised following a modification of the general procedure: from indole **1a** (70 mg, 0.38 mmol), dry methanol (48 µl, 1.14 mmol), N-methyl-4-methoxyindole (184 mg, 1.14 mmol), $PtCl_2$ (10.2 mg, 0.04 mmol) in 1 mL of dry 1,4-dioxane. Obtained after 3h of irradiation under microwave conditions at 130C, and isolated by column chromatography, Pet/EtOAc/DCM, (20:1:1), 79 mg, 60 % of compound **3al** as a bright yellow thick oil.

^{1}H NMR (500 MHz, $CDCl_3$) δ 7.47 (d, *J* = 7.5 Hz, 1H, Ar), 7.24 (d, *J* = 7.3 Hz, 1H, Ar), 7.06 (m, 3H, Ar), 6.82 (d, J= 6.8 Hz, 1H, Ar), 6.47 (d, *J* = 6.5 Hz, 1H, Ar), 6.04 (s, 1H, Ar), 4.96 (m, 1H), 4.20 (m, 1H), 3.89 (s, 3H, OMe), 3.78 (m, 1H), 3.48 (s, 3H), 2.30 (m, 1H), 2.04 (m, 1H), 2.01 (s, 3H, Me), 1.76 (m, 1H), 1.50 (br s, 1H). **^{13}C NMR** (126 MHz, $CDCl_3$) δ 154.8 (1C, Ar), 139.0 (1C, Ar), 136.4 (1C, Ar), 135.8 (1C, Ar), 128.8 (1C, Ar), 126.9 (1CH, Ar), 122.2 (1CH, Ar), 119.9 (1CH, Ar), 118.8 (1CH, Ar), 118.1 (1C, Ar), 117.9 (1CH, Ar), 116.7 (1C, Ar), 106.6 (1CH, Ar), 105.5 (1C, Ar), 102.6 (1CH, Ar), 99.3 (1CH, Ar), 55.3 (1C, OMe), 42.7 (1C, CHH), 32.8 (1C, CH), 30.8 (1C, Me), 28.8 (1C, CHH), 18.5 (1C, CHH), 6.45 (1C, Me).). **HRMS** (FTMS) Calculated for $C_{23}H_{25}N_2O$ $[M]^+$: 345.1961. Found: 345.1961.

2,3'-BIM **3am**

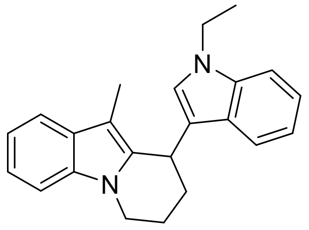

Synthesised following a modification of the general procedure: from indole **1a** (70 mg, 0.38 mmol), dry methanol (48 µl, 1.14 mmol), N-ethylindole (146 mg, 1.14 mmol), $PtCl_2$ (10.2 mg, 0.04 mmol) in 1 mL of dry 1,4-dioxane. Obtained after 3h of irradiation under microwave conditions at 130C, and isolated by column chromatography, Pet/EtOAc/DCM, (16:1:1), 77 mg, 63 % of compound **3am** as a bright orange thick oil. 1% of cyclic intermediate **4a** and 0.3% of intermediate **4a'** were detected on the reaction mixture NMR, although not isolated after column chromatography.

^{1}H NMR (500 MHz, $CDCl_3$) δ 7.66 (d, *J* = 7.5 Hz, 1H, Ar), 7.57 (d, *J* = 7.3 Hz, 1H, Ar), 7.35 (m, 2H, Ar), 7.23 (m, 2H, Ar), 7.14 (m, 2H, Ar), 6.46 (s, 1H, Ar), 4.81 (m, 1H), 4.30 (m, 1H), 4.03 (q, J = 7.2 Hz, CH2), 3.91 (m, 1H), 2.37 (m, 1H), 2.11 (m, 2H), 2.03 (s, 3H, Me), 1.91 (m, 1H), 1.34 (t, J = 7.2 Hz, Me). **^{13}C NMR** (126 MHz, $CDCl_3$) δ 136.4 (1C, Ar), 135.8 (1C, Ar), 135.1 (1C, Ar), 128.7 (1C, Ar), 126.7 (1C, Ar), 126.4 (1CH, Ar), 121.3 (1CH, Ar), 120.2 (1CH, Ar), 119.3 (1CH, Ar), 118.9 (1CH, Ar), 118.6 (1CH, Ar), 118.0 (1CH, Ar), 117.2 (1C, Ar), 109.5 (1CH, Ar), 108.6 (1CH, Ar), 105.9 (1C, Ar), 42.8 (1C, CHH), 40.7 (1C, CHH), 30.4 (1C, CH), 28.3 (1C, CHH), 19.5 (1C, CHH), 15.5 (1C, Me), 8.3 (1C, Me). **HRMS** (FTMS) Calculated for $C_{23}H_{25}N_2$ $[M]^+$: 329.2018. Found: 329.2018.

2,3'-BIM **3bb**:

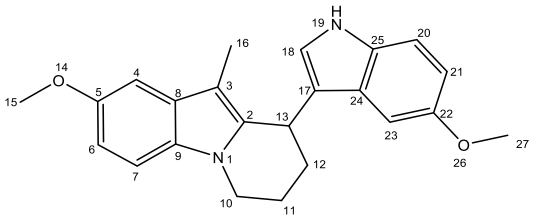

Synthesised using the general procedure from 5-methoxy-3-methyl indolyl allene (71 mg, 0.33 mmol), dry methanol (36 µl, 0.89 mmol), 5-methoxy-1H-indole (132 mg, 0.89 mmol) and $PtCl_2$ (4.4 mg, 0.016 mmol) in 1.7 mL of dry 1,4-dioxane. Obtained by column chromatography Pet/EtOAc, (20:1), 43.6 mg, 0.12 mmol, 40 % of compound **3bb** as an orange/brown solid.

^{1}H NMR (500 MHz, $CDCl_3$) δ 7.78 (s, 1H, H-***19***), 7.22 (m, 2H, H-***7*** + H-***20***), 6.98 (t, *J* = 2 Hz, 2H, H-***4*** + H-***23***), 6.85 (m, 2H, H-***6*** + H-***21***), 6.54 (d, *J* = 2 Hz, 1H, H-***18***), 4.73 (t, *J* = 4.3 Hz, 1H, H-***13***), 4.24 (ddd, *J* = 11.0, 4.9, 3.1 Hz, 1H, H-***10***), 3.87 (m, 4H, H-***10*** + H-***15***), 3.82 (s, 3H, H-***27***), 2.32 (m, 1H, H–***12***), 2.11-2.05 (m, 2H, H-***12*** + H-***11***), 1.99 (s, 3H, H-***16***), 1.90 (m, 1H, H-***11***). **^{13}C NMR** (126 MHz, $CDCl_3$) δ 153.90 (C-***5*** and C-***22***), 124.02 (CH, C-***18***), 111.94 (CH, C-***7*** or C-***20***), 111.90 (CH, C-***6*** or C-***21***), 118.97 (CH, C-***4***), 111.31 (CH, C-***7***), 110.13 (CH, C-***6*** or C-***21***), 109.24 (CH, C-***7*** or C-***20***), 100.97 (CH, C-***23*** or C-***4***), 56.08 (CH_3, C-***15***), 55.93 (CH_3, C-***27***), 42.59 (CH_2, C-***10***), 30.29 (CH, C-***13***), 27.96 (CH_2, C-***12***), 19.47 (CH_2, C-***11***), 8.37 (CH_3 – C-***16***). Some quaternary carbons not detected. **ν_{max}/cm^{-1}**: 3410 (s, NH), 3053, 2942 – 2830 (m, C-H), 1618 – 1578 (w, C=C), 1484 (s, C-H methyl), 1456 (m, C-C in ring), 1227 (s, C-N). **HRMS** (FTMS + p NSI) ((DCM) / MeOH + NH_4OAc): Calc. for $C_{23}H_{24}N_2O_2$ $[M+H]^+$: 360.1910: Found: 361.1911. **M.P.** = 180-182°C.

2,3'BIM **3ga:**

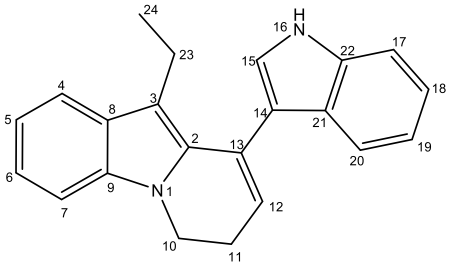

Synthesised using the general procedure from 1-(2,3-butadien-1-yl)-3-ethyl-1H-indole (109.3 mg, 0.55 mmol), $PtCl_2$ (7.4 mg, 0.03 mmol), indole (194.7 mg, 1.66 mmol), methanol (0.074 mL, 1.66 mmol) and 2.77 mL of dry 1,4-dioxane. Obtained by column chromatography, Pet/EtOAc, (20:1), 71.7 mg, 0.23 mmol, 41 % of compound **3ga** as a brown oil.

^{1}H NMR (500 MHz, $CDCl_3$) δ 7.91 (s, 1H, H***16***), 7.67 (d, *J* = 7.8 Hz, 1H, H-***4***), 7.62 (d, *J* = 7.8 Hz, 1H, H-***7***), 7.41 (d, *J* = 8.1 Hz, 1H, H-***17***), 7.37 (d, *J* = 8.1 Hz, 1H, H-***20***), 7.27 – 7.20(m, 2H, H-***5***+H-***6***), 7.19 – 7.13 (m, 2H, H-***18*** + H-***19***), 6.60 (dd, *J* = 2.3, 0.7 Hz, 1H, H-***15***), 4.86 (t, *J* = 3.7 Hz, 1H, H-***13***), 4.33 (ddd, *J* = 11.2, 5.0, 2.9 Hz, 1H, H-***10***), 3.94 (ddd, *J* = 11.2, 4.8 Hz, 1H, H-***10***), 2.55 (q, *J* = 7.5 Hz, 2H, H-***23***), 2.44 – 2.38 (m, 1H, H-***12***), 2.24 – 2.15 (m, 1H, H-***12***), 2.15 – 2.07 (m, 1H, H-***11***), 1.97 – 1.89 (m, 1H, H-***11***), 1.06 (t, *J* = 7.5 Hz, 3H, H-***24***). **^{13}C NMR** (126 MHz, $CDCl_3$) δ 136.09 (C-***22***), 134.37 (C-***2***), 123.39 (CH, C-***15***), 121.93 (CH, C-***5***/C-***6***), 120.20 (CH, C-***5***/C-***6***), 119.37 (CH, C-***18***/C-***19***), 118.97 (CH, C-***18***/C-***19***), 118.88 (CH, C-***4***), 118.41 (CH, C-***7***), 111.33 (CH, C-***17***), 108.76 (CH, C-***20***), 42.58 (N-CH_2, C-***10***), 30.21 (CH, C-***13***), 28.04 (CH_2, C-***11***), 19.30 (CH_2, C-***12***), 17.27 (CH_2, C-***23***), 14.94 (CH_2, C-***24***). Some quaternary carbons not detected.

HRMS(FTMS + p APCI, ASAP) (MeOH) Calc. for $C_{22}H_{21}N_2$ $[M\text{-}H]^+$: 313.1699. Found: 313.1699. Calc. for $C_{22}H_{23}N_2$ $[M+H]^+$: 315.1856. Found: 315.1856.

2,3'BIM **3ha** and cycle **4h'**:

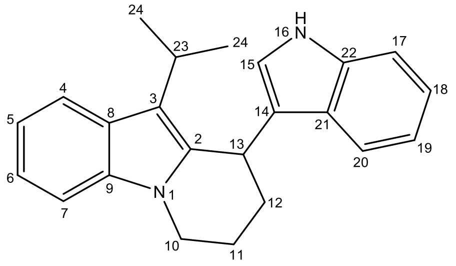

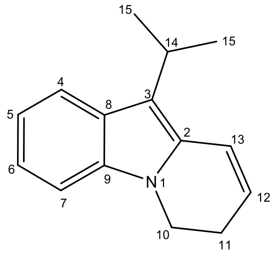

Synthesised using the general procedure from 1-(2,3-butadien-1-yl)-3-isopropyl-1H-indole (167.1 mg, 0.791 mmol), $PtCl_2$ (10.52 mg, 0.040 mmol), indole (278 mg, 2.372 mmol), methanol (0.096 mL, 2.372 mmol) and 3.95 mL of dry 1,4-dioxane. Obtained by ISCO column chromatography using 2 x 12g silica gel Redisep columns eluting with a gradient of i-hexane to 5 % ethyl acetate using the instrument's default settings to yield 45.3 mg, 0.215 mmol, 27 % of compound **4h'** as a brown oil and 18.8 mg, 0.057 mmol, 7 % of compound **3ha** as a brown oil.

Cycle 4h': **^{1}H NMR** (500 MHz, $CDCl_3$) δ 7.60 (d, *J* = 8.0 Hz, 1H, H-***4***), 7.14 (d, *J* = 8.2 Hz, 1H, H-***7***), 7.08 (ddd, *J* = 8.0, 7.0, 1.1 Hz, 1H, H-***5***), 6.95 (ddd, *J* = 8.2, 7.0, 1.1 Hz, 1H, H-***6***), 6.64 (dt, *J* = 10.0, 1.8 Hz, 1H, H-***13***), 5.87 (dt, *J* = 10.0, 4.5 Hz, 1H, H-***12***), 3.97 (t, *J* = 6.9 Hz, 2H, H-***10***), 3.21 (heptet, *J* = 7.1 Hz, 1H, H-***14***), 2.56 – 2.50 (m, 2H, H-***11***), 1.35 (d, *J* = 7.1 Hz, 6H, H-***15***). **^{13}C NMR** (126 MHz, $CDCl_3$) δ 130.13 (C-***2***), 122.94 (CH, C-***12***), 121.69 (CH, C-***5***), 120.29 (CH, C-***4***), 118.96 (CH, C-***6***), 118.66 (CH, C-***13***), 108.67 (CH, C-***7***), 39.71 (CH_2, C-***10***), 25.64 (CH, C-***14***), 24.29 (CH_2, C-***11***), 23.59 (CH_3, C-***15***). Some quaternary carbons not detected. **HRMS (**FTMS + p APCI) (DCM): Calc. for $C_{15}H_{16}N$ $[M\text{-}H]^+$: 210.1277. Found: 210.1280.

2,3'-BIM 3ha: **^{1}H NMR** (500 MHz, $CDCl_3$) δ 7.76 (s, 1H, H-***16***), 7.66 (d, *J* = 7.9 Hz, 1H, H-***4***), 7.58 (d, *J* = 7.8 Hz, 1H, H-***7***), 7.29 (d, *J* = 8.1 Hz, 1H, H-***17***), 7.25 (d, *J* = 8.1 Hz, 1H, H-***20***), 7.16 – 7.12 (m, 1H, H-***5***), 7.12 – 7.09 (m, 1H, H-***6***), 7.09 – 7.06 (m, 1H, H-***18***), 7.04 – 7.00 (m, 1H, H-***19***), 6.43 (dd, *J* = 2.3, 0.8 Hz, 1H, H-***15***), 4.79 (dd, *J* = 5.0, 2.7 Hz, 1H, H-***13***), 4.22 (ddd, *J* = 11. 5, 5.4, 1.5 Hz, 1H, H-***10***), 3.78 (td, *J* = 11.5, 4.9 Hz, 1H, H-***10***), 2.88 (heptet, *J* = 7.1 Hz, 1H, H-***23***), 2.33 – 2.27 (m, 1H, H-***12***), 2.09 – 2.03 (m, 1H, H-***12***), 2.02 – 1.91 (m, 1H, H-***11***), 1.82 – 1.75 (m, 1H, H-***11***), 1.25 (d, *J* = 7.1 Hz, 3H, H-***24***), 1.07 (d, *J* = 7.0 Hz, 3H, H-***24***). **^{13}C NMR** (126 MHz, $CDCl_3$) δ 136.67 (C-***9***), 136.53 (C-***22***), 133.51 (C-***2***), 126.50 (C-***8***), 126.11 (C-***8***), 123.55 (CH, C-***15***), 121.94 (CH, C-***5***), 120.09 (CH, C-***4***), 119.96 (CH, C-***6***), 119.49 (C-***3***), 119.38 (CH, C-***18***), 118.90 (CH, C-***7***), 118.62 (CH, C-***19***), 116.49 (C-***14***), 111.35 (CH, C-***17***), 108.97 (CH, C-***20***), 42.53 (CH_2, C-***10***), 30.11 (CH_2, C-***13***), 27.77 (CH_2, C-***11***), 25.57 (CH, C-***23***), 23.33 (CH_3, C-***24***), 22.33 (CH_3, C-***24***), 18.88 (CH_2, C-***12***). **HRMS** (FTMS + p NSI) ((MeOH) / MeOH + NH_4OAc): Calc. for $C_{23}H_{25}N_2$ $[M+H]^+$: 329.2012. Found: 329.2016.

2,3'-BIM **3ia** and cycle **4i'**

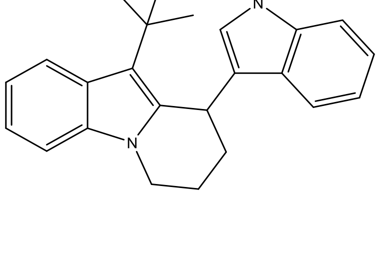

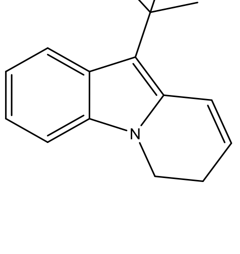

Synthesised using the general procedure from 1-(2,3-butadien-1-yl)-3-(2-methyl-2-propanyl)-1H-indole (60 mg, 0.26 mmol), $PtCl_2$ (3.5 mg, 0.013 mmol), indole (91 mg, 0.78 mmol), methanol (0.030 mL, 0.78 mmol) and 1.3 mL of dry 1,4-dioxane. Obtained column chromatography eluting, Petroleum ether/DCM/AcOEt 14:1:1, 14 mg, 0.062 mmol, 25 % of compound **4i'** as a bright yellow oil and 15 mg, 0.044 mmol, 19 % of compound **3ja** as a dark yellow oil.

Cycle 4i': **^{1}H NMR** (500 MHz, $CDCl_3$) δ = 7.78 (dd, *J*=8.2, 1.1, 1H), 7.15 (d, *J*=8.2, 1H), 7.08 (ddd, *J*=8.2, 7.0, 1.1, 1H), 7.01 – 6.92 (m, 2H), 5.88 (dt, *J*=10.4, 4.5, 1H), 3.97 (t, *J*=6.9, 2H), 2.49 (tdd, *J*=6.7, 4.5, 1.8, 2H), 1.49 (s, 9H). **^{13}C NMR** (126 MHz, $CDCl_3$) δ = 137.13, 129.95, 127.33, 123.43, 122.38, 121.71, 121.62, 118.54, 108.73, 100.10, 39.91, 34.20, 32.57, 24.04. **HRMS** (FTMS + p APCI, ASAP) (MeOH) Calc. for $C_{16}H_{20}N$ $[M+H]^+$: 226.1590. Found: 226.1586.

2,3'-BIM 3ia: **^{1}H NMR** (500 MHz, $CDCl_3$) δ = 7.89 (dt, *J*=8.1, 0.9, 1H), 7.65 (d, *J*=7.7, 1H), 7.36 (dt, *J*=8.1, 0.9, 1H), 7.31 (dt, *J*=8.2, 0.9, 1H), 7.24 – 7.14 (m, 3H), 7.10 (ddd, *J*=8.2, 7.0, 1.2, 1H), 6.38 (dd, *J*=2.4, 1.0, 1H), 5.37 – 5.08 (m, 0H), 4.35 (dd, *J*=11.7, 6.0, 1H), 3.83 (ddd, *J*=12.5, 11.6, 5.3, 1H), 2.43 – 2.35 (m, 1H), 2.13 (tdd, *J*=13.0, 4.6, 2.6, 1H), 2.01 (dddt, *J*=16.3, 13.3, 8.8, 2.6, 1H), 1.84 – 1.72 (m, 1H), 1.40 (s, 9H). **^{13}C NMR** (126 MHz, $CDCl_3$) δ = 136.98 (C), 136.78 (C), 133.56 (C), 127.29 (C), 125.79 (C), 124.29 (C), 122.05 (CH), 121.97 (CH), 121.00 (C), 120.24 (CH), 119.49 (CH), 118.93 (CH), 118.57 (CH), 118.30 (C), 111.41 (CH), 109.03 (CH), 43.07 (CH_2), 33.93 (C), 31.79 (CH), 31.46 (3 x CH_3), 27.53 (CH_2), 18.18 (CH_2). **HRMS** (FTMS + p APCI, ASAP) (MeOH) Calc. for $C_{24}H_{25}N_2$ $[M-H]^+$: 341.2012. Found: 343.2008. Calc. for $C_{24}H_{27}N_2$ $[M+H]^+$: 343.2169. Found: 343.2167.

2,3'-BIM **3ja**

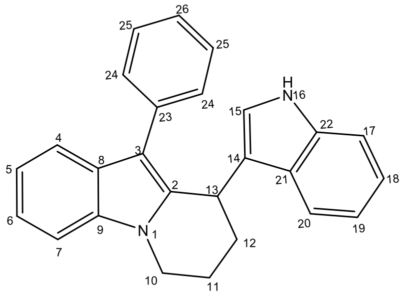

Synthesised using the general procedure from 1-(2,3-butadien-1-yl)-3-phenyl-1H-indole (233 mg, 0.950 mmol), $PtCl_2$ (13 mg, 0.047 mmol), indole (334 mg, 2.85 mmol), methanol (0.115 mL, 2.85 mmol) and 4.75 mL of dry 1,4-dioxane. Obtained by ISCO column chromatography using an 12g silica gel Redisep column eluting with a gradient of i-hexane to 2.5% ethyl acetate using the instrument's default settings to yield traces (<5%) of compounds **4j** and **4j'** as an inseparable mixture (1:1) as a brown oil and 39.1 mg, 0.108 mmol, 11 % of compound **3ja** as a brown oil.

2,3'-BIM 3ja: **^{1}H NMR** (500 MHz, $CDCl_3$) δ 7.93 (s,1H, H-***16***), 7.82 (d, *J* = 7.8 Hz, 1H, H-***17***), 7.62 (d, *J* = 8.2 Hz, 1H, H-***20***), 7.42 – 7.38 (m, 4H, H-***25***), 7.31 – 7.28 (m, 1H, H-***26***), 7.27 – 7.20 (m, 4H, H-***4*** +H-***7***), 7.18 – 7.12 (m, 2H, H-18 + H-***19***), 6.63 (d, *J* = 1.6 Hz, 1H, H-***15***), 4.92 (t, *J* = 3.7 Hz, 1H, H-***13***), 4.45 (ddd, *J* = 11.6, 5.8, 1.9 Hz, 1H, H-***10***), 3.98 (td, *J* = 11.6, 5.5 Hz, 1H, H-***10***), 2.47 – 2.41 (m, 1H, H-***12***), 2.24 – 2.17 (m, 1H, H-***12***), 2.17 – 2.09 (m, 1H, H-***11***), 1.99 – 1.92 (m, 1H, H-***11***). **^{13}C NMR** (126 MHz, $CDCl_3$) δ 136.79 (C-***9***), 136.69 (C-***22***), 136.26 (C-***2***), 135.40 (C-***23***), 128.77 (C-***Ar***), 128.28 (C-***Ar***), 127.41 (C-***Ar***), 125.67 (C-***Ar***), 125.29 (C-***Ar***), 123.29 (C-***Ar***), 123.97 (CH, CH=C, C-***15***), 121.98 (C-***Ar***), 121.07 (C-***Ar***), 120.20 (C-***Ar***), 119.73 (C-***Ar***), 119.40 (C-***Ar***), 119.34 (CH, C-***17***), 119.10 (CH, C-***20***), 112.41 (C-***26***), 111.30 (CH, C-***25***), 109.20, (CH, C-

24), 42.88 (N-CH_2, C-***10***), 30.69 (CH, C-***13***), 27.38 (CH_2, C-***11***), 18.44 (CH_2, C-***12***). **HRMS** (FTMS + p NSI) ((DCM) / MeOH + NH_4OAc): Calc. for $C_{26}H_{23}N_2$ $[M+H]^+$ 363.1856: Found: 363.1857.

Trisindole **5ka** Allyl indole **6ka**

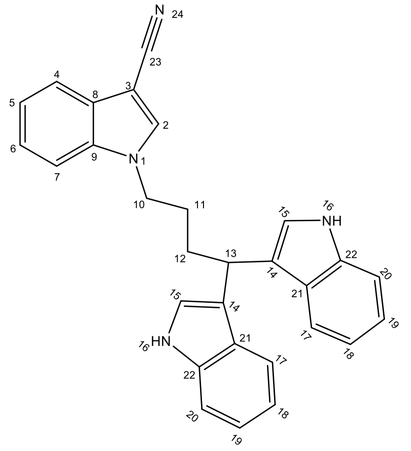

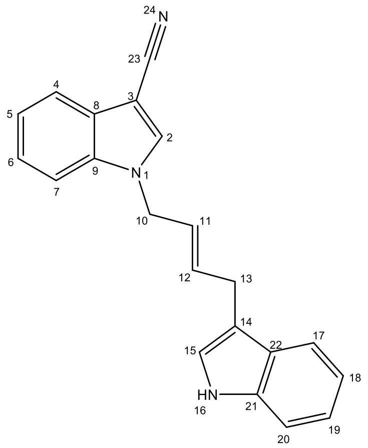

Synthesised using the general procedure from 1-(2,3-butadien-1-yl)-3-carbonitrile-1H-indole (140.2 mg, 0.722 mmol), $PtCl_2$ (9.60 mg, 0.036 mmol), indole (254 mg, 2.165 mmol), methanol (0.088 mL, 2.165 mmol) and 3.61 mL of dry 1,4-dioxane. Obtained by ISCO column chromatography using a 12g silica gel Redisep column eluting with a gradient of i-hexane to 40% ethyl acetate using the instrument's default settings to yield 33 mg, 0.106 mmol, 15 % of compound **6ka** as a brown oil and 128 mg, 0299 mmol, 41 % of compound **5ka** as a brown oil.

<u>Trisindole 5ka</u>: **^{1}H NMR** (500 MHz, $CDCl_3$) δ 7.85 (s, 2H, H-***16***), 7.67 – 7.63 (m, 1H, H-***2***), 7.42 (d, *J* = 8.0 Hz, 2H, H-***17***), 7.23 (d, *J* = 8.1 Hz, 2H, H-***20***), 7.19 – 7.12 (m, 2H, H-***4*** + H-***7***), 7.09 – 7.04 (m, 2H, H-***18***), 6.97 – 6.91 (m, 2H, H-***19***), 6.77 (d, *J* = 2.2 Hz, 2H, H-***15***), 4.38 (t, *J* = 7.5 Hz, 1H, H-***13***), 3.98 (t, *J* = 7.1 Hz, 2H, H-***10***), 2.13 (dt, *J* = 15.4, 7.6 Hz, 2H, H-***12***), 1.87 (dt, *J* = 15.7, 7.4 Hz, 2H, H-***11***). **^{13}C NMR** (126 MHz, $CDCl_3$) δ 136.60 (C-***9***), 135.36 (C-***22***), 134.70, 127.91 (C-***8***), 126.81 (C-***21***), 123.69 (CH, C-***4*** +C-***7***), 122.03 (CH, C-***4*** and C-***7*** and C-***18***), 121.45 (CH, CH=C, C-***15***), 119.91 (CH, C-***2***), 119.28 (CH, C-***17***), 119.25 (CH, C-***19***), 111.30 (CH, C-***20***), 110.62 (CH, C-***4*** +C-***7***), 47.19 (N-CH_2, C-***10***), 33.77 (CH, C-***13***), 32.59 (CH_2, C-***12***), 28.41 (CH_2, C-***11***). Quaternary carbons detected. **HRMS** (FTMS + p NSI) ((MeOH) / MeOH + NH_4OAc): Calc. for $C_{29}H_{25}N_4$ $[M+H]^+$: 429.2074. Found: 429.2080.

<u>Allyl indole 6ka:</u> **^{1}H NMR** (500 MHz, $CDCl_3$) δ 7.93 (s, 1H, H-***16***), 7.69 (m, 1H, H-***4***), 7.52 (s, 1H, H-***2***), 7.45 (dd, *J* = 7.9, 0.9 Hz, 1H, H-***7***), 7.35 – 7.28 (m, 2H, H-***17***+ H-***20***), 7.23 (dtd, *J* = 14.2, 7.1, 1.3 Hz, 2H, H-***5***+ H-***6***), 7.13 (ddd, *J* = 8.2, 7.1, 1.1 Hz, 1H, H-***18***), 7.04 (ddd, *J* = 8.0, 7.1, 1.0 Hz, 1H, H-***19***), 6.87 (d, *J* = 2.3 Hz, 1H, H-***15***), 5.91 (dtt, *J* = 15.4, 6.2, 1.4 Hz, 1H, H-***12***), 5.65 (dtt, *J* = 15.4, 6.1, 1.6 Hz, 1H, H-***11***), 4.65 (dd, *J* = 6.2, 1.2 Hz, 2H, H-***10***), 3.47 (d, *J* = 6.1, 1.2 Hz, 2H, H-***13***). **^{13}C NMR** (126 MHz, $CDCl_3$) δ 135.05 (CH, CH=C, C-***11***), 134.46 (CH, C-***2***), 123.96 (CH, CH=C, C-***12***), 123.77 (CH, C-***5***/C-***6)***, 122.20 (CH, C-***5***/C-***6***), 122.24 (CH, C-***18***), 121.75 (CH, CH=C, C-***15***), 119.91 (CH, C-***4***), 119.46 (CH, C-***19***), 119.16 (CH, C-***17***), 119.19 (CH, C-***20***), 110.90 (CH, C-***7***), 48.77 (CH_2, C-***10***), 28.05 (CH_2, CH_2, C-***13***). Quaternary carbons not detected. **HRMS** (FTMS + p NSI) ((DCM) / MeOH + NH_4OAc): Calc. for $C_{21}H_{17}N_3$ $[M+H]^+$: 312.1495. Found: 312.1498.

Trisindole **5la**

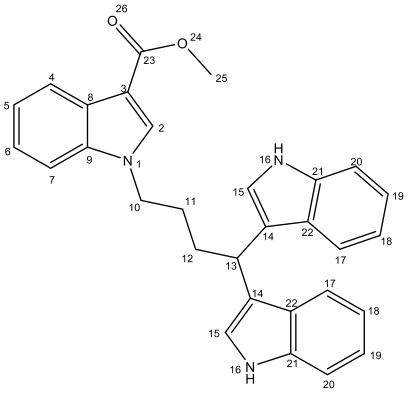

Allyl indole **6la**

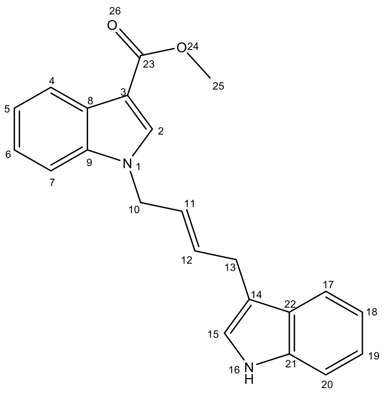

Synthesised using the general procedure from 1-(2,3-butadien-1-yl)-3-carboxylate-1H-indole (210.1 mg, 0.924 mmol), $PtCl_2$ (12 mg, 0.046 mmol), indole (325 mg, 2.77 mmol), methanol (0.112 mL, 2.77 mmol) and 4.75 mL of dry 1,4-dioxane. Obtained by ISCO column chromatography using a 12g silica gel Redisep column eluting with a gradient of i-hexane to 100 % ethyl acetate using the instrument's default settings to yield 39.7 mg, 0.115 mmol, 13 % of compound **6la** as a brown oil and 136.9 mg, 0.297 mmol, 32 % of compound **5la** as a brown oil.

Trisindole 5la: ^{1}**H NMR** (500 MHz, $CDCl_3$) δ 8.08 (d, *J* = 4.9, 3.8 Hz, 1H, H-***4***), 7.81 (s, 2H, H-***16***), 7.63 (s, 1H, H-***2***), 7.41 (d, *J* = 8.0 Hz, 2H, H-***17***), 7.19 (d, *J* = 8.1 Hz, 2H, H-***20***), 7.16 – 7.14 (m, 1H, H-***7***), 7.12 – 7.10 (m, 2H, H-***5*** + H-***6***), 7.07 – 7.03 (m, 2H, H-***19***), 6.94 – 6.90 (m, 2H, H-***18***), 6.69 (d, *J* = 2.2 Hz, 2H, H-***15***), 4.35 (t, *J* = 7.5 Hz, 1H, H-***13***), 3.97 (t, *J* = 7.1 Hz, 2H, H-***10***), 3.81 (s, 3H, H-***25***), 2.10 (m, 2H, H-***12***), 1.86 (m, 2H, H-***11***). ^{13}**C NMR** (126 MHz, $CDCl_3$) δ 165.7 (C-***23***), 136.59 (C-***9***), 136.54 (C-***21***), 134.38 (CH, C-***2***), 126.87 (C-***8***), 126.71 (C-***22***), 122.67 (CH, C-***4***), 121.91 (CH, C-***19***), 121.84 (CH, C-***7***), 121.74 (CH, C-***5*** or C-***6***), 121.52(CH, C-***15***), 119.39 (CH, C-***17***), 119.18 (CH, C-***18***), 111.25 (CH, C-***20***), 110.15 (CH, C-***5*** or C-***6***), 106.76 (C-***3***), 51.02 (CH_3, C-***25***), 46.99 (CH_2, C-***10***), 33.72 (CH_2, C-***13***), 32.65 (CH_2, C-***12***), 28.38 (CH_2, C-***11***). **HRMS** (FTMS + p NSI) ((MeOH) / MeOH + NH_4OAc): Calc. for $C_{30}H_{27}N_3O_2Na$ $[M+Na]^+$: 484.1995. Found: 484.1989.

Allyl indole 6la: ^{1}**H NMR** (500 MHz, $CDCl_3$) δ 8.10 (m, 1H, H-***4***), 7.96 (s, 1H, H-***16***), 7.74 (s, 1H, H-***2***), 7.45 (m, 1H, H-***17***), 7.28 (m, 1H, H-***7***), 7.26 (d, *J* = 8.1 Hz, 1H, H-***20***), 7.20 – 7.17 (m, 2H, H-***5*** + H-***6***), 7.13 – 7.08 (m, 1H, H-***19***), 7.04 – 6.99 (m, 1H, H-***18***), 6.82 (d, *J* = 2.2 Hz, 1H, H-***15***), 5.86 (dtt, *J* = 15.3, 6.3, 1.2 Hz, 1H, H-***12***), 5.64 (dtt, *J* = 15.2, 6.1, 1.5 Hz, 1H, H-***11***), 4.61 (dd, *J* = 6.1, 1.0 Hz, 2H, H-***10***), 3.43 (dd, *J* = 6.4, Hz, 2H, H-***13***), 3.82 (s, 3H, H-***25***). ^{13}**C NMR** (126 MHz, $CDCl_3$) δ 165.6 (C-***23***), 136.6 (C-***9***), 136.4 (C-***21***), 134.3 (CH, C-***11***), 134.15 (CH, C-***2***), 127.2 (C-***8***), 126.8 (C-***22***), 124.5(CH, C-***12***), 122.7 (CH, C-***5*** or C-***6***), 122.1 (CH, C-***19***), 121.9 (CH, C-***5*** or C -***6***), 121.9 (CH, CHC, C-***15***), 121.7 (CH, C-***4***), 119.4 (CH, C-***18***), 118.9 (CH, C-***17***), 113.7 (C-***14***), 111.22 (CH, C-***20***), 110.3 (CH, C-***7***), 107.1 (C-***3***), 51.0 (CH_3, C-***25***) , 48.7 (N-CH_2 C-***10***), 28.2 (CH_2, C-***13***). **HRMS** (FTMS + p NSI) ((MeOH) / MeOH + NH_4OAc): Calc. for $C_{22}H_{21}N_2O_2$ $[M+H]^+$: 345.1598. Found: 345.1601.

Trisindole **5ma**

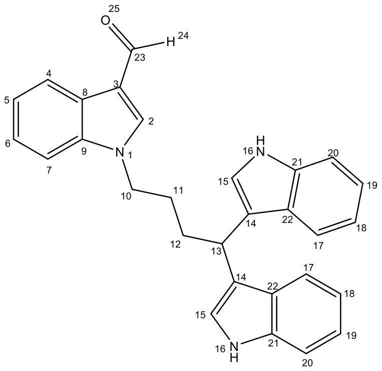

Allyl indole **6ma**

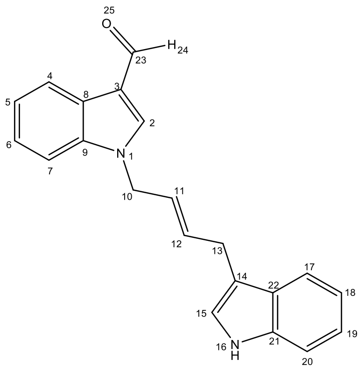

Synthesised using the general procedure from 1-(2,3-butadien-1-yl)-3-formyl-1H-indole (44.5 mg, 0.226 mmol), $PtCl_2$ (3.00 mg, 0.011 mmol), indole (79 mg, 0.677 mmol), methanol (0.027 mL, 0.677 mmol) and 1.1 mL of dry 1,4-dioxane. Obtained by ISCO column chromatography using a 12g silica gel Redisep columns eluting with a gradient of i-hexane to 40 % ethyl acetate using the instrument's default settings to yield 3.6 mg, 0.011 mmol, 5 % of compound **6ma** as a brown oil and 4.6 mg, 0.011 mmol, 5 % of compound **5ma** as a brown oil.

Trisindole 5ma: ^{1}**H NMR** (500 MHz, $CDCl_3$) δ 9.95 (s, 1H, H-***24***), 8.33 (d, *J* = 6.9, 1.3 Hz, 1H, H-***4***), 7.94 (s, 2H, H-***16***), 7.58 (s, 1H, H-***2***), 7.55 (d, *J* = 7.9 Hz, 1H, H-***17***), 7.37 (d, *J* = 8.2 Hz, 1H, H-***20***), 7.35 – 7.30 (m, 2H, H-***5***+H-***6***), 7.27 (dd, *J* = 8.4, 6.9 Hz, 1H, H-***7***), 7.20 (ddd, *J* = 8.1, 7.0 Hz, 2H, H-***19***), 7.06 (ddd, *J* = 8.0, 7.1, 0.9 Hz, 2H, H-***18***), 6.92 (d, *J* = 2.2 Hz, 2H, H-***15***), 4.54 (t, *J* = 7.5 Hz, 1H, H-***13***), 4.19 (t, *J* = 7.1 Hz, 2H, H-***10***), 2.31 (m, 2H, H-***12***), 2.09 (m, 2H, H-***11***). ^{13}**C NMR** (126 MHz, $CDCl_3$) 138.35 (CH), 123.72 (CH), 123.01 (CH), 122.18 (CH), 122.10 (CH), 121.47 (CH), 119.42 (CH), 119.34 (CH), 111.41 (CH), 110.07 (CH), 47.44 (CH_2), 33.94 (CH), 32.58 (CH_2), 28.50 (CH_2). Quaternary carbons not detected. **HRMS** (FTMS + p NSI) ((MeOH)/ MeOH + NH_4OAc): Calc. for $C_{29}H_{26}N_3O$ $[M+H]^+$: 432.2070. Found: 432.2070

Allyl indole 6ma: ^{1}**H NMR** (500 MHz, $CDCl_3$) δ 10.0 (s, 1H, H-***24***), 8.34 (dd, *J* = 5.9, 3.0 Hz, 1H, H-***4***), 8.04 (s, 1H, H-***16***), 7.74 (s, 1H, H-***2***), 7.58 (d, *J* = 7.9 Hz, 1H, H-***17***), 7.44 – 7.38 (m, 2H, H-***7*** + H-***20***), 7.35 (dd, *J* = 6.2, 3.0 Hz, 2H, H-***5*** + H-***6***), 7.24 (t, *J* = 7.5 Hz, 1H, H-***19***), 7.14 (t, *J* = 7.5 Hz, 1H, H-***18***), 6.99 (d, *J* = 1.5 Hz, 1H, H-***15***), 6.05 (dt, *J* = 13.8, 6.2 Hz, 1H, H-***11***), 5.80 (dt, *J* = 13.8, 6.2 Hz, 1H, H-***12***), 4.78 (d, *J* = 6.2 Hz, 2H, H-***10***), 3.59 (d, *J* = 6.2 Hz, 2H, H-***13***). ^{13}**C NMR** (126 MHz, $CDCl_3$) 184.59 (C-***23***), 140.19, 138.06 (CH, C-***2***), 135.11 (CH, C-***11***), 124 (CH, C-***5***/C-***6***), 123.95 (CH, C-***12***), 122.97 (CH, C-***5*** or C-***6***), 122.26 (CH, C-***4***), 121.82 (CH, C-***19***), 121.79 (CH, C-***15***), 119.48 (CH, C-***18***), 119.23 (C-***3***), 118.91 (CH, C-***17***), 111.25 (CH, C-***7*** or C-***20***), 110.32 (CH, C-***7*** or C-***20***), 48.87 (CH_2, N-CH_2 C-***10***), 28.22 (CH_2, C-***13***), 27.97. Quaternary carbons not detected. **HRMS** (FTMS + p NSI) ((MeOH)/ MeOH + NH_4OAc): Calc. for $C_{21}H_{18}N_2ONa$ $[M+Na]^+$: 337.1311. Found: 337.1315.

Trisindole **5na**

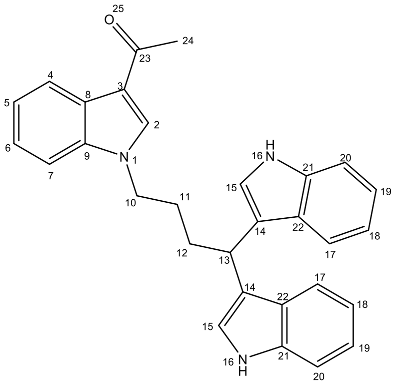

Allyl indole **6na**

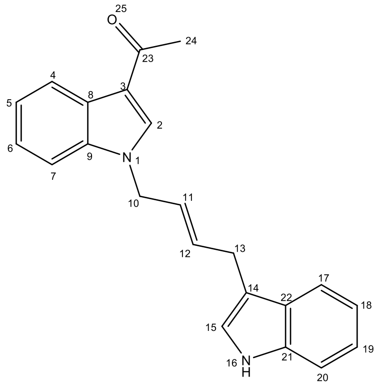

Synthesised using the general procedure from 1-(2,3-butadien-1-yl)-3-ethanoate-1H-indole (213.3 mg, 1.010 mmol), $PtCl_2$ (13 mg, 0.05 mmol), indole (335 mg, 3.03 mmol), methanol (0.123 mL, 3.03 mmol) and 4.75 mL of dry 1,4-dioxane. Obtained by ISCO column chromatography using a 12g silica gel Redisep column eluting with a gradient of i-hexane to 100 % ethyl acetate using the instrument's default settings to yield 36.4 mg, 0.111 mmol, 11 % of compound **6na** as a brown oil and 103.4 mg, 0.232 mmol, 23 % of compound **5na** as a brown oil.

Trisindole 5na: **^{1}H NMR** (500 MHz, $CDCl_3$) δ 8.41 (m, 1H, H-***4***), 7.94 (s, 1H, H-***16***), 7.63 (s, 1H, H-***2***), 7.55 (d, *J* = 8.1 Hz, 2H, H-***17***), 7.37 (d, *J* = 8.1 Hz, 2H, H-***20***), 7.32 (dd, *J* = 5.9, 2.1 Hz, 1H, H-***7***), 7.28 – 7.25 (m, 2H, H-***5*** + H-***6***), 7.22 – 7.17 (m, 2H, H-***19***), 7.09 – 7.04 (m, 2H, H-***18***), 6.90 (d, *J* = 2.2 Hz, 2H, H-***15***), 4.53 (t, *J* = 7.5 Hz, 1H, H-***13***), 4.17 (t, *J* = 7.1 Hz, 2H, H-***10***), 2.49 (s, 3H, H-***24***), 2.30 (m, 2H, H-***12***), 2.12 - 2.04 (m, 2H, H-***11***). **^{13}C NMR** (126 MHz, $CDCl_3$) δ 193.2 (C-***23***), 136.84 (C-***9***), 136.61 (C-***21***), 134.99 (CH, C-***2***), 126.85 (C-***8***), 126.37 (C-***22***), 122.61 (CH, C-***4***), 121.95 (CH, C-***19***), 121.54 (CH, C-***15***), 119.37 (CH, C-***17***), 119.20 (CH, C-***18***), 111.30 (CH, C-***20***), 109.98 (CH, C-***7***), 47.04 (N-CH_2, C-***10***), 33.78 (CH_2, C-***13***), 32.67 (CH_2, C-***12***), 28.41 (CH_2, C-***11***), 27.63 (CH_3, C-***24***). **HRMS** (FTMS + p NSI) ((MeOH) / MeOH + NH_4OAc): Calc. for $C_{22}H_{21}N_2O$ $[M+H]^+$: 329.1648. Found: 329.1652.

Allyl indole 6na: **^{1}H NMR** (500 MHz, $CDCl_3$) δ 8.30 (m, 1H, H-***4***), 7.96 (s, 1H, H-***16***), 7.64 (s, 1H, H-***2***), 7.47 (m, 1H, H-***17***), 7.29 (m, 1H, H-***6*** + H-***7***), 7.23 – 7.19 (m, 2H, H-5+ H-***20***), 7.15 – 7.10 (m, 1H, H-***19***), 7.03 (ddd, *J* = 7.9, 7.1, 0.9 Hz, 1H, H-***18***), 6.87 (d, *J* = 2.2 Hz, 1H, H-***15***), 5.92 (dtt, *J* = 15.3, 6.3, 1.3 Hz, 1H, H-***12***), 5.69 (dtt, *J* = 15.3, 6.1, 1.5 Hz, 1H, H-***11***), 4.65 (dd, *J* = 6.1, Hz, 2H, H-***10***), 3.47 (d, *J* = 6.3, Hz, 2H, H-***13***), 2.41 (s, 3H, H-***24***). **^{13}C NMR** (126 MHz, $CDCl_3$) δ 193.15 (C-***23***), 136.89 (C-***9***), 136.45 (C-***21***), 134.70 (CH, C-***12***), 134.60 (CH, C-***2***), 127.22 (C-***8***), 126.47 (C-***22***), 124.32 (CH, C-***11***), 122.63 (CH, C-***5*** + C-***20***), 122.59 (CH, C-***4***), 122.20 (CH, C-***19***), 121.84 (CH, CH=C, C-***15***), 119.46 (CH, C-***18***), 118.89 (CH, C-***17***), 117.14 (C-***3***), 113.63 (C-***14***), 111.26 (CH, C-***6*** or C-***7***), 110.09 (CH, C-***6*** or C-***7***), 48.70 (N-CH_2, C-***10***), 28.22 (CH_2, C-***13***), 27.62 (CH_3, C-***24***). **HRMS** (FTMS + p NSI) ((MeOH) / MeOH + NH_4OAc): Calc. for $C_{30}H_{28}N_3O$ $[M+H]^+$: 446.2227. Found: 446.2226.

Trisindole **5oa** Allyl indole **6oa**

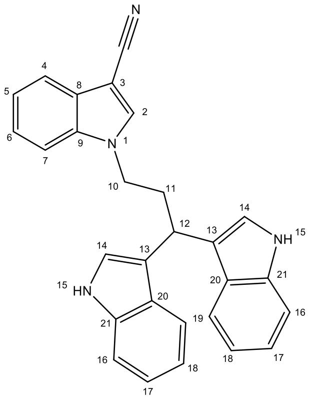

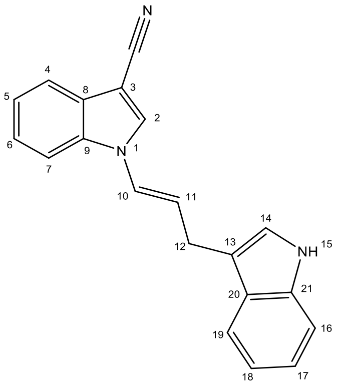

Synthesised using the general procedure from 3-cyanoindolyl allene (50 mg, 0.28 mmol), $PtCl_2$ (3.7 mg, 0.014 mmol), indole (98 mg, 0.84 mmol) and dry methanol (22 µl, 0.84mmol) in 2 mL of dry 1,4-dioxane (0.15 M). Obtained by column chromatography, Pet/EtOAc (50:1) 47.2 mg, 0.11 mmol, 41 % of product **6oa** as brown/orange liquid and 22.7 mg, 0.076 mmol, 27 % of product **5oa** as an orange/brown solid.

Trisindole 5oa: ^{1}**H NMR** (500 MHz, Acetone) δ 10.06 (s, 2H, H-***15***), 8.00 (s, 1H, H-***2***), 7.69 – 7.65 (m, 1H, H-***7***), 7.49 (d, *J* = 8.0 Hz, 2H, H-***16***), 7.40 (dd, *J* = 7.0, 1.0 Hz, 1H, H-***4***), 7.37 (d, *J* = 8.1 Hz, 2H, H-***19***), 7.32 (d, *J* = 1.5 Hz, 2H, H-***14***), 7.29 – 7.22 (m, 2H, H-***6*** and H-***5***), 7.07 – 7.01 (m, 2H, H-***18***), 6.90 (ddd, *J* = 8.0, 7.1, 1.0 Hz, 2H, H-***17***), 4.56 (t, *J* = 7.6 Hz, 1H, H-***12***), 4.46 – 4.37 (m, 2H, H-***10***), 2.88 (q, *J* = 7.6 Hz, 2H, H-***11***). ^{13}**C NMR** (126 MHz, $CDCl_3$) δ 136.82 (C), 135.42 (C), 135.12 (2CH, C-***16***), 128.17 (C), 126.63 (C), 123.82 (CH, C-***4***), 122.39 (2CH, C-***18***), 122.23 (CH, C-***6***), 121.76 (2CH, C-***14***), 120.13 (CH, C-***7***), 119.64 (2CH, C-***17***), 119.37 (CH, C-***2***), 118.50 (C), 116.08 (C), 111.48 (2CH, C-***19***), 110.85 (CH, C-***4***), 85.68 (C), 46.19 (CH_2, C-***10***), 35.38 (CH_2, C-***11***), 31.73 (CH, C-***12***). **HRMS:** Calculated for $C_{28}H_{22}N_4$ $[M+H]^+$: 415.1918 Found: 415.1917.

Allyl indole 6oa: ^{1}**H NMR** (500 MHz, $CDCl_3$) δ 8.06 (s, 1H, H-***15***), 7.81 (s, 1H, H-***2***), 7.75 (d, *J* = 7.5 Hz, 1H, H-***7***), 7.64 (d, *J* = 8.0 Hz, 1H, H-***4***), 7.46 – 7.40 (m, 2H, H-***19*** and H-***16***), 7.36 – 7.32 (m, 1H, H-***17***), 7.32 – 7.29 (m, 1H, H-***6***), 7.26 – 7.22 (m, 1H, H-***18***), 7.15 (ddd, *J* = 8.0, 7.3, 1.0 Hz, 1H, H-***5***), 7.10 (d, *J* = 2.3 Hz, 1H, H-***14***), 7.03 (dt, *J* = 3.1, 1.6 Hz, 1H, H-***10***), 6.26 – 6.20 (m, 1H, H-***11***), 3.75 (ddd, *J* = 6.6, 1.6, 1.0 Hz, 2H, H-***12***). ^{13}**C NMR** (126 MHz, $CDCl_3$) δ 136.66 (C), 134.92 (C), 131.69 (CH, C-***2***), 127.83 (C), 127.23 (C), 124.62 (CH, C-***17***), 123.78 (CH, C-***10***), 122.95 (CH, C-***6***), 122.55 (CH, C-***18***), 122.18 (CH, C-***14***), 120.95 (CH, C-***11***), 120.13 (CH, C-***7***), 119.82 (CH, C-***5***), 118.96 (CH, C-***4***), 115.63 (C), 113.32 (C), 111.54 (CH, C-***19***), 110.91 (CH, C-***16***), 88.23 (C), 26.16 (CH_2, C-***12***). **HRMS** (FTMS + p NSI) (DCM/ MeOH + NH_4OAc): Calc. for $C_{20}H_{15}N_3$ $[M+H]^+$: 298.1341 Found: 298.1339.

Cycle **4p'** Cycle **4p**

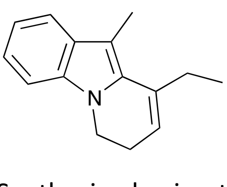

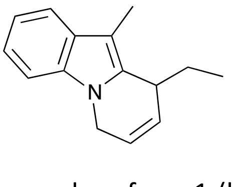

Synthesised using the general procedure from 1-(hexa-4,5-dien-1-yl)-3-methyl-1H-indole **1p** (330 mg, 1.5 mmol), platinum (II) chloride (20 mg, 0.075), indole (550 mg, 4.7 mmol), dry methanol (190 µL, 4.7 mmol) and 10 mL (0.15 M) of dry 1,4-dioxane. Obtained by column chromatography eluting with a gradient of 0-10% ethyl acetate in hexane, 165 mg, 0.78 mmol, 52 % of compounds **4p'** and **4p** as an inseparable mixture (1:0.1) as a brown oil.

Cycle 4p': ^{1}**H NMR** (500 MHz, $CDCl_3$) δ = 7.57 (dt, *J* = 7.8, 0.9, 1H), 7.29 – 7.18 (m, 2H), 7.09 (ddd, *J* = 8.0, 6.6, 1.4, 1H), 5.77 (tt, *J* = 4.8, 1.4 Hz 1H), 4.04 (t, *J* = 7.0, 2H), 2.67 (m, 2H), 2.59 – 2.52 (m, 2H), 2.51 (s, 3H), 1.23 (t, *J* = 7.0, 3H). ^{13}**C NMR** (126 MHz, $CDCl_3$) δ = 136.04 (C), 135.50 (C), 131.83 (C), 129.56 (C), 122.02 (CH), 119.29 (CH), 118.77 (CH), 118.71 (CH), 108.44 (CH), 106.71 (C), 39.92 (CH_2), 26.79 (CH_2), 24.45 (CH_2), 13.87 (CH_3), 10.35 (CH_3). **HRMS** (FTMS + p APCI, ASAP) (MeOH) Calc. for $C_{15}H_{18}N$ $[M+H]^+$: 212.1434. Found: 212.1429.

Compound 3an

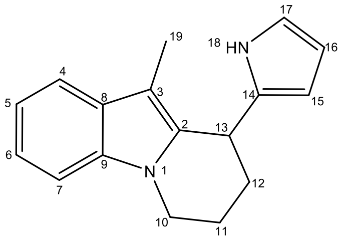

Synthesised using the general procedure from 3-methyl indolyl allene (100 mg, 0.55 mmol), $PtCl_2$ (7.3 mg, 0.0275 mmol), pyrrole (111 mg, 1.65 mmol) and dry methanol (67 µl, 1.65 mmol) in 2.75 mL of dry 1,4-dioxane. Obtained by column chromatography, Pet/EtOAc, 20:1, 89.8 mg, 0.36 mmol, 65 % of compound **3an** as a brown solid compound.

^{1}**H NMR** (500 MHz, $CDCl_3$) δ 7.77 (bs, 1H, H-***18***), 7.59 (d, *J* = 7.8 Hz, 1H, H-***4***), 7.32 (d, *J* = 8.1 Hz, 1H, H-***7***), 7.24 (m, 1H, H-***6***), 7.18 (m, 1H, H-***5***), 6.58 (dd, *J* = 4.0, 2.5 Hz, 1H, H-***17***), 6.22 (dd, *J* = 5.8, 2.7 Hz, 1H, H-***16***), 6.04 (bs,1H, H-***15***), 4.55 (t, *J* = 4.5 Hz, 1H, H-***13***), 4.20 (dt, *J* = 11.2, 4.7 Hz, 1H, H-***10***), 3.91 (m, 1H, H-***10***), 2.26 (m, 1H, H-***12***), 2.18 (m, 1H, H-***12***), 2.14 (s, 3H, H-***19***), 2.08 (m, 1H, H-***11***), 2.00 (m, 1H, H-***11***). ^{13}**C NMR** (126 MHz, $CDCl_3$) δ 136.2 (C-***3***), 133.5 (C-***2***), 132.9 (C-***4***), 128.6 (C-***9***), 121.0 (CH, Ar, C-***6***), 119.3 (CH, Ar, C-***5***), 118.3 (CH, Ar, C-***4***), 116.0 (CH, CH=C, C-***17***), 108.9 (CH, Ar, C-***7***), 108.8 (CH, Ar, C-***16***), 104.7 (CH, CH=C, C-***15***), 42.5 (CH_2, C-***10***), 32.2 (CH, C-***13***), 29.5 (CH_2, C-***12***), 20.0 (CH_2, C-***11***), 8.25 (CH_3, C-***19***). ν_{max}**/cm^{-1}:** 3407 (m, br, N-H), 2930 (s, C-H), 2860 (m, C-H), 1695, 1667, 1462, 1360 (m, C-N), 739. **HRMS** (FTMS + p NSI) (DCM/ MeOH + NH_4OAc): Calculated for $C_{17}H_{18}N_2$ $[M+H]^+$: 251.1545. Found: 251.1543.

Dimers

In reactions with external indoles (entries 8, 10, 11 and 12, Table 1) and in the reaction with the ethyl derivative in C3 of the indolylallene (entry 2, Table 2), small amounts (3-10 %) of a dimer were isolated (see tables 1 and 2 in main manuscript). The structure of the dimers was confirmed by HRMS and tentatively assigned by NMR analysis (^{1}H, ^{13}C, COSY, HSQC and HMBC)

Dimer **7a**

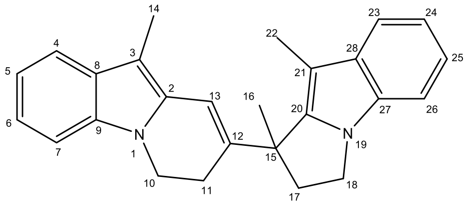

^{1}**H NMR** (500 MHz, $CDCl_3$) δ 7.58 – 7.50 (m, 2H) 8H 7.30 – 7.04 (m, 6H), 6.65 (s, 1H, H-***13***), 4.18 – 3.92 (m, 4H, H-***10*** and H-***18***), 2.83 – 2.41 (m, 4H, H-***11*** and H-***17***), 2.32 (s, 3H, H-***22***), 2.20 (s, 3H, H-***14***), 1.69 (s, 3H, H-***16***). ^{13}**C NMR** (126 MHz, $CDCl_3$) δ 144.96 (C_q), 138.98 (C_q), 136.39 (C_q), 133.44 (C_q), 132.19 (C_q), 131.77 (C_q), 129.58 (C_q), 121.95 (C-***Ar***), 120.67 (C-***Ar***), 119.03 (C-***Ar***), 118.84 (C-***Ar***), 118.77 (C-***Ar***), 118.74 (C-***Ar***), 112.82 (C-***13***), 109.45 (C-***Ar***), 108.52 (C-***Ar***), 107.37

(C_q), 101.45 (C_q), 46.98 (C_q), 42.36(C-***11***, C-***17***, C-***10*** or C-***18***), 40.60 (C-***10*** or C-***18***), 26.31 (C-***11*** or C-***17***), 23.87 (C-***16***), 8.42 (C-***22***), 8.31 (C-***14***). **HRMS** ASAP (Solid): Calc. for $C_{26}H_{27}N_2$ $[M+H]^+$: 367.2096: Found: 367.2174.

Dimer **7g**

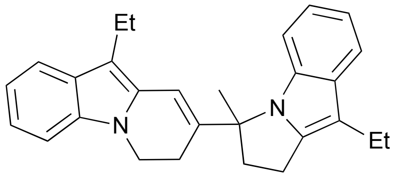

^{1}**H NMR** (500 MHz, $CDCl_3$) δ = 7.59 (d, *J*=7.9, 1H), 7.56 (d, *J*=7.9, 1H), 7.24 – 7.13 (m, 4H), 7.09 (t, *J*=7.5, 1H), 7.05 (t, *J*=7.4, 1H), 6.64 (s, 1H), 4.24 – 4.02 (m, 3H), 4.01 – 3.82 (m, 1H), 2.78 (q, *J*=7.3, 2H), 2.69 (q, *J*=7.3, 2H), 2.77 – 2.54 (m, 2H), 2.54 – 2.31 (m, 2H), 1.70 (s, 3H), 1.25 (t, *J*=7.3, 3H), 1.23 (t, *J*=7.3, 3H). ^{13}**C NMR** (126 MHz, $CDCl_3$) δ = 144.40, 139.19, 136.49, 132.51, 132.42, 131.49, 128.61, 121.86, 120.64, 119.30, 119.06, 119.01, 118.69, 114.31, 112.75, 109.59, 108.66, 108.58, 47.18, 42.53, 42.30, 40.51, 26.17, 24.44, 22.85, 17.28, 16.17, 15.81. **HRMS** ASAP (Solid): Calc. for $C_{28}H_{31}N_2$ $[M+H]^+$: 395.2487. Found: 395.479.

5. 6-Endo Cyclisation Products as Intermediates in the formation of 2,3'-BIMs and deuterium labelling studies

5.1. Synthesis of 4a with gold catalysis[2]

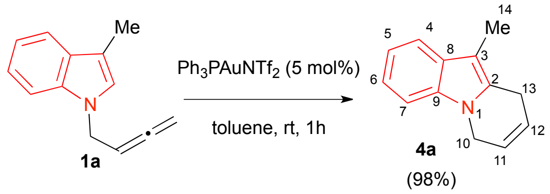

[Bis(trifluromethanesulfonyl)imidate](triphenylphosphine)gold(I) (22 mg, 0.0275 mmol) and 1-(2, 3-butadien-1-yl)-3-methyl-1H-indole (100 mg, 0.55 mmol) were added to a microwave vial, capped and flushed with N_2. The solids were dissolved in 14 mL toluene (0.04 M) and stirred at rt for 1 hour. The resulting reaction mixture was filtered through celite and washed with DCM. Compound **4a** was isolated after concentration in vacuum, 98 mg, 0.54 mmol, 98 % as a brown oil.

<u>Cycle 4a</u>: ^{1}**H NMR** (500 MHz, $CDCl_3$) δ 7.48 – 7.42 (m, 2H, H-***7***), 7.21– 7.19 (m, 1H, H-***4***), 7.12 – 7.02 (m, 2H, H-***5*** + H-***6***), 6.05 – 5.94 (m, 2H, H-***11*** + H-***12***), 4.57 – 4.52 (m, 2H, H-10), 3.48 – 3.42 (m, 2H, H-***13***), 2.19 (s, 3H, H-***14***). ^{13}**C NMR** (126 MHz, $CDCl_3$) δ 122.08 (C-***12***), 120.52 (C-***11***), 120.24 (C-***Ar***), 119.14 (C-***Ar***), 117.74 (C-***Ar***), 108.48 (C-***Ar***), 41.82 (C-***10***), 22.81 (C-***13***), 8.28 (C-***14***). Spectra consistent with previously published data.[2]

2 J. Barluenga, M. Piedratig, A. Ballesteros, A. L. S. Sobrino and J. M. Gonzalez, *Chem. Eur. J.* **2010**, *16*, 11827-18831.

5.2 Synthesis of deuterated indoles and indolyl allenes

5.2.a. Synthesis of 1-(2,3-butadien-1-yl)-3-methyl-2-deuterio-1H-indole (1a) from 3-methyl-1H-indole (11)[3]

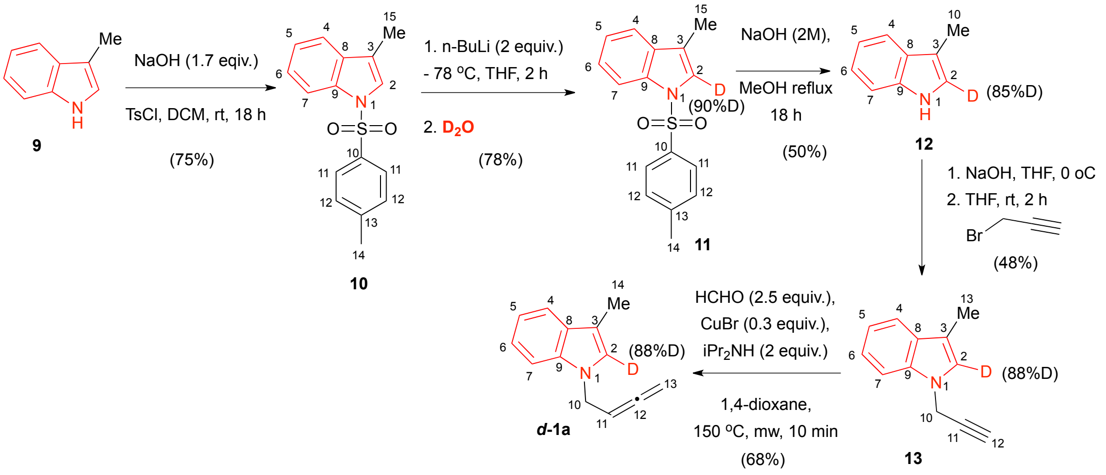

Sodium hydroxide (267 mg, 6.7 mmol) was dissolved in DCM (15 mL, 0.25 M) under N_2. 3-Methylindole **9** (500 mg, 3.82 mmol) was added and the reaction mixture was stirred for 30 minutes. A solution of p-toluenesulfonylchloride (798 mg, 4.2 mmol) was added and the reaction was stirred for 18 hours at rt. The reaction was stopped and filtered, the filtrate was concentrated in vacuum, column chromatography using Pet:EtOAc (20:1) was used to isolate 811.7 mg, 2.8 mmol, 75 % of compound **10** as a white solid. Compound **10**: ^{1}**H NMR** (500 MHz, $CDCl_3$) δ 7.98 (d, *J* = 8.2 Hz, 1H, H-***4***), 7.74 (d, *J* = 8.2 Hz, 2H, H-***12***), 7.45 (d, *J* = 7.3 Hz, 1H, H-***7***), 7.31 (m, 2H, H-***2*** + H-***6***), 7.23 (td, *J*=7.5, 1.1 Hz, 1H, H-***5***), 7.19 (dd, *J* = 8.2, 1.0 Hz, 2H, H-***11***), 2.33 (s, 3H, H-***13***), 2.24 (d, *J* = 1.2 Hz, 3H, H-***14***). ^{13}**C NMR** (126 MHz, $CDCl_3$) δ 144.76 (C-***13***), 135.57 (C-***10***), 135.39 (C-***9***), 131.91 (C-***8***), 129.8 (CH, C-***11***), 126.7 (CH, C-***12***), 124.5 (CH, C-***2***), 123.0 (CH, C-***5***), 122.9 (CH, C-***6***), 119.3 (CH, C-***4***), 118.71 (C-***3***), 113.8 (CH, C-***7***), 21.7 (CH_3, C-***14***), 9.8 (CH_3, C-***15***).

3-Methyl-1-tosyl-1H-indole **10** (811.7 mg, 2.8 mmol) was dissolved in 11.4 mL of dry THF under N_2 and cooled to -78°C. n-Butyl lithium (2.25 ml, 5.64 mmol) was added drop wise to the solution and the reaction mixture was stirred at rt for 2 hours. The reaction was re-cooled to -78°C, quenched with D_2O and warmed tort. Et_2O followed by anhydrous K_2CO_3 were added. The solid precipitate was filtered off and the filtrate evaporated under pressure to give 634.9 mg, 2.2 mmol, 78 % of compound **11** as a yellow solid with ~90 % D incorporation. Compound **11**: ^{1}**H NMR** (500 MHz, $CDCl_3$) δ 7.98 (d, *J* = 8.2 Hz, 1H, H-***4***), 7.74 (d, *J* = 8.2 Hz, 2H, H-***12***), 7.45 (d, *J* = 7.3 Hz, 1H, H-***7***), 7.31 (ddd, *J* =8 .4, 7.2, 1.3 Hz, 1H, H-***6***), 7.23 (td, *J*=7.5, 1.1 Hz, 1H, H-***5***), 7.21 – 7.17 (m, 2H, H-***11***), 2.33 (s, 3H, H-***13***), 2.24 (d, *J* = 1.2 Hz, 3H, H-***14***).

3 A. R. Katritzky and K. Akutagawa, *Tetrahedron Lett.* **1985**, *26*, 5935-5938.

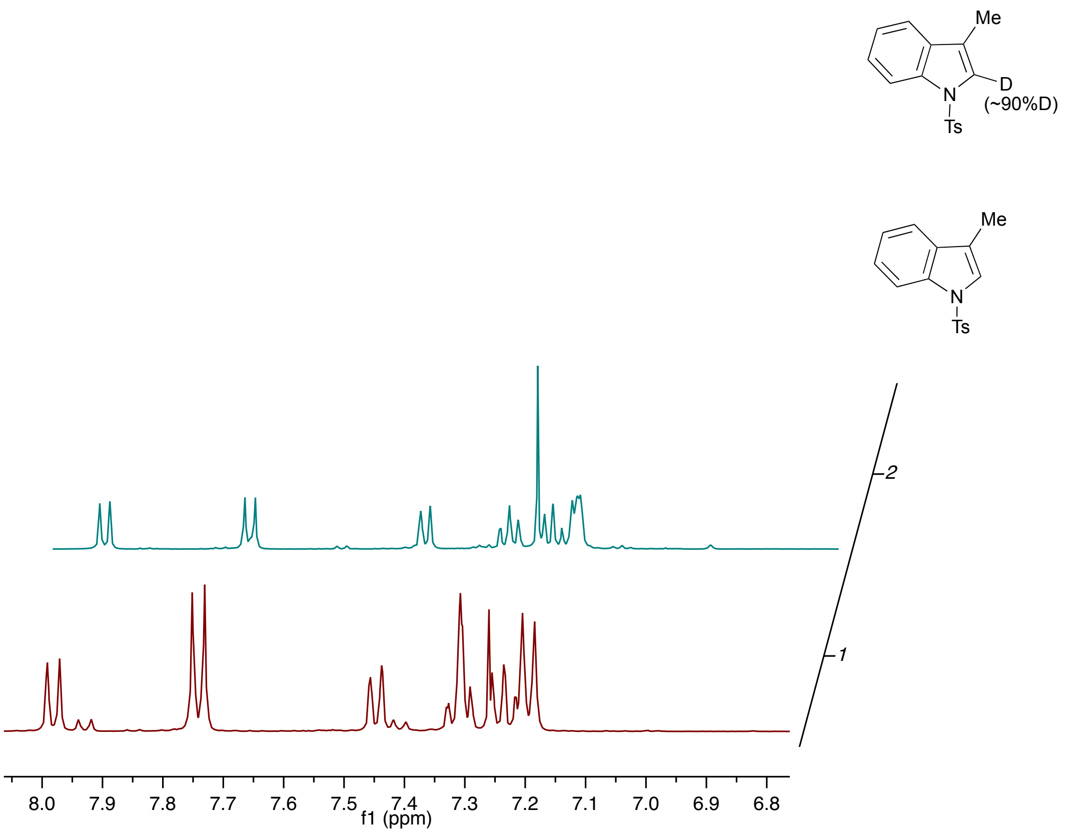

1-Phenylsulphonyl-[2-^{2}H]-3-methylindole **11** (634.9 mg, 2.2 mmol) was added to a round bottomed flask. Methanol (8.5 mL) was added followed by 2.3 mL of a 2M NaOH solution and the mixture was heated at reflux under nitrogen overnight. The reaction did not go to completion, but deprotection was observed by NMR to be around 50 %. The reaction mixture was cooled down, poured into water (10 mL) and extracted with Et_2O. The organic layer was dried with $MgSO_4$, filtered and concentrated in vacuum to give **12** 96.9 mg, 0.7 mmol, 45 % as a pale yellow solid with 85 % deuterium incorporation. This compound was used in the next step without further purification. Compound **12**: **^{1}H NMR** (500 MHz, $CDCl_3$) δ 7.90 (s, 1H, H-***1***), 7.62 (dd, *J* = 7.9, 0.6 Hz, 1H, H-***4***), 7.39 (d, *J* = 8.1 Hz, 1H, H-***7***), 7.23 (ddd, *J* = 8.1, 7.1, 1.2 Hz, 1H, H-***6***), 7.16 (td, *J* = 7.6, 1.0 Hz, 1H, H-***5***), 7.01 (d, *J* = 1.0 Hz, 0.15H, H-***2,*** H^2), 2.38 (s, 3H, H-***10***). **^{13}C NMR** (126 MHz, $CDCl_3$) δ 121.88 (CH, C-***6***), 119.12 (CH, C-***5***), 118.84 (CH, C-***4***), 110.93 (CH, C-***7***), 9.66 (CH_3, C-***10***). Some quaternary carbons not detected.

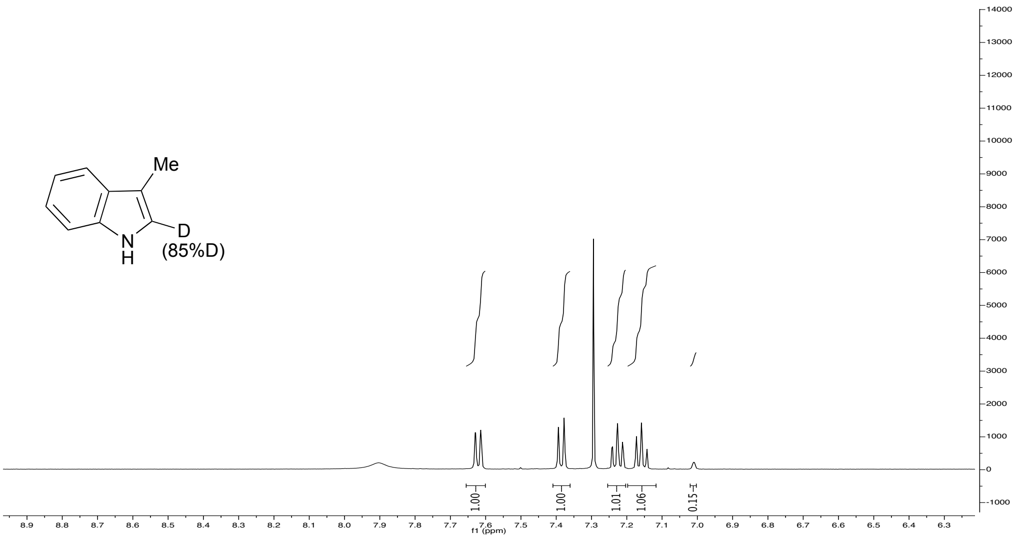

Sodium hydride (41 mg, 1.03 mmol) added to round bottomed flask under N_2 and dissolved in 11 mL THF. The reaction was cooled to 0°C and a solution of 2-deuterio-3-methyl-1H-indole **12** (271.4 mg 0.65 mmol) in THF was added and stirred for 30 minutes at 0°C. Propargyl bromide (145 mg, 0.98 mmol) was slowly added and the reaction warmed to room temperature, after 2 hours the reaction was quenched with $NaHCO_3$ and worked up with Et_2O and water. Obtained after column chromatography, Hex:EtOAc, 20:1 to give compound **13**, 53 mg, 0.3 mmol, 48 % as a yellow oil with 88 % deuterium incorporation at position 2. Compound **13**: **^{1}H NMR** (500 MHz, $CDCl_3$) δ 7.60-7.57 (m, 1H, H-***4***), 7.36 (d, *J* = 8.2 Hz, 1H, H-***7***), 7.25 (ddd, *J* = 8.2, 7.0, 1.2 Hz, 1H, H-***6***), 7.13 (ddd, *J* = 7.9, 7.1, 1.0 Hz, 1H, H-***5***), 6.98 (d, *J* = 1.0 Hz, 0.12 H, H-***2***, H^2), 4.83 (d, *J* = 2.5 Hz, 2H, H-***10***), 2.37 (t, *J* = 2.5 Hz, 1H, H-***12***), 2.34 (s, 3H, H-***13***). **^{13}C NMR** (126 MHz, $CDCl_3$) δ 121.82 (CH, C-***5***), 119.19 (CH, C-***6*** and C-***7***), 109.12 (CH, C-***4***), 73.10 (CH, C-***12***), 35.46 (CH_2, C-***10***), 9.55 (CH_3, C-***13***). Some quaternary carbons not detected.

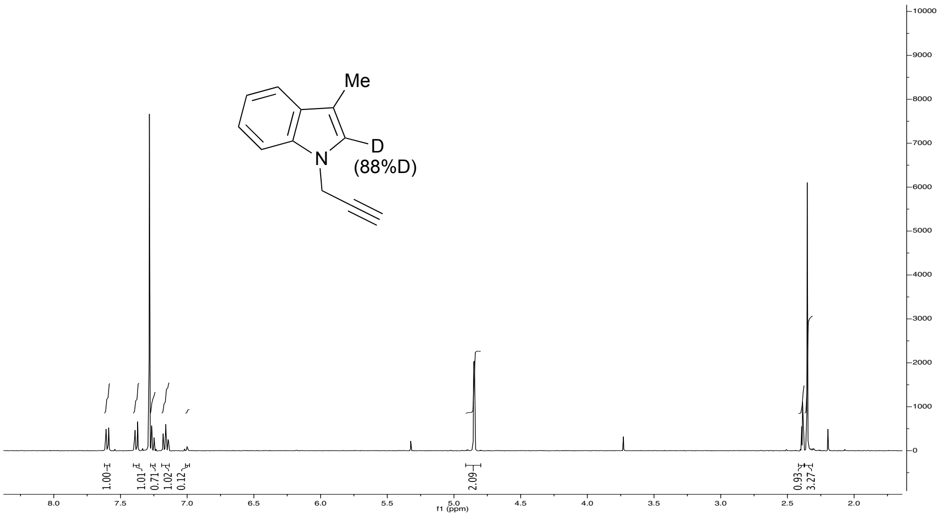

Paraformaldehyde (33 mg, 1.09 mmol), CuBr (19 mg, 0.132 mmol) and 3-methyl-1-(2-propyn-1-yl)-1H-indole (74 mg, 0.44 mmol) were added to a microwave vial, sealed, flushed with N_2 and dissolved in 2.2 mL dioxane. *i*Pr_2NH (123 µl, 0.88 mmol) was added drop wise and the reaction was heated at 150°C using microwave irradiation for 10 minutes. The reaction was filtered through celite, washed with DCM and concentrated in vacuum. Obtained after column chromatography using Pet Ether/EtOAc, 70:1, to give compound ***d*-1a**, 55.1 mg, 0.3 mmol, 68 % as a yellow oil with 88 % deuterium incorporation. Compound ***d*-1a**: **^{1}H NMR** (500 MHz,

$CDCl_3$) δ 7.57 (d, *J* = 7.9 Hz, 1H, H-***4***), 7.32 (d, *J* = 8.2 Hz, 1H, H-***7***), 7.21 (t, *J* = 7.6 Hz, 1H, H-***6***), 7.11 (t, *J* = 7.4 Hz, 1H, H-***5***), 6.90 (s, 0.12 H, H-***2,*** ^{2}H), 5.33 – 5.27 (m, 1H, H-***11***), 4.84 (dt, *J* = 6.6, 2.5 Hz, 2H, H-***13***), 4.68 (dt, *J* = 6.6, 2.6 Hz, 2H, H-***10***), 2.33 (d, *J* = 2.2 Hz, 3H, H-***14***). **^{13}C NMR** (126 MHz, $CDCl_3$) δ 121.47 (CH, C-***6***), 119.04 (CH, C-***5***), 118.74 (CH, C-***4***), 109.43(CH, C-***7***), 87.59 (CH, CH=C=C, C-***11***), 67.11 (CH_2, CH_2=C=C, C-***13***), 45.14 (N-CH_2, C-***10***), 9.57 (CH_3, C-***14***). Some quaternary carbons not detected.

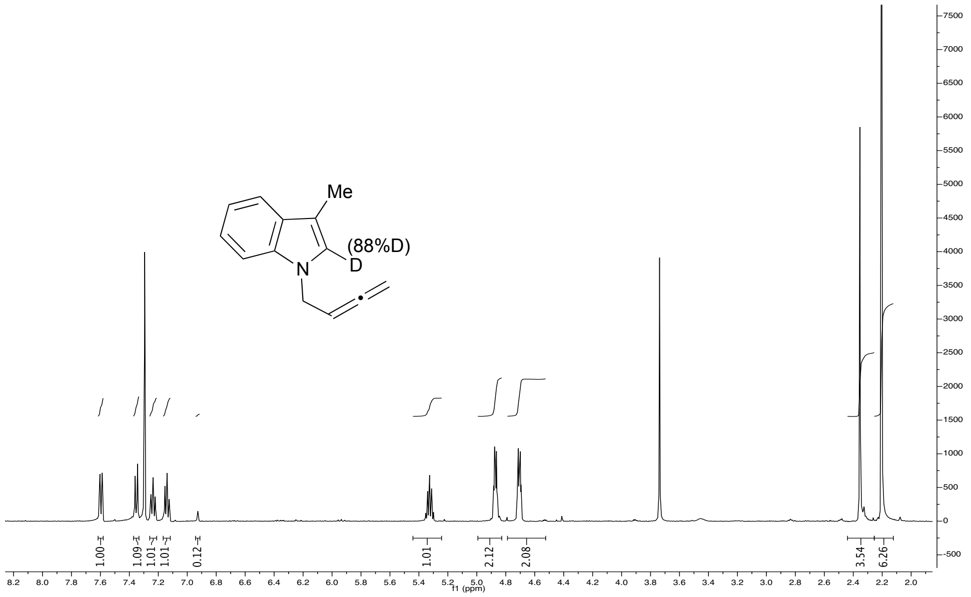

5.2.b. Synthesis of deuterated indolyl allene *d*$_2$-1a:

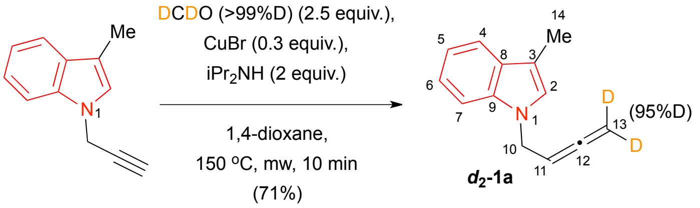

Deuterated paraformaldehyde (98 % D, 280 mg, 8.75 mmol), CuBr (150 mg, 1.05 mmol) and 3-methyl-1-(2-propyn-1-yl)-1H-indole (592 mg, 3.5 mmol) were added to a microwave vial, sealed and flushed with N_2. *i*Pr_2NH (2 eqs) was added drop wise and the reaction was heated at 150°C using microwave irradiation for 10 minutes. The reaction was filtered through celite, washed with DCM and concentrated in vacuum. Compound **59** was obtained after column chromatography, Pet Ether/EtOAc, 20:1, 462.4 mg, 2.5 mmol, 71 % as a yellow oil with >95 % D incorporation. **^{1}H NMR** (500 MHz, $CDCl_3$) δ 7.57 (d, *J* = 7.9 Hz, 1H, H-***4***), 7.32 (d, *J* = 8.2 Hz, 1H, H-***7***), 7.21 (t, *J* = 7.6 Hz, 1H, H-***6***), 7.11 (t, *J* = 7.4 Hz, 1H, H-***5***), 6.90 (s, 1H, H-***2***), 5.29 (t, *J* = 6.8 Hz, 1H, H-***11***), 4.68 (d, *J* = 6.8 Hz, 2H, H-***10***), 2.24 (s, 3H, H-***14***).

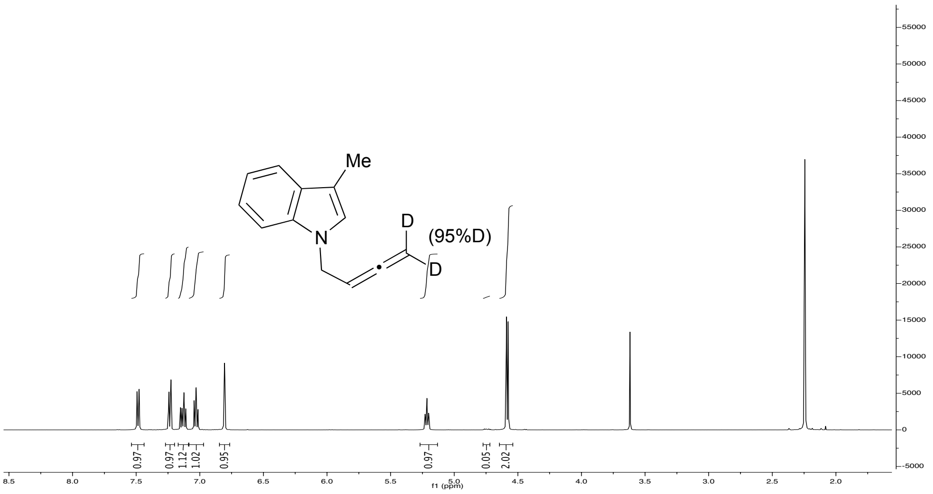

5.2.c. Synthesis of 1-methyl-(3-^{2}H)-1H-indole, *d*-2i:

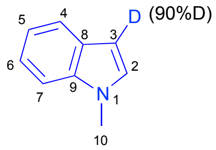

All glassware for this experiment was prewashed with deuterium oxide and dried to minimize proton exchange during the reaction.

To a pre-washed round bottomed flask was added 1-methylindole (1220 mg, 9.3 mmol), 2.5 mL D_2O was added, the reaction mixture was heated to 80°C and left for 18 hours. The reaction mixture was worked up with DCM and D_2O. Product obtained after concentrating in vacuum, to yield 1093 mg, 8.27 mmol, 89 % of compound ***d*-2i** with 90 % D incorporation. **^{1}H NMR** (500 MHz, $CDCl_3$) δ 7.71 – 7.67 (m, 1H, H-***4***), 7.38 (d, *J* = 8.2 Hz, 1H, H-***7***), 7.30 – 7.26 (m, 1H, H-***5***), 7.17 (m, 1H, H-***6***), 7.10 (s, 1H, H-***2***), 6.55 (d, *J* = 3.1 Hz, 0.1H, H^{2}-***3***), 3.85 (s, 3H, H-***10***). **^{13}C NMR** (126 MHz, $CDCl_3$) δ 136.7 (C-***9***), 128.8 (C-***8***), 128.5 (CH, C-***2***), 121.5 (CH, C-***5***), 120.9 (CH, C-***4***), 119.3 (CH, C-***6***), 109.2 (CH, C-***7***), 32.8 (CH_3, C-***10***).

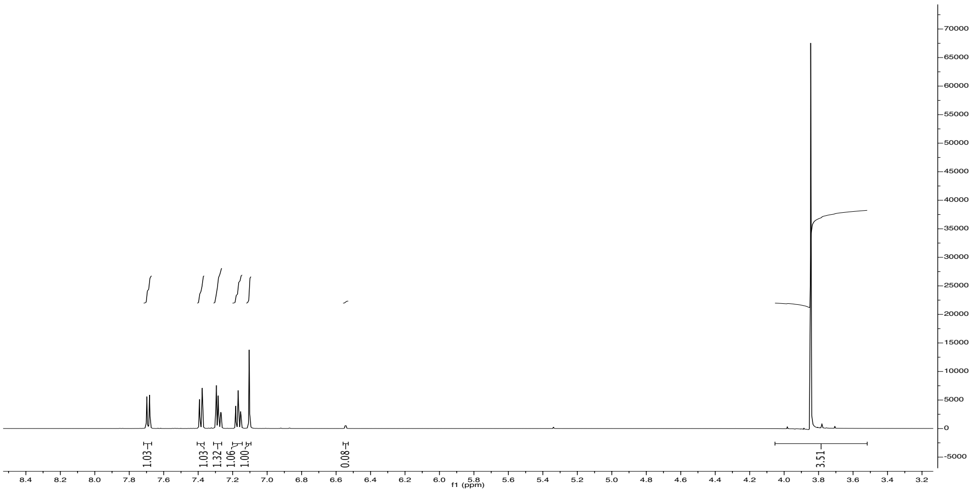

5.3. Reaction profile of 1a with *d*-2i monitored by ^{1}H NMR

Platinum-catalysed reaction was carried out under optimal conditions and microwave irradiation with the previously isolated 6-endo cycle **4a** and external *N*-methyl indole ***d*-2i** with deuterium incorporated into position 3 of the indole (Figure 5.3.1) using dimethylsulfone as internal reference. Samples of the reaction mixture were taken every 10 minutes over a 90-minute period and the progress of the reaction was analysed by ^{1}H NMR of the crude using the signals corresponding to the protons in position *a* of the three compounds: 4 ppm (CH_2, **4a**), 4.5 ppm (CH_2, **4a'**) and 4.18 ppm (CH diastereotopic, **3ai**) to measure the integrals/concentrations of products **4a**, **4a'** and **3ai** over time using the signal of the dimethylsulfone as reference.

Figure 5.3.1. Platinum-catalysed reaction of cycle **4a** with deuterated *N*-methyl indole ***d*-2i** and ^{1}H-NMR profile of the reaction highlighting consumption of **4a** and formation of **4a'**and **3ai** over 90 minutes.

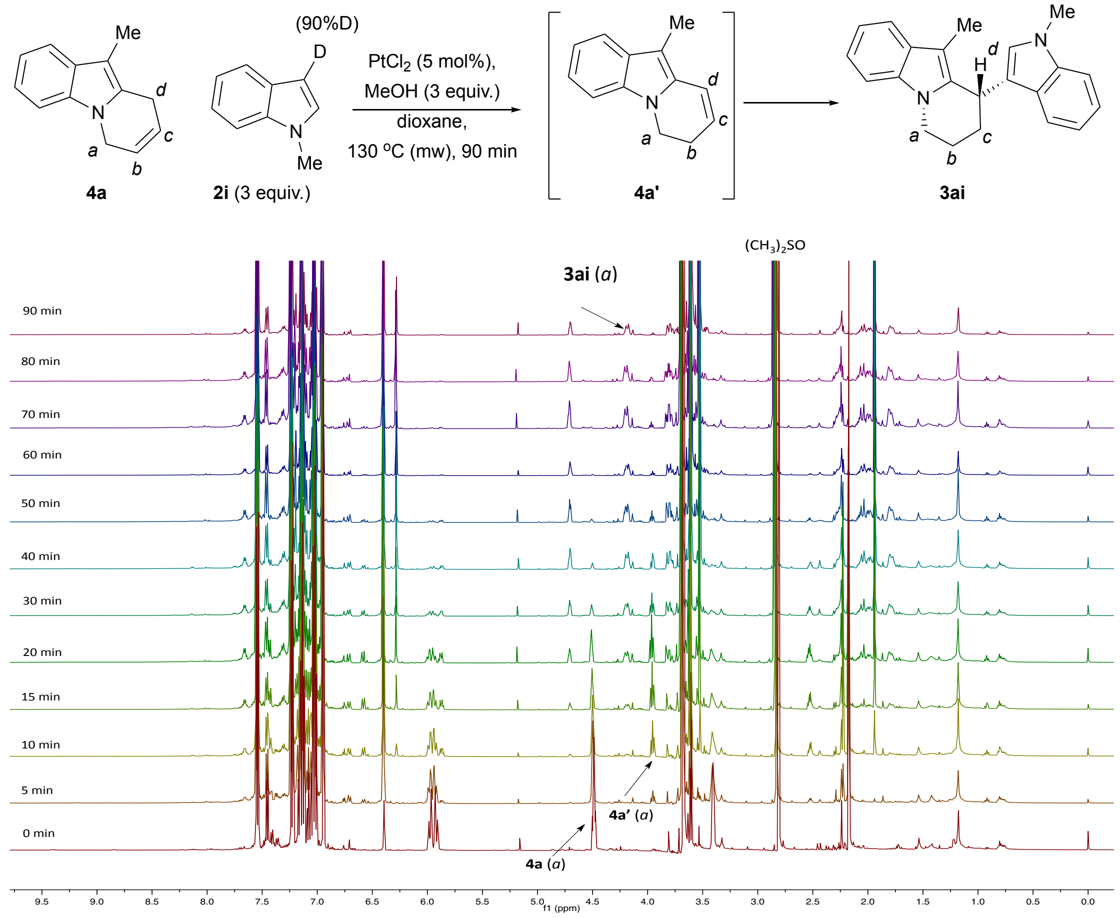

The plot the concentration of the products over time (Figure 5.3.2) shows that the non-conjugated cycle **4a** isomerizes to the conjugated cycle **4a'** within the first 20 minutes. Also noted is that as soon as isomerisation to cycle **4a'** begins, so does the formation of compound **3ai**, although at a slower rate. At 20 minutes, concentration of cycle **4a'** starts to decrease as the concentration of **3ai** continues to increase and once both of the cycles are consumed the reaction is complete.

Figure 5.3.2. Reaction profile showing the consumption of cycle **4a**, formation and consumption of cycle **4a'** with subsequent formation of 2,3'-BIM **3ai**.

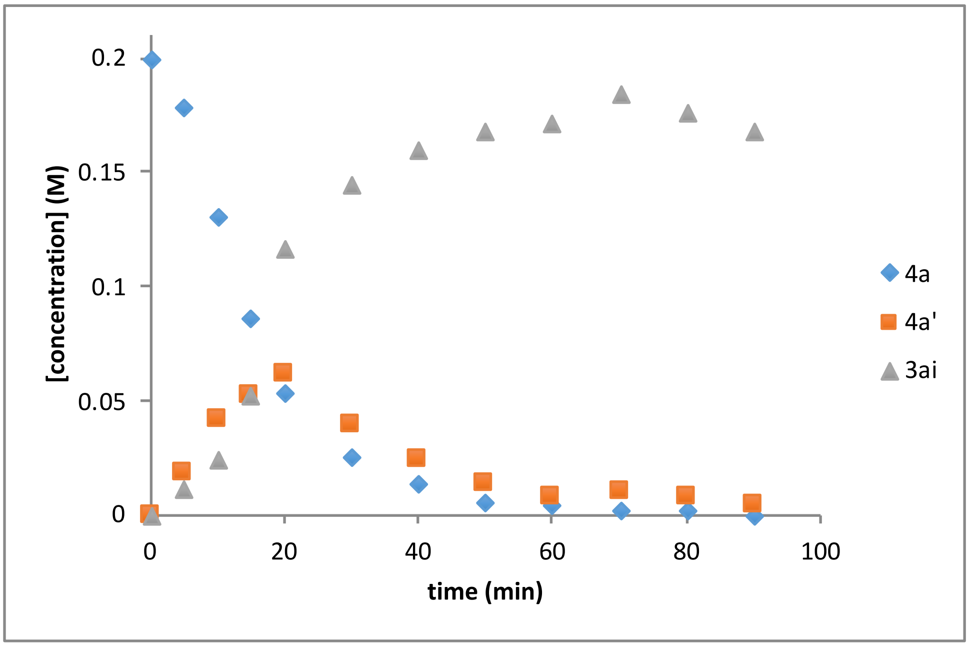

The reaction was carried out with deuterated *N*-methyl indole ***d*-2i**, with 90 % deuterium incorporation at the beginning of the reaction. After 5 minutes we observed a loss of deuterium in position 3. Overall we observe a deuterium loss in the external indole of 41 % from position 3 and analysis of the NMRs shows that deuterium is incorporated into cycle **4a'** at position *d* within the first 10 minutes. Analysis also shows that deuterium is incorporated into position *d* of compound **3ai** after 10 minutes with around 31 % deuterium incorporated in the final compound.

5.4. Experiments with deuterated intermediates

5.4.a. 3-Methyl-*N*-(2,3-butadienyl)indole ***d*₂-1a** was reacted using gold catalysis to form the non-conjugated cycle ***d*₂-4a** in an 82 % yield, with 90 % deuterium incorporation at position d (**Figure 5.4.1**).

Figure 5.4.a. Au-catalysed cyclisation of deuterated 3-methyl-*N*-(2,3-butadienyl) indole ***d*₂-1a** with retention of deuterium.

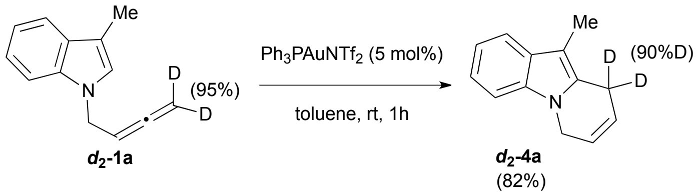

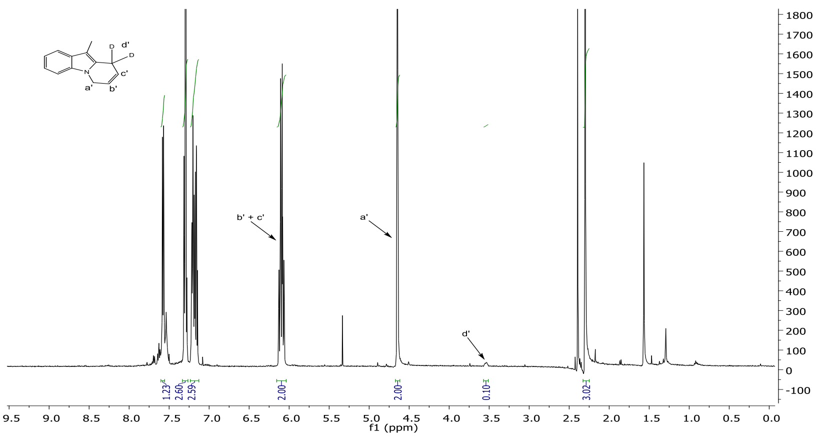

5.4.b. Cycle ***d*₂-4a** was reacted under platinum conditions without the external indole. Successful isomerisation occurred with >99 % conversion to cycle ***d*₂-4a’** with an 82 % yield. Analysis of the ^{1}H NMR identified that deuterium was incorporated into positions *c* and *d* in 26 and 40 % respectively (**Figure 5.4.b**). Some deuterium loss is observed in the process (~ 20%).

Figure 5.4.b. Pt catalysed isomerisation of cycle ***d*₂-4a** to cycle ***d*₂-4a’**.

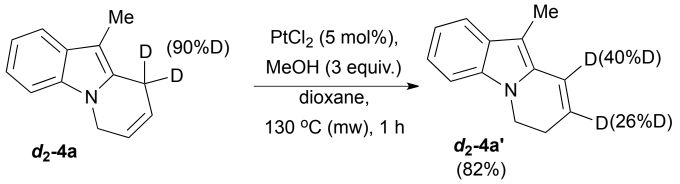

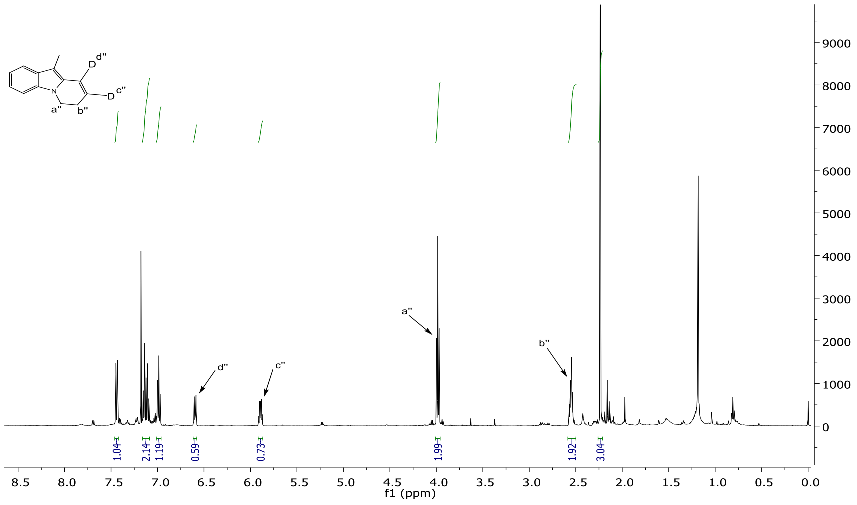

5.4.c. When cycle ***d*₂-4a** was subject to optimized conditions in the presence of external indole **2i**, only traces of the desired 2,3’-BIM were identified in the crude ^{1}H NMR and only cycles ***d*₂-4a** and ***d*₂-4a’** were isolated from the reaction mixture in a 75 % yield in a mixture with a ratio of 1:0.7 (***d*₂-4a: *d*₂-4a’**) and that cycle ***d*₂-4a** had 29 % deuterium incorporation at position *d* and cycle ***d*₂-4a’** had deuterium in the expected *c* and *d* positions with 6 and 30 % respectively. Also noted from the crude NMR is the incorporation of 12 % deuterium into position 3 of the external indole **2i** (**Scheme 5.4.c**).

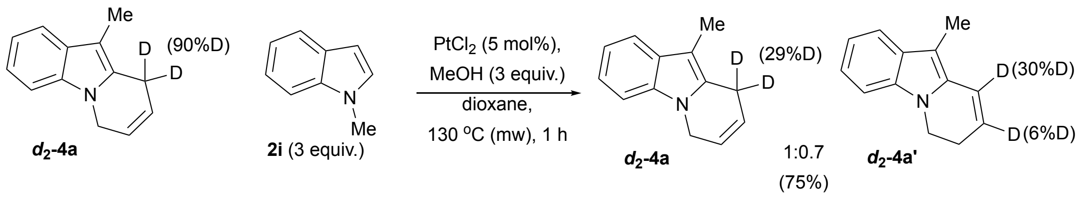

Scheme 5.4.c. Pt-catalysed reaction of cycle ***d*₂-4a** with external indole **2i**.

5.4.d. When cycle ***d*₂-4a’** obtained from the reaction in **Figure 5.4.b** was subject to the platinum conditions with the external indole **2i**, cycle ***d*₂-4a’** and product **3ai** were isolated in 44 and 38 % yields respectively. Analysis of cycle ***d*₂-4a’** shows deuterium retention in position *d* and partial loos in *c*, whereas analysis of compound **3ai** by both ^{1}H NMR and HSQC NMR indicated that no deuterium was incorporated into the final compound (**Scheme 5.4.d**).

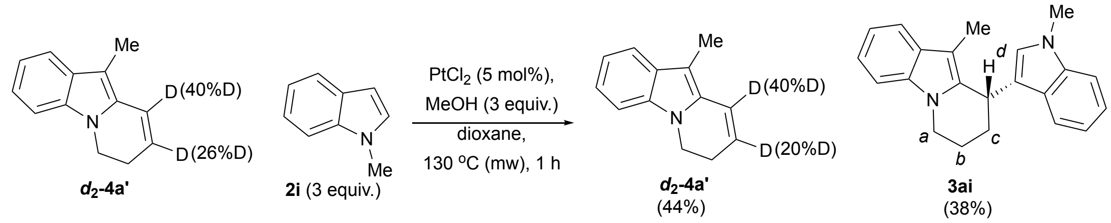

Scheme 5.4.d. Pt-catalysed reaction of cycle ***d*₂-4a’** with external indole **2h**.

5.4.e. 3-Methyl-2-deuterio-1-(2,3-butadien-1-yl) indole ***d*-1a** was reacted under gold catalysis conditions to give the non-conjugated cycle ***d*-4a**. Analysis of the crude ^{1}H NMR showed incorporation of 50 % deuterium at position c’ (**Figure 5.4.e.**).

Scheme 5.4.e. Au-catalysed cyclisation of 3-methyl-2-deuterio-1-(2,3-butadien-1-yl) indole ***d*-1a**.

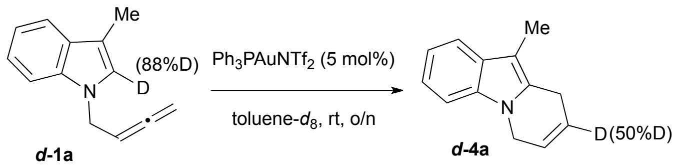

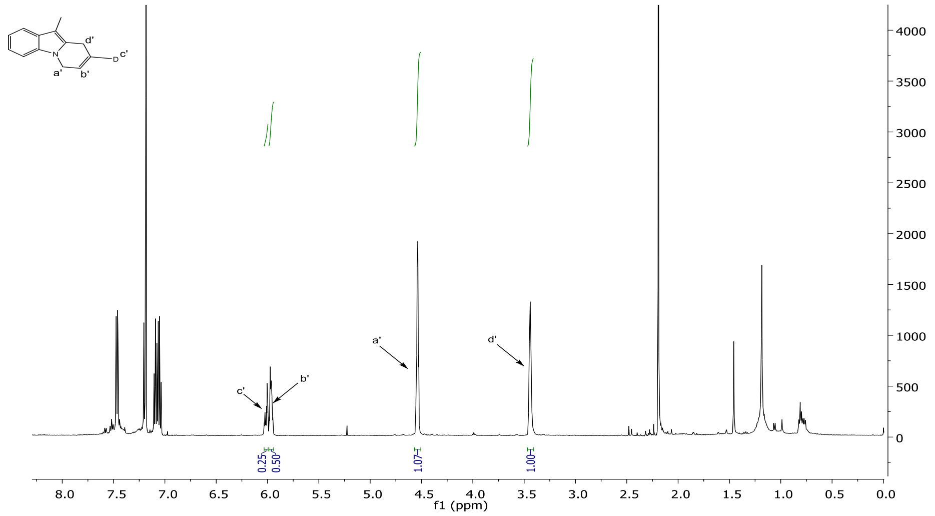

5.4.f. Isomerisation of cycle ***d*-4a** was carried out under platinum conditions with no external indole, however 100 % conversion was not achieved and cycles ***d*-4a** and ***d*-4a’**

were obtained in a ratio of 1:5 (**Scheme 5.4.f**). The crude ^{1}H NMR shows deuterium incorporation of both cycles at position c, with cycle ***d*-4a** containing 8 % and cycle ***d*-4a’** containing 16 %.

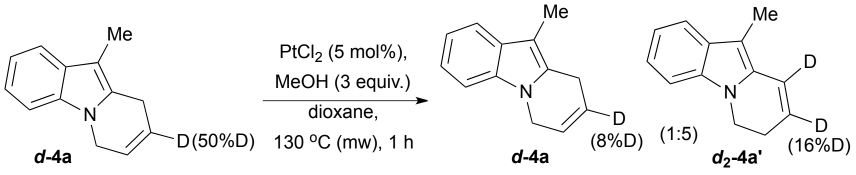

Scheme 5.4.f. Platinum-catalysed isomerisation of cycle ***d*-4a** to ***d*-4a’**.

5.4.g. The mixture two deuterated cycles obtained in the reaction shown in Scheme 5.4.f. was reacted with external indole **2i** using standard platinum conditions, this resulted in the formation of the 2,3’-BIM ***d*$_n$-3ai** and cycle ***d*-4a’** (**Scheme 5.4.g**). Analysis of the crude ^{1}H NMR showed a ratio of 1:2 (***d*-4a’**:***d*$_n$-3ai**) and deuterium incorporation was found ` 8 % deuterium was incorporated at position c’’ of ***d*-4a’** and only 8 % was observed at position d of compound ***d*$_n$-3ai**.

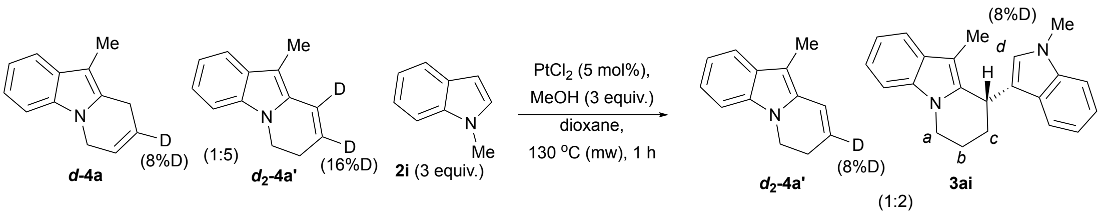

Scheme 5.4.g. Platinum catalysed reaction of cycles ***d*-4** and ***d*-4’** with indole **2i**.

6. Further Labelling Experiments

6.1. Deuteration experiments

Reactions were carried out using deuterated and undeuterated starting materials **1a**, ***d*$_2$-1a**, ***d*-1a**, **2i**, ***d*-2i** or a combination of them in the absence and presence of methanol, either CH_3OH or CD_3OD (Table 6.1). The ration of the three products and the deuterium incorporation in the different positions observed for all the compounds are indicated for each reaction. The deuterium incorporation was analysed by measuring the integrals of the different signals of all the products in the ^{1}H NMR of the crude and by analysis of the ^{1}H NMR and the HSQC of the purified compounds when possible.

Representative analysis of a pure sample of 2,3’BIM ***d*$_n$-3ai** obtained in the reaction in entry 1 in Table 6.1 is shown below: Analysis of the ^{1}H NMR of the purified ***d*$_n$-3ai** in **figure 6.1a** shows deuterium incorporation in positions b to d, this was established by comparing the spectrum of the deuterated product with the non-deuterated example, analysing the disappearance of signals or change in multiplicity on those positions and measuring the integrals in relationship to the signal of the proton at 6.25 ppm corresponding to one proton on the C2 position on the *N*-methyl indole. Position *a* is unaffected in the reaction, whereas positions *b* to *d* have significantly lower integrals which supports deuterium incorporation. Interestingly, both diastereotopic positions of methylenes *b* and *c* showed some degree of deuterium incorporation. These are also supported by analysis of the HSQC (**Figure 6.1b**) where we observe a mixture of CH_2 (blue spots) and CHD (red spots, shifted to the top right of the blue signals due to the isotope effect), the intensity of these two signals are in proportion to the calculated % D incorporation.

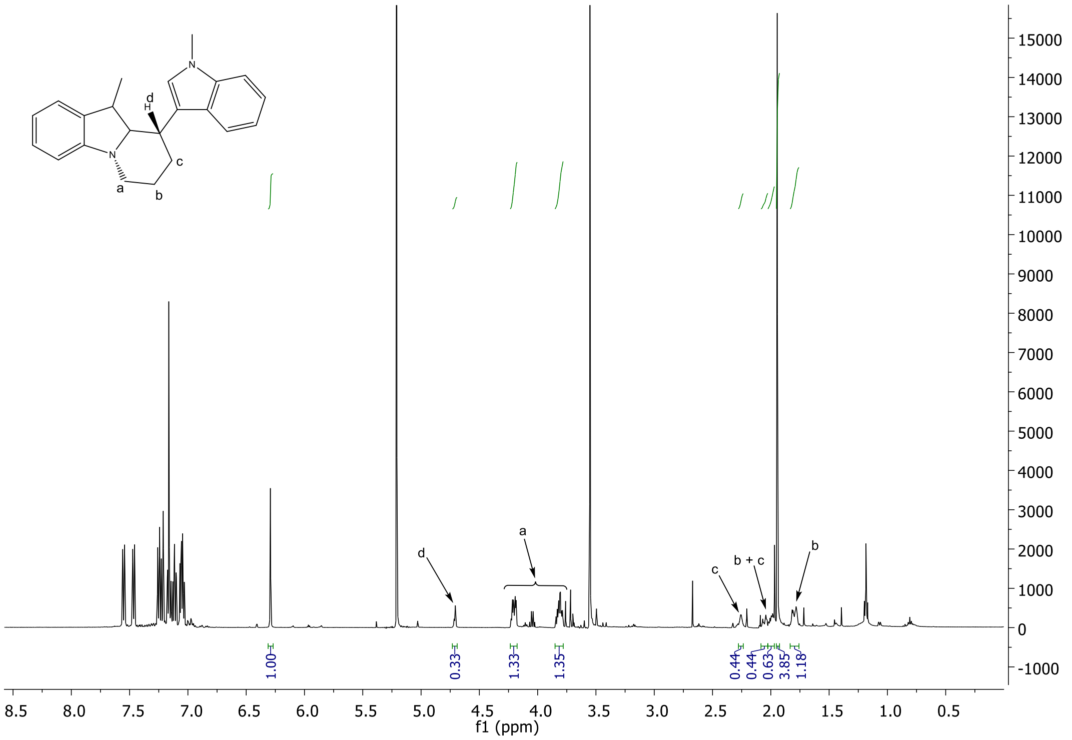

Figure 6.1a. ^{1}H NMR highlighting deuterium incorporation in compound ***d*$_n$-3ai** from reaction shown in **entry 1 Table 6.1.**

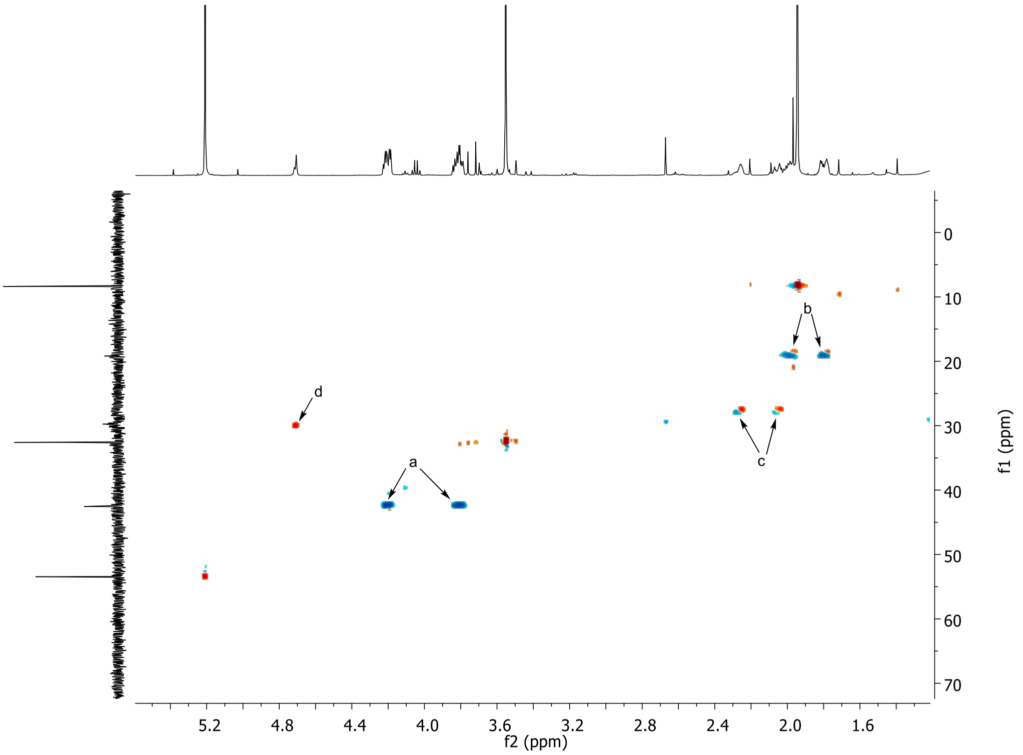

Figure 22. HSQC NMR for compound ***d*$_n$-3ai**, showing clearly the deuterium incorporation at positions b and c.

Table 6.1. Summary of deuteration experiments

Entry	*d*-Starting material	MeOH source	***d*$_n$-4a : *d*$_n$-4a'** 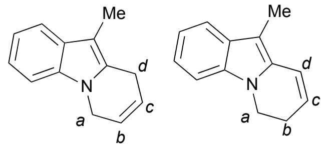		***d*$_n$-3ai** 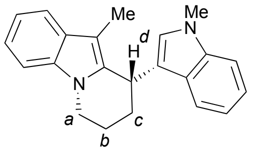	%D in recovered indole
1	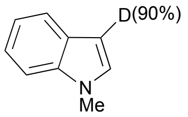	MeOH (3 eq)	Traces	0 : 0.4 : 1[a]	31% yield	62 (28% loss)
			--[d]	(CH$_2$), b: 20%D[b]<br>(CH), c: 50%D[b]<br>(CH), d: 84%D[b]	(CH$_2$), b: 37, 12%D<br>(CH$_2$), c: 55, 55%D<br>(CH), d: 67%D	
2	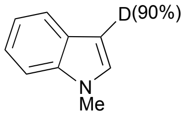	CD_3OD (3 eq)	3% yield	0.3 : 1 : 0.7[a]	19% yield	55 (35% loss)
			(CH), b: 0%D<br>(CH), c: 38%D<br>(CH$_2$), d: 46%D	(CH$_2$), b: 35%D<br>(CH), c: 28%D<br>(CH), d: 11%D	(CH$_2$), b: 0, 28%D<br>(CH$_2$), c: 14, 20%D<br>(CH), d: 58%D	
3	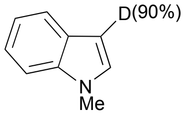	--	7% yield	0 : 2 : 1[a]	4% yield	27 (63% loss)
			--[d]	(CH$_2$), b: 36%D<br>(CH), c: 31%D<br>(CH), d: 32%D	(CH$_2$), b: 0, 9%D<br>(CH$_2$), c: 0, 23%D<br>(CH), d: 27%D	
4	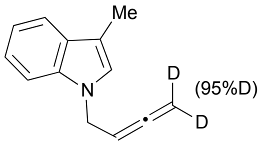	MeOH (3 eq)	12%	0 : 1 : 1[a]	31%	24 (gain)
			--[d]	(CH$_2$), b: 22%D<br>(CH), c: 34%D<br>(CH), d: 54%D	(CH$_2$), b: 0-50%D[c]<br>(CH$_2$), c: 0-50%D[c]<br>(CH), d: 20%D[a] (8%D after column)	
5	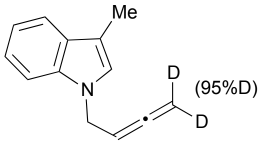	CD_3OD (3 eq)	1 : 1[a]		--[d]	42 (gain)
			(CH), b: 10%D[b]<br>(CH), c: 17%D[b]<br>(CH$_2$), d: 30%D[b]	(CH$_2$), b: 15%D[b]<br>(CH), c: 68%D[b]<br>(CH), d: 76%D[b]		

a. Ratio of products in the ^{1}H NMR of the crude. **b.** Measured in the ^{1}H NMR of the crude. **c.** Not accurate due to overlap of the signals. **d.** Not formed in the reaction.

Entry	*d*-Starting material	MeOH source	***d*$_n$-4a : *d*$_n$-4a'**		***d*$_n$-3ai**	%D in recovered indole
			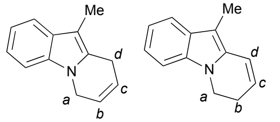		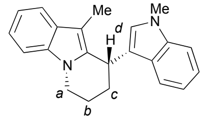	
6	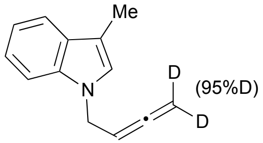	--	1.6 : 1[a]		-- [d]	20 (gain)
			(CH), b: 0%D[b]<br>(CH), c: 0%D[b]<br>(CH_2), d: 31%D[b]	(CH_2), b: 5%D[b]<br>(CH), c: 66%D[b]<br>(CH), d: 70%D[b]		
7	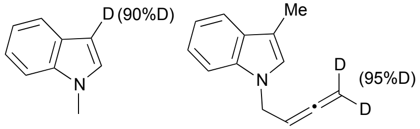	MeOH (3 eq)	1.5 : 1[a]		--[d]	42 (48% loss)
			(CH), b: 10%D[b]<br>(CH), c: 10%D[b]<br>(CH_2), d: 68%D[b]	(CH_2), b: 10%D[b]<br>(CH), c': 76%D[b]<br>(CH), d'': 82%D[b]		
8	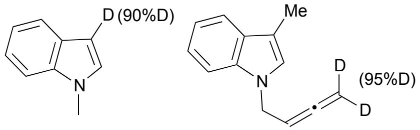	CD_3OD (3 eq)	1.6 : 1[a]		--[d]	20 (70% loss)
			(CH), b: 12%D[b]<br>(CH), c: 34%D[b]<br>(CH_2), d: 68%D[b]	(CH_2), b: 24%D[b]<br>(CH), c: 74%D[b]<br>(CH), d: 77%D[b]		59 (31% loss)
9	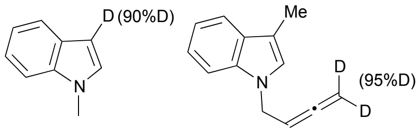	--	1.2 : 1[a]		--[d]	31 (59% loss)
			(CH), b: 50%D[b]<br>(CH), c: 20%D[b]<br>(CH_2), d: 65%D[b]	(CH_2), b: 15%D[b]<br>(CH), c: 80%D[b]<br>(CH), d: 86%D[b]		

a. Ratio of products in the ^{1}H NMR of the crude. **b.** Measured in the ^{1}H NMR of the crude. **c.** Not accurate due to overlap of the signals. **d.** Not formed in the reaction

Entry	*d*-Starting material	MeOH source	***d*n-4a:*d*n-4a'**		***d*n-3ai**	%D in recovered indole
			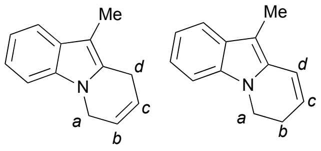		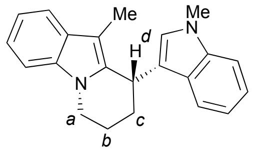	
10	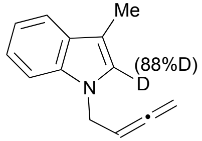	MeOH (3 eq)	1 : 2.4 : 1.4			20 (gain)
			(CH), b: 0%D[b]<br>(CH), c: 0%D[b]<br>(CH_2), d: 15%D[b]	(CH_2), b: 30%D[b]<br>(CH), c: 16%D[b]<br>(CH), d: 21%D[b]	(CH_2), b: 0-50%D[c]<br>(CH_2), c: 0-50%D[c]<br>(CH), d: 30%D	
11	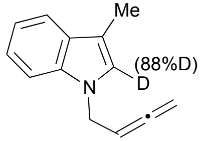	CD_3OD (3 eq)	0.2 : 1 : 0.4			32 (gain)
			(CH), b: 42%D[b]<br>(CH), c: 73%D[b]<br>(CH_2), d: 89%D[b]	(CH_2), b: 33%D<br>(CH), c: 62%D<br>(CH), d: 47%D	(CH_2), b: 0-50%D[c]<br>(CH_2), c: 0-50%D[c]<br>(CH), d: 29%D	
12	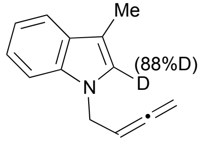	--	0.3 : 1 : 0.4			13 (gain)
			(CH), b': 20%D<br>(CH), c': 10%D<br>(CH_2), d': 0-50%D[c]	(CH_2), b'': 11%D<br>(CH), c': 33%D<br>(CH), d'': 16%D	(CH_2), b: 0-50%D[c]<br>(CH_2), c: 0-50%D[c]<br>(CH), d: 10%D	

a. Ratio of products in the ^{1}H NMR of the crude. **b.** Measured in the ^{1}H NMR of the crude. **c.** Not accurate due to overlap of the signals. **d.** Not formed in the reaction.

6.2. ¹³C-Labelling experiments

6.2.a. Synthesis of ¹³C-1a

¹³C-labelled 3-methyl-*N*-(2,3-butadienyl)indole **¹³C-1a**, with the ¹³C in the terminal carbon of the allene, was synthesized by standard Crabbé homologation using ¹³C-paraformaldehyde.

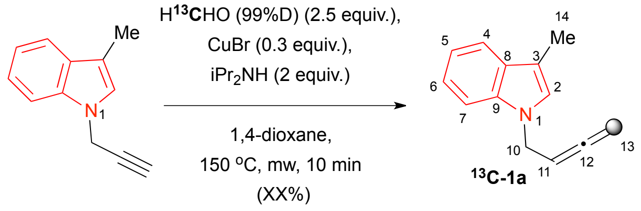

¹³C Paraformaldehyde (99 % atom) (229 mg, 7.38 mmol), CuBr (127 mg, 0.885 mmol) and 3-methyl-1-(2-propyn-1-yl)-1H-indole (500 mg, 2.95 mmol) were added to a microwave vial, sealed, flushed with N_2 and dissolved in 14.75 mL dioxane. *i*Pr_2NH (827 µl, 5.9 mmol) was added drop wise and the reaction was heated at 150°C using microwave irradiation for 10 minutes. The reaction was filtered through Celite, washed with DCM and concentrated in vacuum. **¹³C-1a** was obtained after column chromatography using Pet Ether/EtOAc, 30:1, 428.7 mg, 2.32 mmol, 79 % as a yellow oil.

¹H NMR (500 MHz, $CDCl_3$) δ 7.59 (dt, *J* = 7.9, 1.0 Hz, 1H, H-***4***), 7.34 (dt, *J* = 8.2, 0.9, Hz, 1H, H-***7***), 7.23 (ddd, *J* = 8.2, 7.0, 1.2Hz, 1H, H-***5***), 7.13 (ddd, *J* = 7.9, 7.0, 1.0 Hz, H-***6***), 6.91 (d, *J* = 1.0 Hz, 1H, H-***2***), 5.39 – 5.18 (m, 1H, H-***11***), 4.87 (ddt, $J_{C\text{-}H}$ = 168.73 Hz, $J_{H\text{-}H}$ = 6.6, 2.7 Hz, 2H, H-***13***), 4.70 – 4.68 (m, 2H, H-***10***), 2.35 (d, *J* = 1.0 Hz, 3H, H-***14***). **¹³C NMR** (126 MHz, $CDCl_3$) δ 76.92 ($^{13}CH_2$, C-***13***).

¹H NMR (500 MHz, $CDCl_3$)

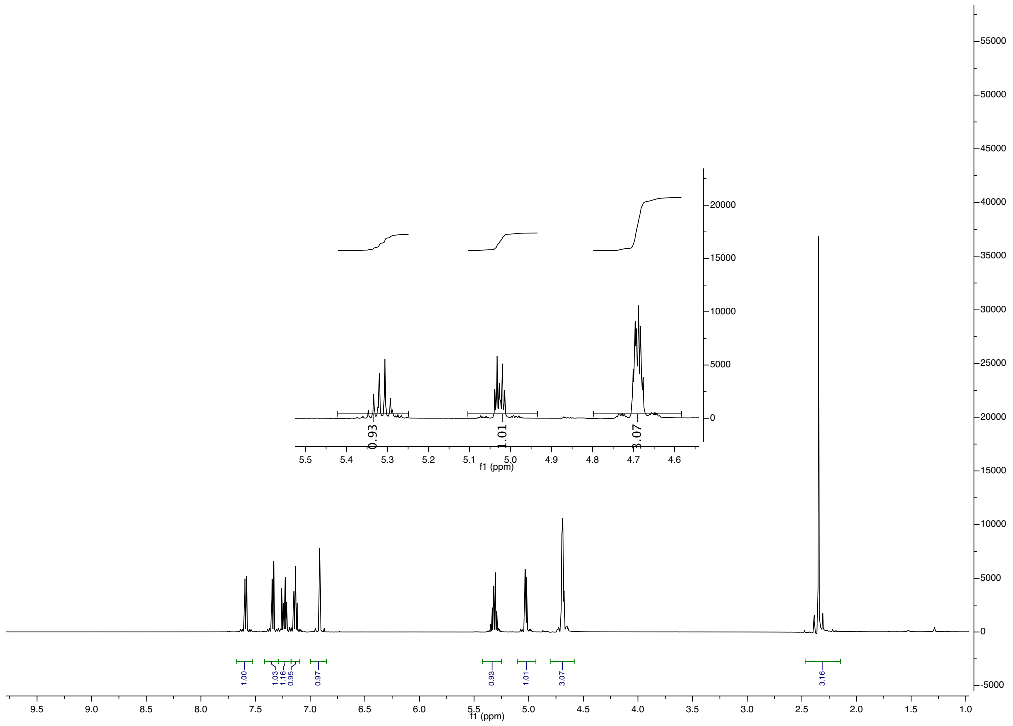

^{13}C NMR (126 MHz, $CDCl_3$)

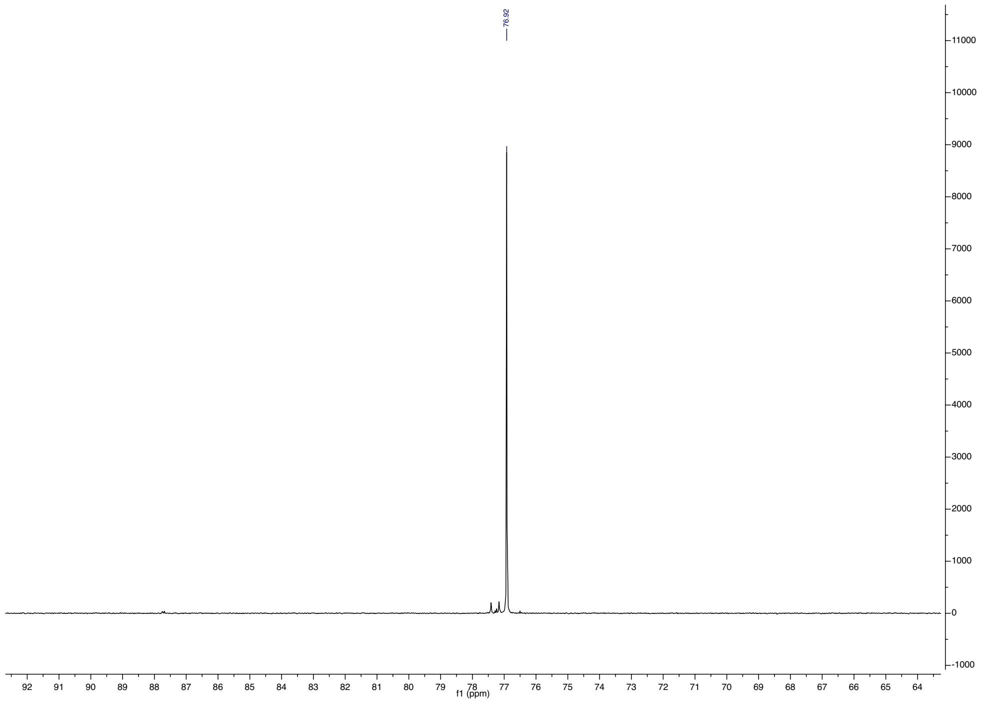

6.2.b. Reaction profile of ^{13}C-1a with indole *d*-2i

^{13}C-labelled 3-methyl-*N*-(2,3-butadienyl)indole **^{13}C-1a** was reacted with deuterated *N*-methyl indole ***d*-2i** using mw irradiation in the presence of deuterated methanol under normal optimised conditions using dimethylsulfone as the internal standard (**Scheme 6.2.b**).

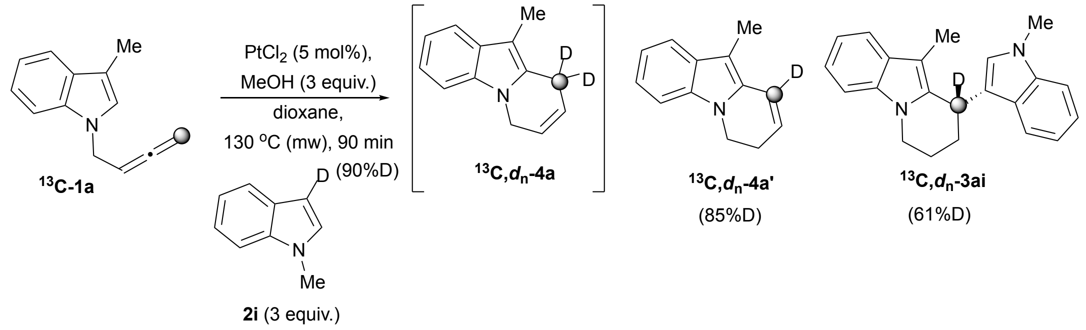

Scheme 6.2.b. Platinum-catalysed reaction of ^{13}C-labelled 3-methyl-*N*-(2,3-butadienyl)indole **^{13}C-1a** with deuterated *N*-methyl indole ***d*-2h** in the presence of deuterated methanol.

The reaction was monitored by ^{1}H NMR and ^{13}C NMR over a 90-minute period, with samples taken every 10 minutes and the progress of the reaction was analysed by ^{1}H NMR of the crude using the signals corresponding to the protons in position *a* of the three compounds: 4 ppm (CH_2, **4a**), 4.5 ppm (CH_2, **4a'**) and 4.18 ppm (CH diastereotopic, **3ai**) to measure the integrals/concentrations of products **4a**, **4a'** and **3ai** over time using the signal of the dimethylsulfone as reference. Analysis of the ^{1}H NMR spectra of the crude of the samples showed very fast reaction of the allenyl indole **1a** to form cycle **4a** and isomerization of cycle **4a** to **4a'** as seen before. However, in this case full conversion of

cycle **4a'** to the BIM **3ai** was not observed probably due to a kinetic isotope effect on the 13C-analogues (Figure 6.2.b.1, compare with graph in Figure 5.3.2).

Figure 6.2.b.1. Reaction profile showing the reaction progress for the ^{13}C labelled reaction in **scheme 6.2.b**.

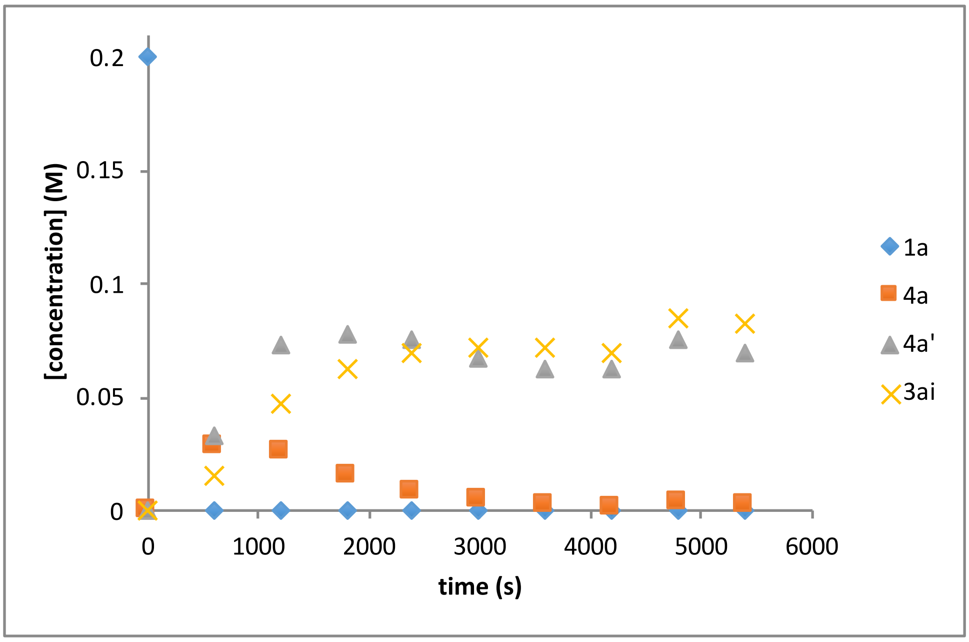

Analysis of the ^{13}C NMR spectra showed high deuterium incorporation in the labeled carbon in all compounds from early stages of the reaction (Figure 6.2.b.2).

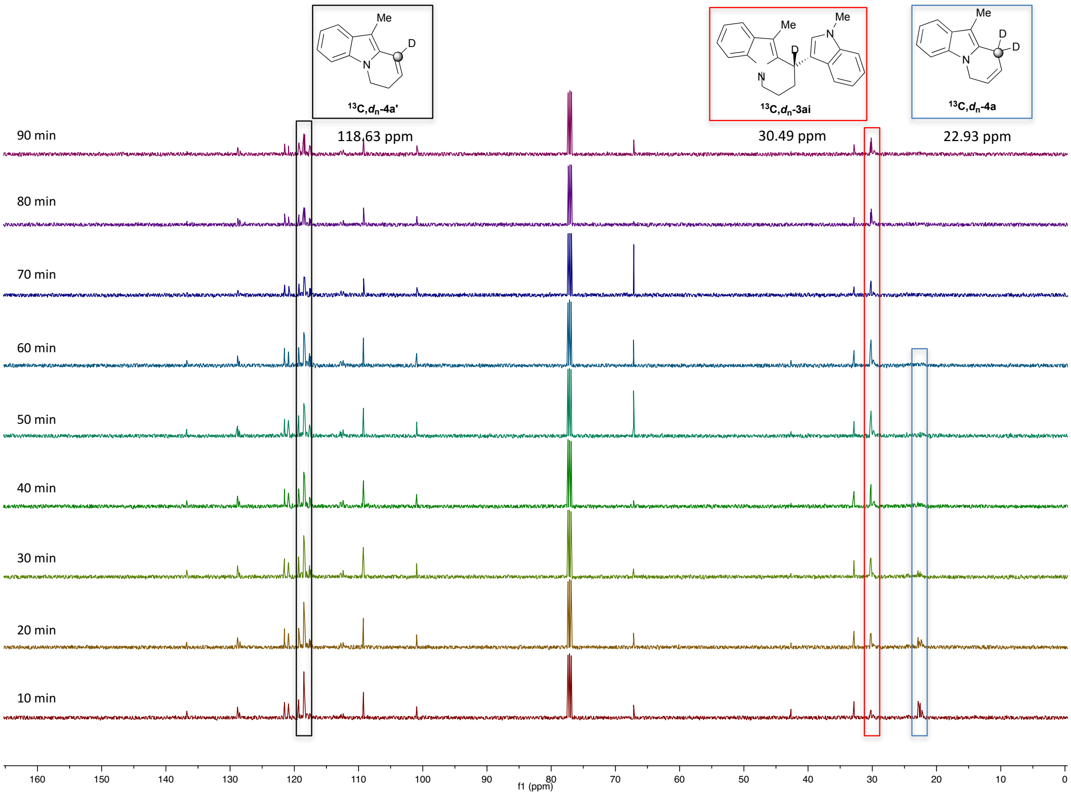

6.3. Reaction of 3ai in the presence of $PtCl_2$ and CD_3OD

The isolated non-labeled 2,3’-BIM **3ai** was reacted under platinum conditions in the presence of deuterated methanol to determine if deuterium incorporation can occur further at position *d* in an out-of-cycle process (**Scheme 6.3**). The ^{1}H NMR after 1 hour shows 42 % deuterium incorporation at position *d* only (**Figure 6.3**).

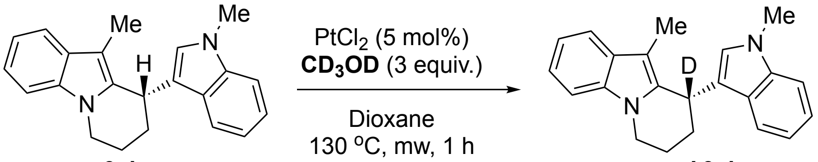

3ai *d*-**3ai**

Scheme 6.3. Platinum-catalysed reaction to highlight deuterium exchange at position d of compound **3ai** in the presence of deuterated methanol.

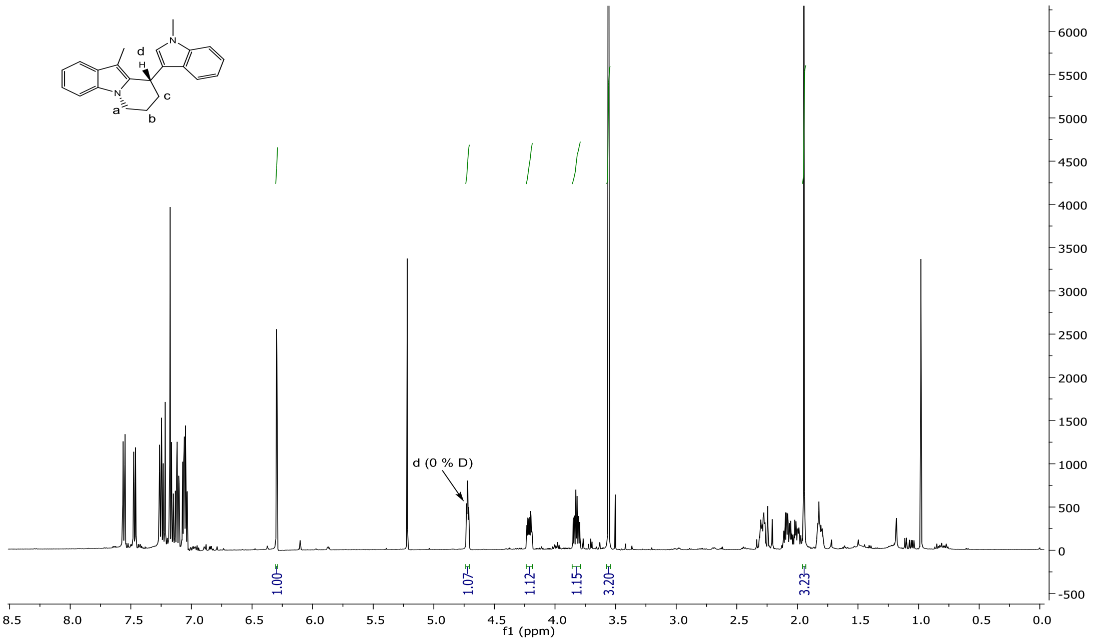

Figure 6.3a. ^{1}H NMR spectra of **3ai** before the reaction shown in **scheme 6.3**.

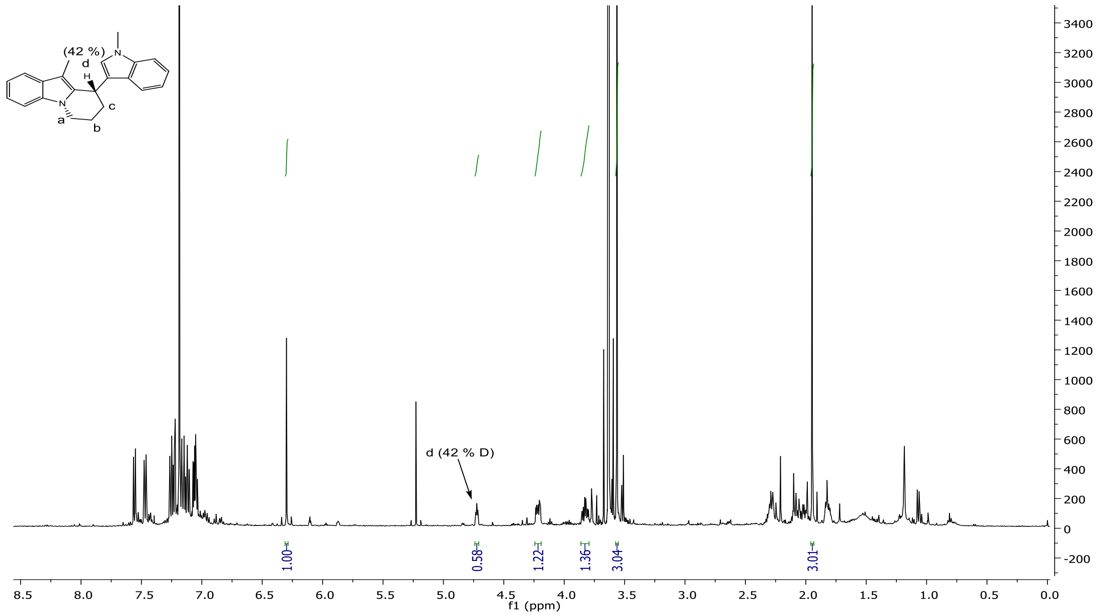

Figure 6.3b. ^{1}H NMR spectra of **3ai** after the reaction shown in **scheme 6.3**.

7. NMR spectra

Cycle 4a **Cycle 4a'**

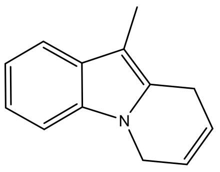

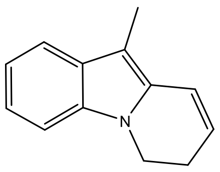

Cycle 4a

^{1}H NMR (500 MHz, $CDCl_3$, 25°C, TMS)

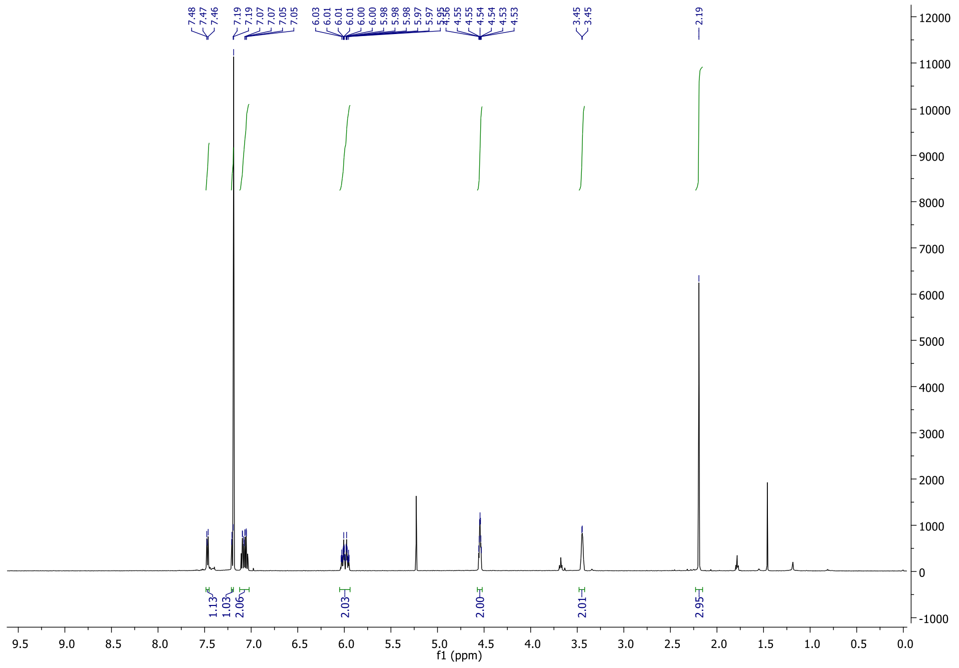

^{13}C NMR (126 MHz, $CDCl_3$, 25°C)

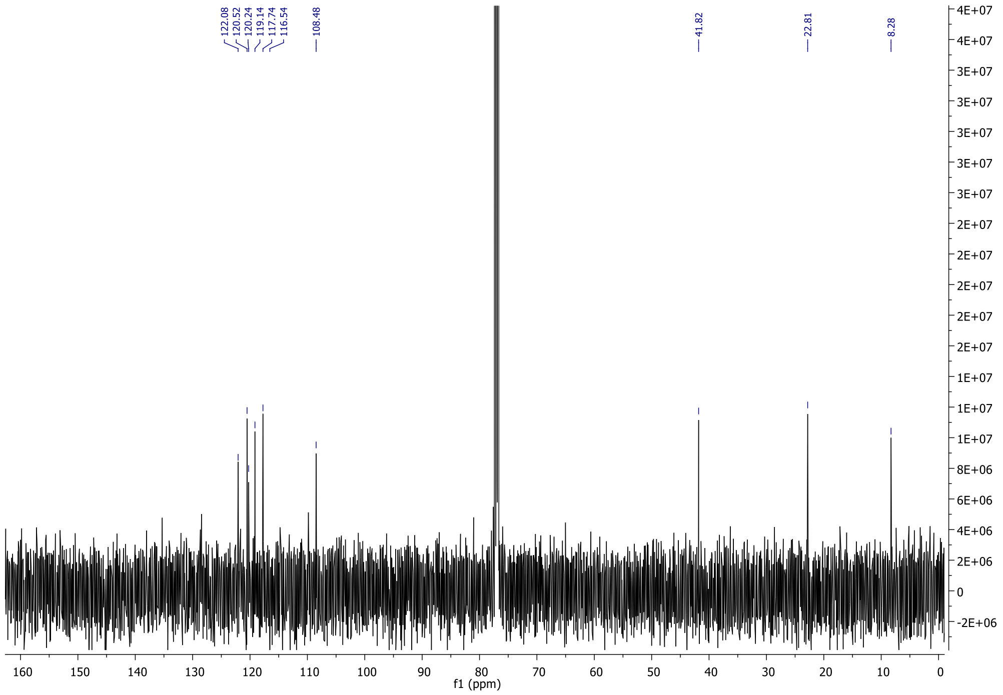

2D gCOSY ($CDCl_3$)

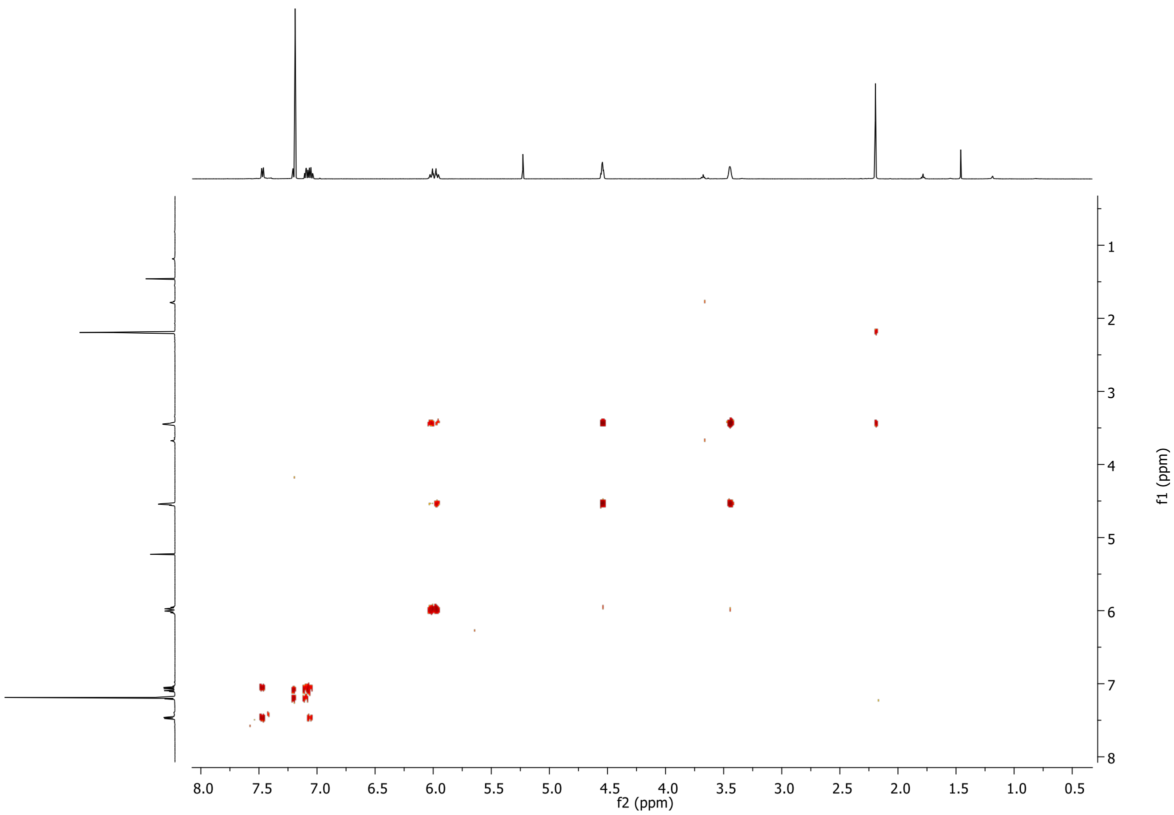

2D HSQC ($CDCl_3$)

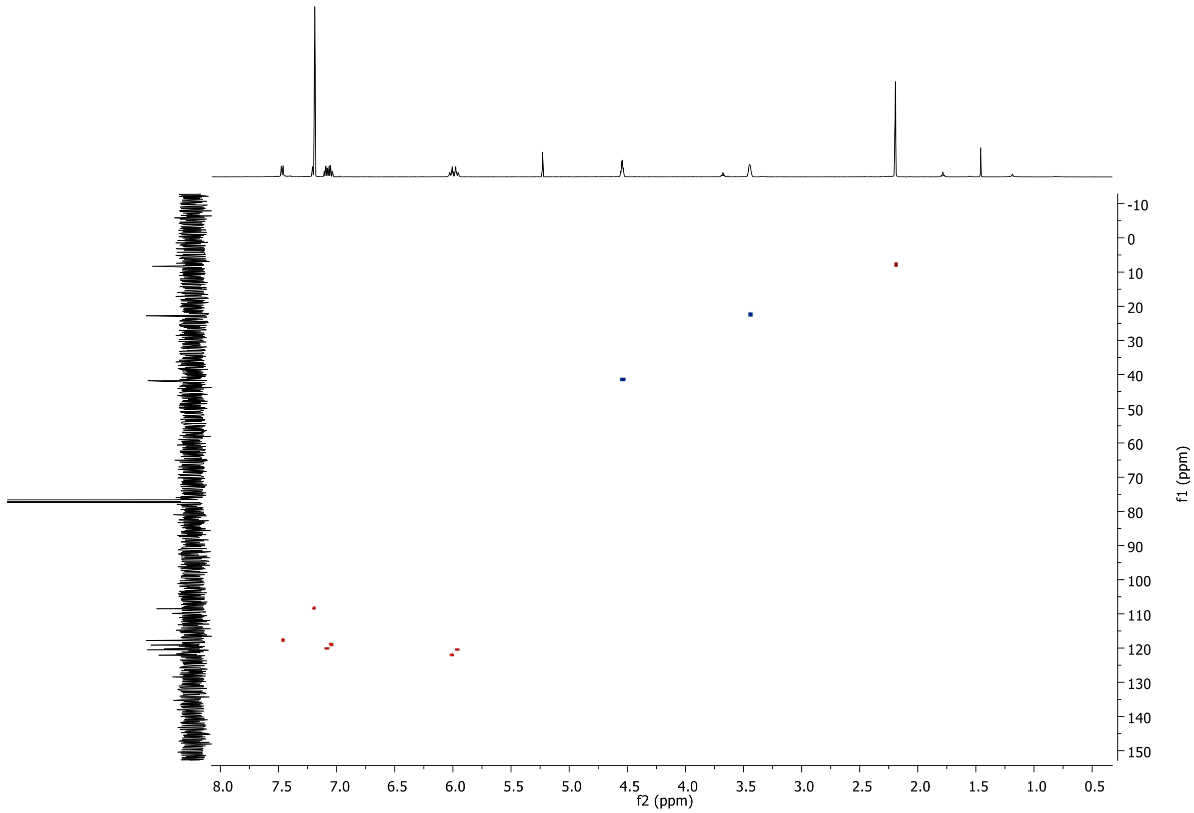

Cycle 4a’

^{1}H NMR (500 MHz, $CDCl_3$, 25°C, TMS)

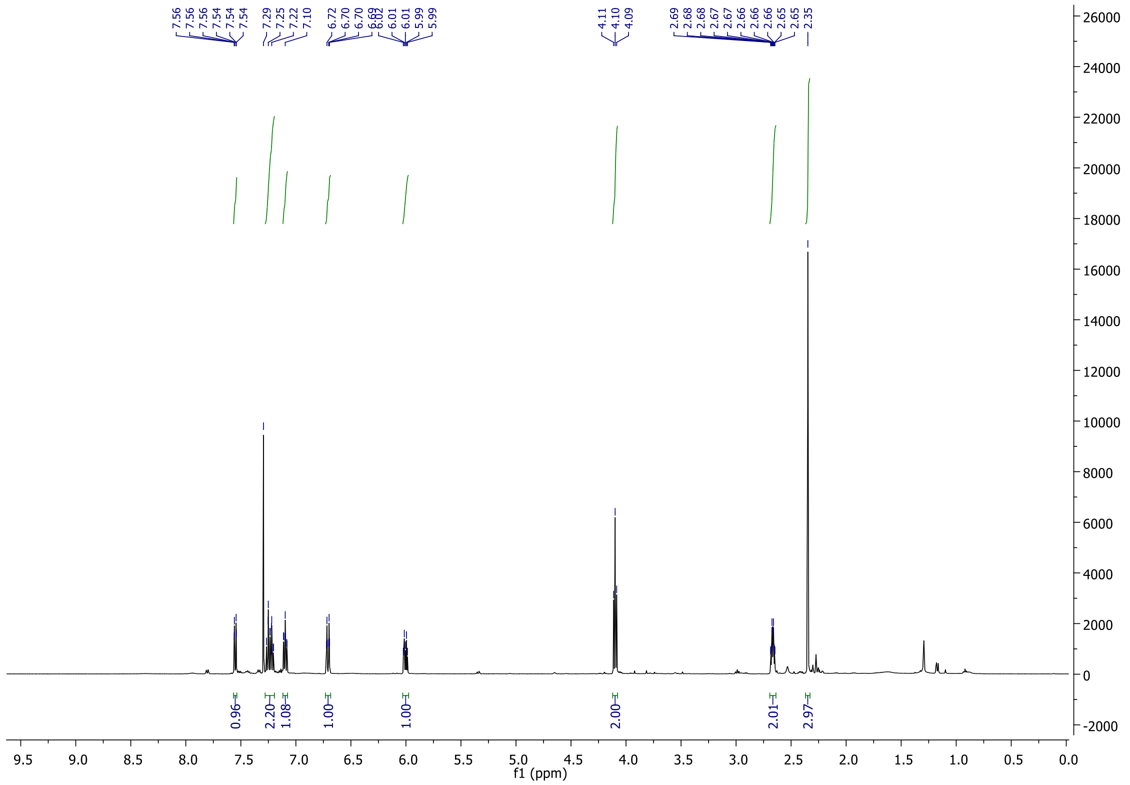

^{13}C NMR (126 MHz, $CDCl_3$, 25°C)

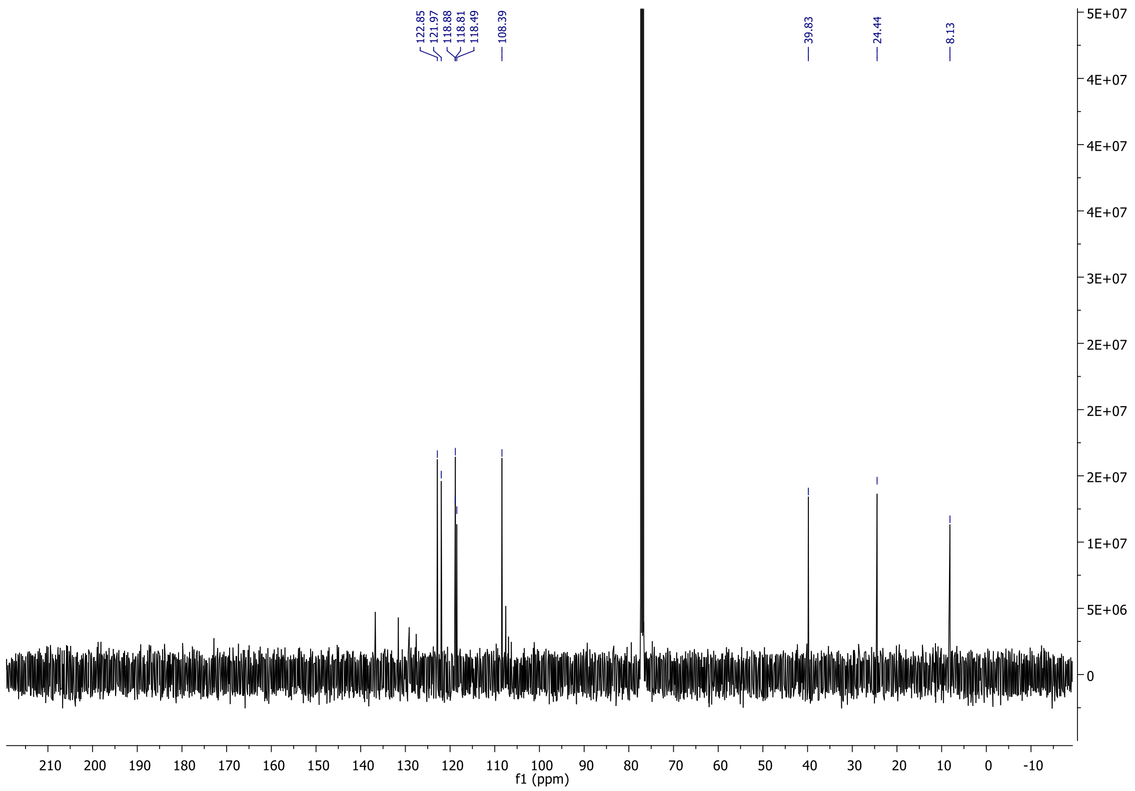

2D gCOSY ($CDCl_3$)

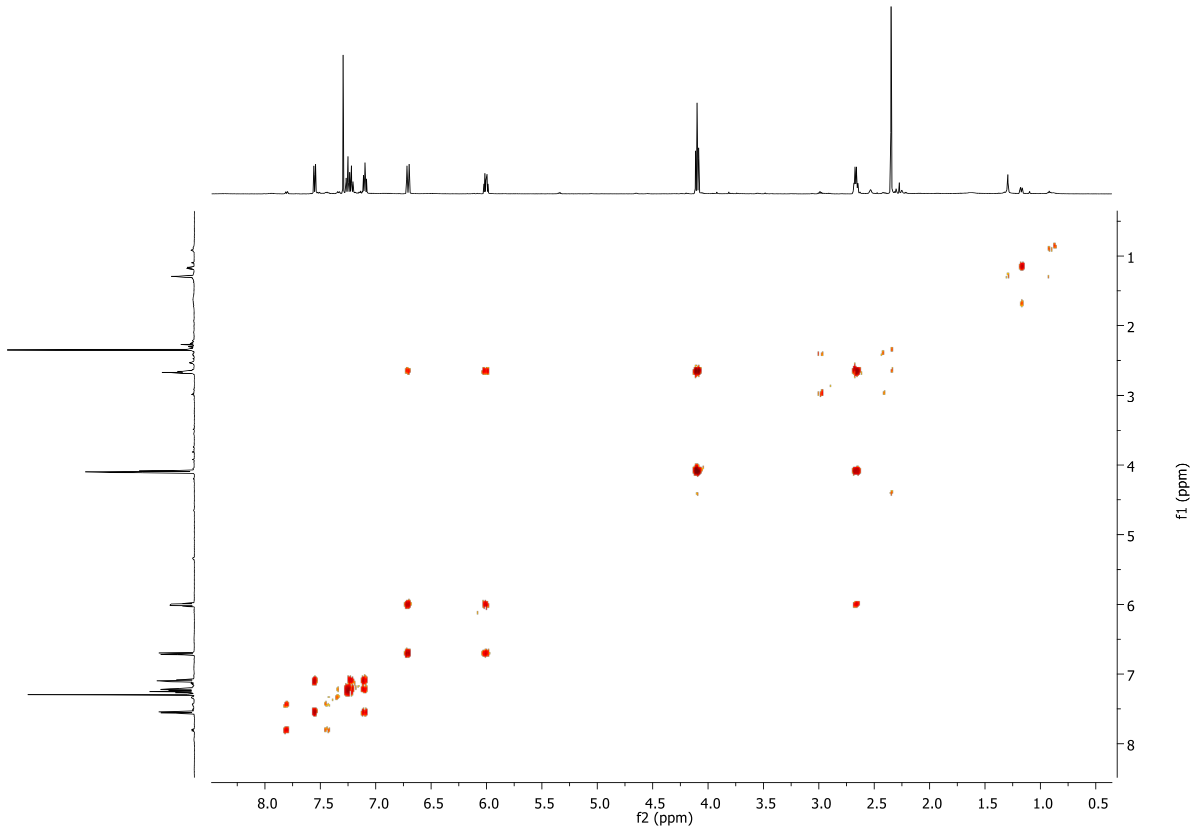

2D HSQC ($CDCl_3$)

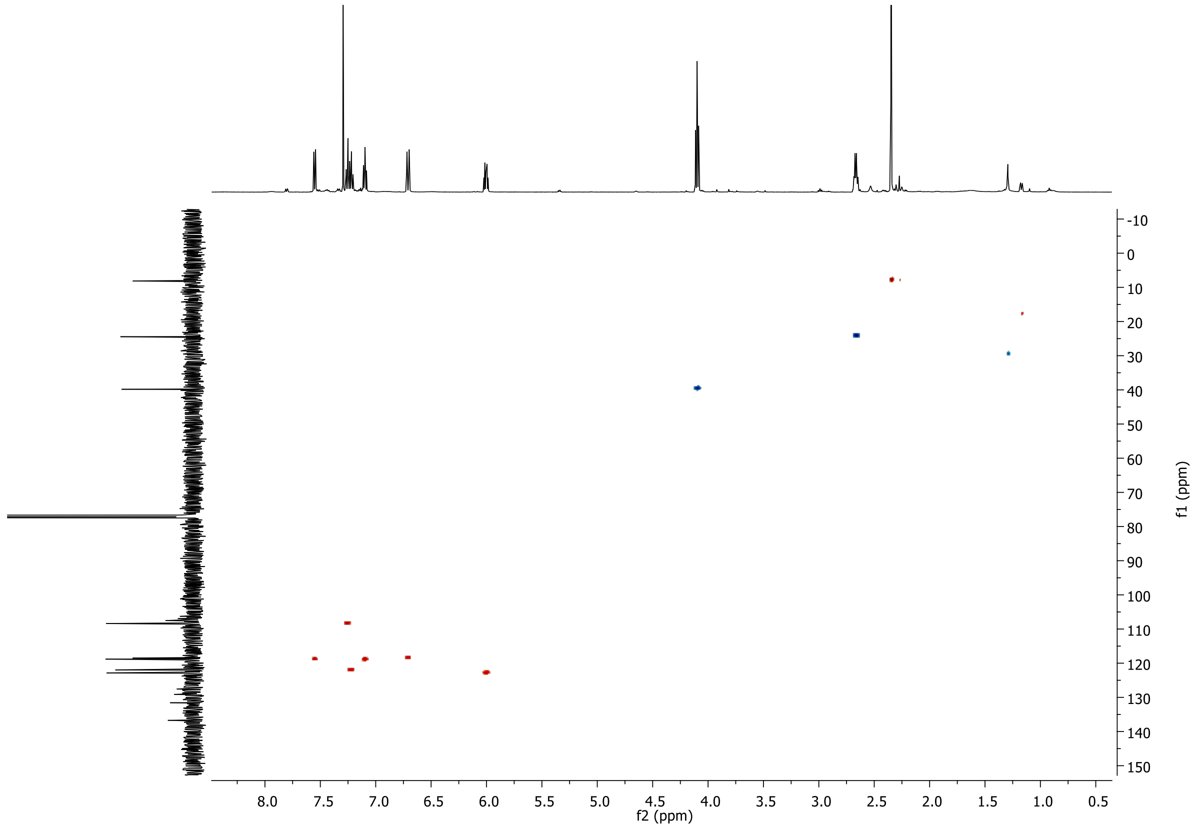

2,3’-BIM 3aa

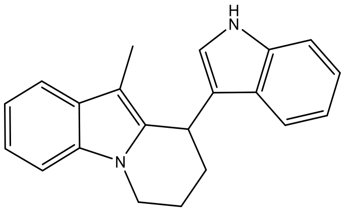

^1H NMR (500 MHz, $CDCl_3$, 25°C, TMS)

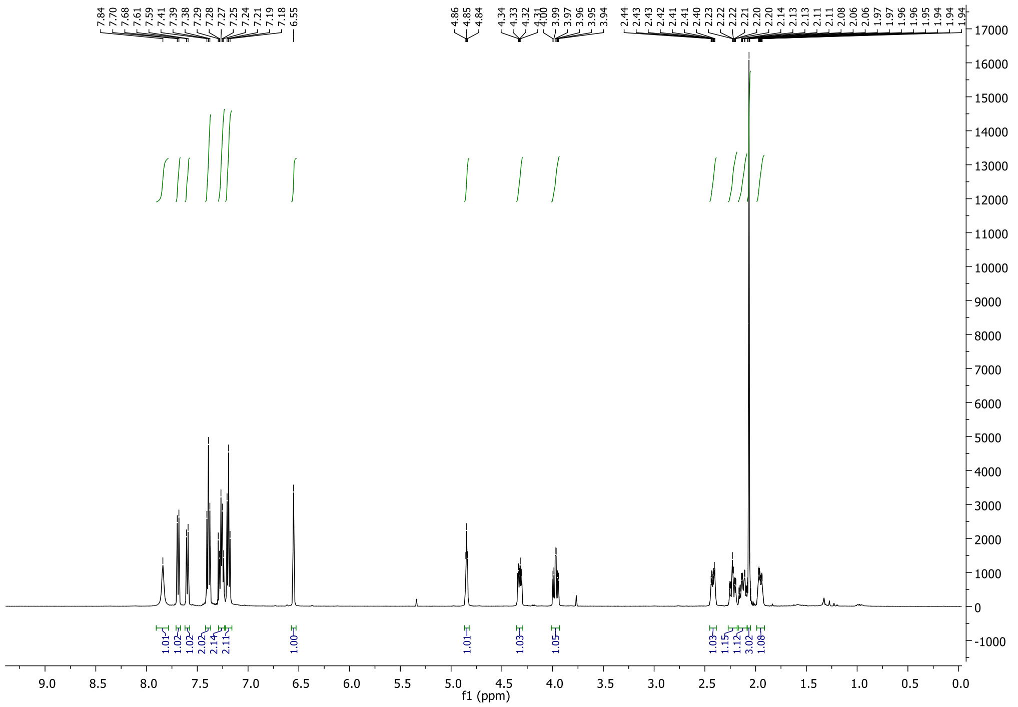

^{13}C NMR (126 MHz, $CDCl_3$, 25°C)

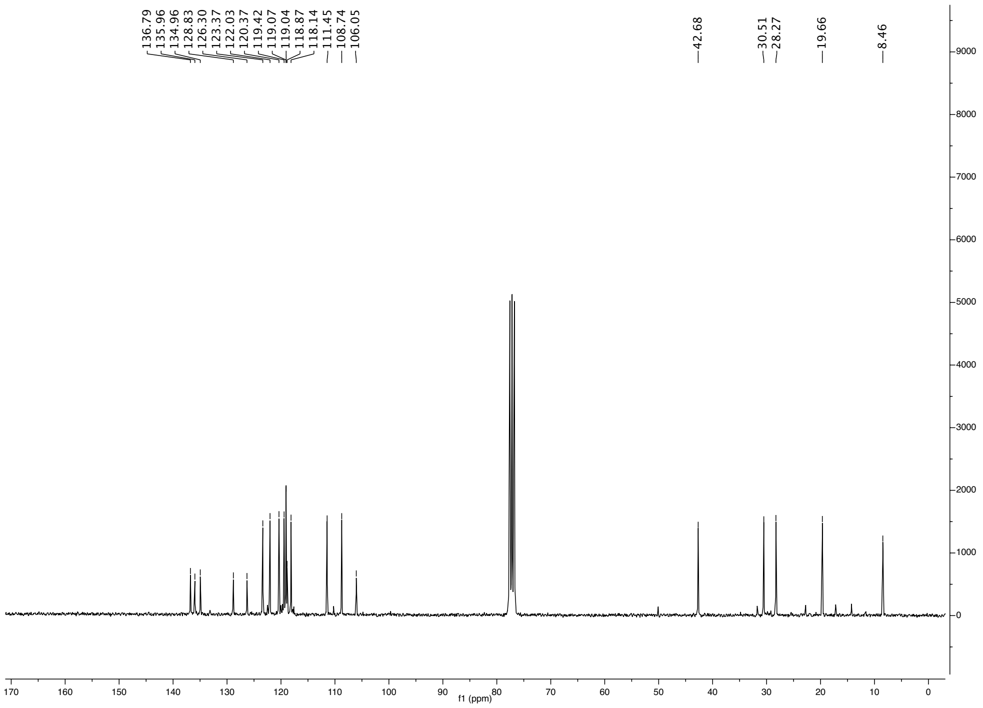

2,3'-BIM 3ba

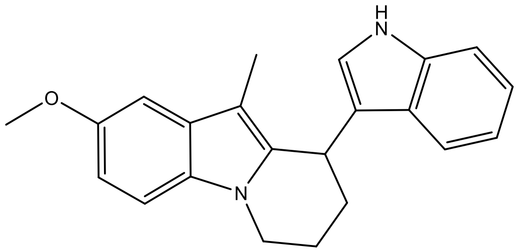

^{1}H NMR (500 MHz, $CDCl_3$, 25°C, TMS)

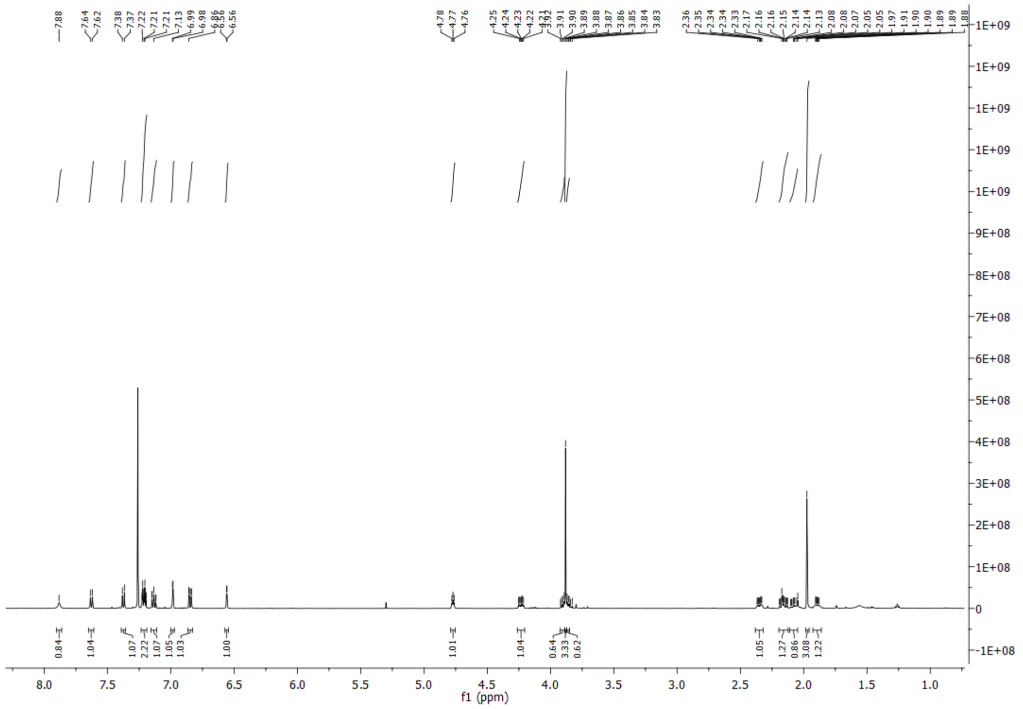

^{13}C NMR (126 MHz, $CDCl_3$, 25°C)

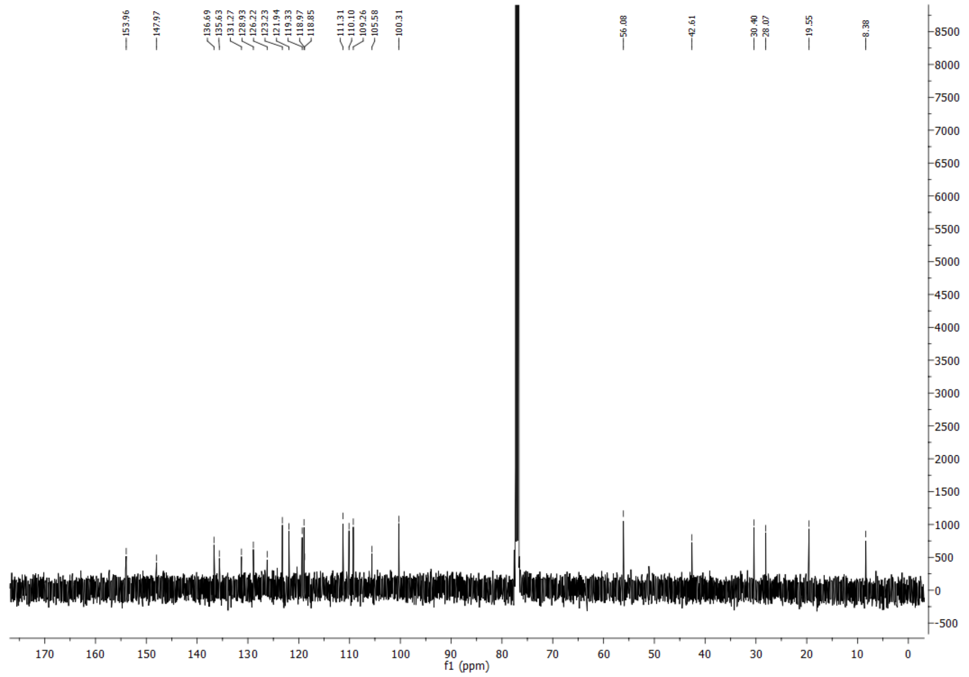

2D gCOSY ($CDCl_3$)

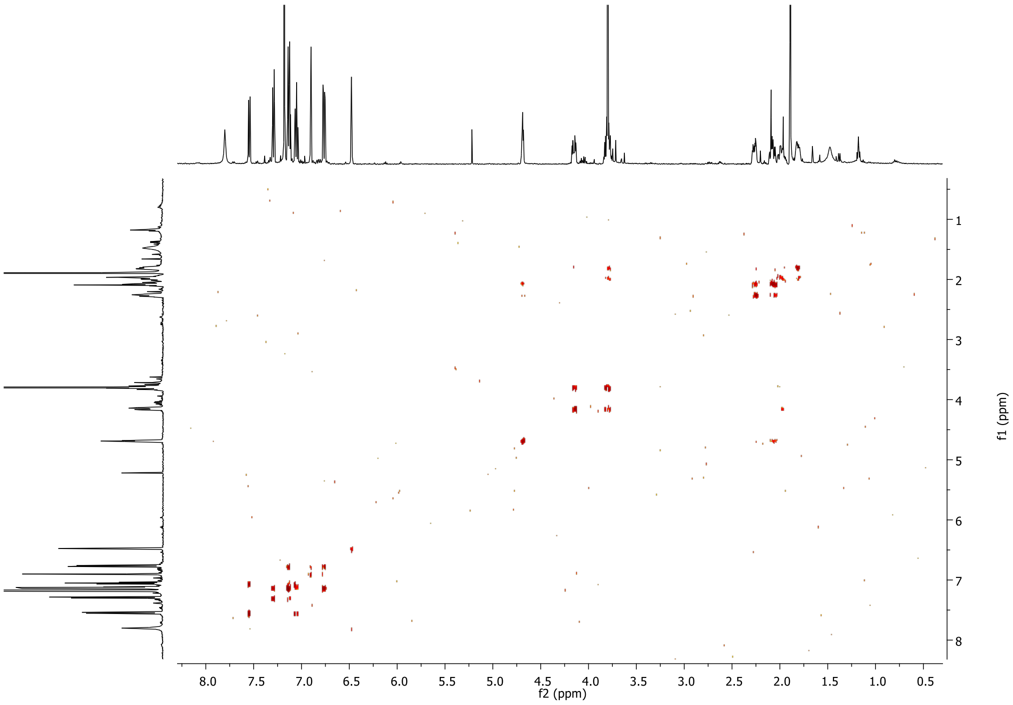

2D HSQC ($CDCl_3$)

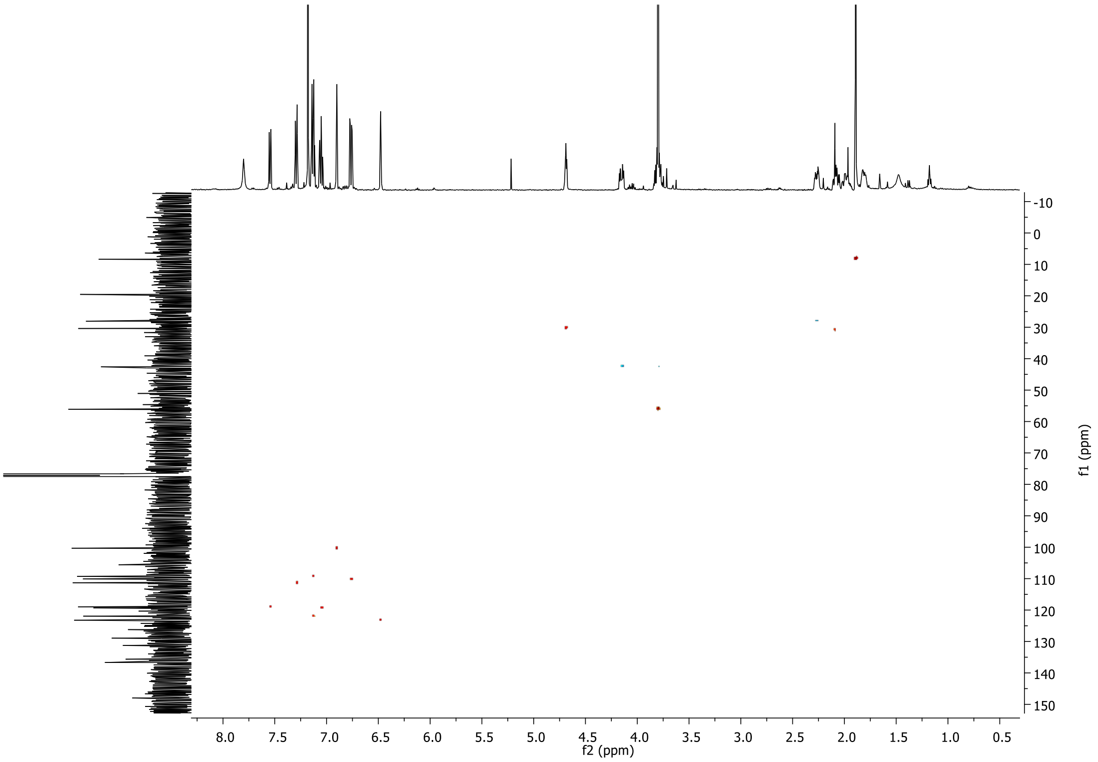

2,3-BIM 3ca

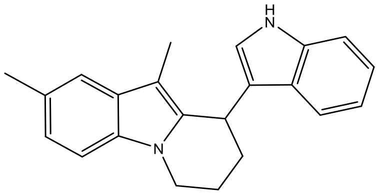

^{1}H NMR (500 MHz, $CDCl_3$, 25°C, TMS)

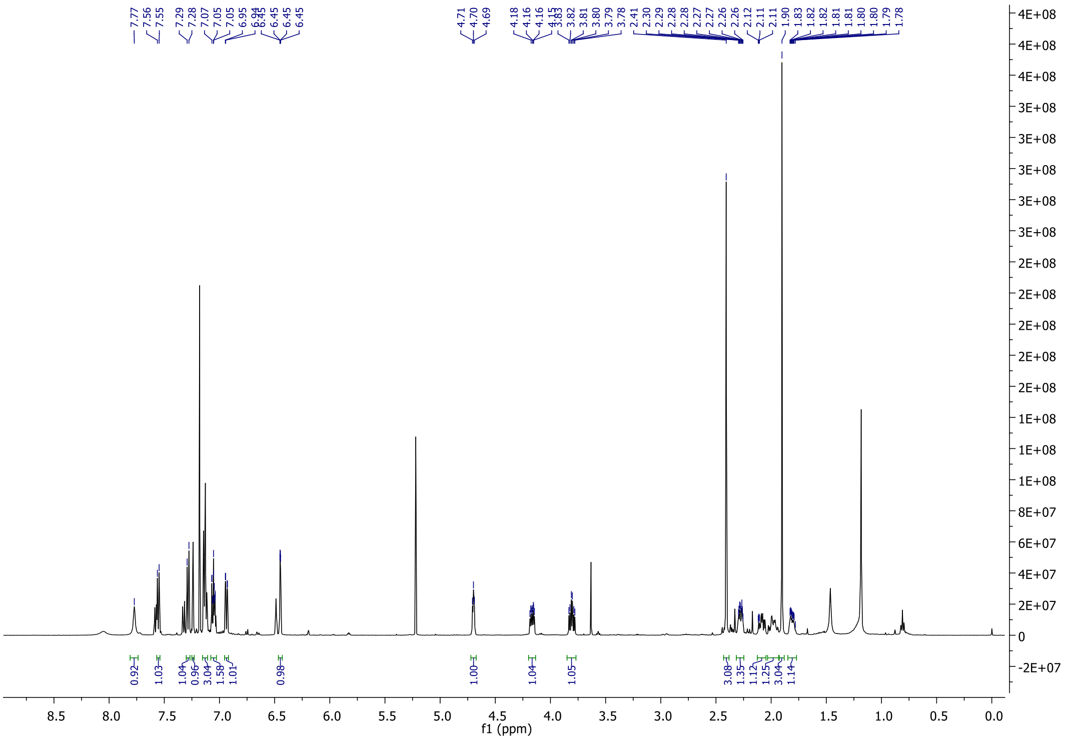

^{13}C NMR (126 MHz, $CDCl_3$, 25°C)

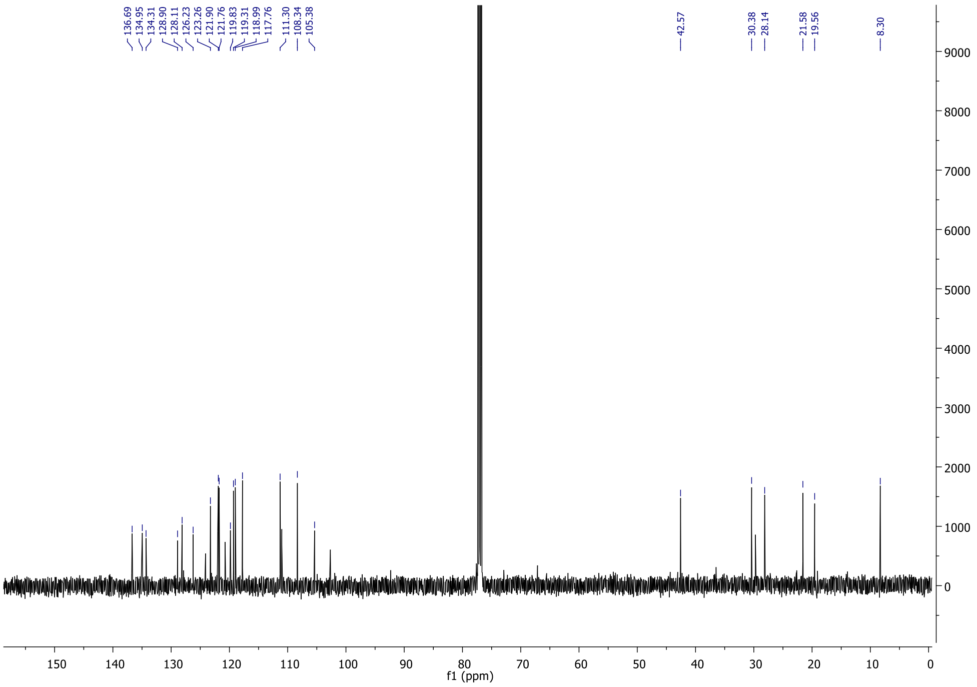

2D gCOSY ($CDCl_3$)

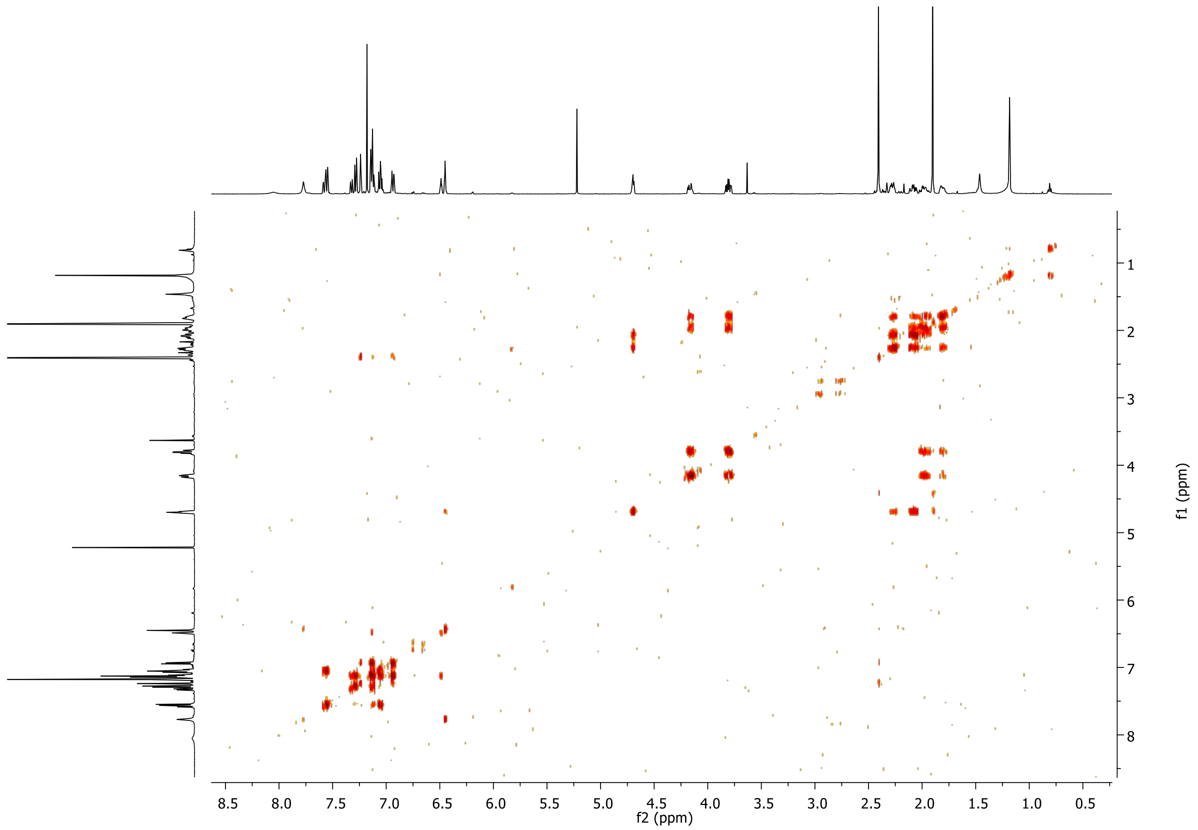

2D HSQC ($CDCl_3$)

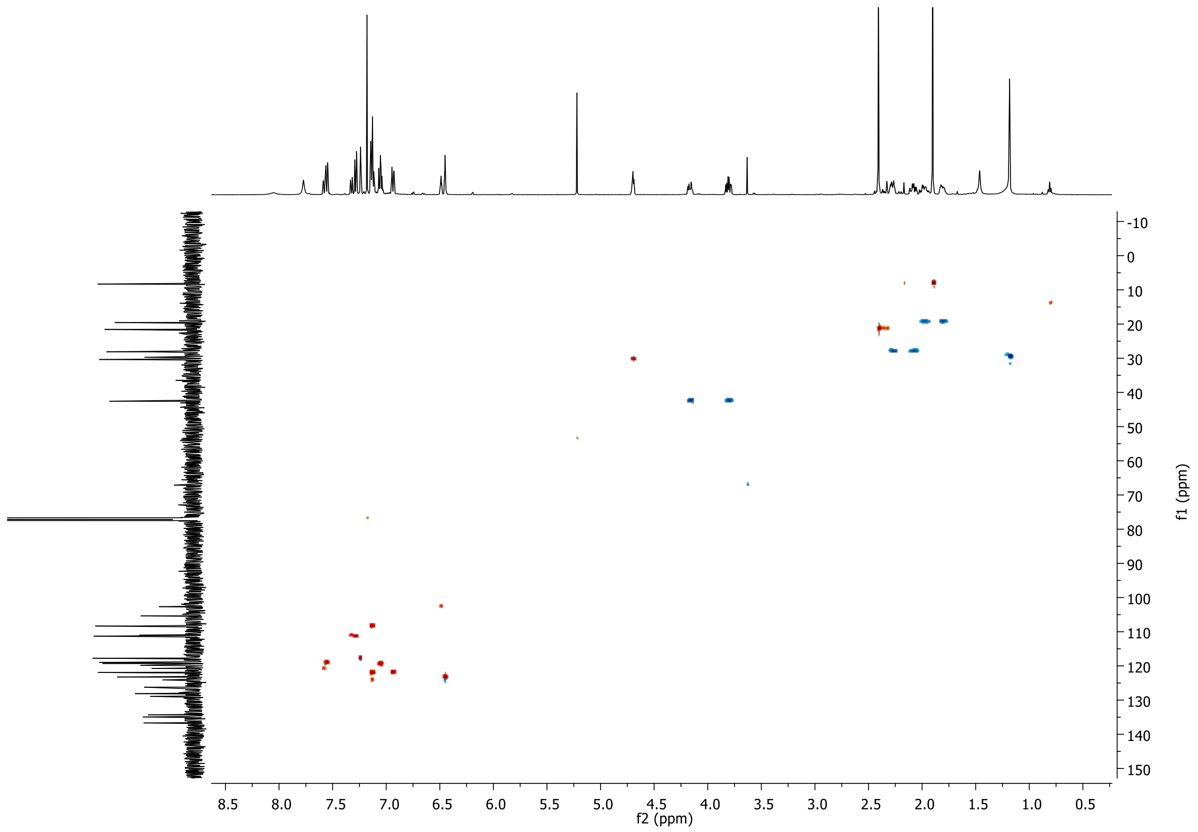

2,3'-BIM 3da

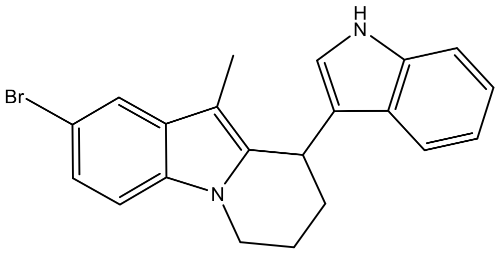

^{1}H NMR (500 MHz, $CDCl_3$, 25°C, TMS)

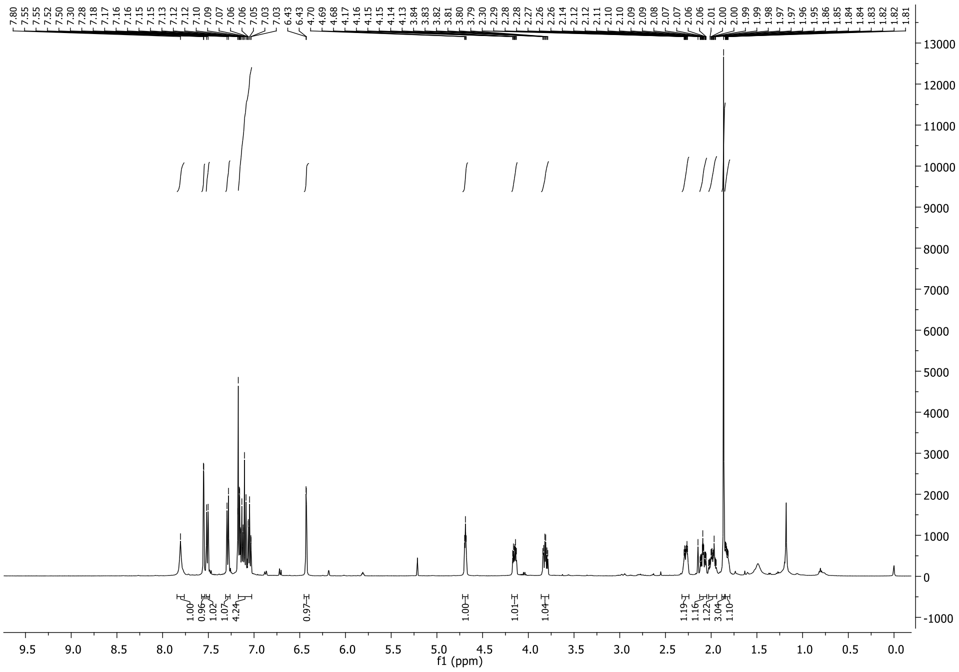

^{13}C NMR (126 MHz, $CDCl_3$, 25°C)

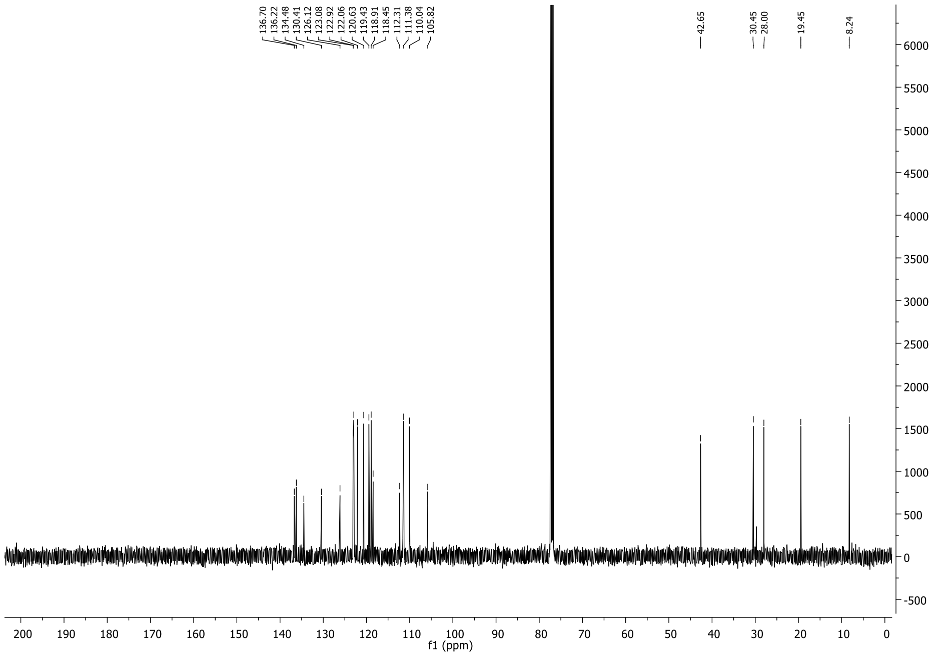

2D gCOSY ($CDCl_3$)

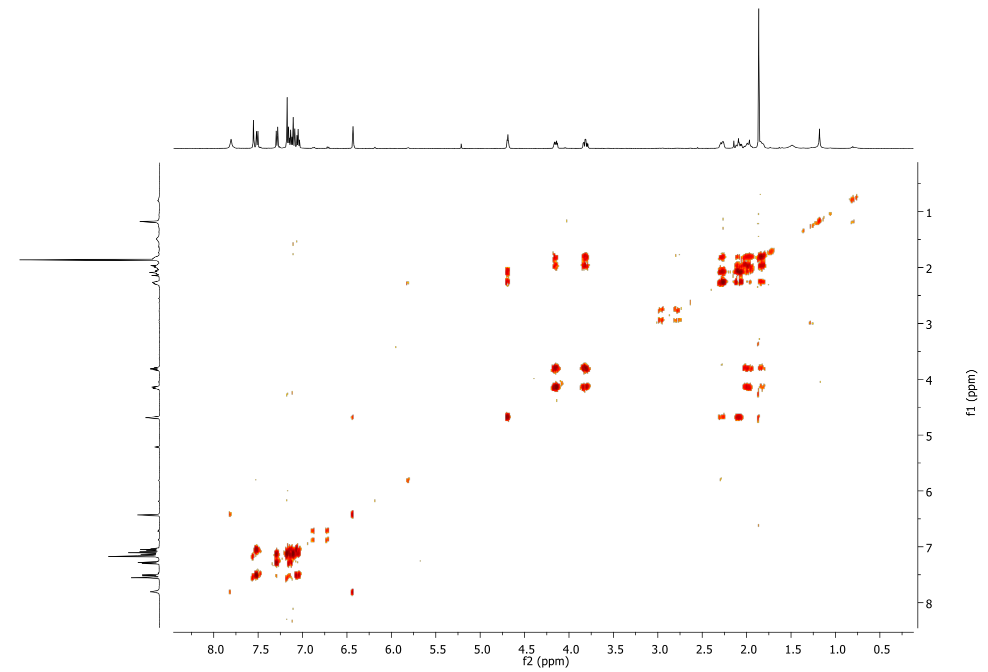

2D HSQC ($CDCl_3$)

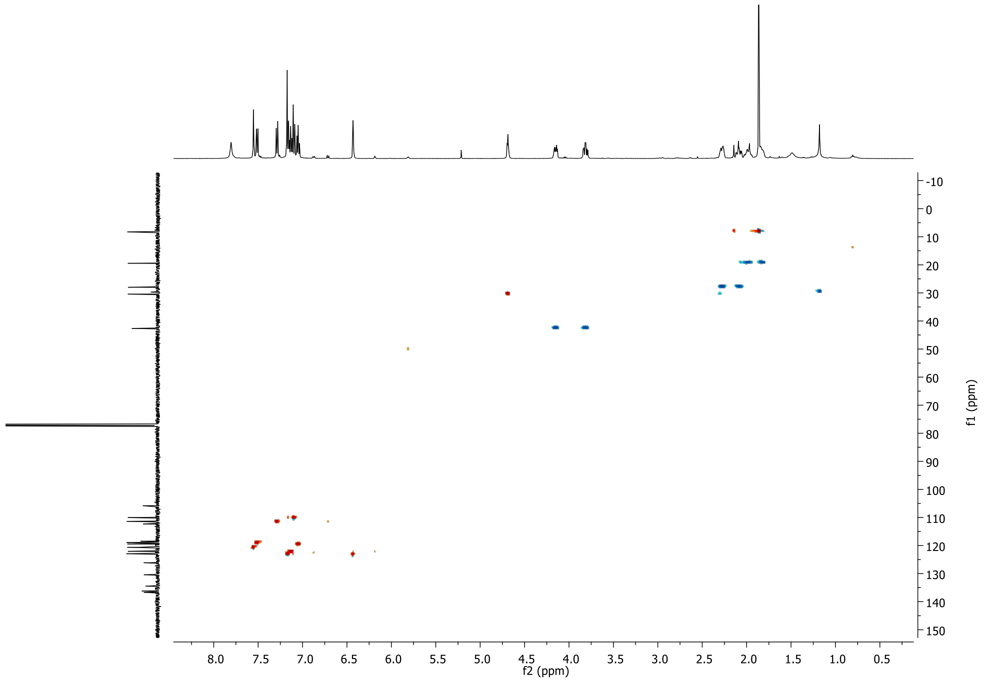

Cycles 4d:4d' (1:0.95)

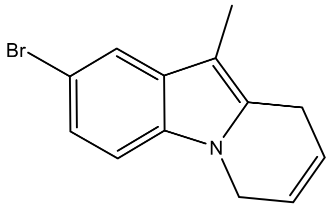

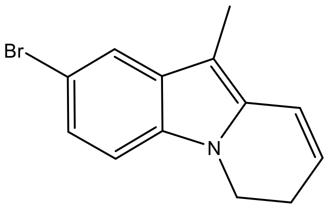

^{1}H NMR (500 MHz, $CDCl_3$, 25°C, TMS)

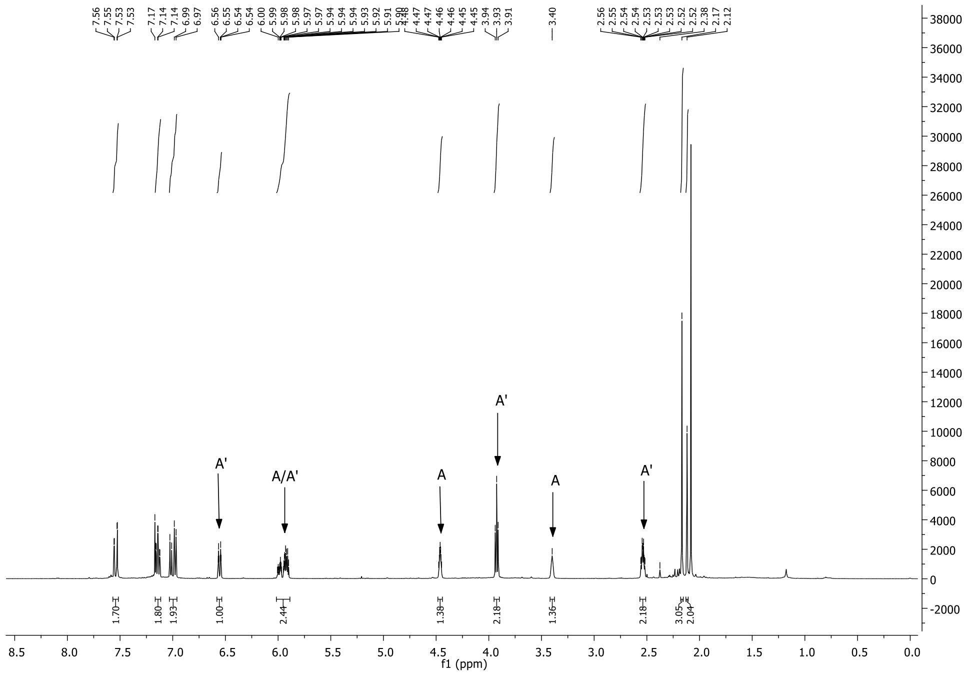

^{13}C NMR (126 MHz, $CDCl_3$, 25°C)

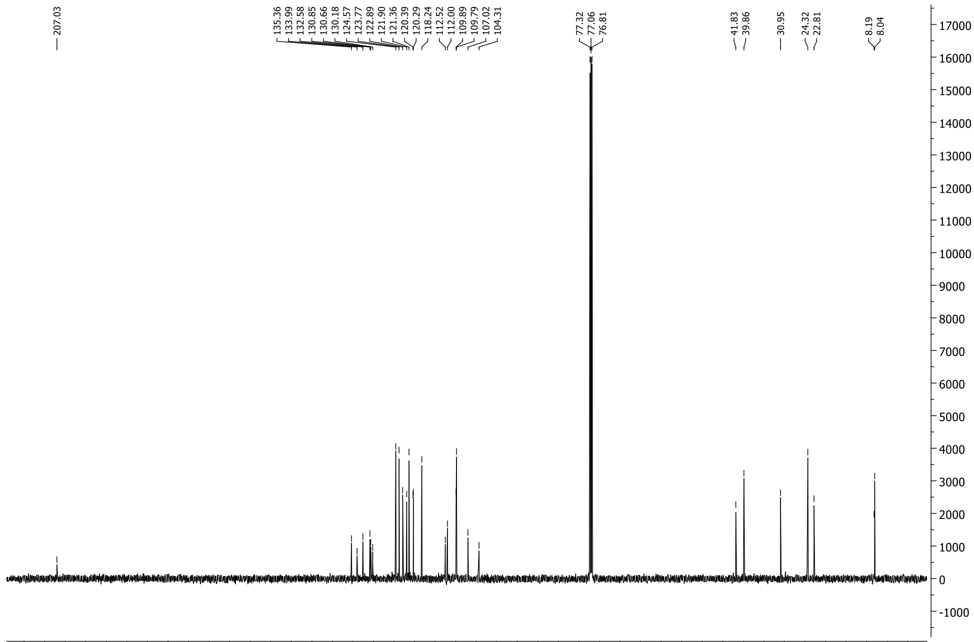

2D gCOSY ($CDCl_3$)

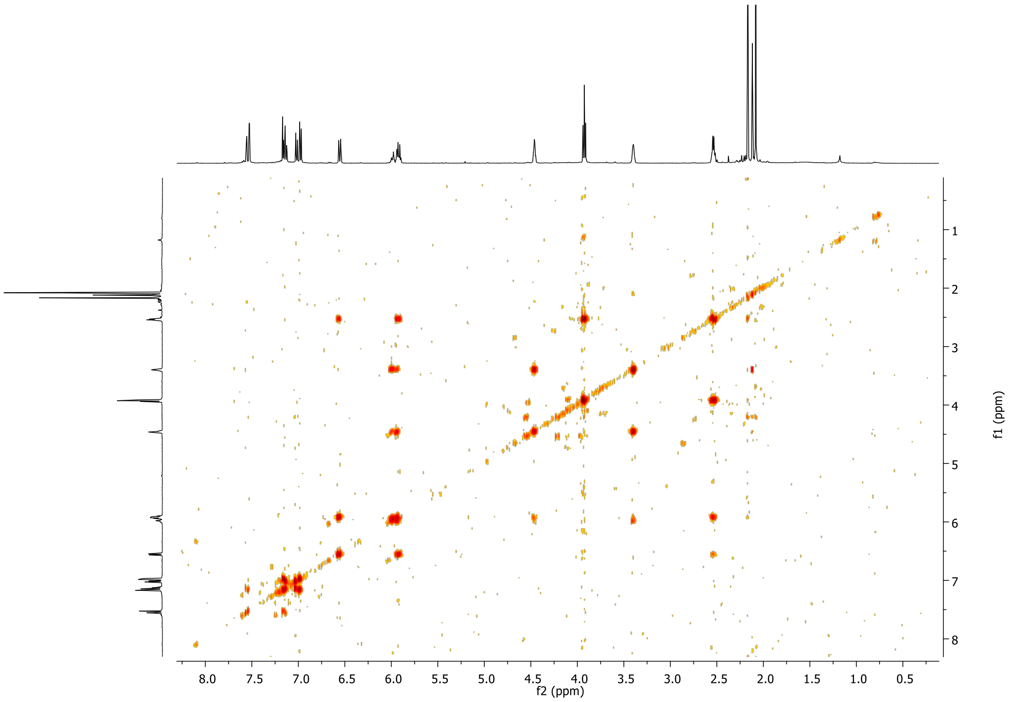

2D HSQC ($CDCl_3$)

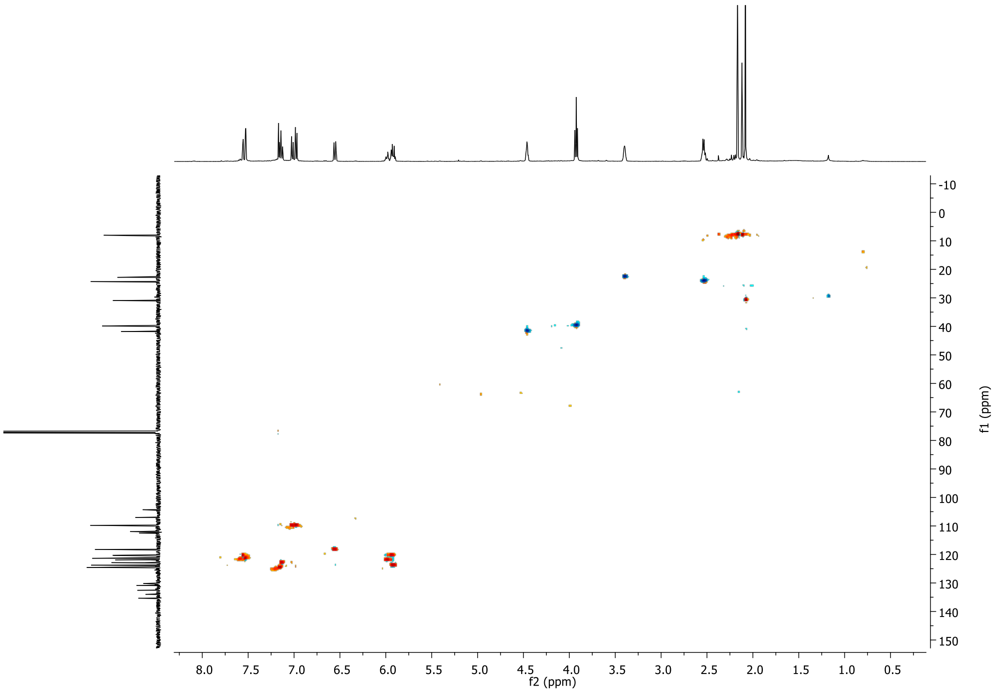

Supporting Information

Cycles 4e:4e' (0.78:1)

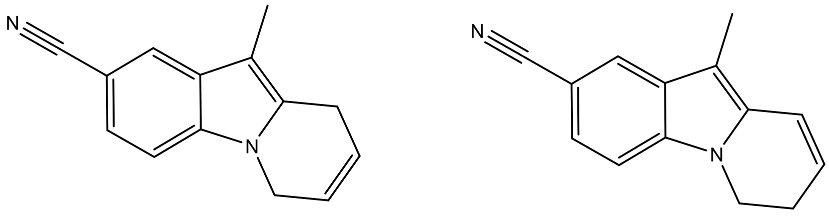

^{1}H NMR (500 MHz, CDCl$_3$, 25°C, TMS)

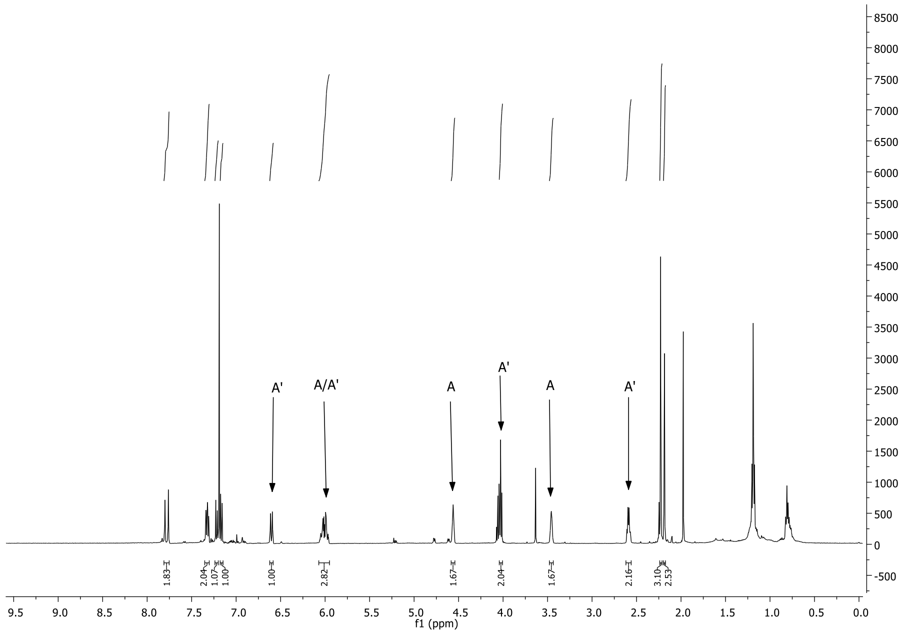

^{13}C NMR (126 MHz, CDCl$_3$, 25°C)

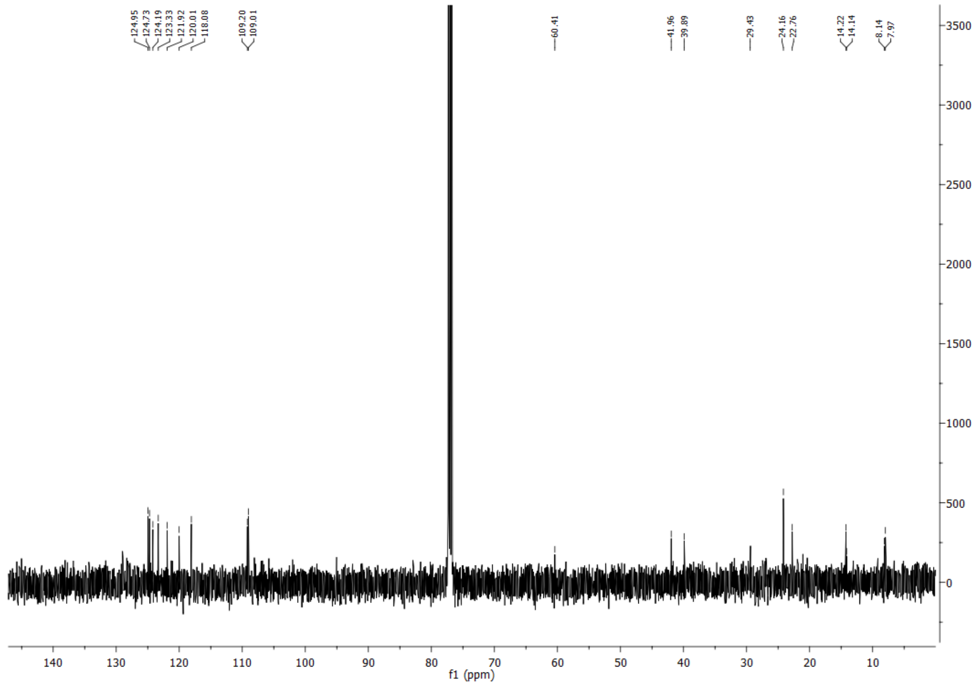

2D gCOSY ($CDCl_3$)

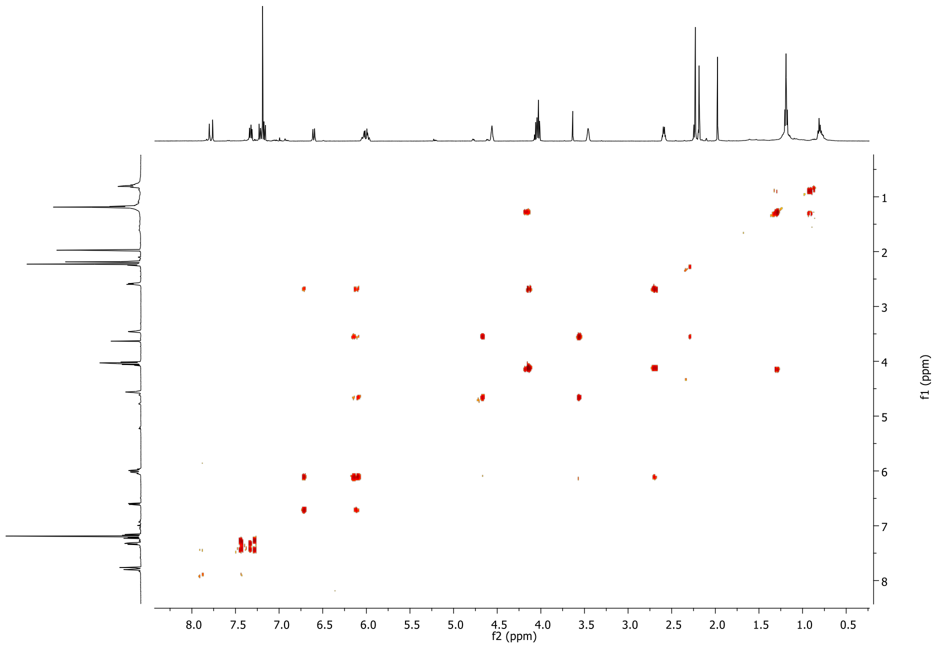

2D HSQC ($CDCl_3$)

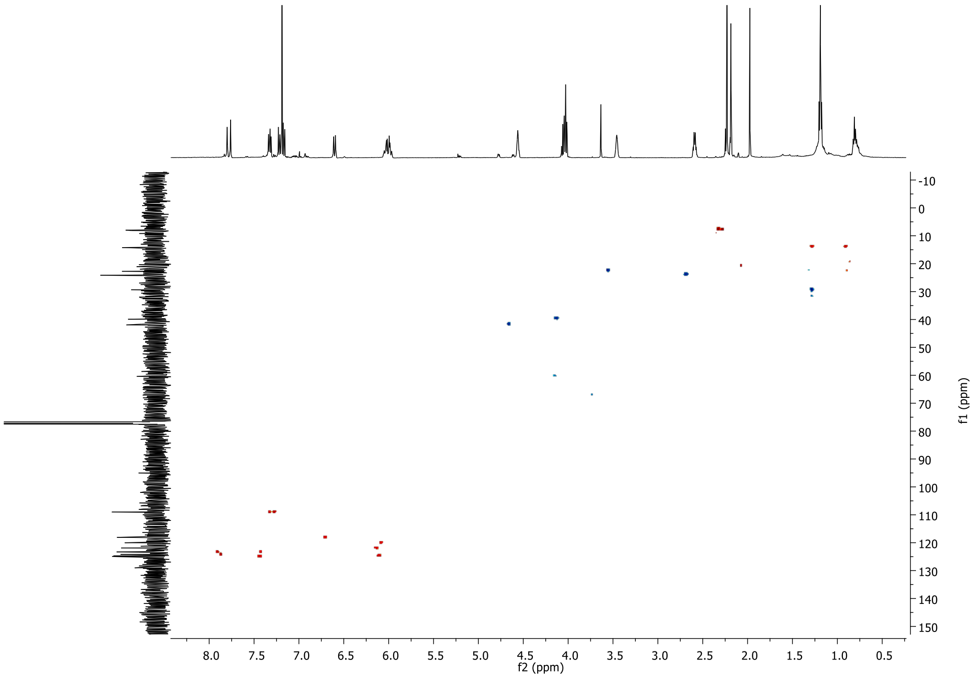

Cycles 4f:4f' (0.8:1)

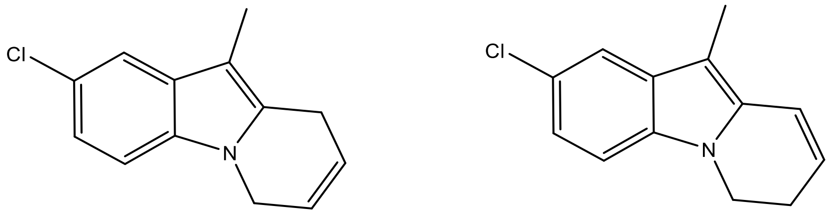

^{1}H NMR (500 MHz, $CDCl_3$, 25°C, TMS)

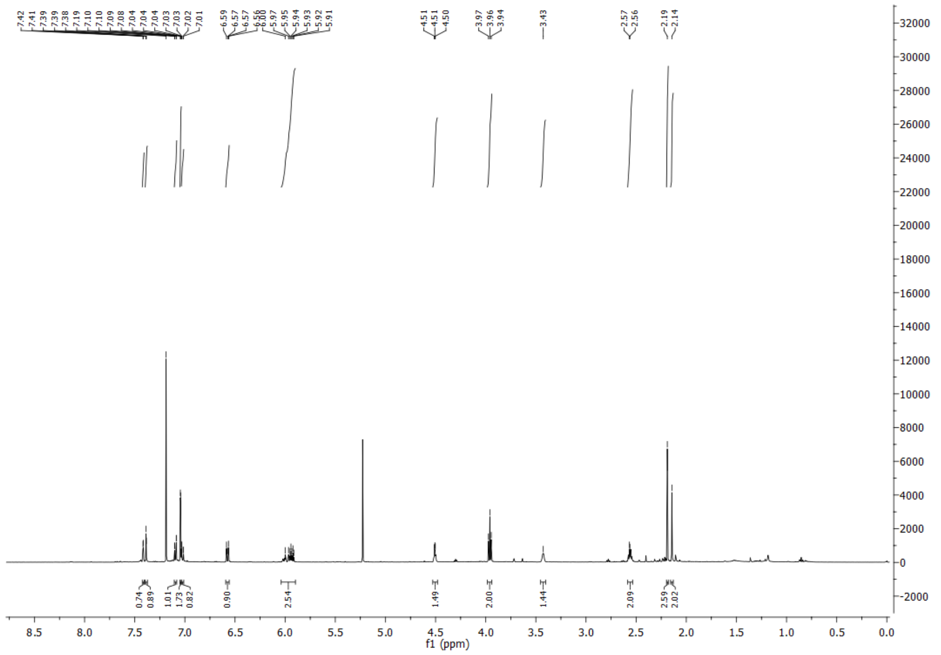

^{13}C NMR (126 MHz, $CDCl_3$, 25°C)

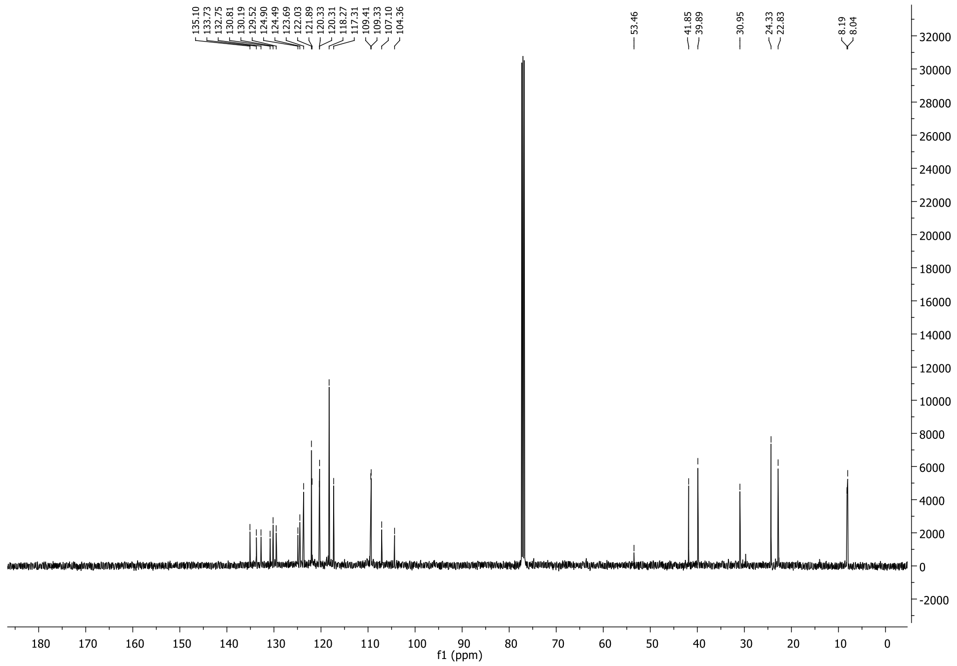

2D gCOSY ($CDCl_3$)

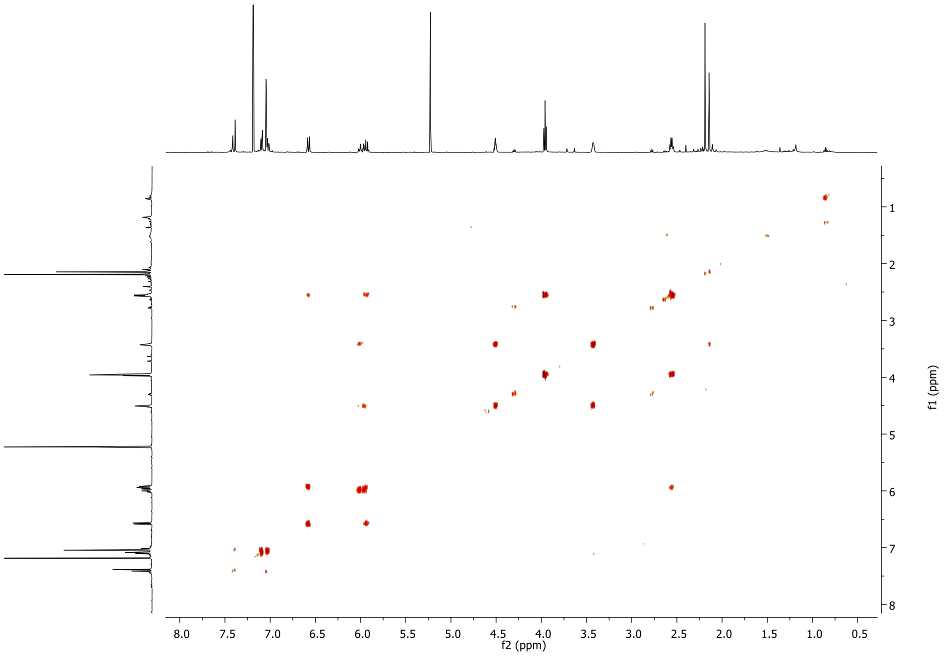

2D HSQC ($CDCl_3$)

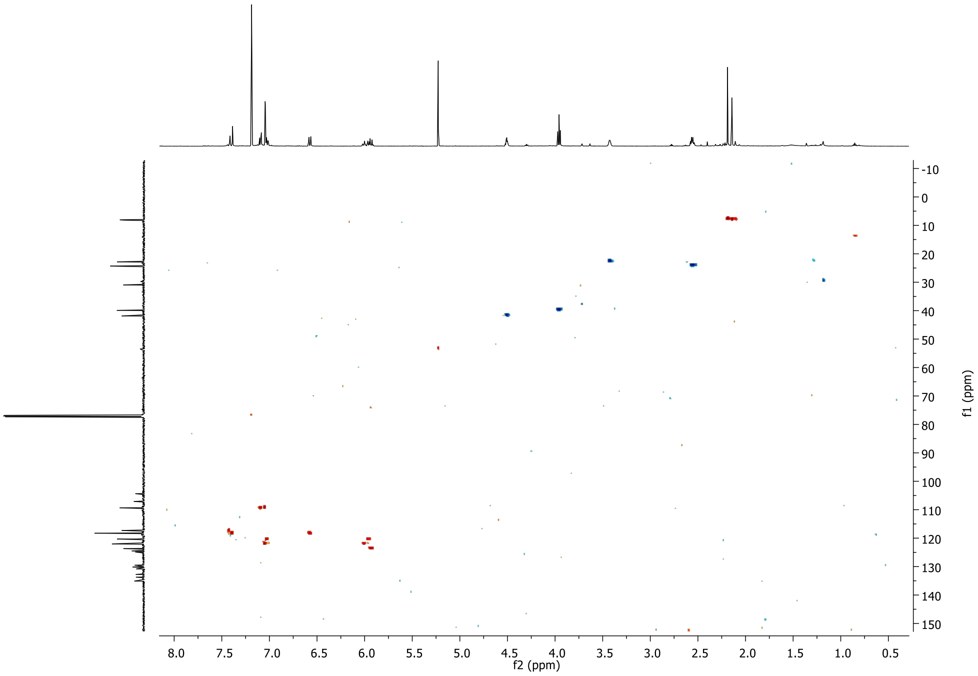

2,3'-BIM 3ab

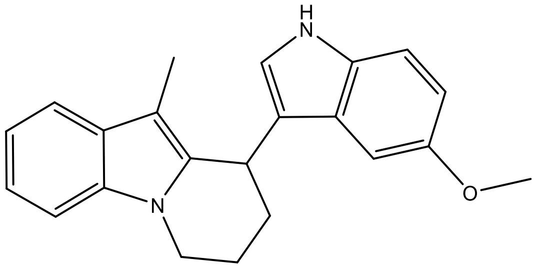

^{1}H NMR (500 MHz, $CDCl_3$, 25°C, TMS)

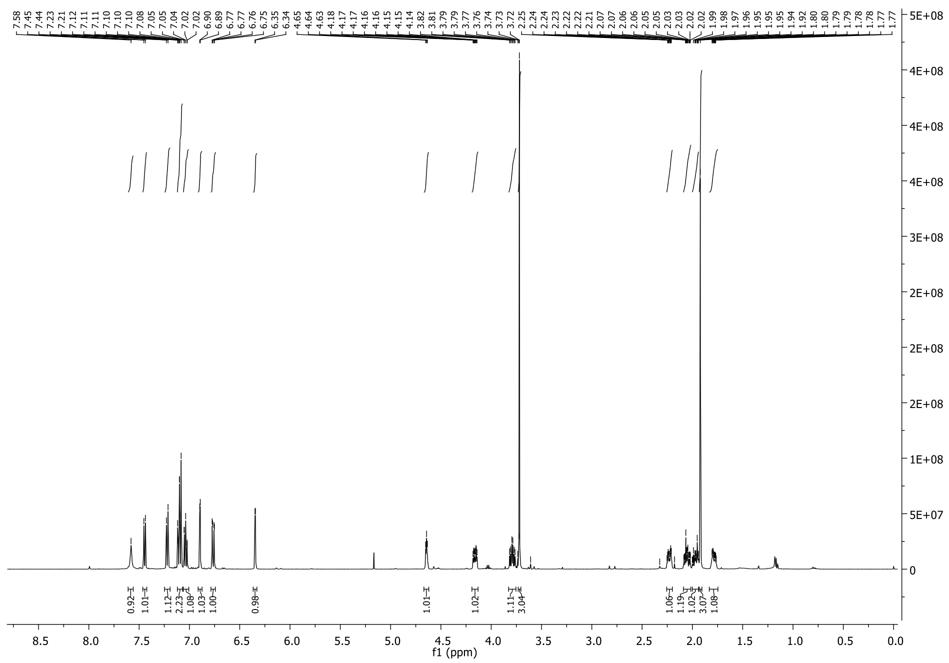

^{13}C NMR (126 MHz, $CDCl_3$, 25°C)

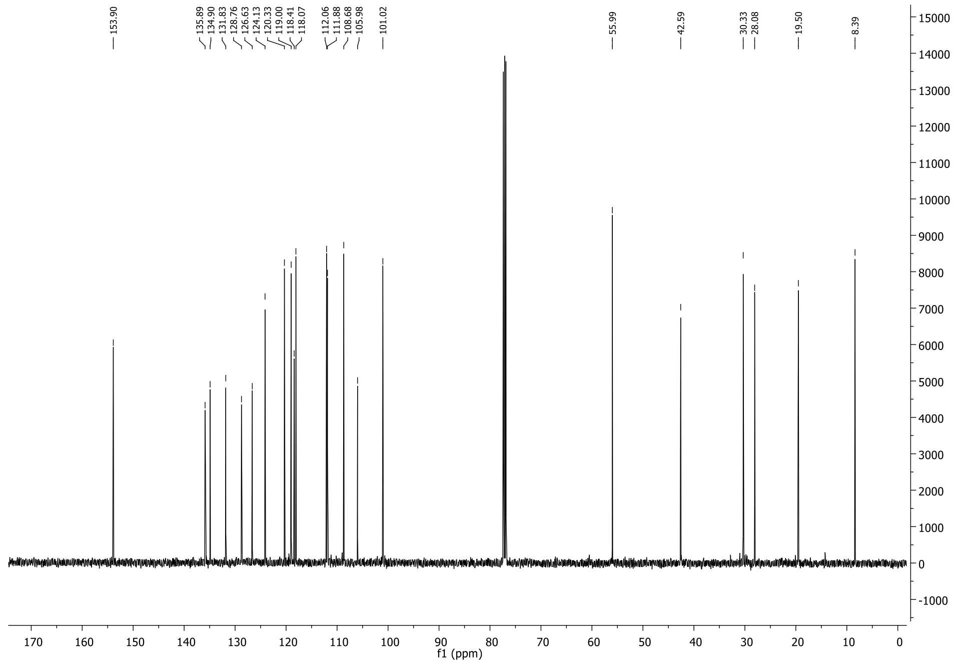

2D gCOSY ($CDCl_3$)

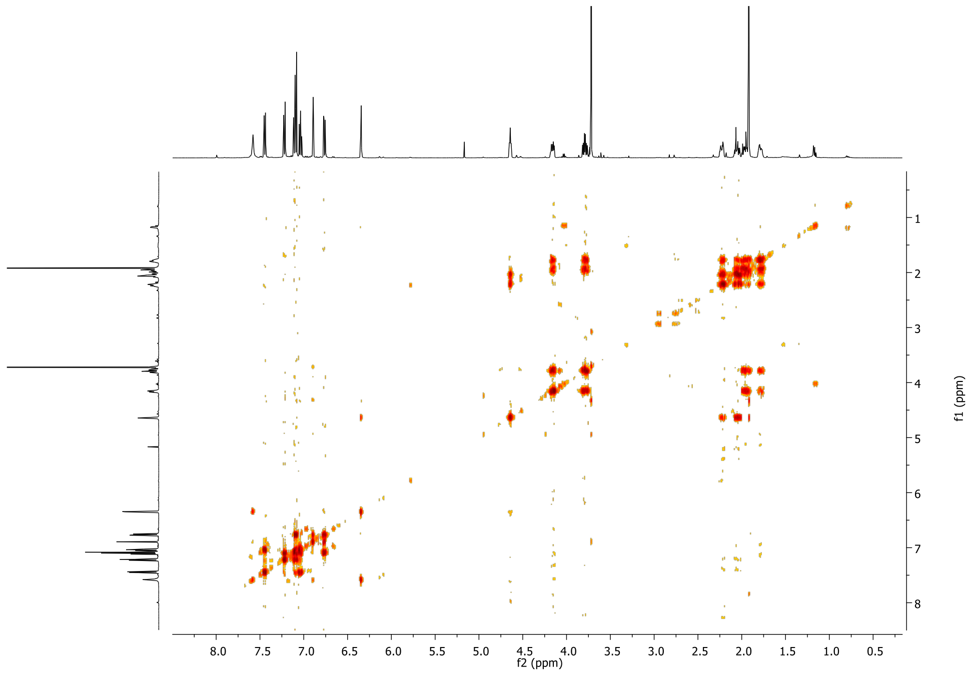

2D HSQC ($CDCl_3$)

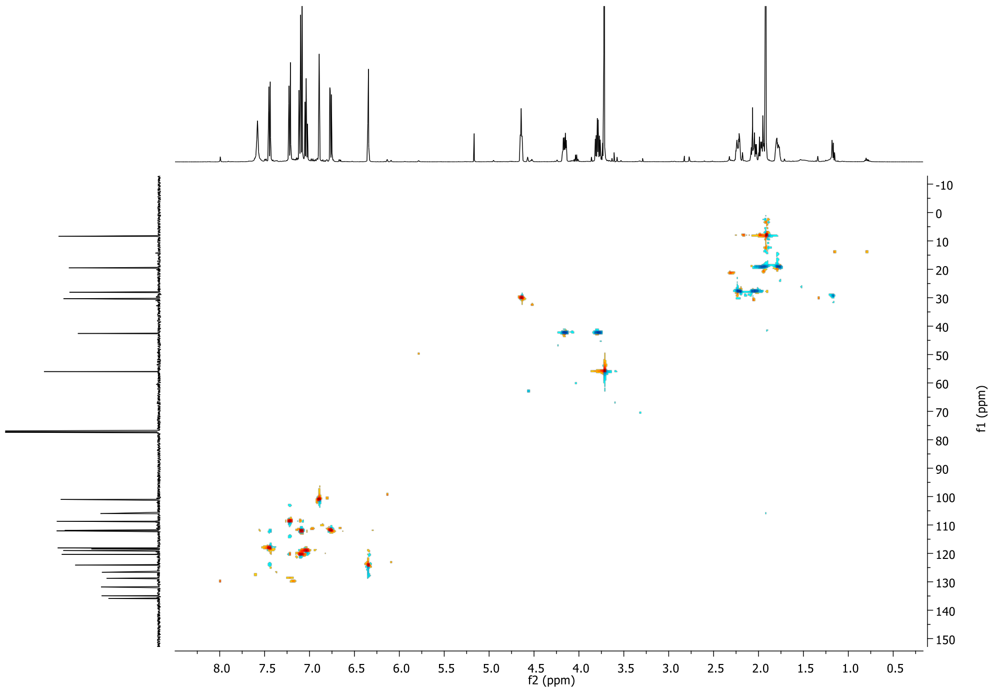

2,3'-BIM 3ac

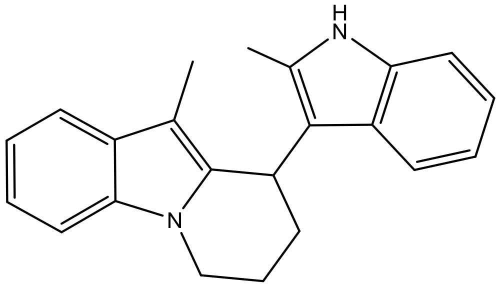

^{1}H NMR (500 MHz, $CDCl_3$, 25°C, TMS)

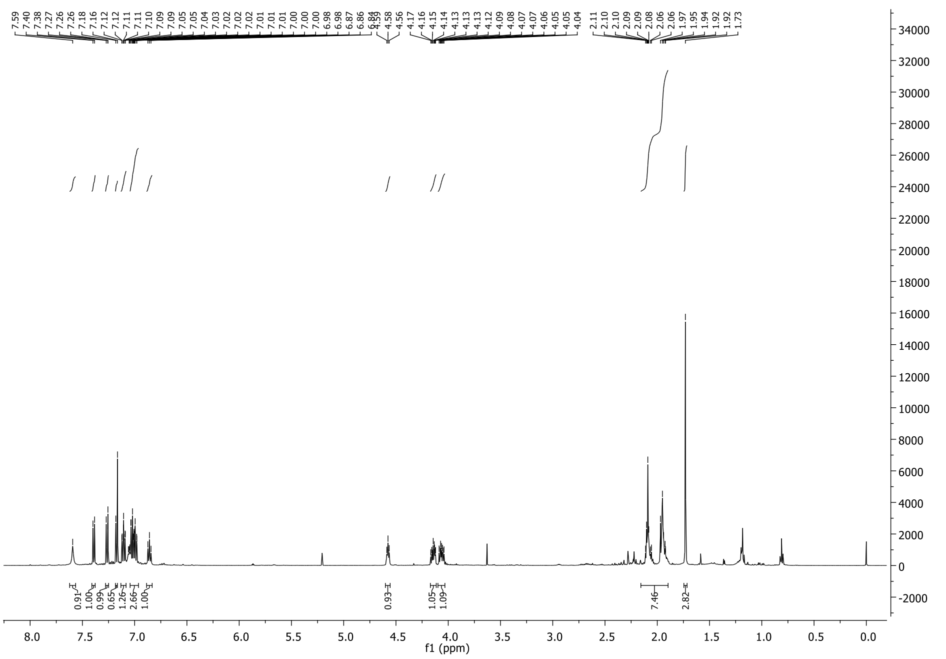

^{13}C NMR (126 MHz, $CDCl_3$, 25°C)

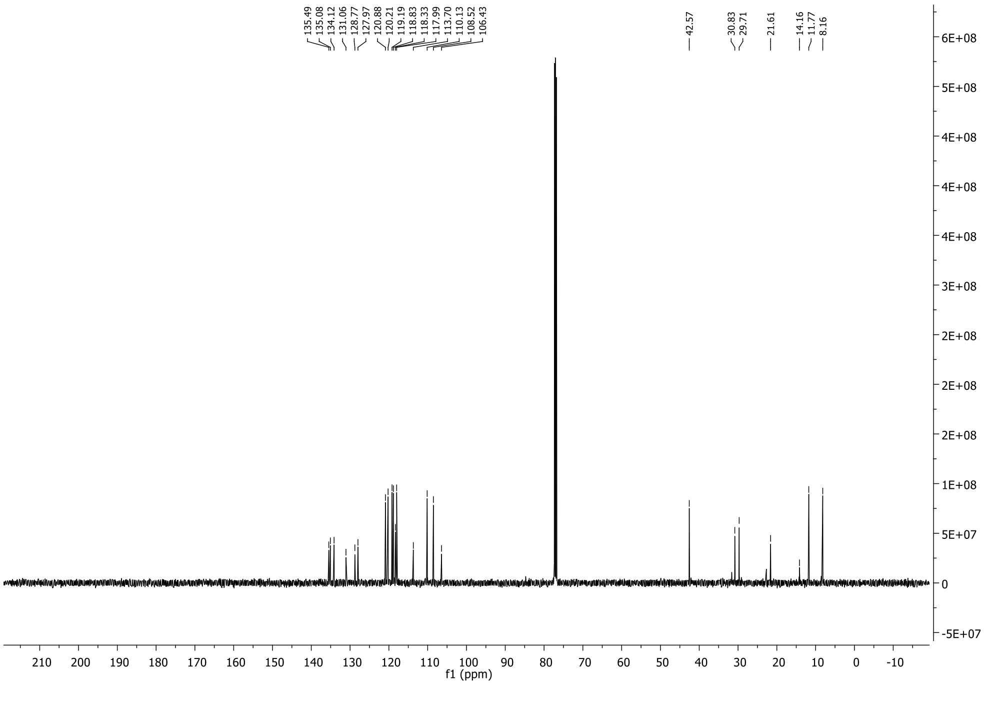

Supporting Information

2D gCOSY ($CDCl_3$)

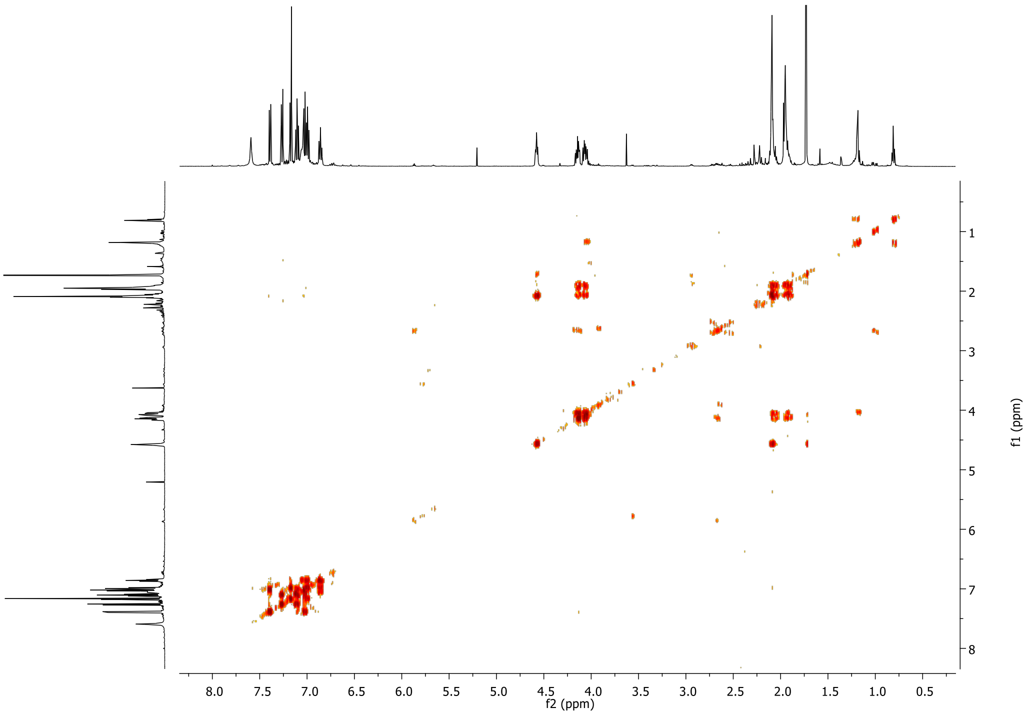

2D HSQC ($CDCl_3$)

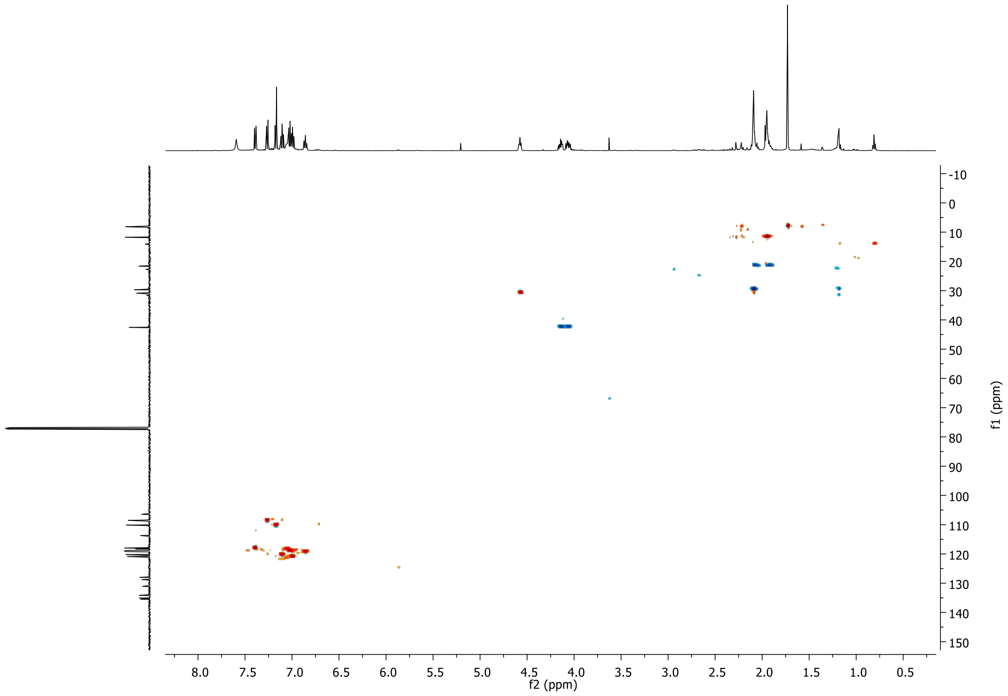

2,3'-BIM 3ad

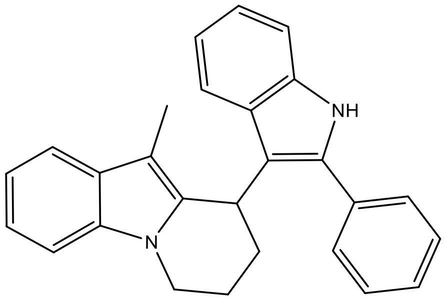

^{1}H NMR (500 MHz, $CDCl_3$, 25°C, TMS)

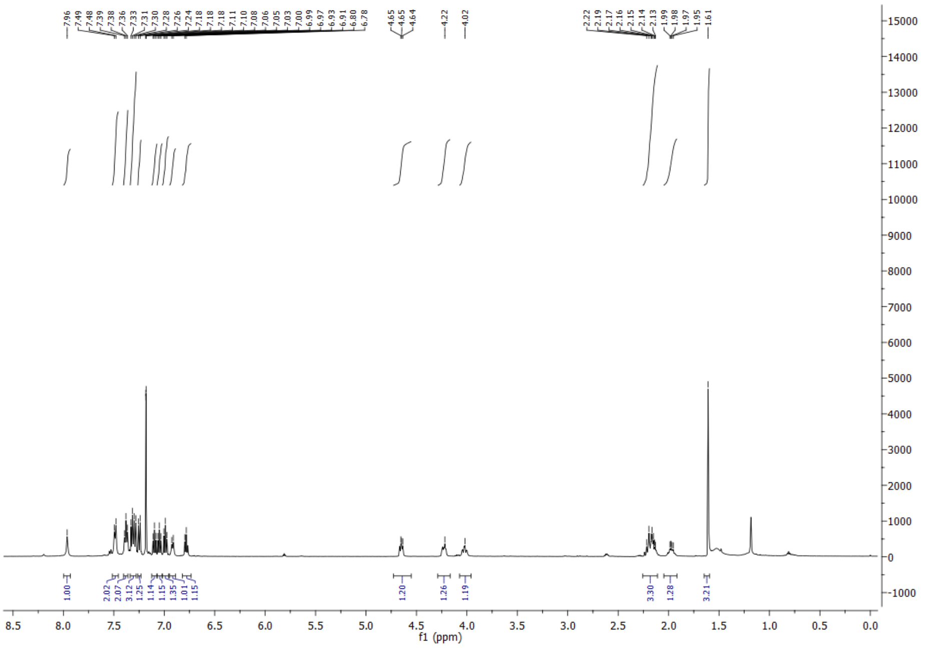

^{13}C NMR (126 MHz, $CDCl_3$, 25°C)

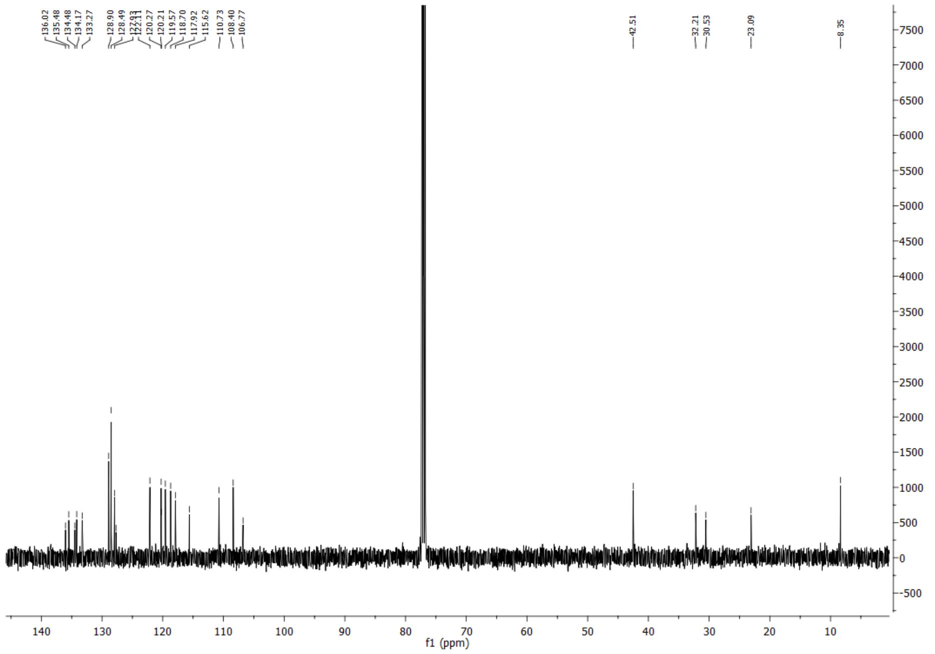

2D gCOSY ($CDCl_3$)

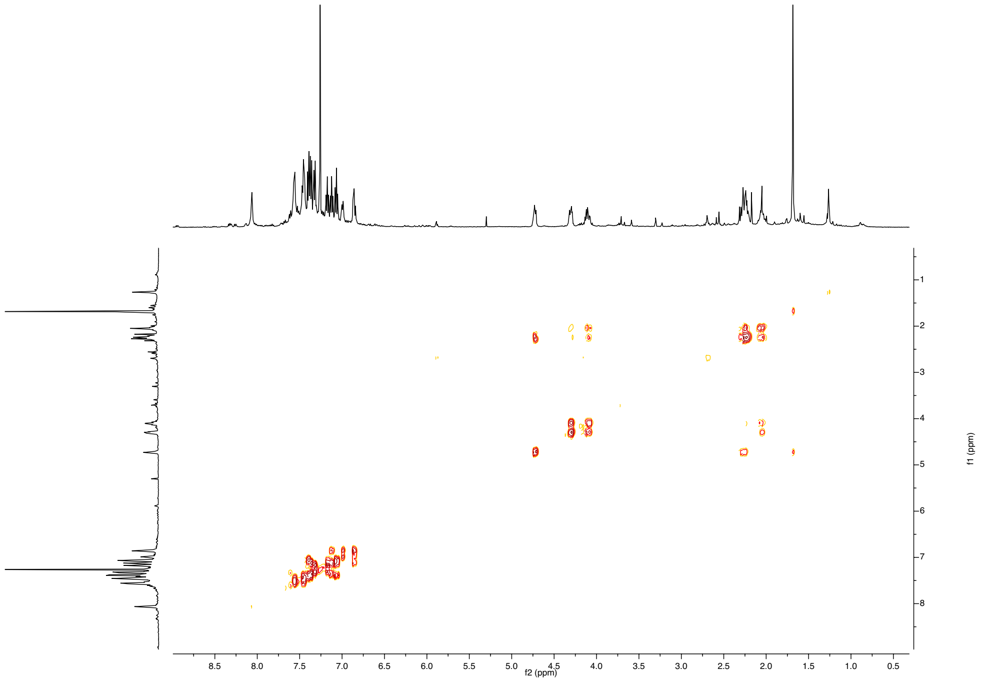

NOESY

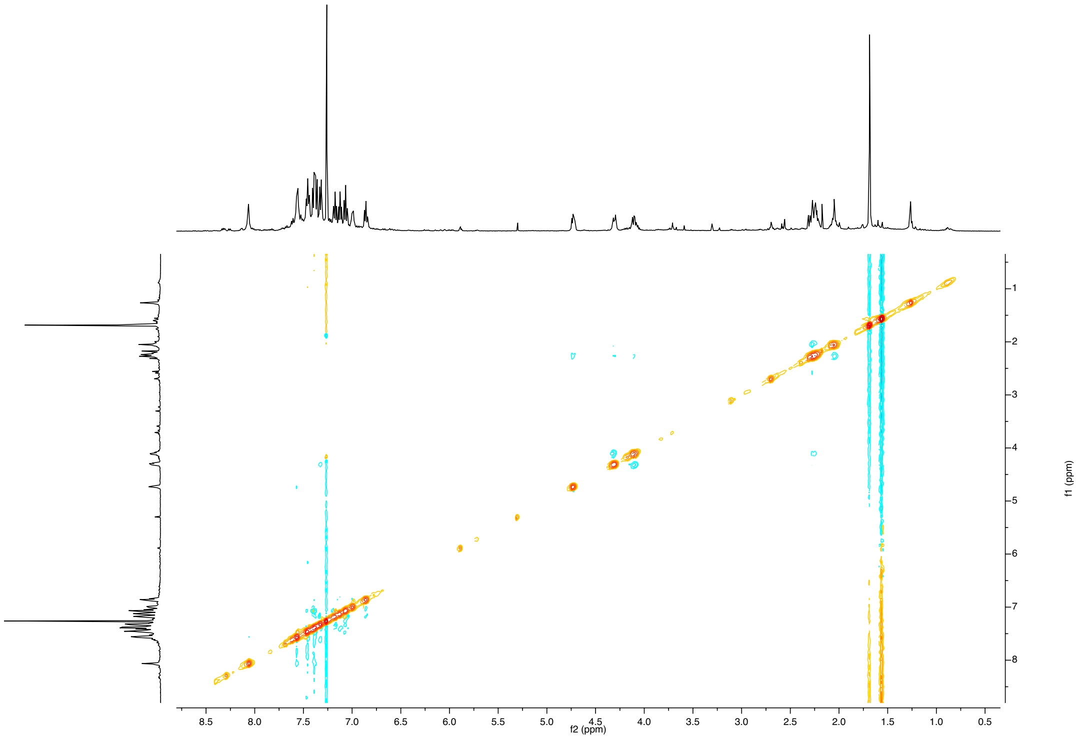

2,3'-BIM 3ai

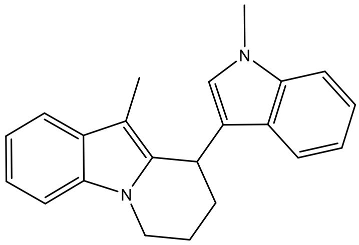

^{1}H NMR (500 MHz, $CDCl_3$, 25°C, TMS)

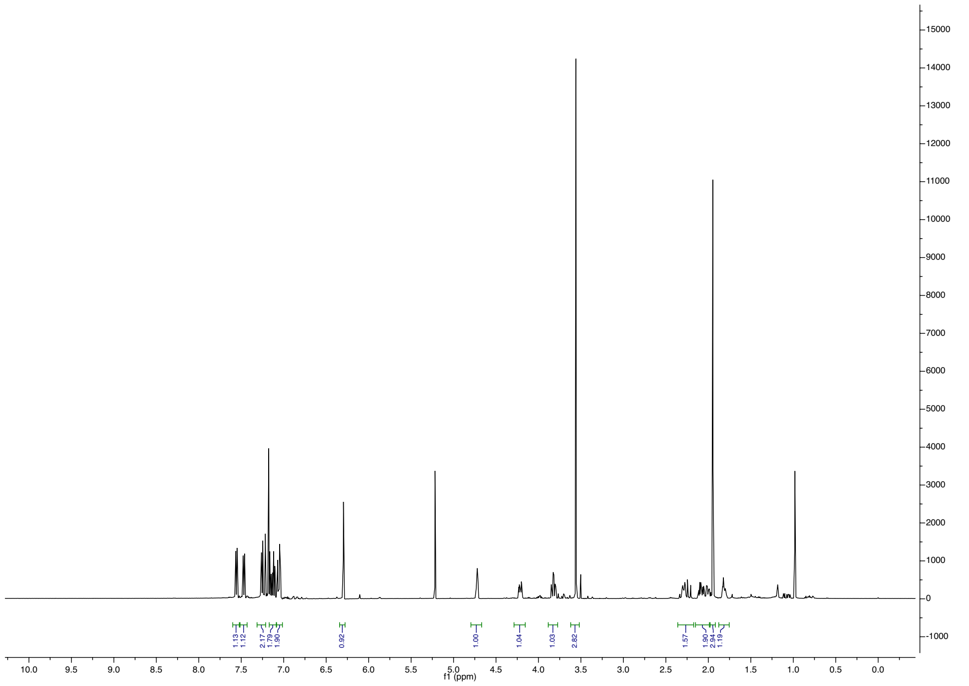

^{13}C NMR (126 MHz, $CDCl_3$, 25°C)

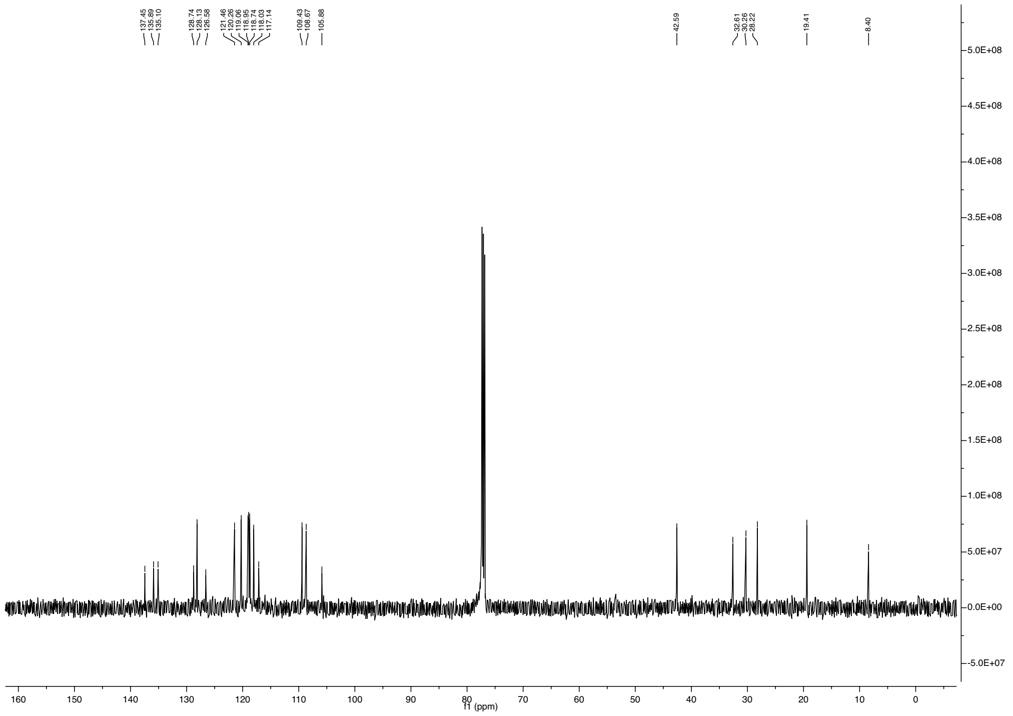

2D gCOSY ($CDCl_3$)

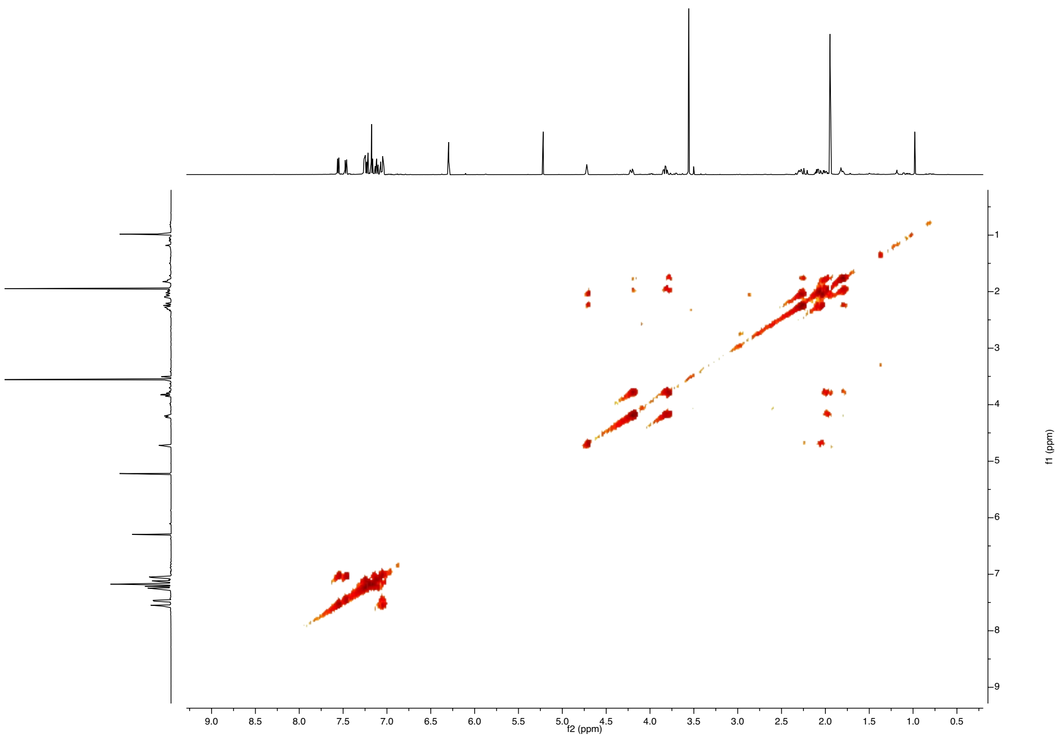

2D HSQC ($CDCl_3$)

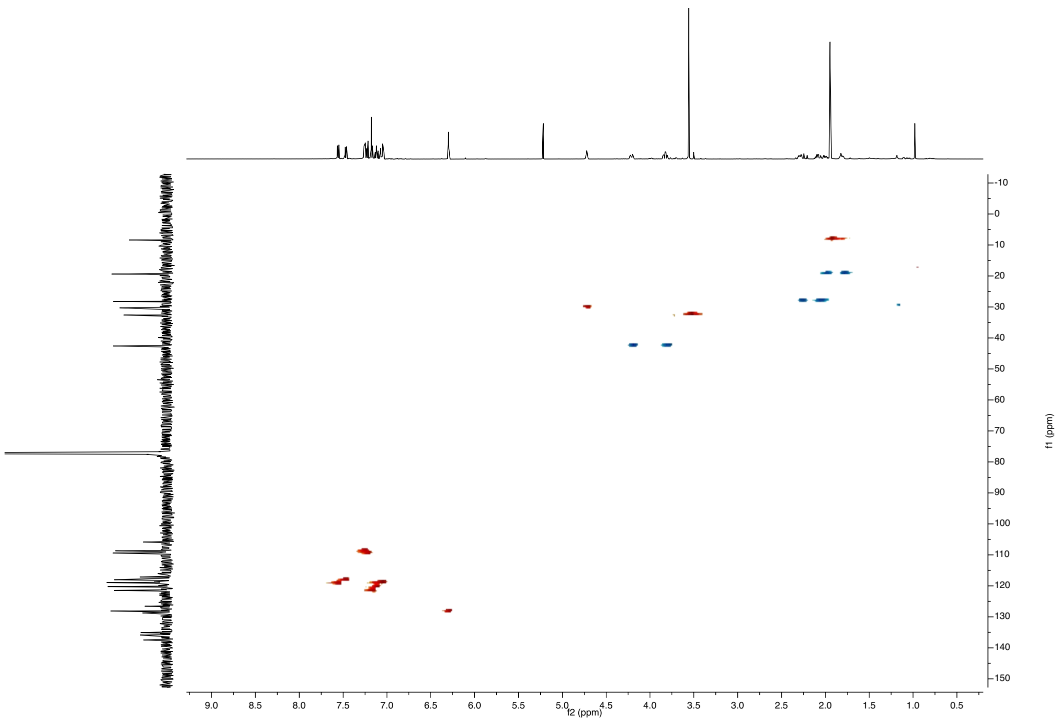

2,3’-BIM 3aj

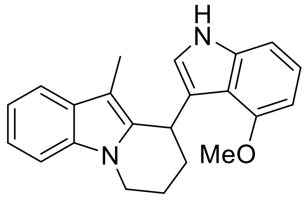

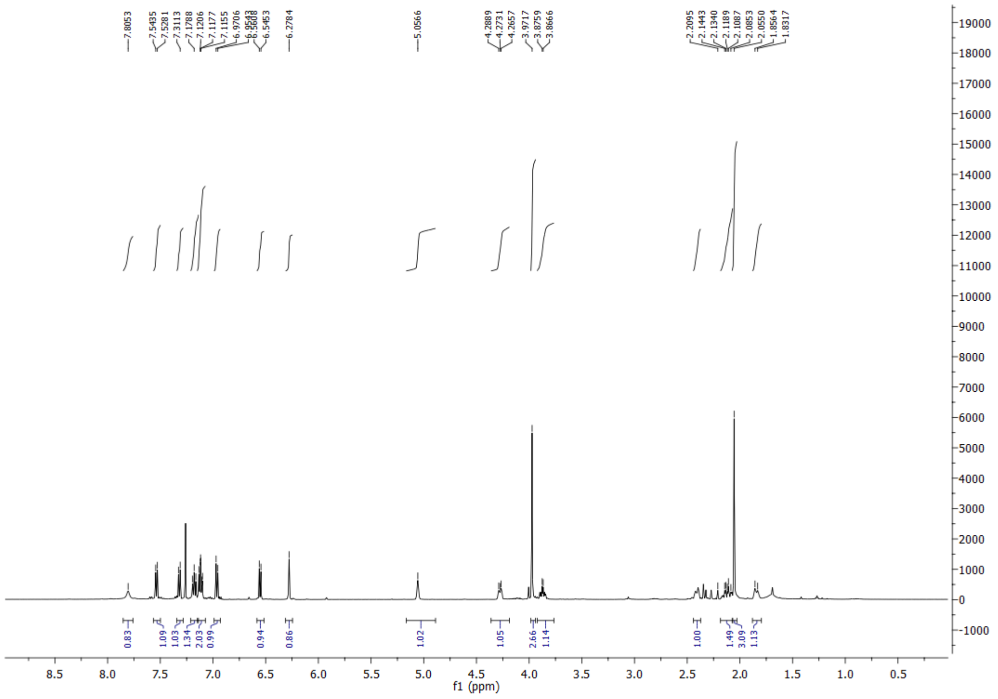

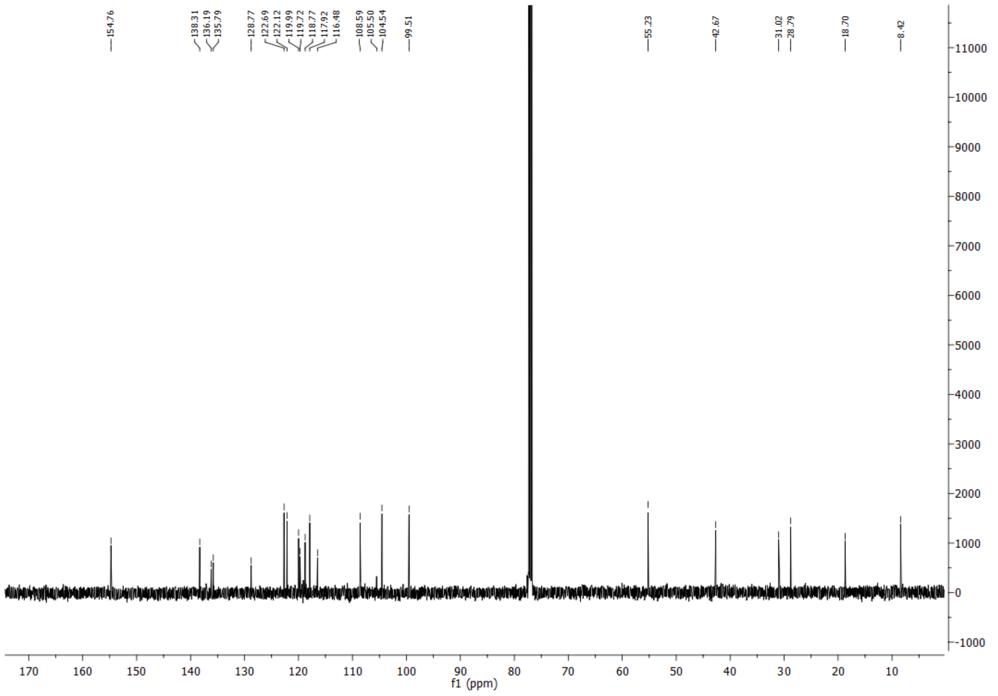

2,3'-BIM 3ak

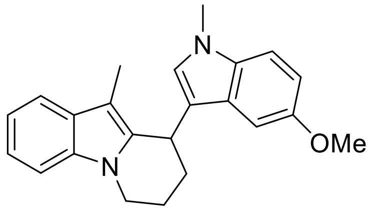

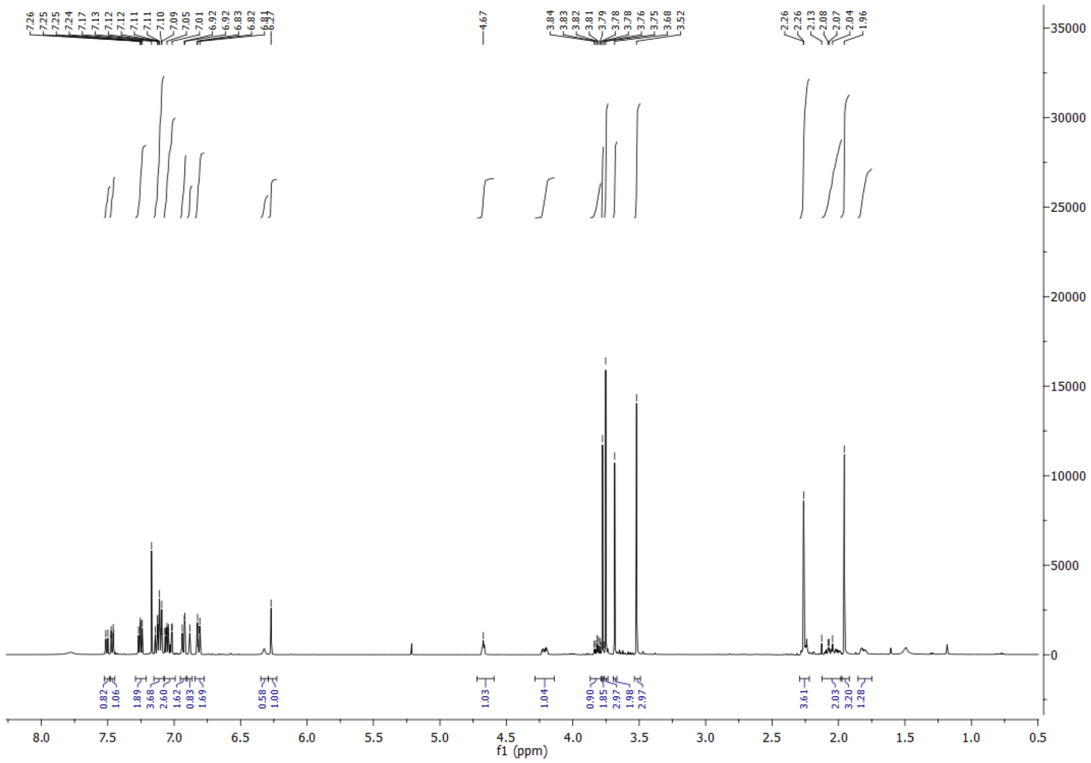

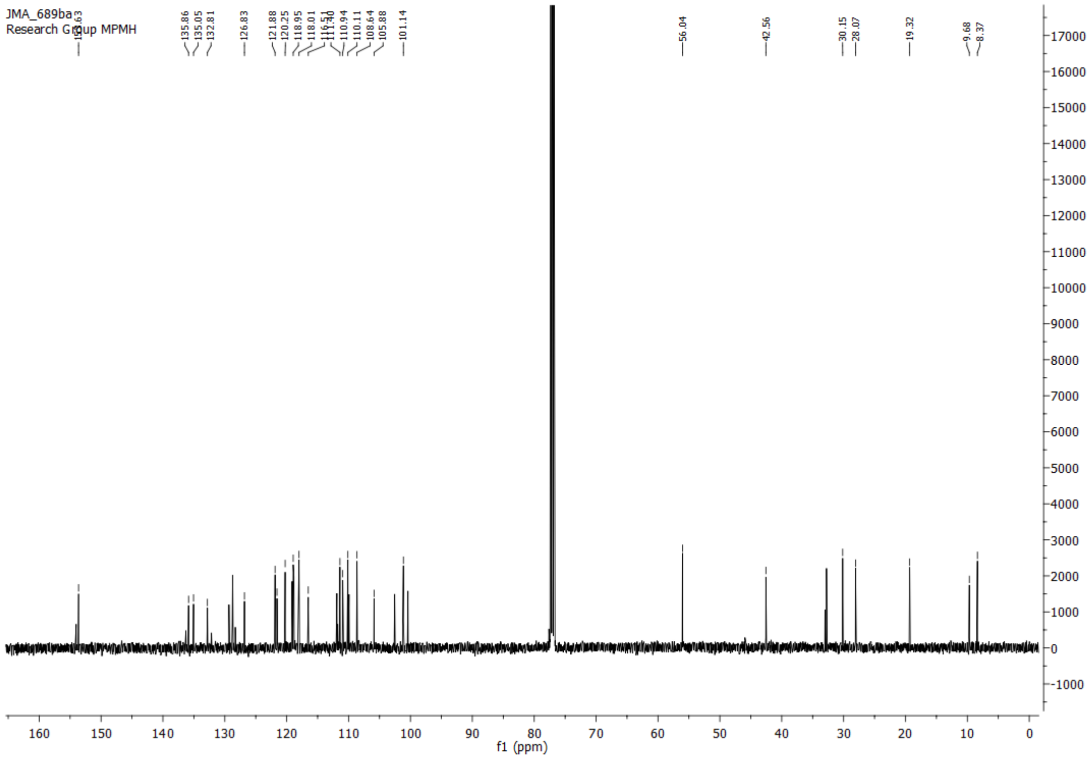

2,3'-BIM 3al

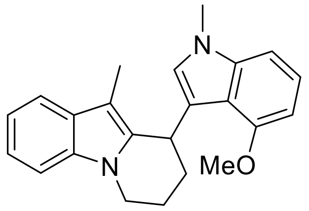

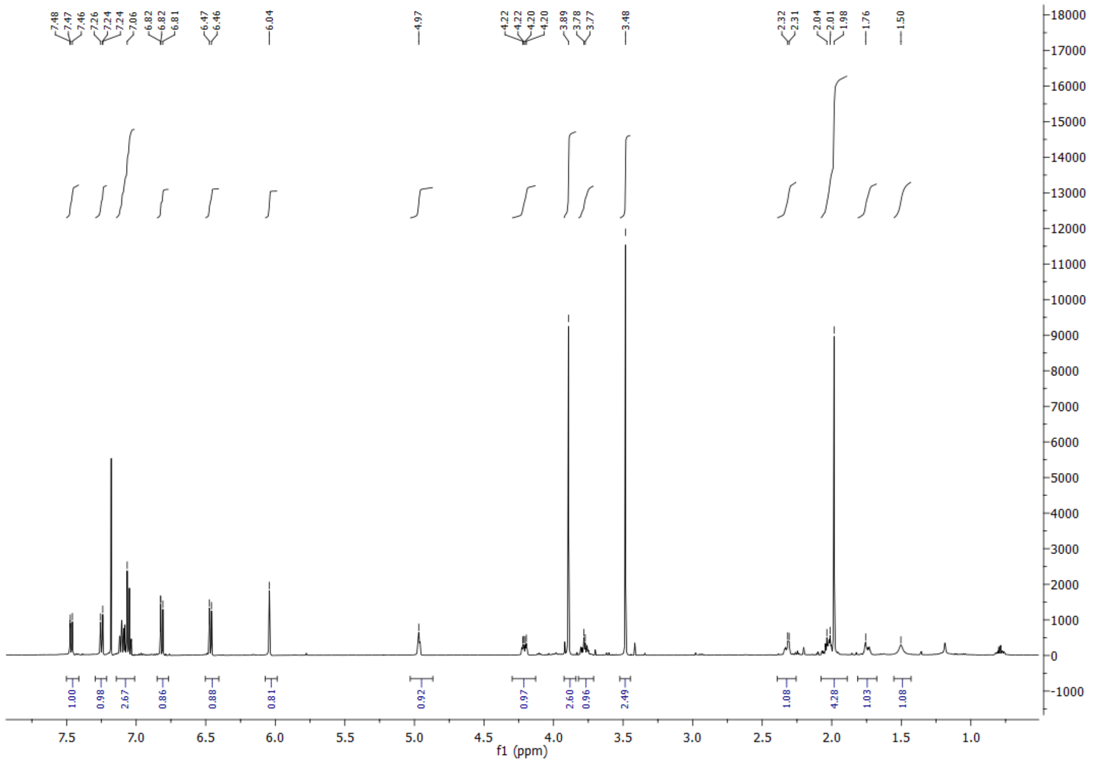

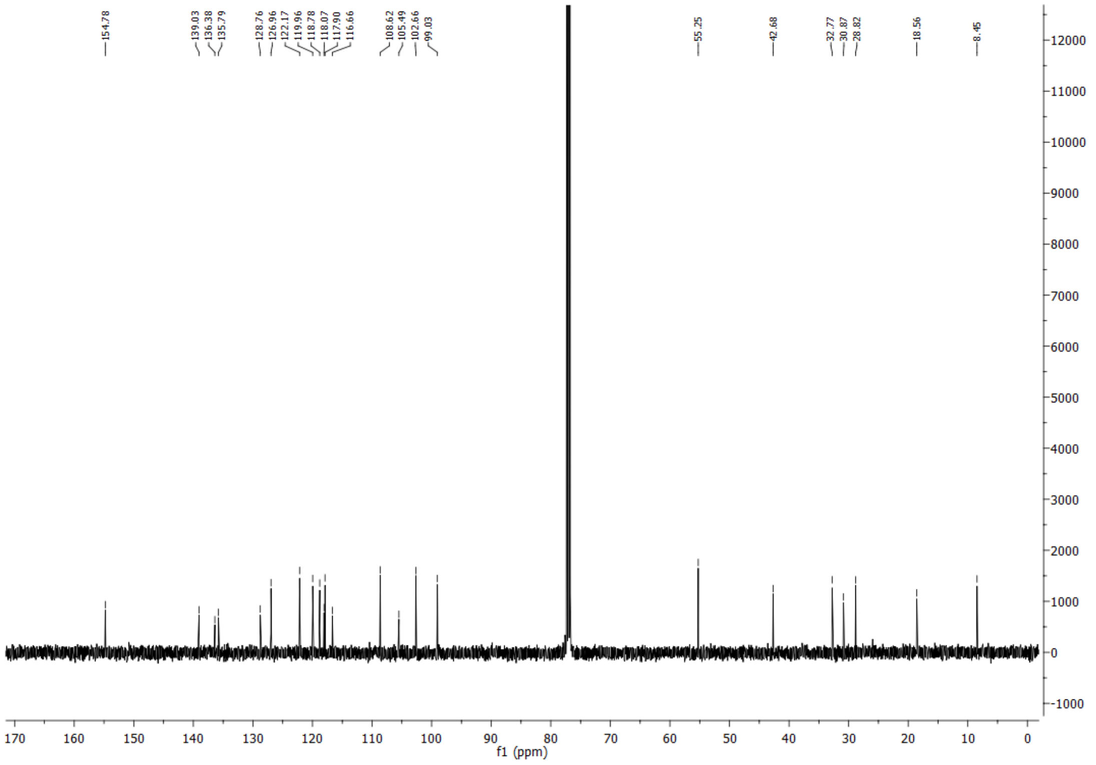

2,3'-BIM 3am

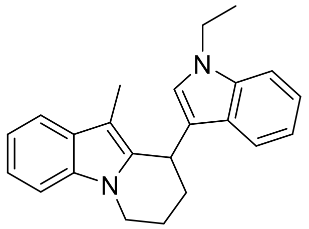

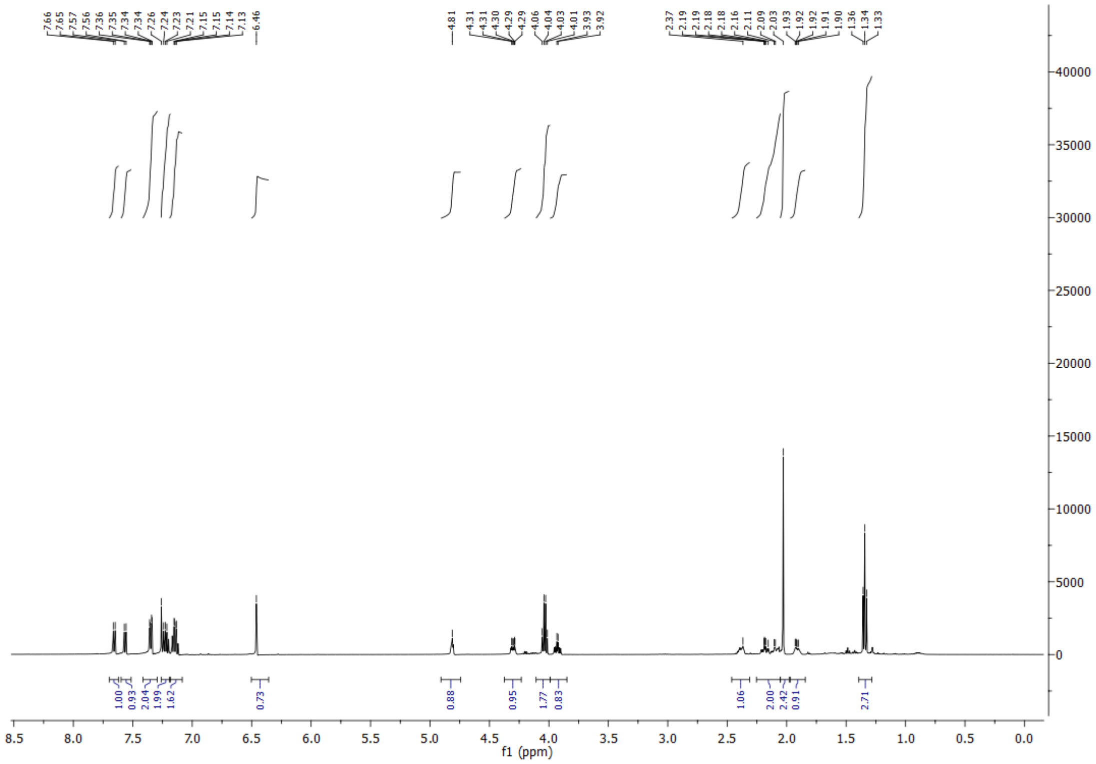

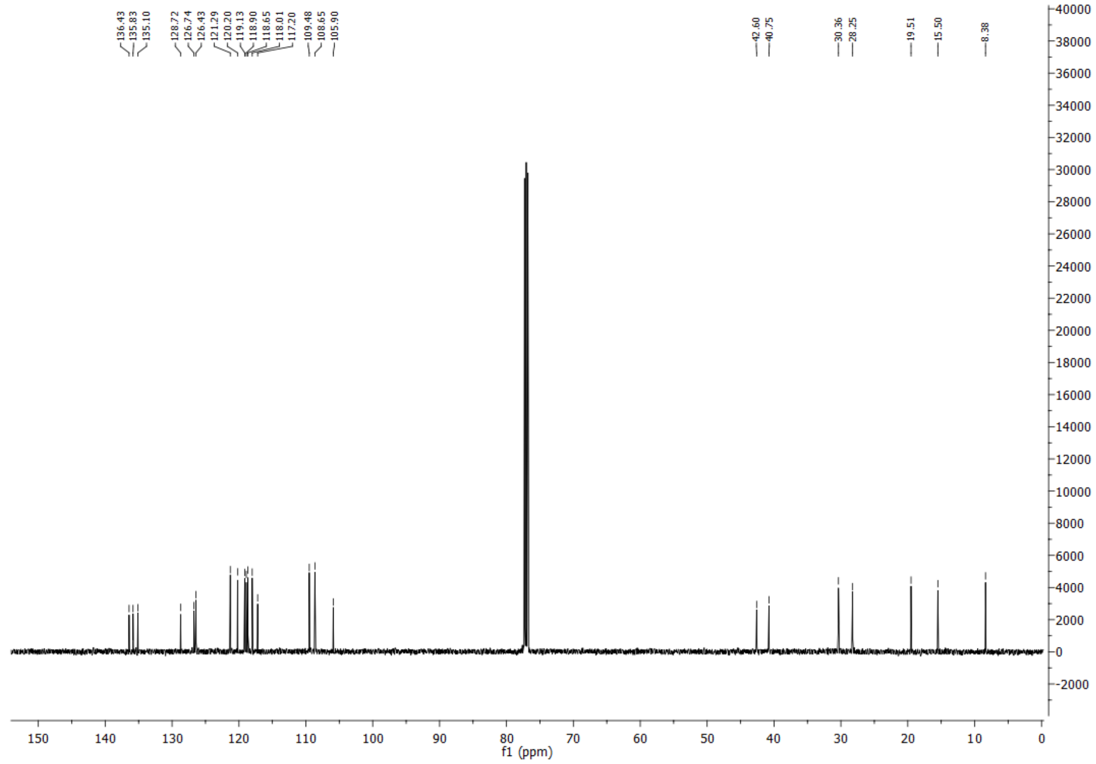

2,3'-BIM 3bb

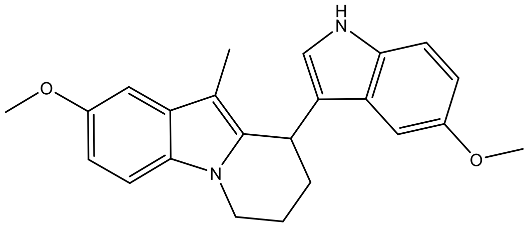

^{1}H NMR (500 MHz, $CDCl_3$, 25°C, TMS)

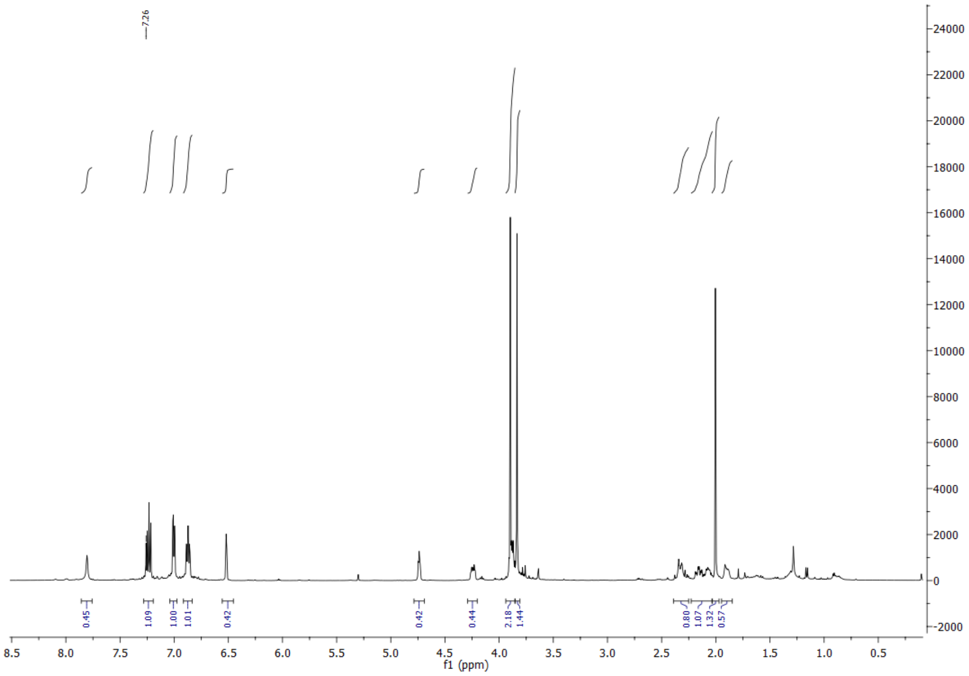

^{13}C NMR (126 MHz, $CDCl_3$, 25°C)

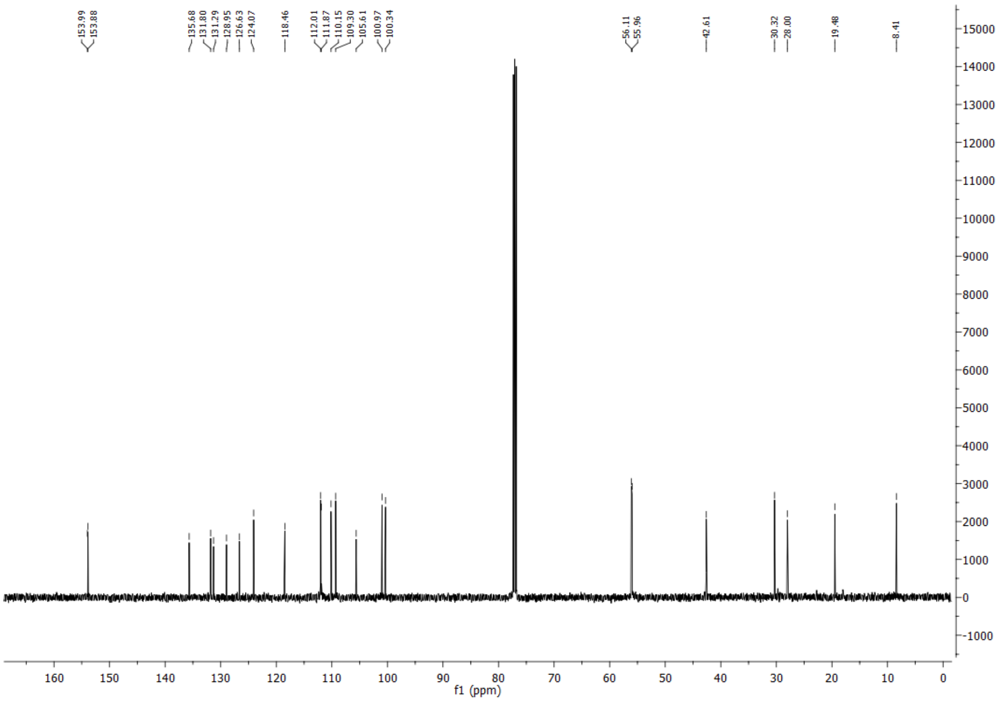

2D gCOSY ($CDCl_3$)

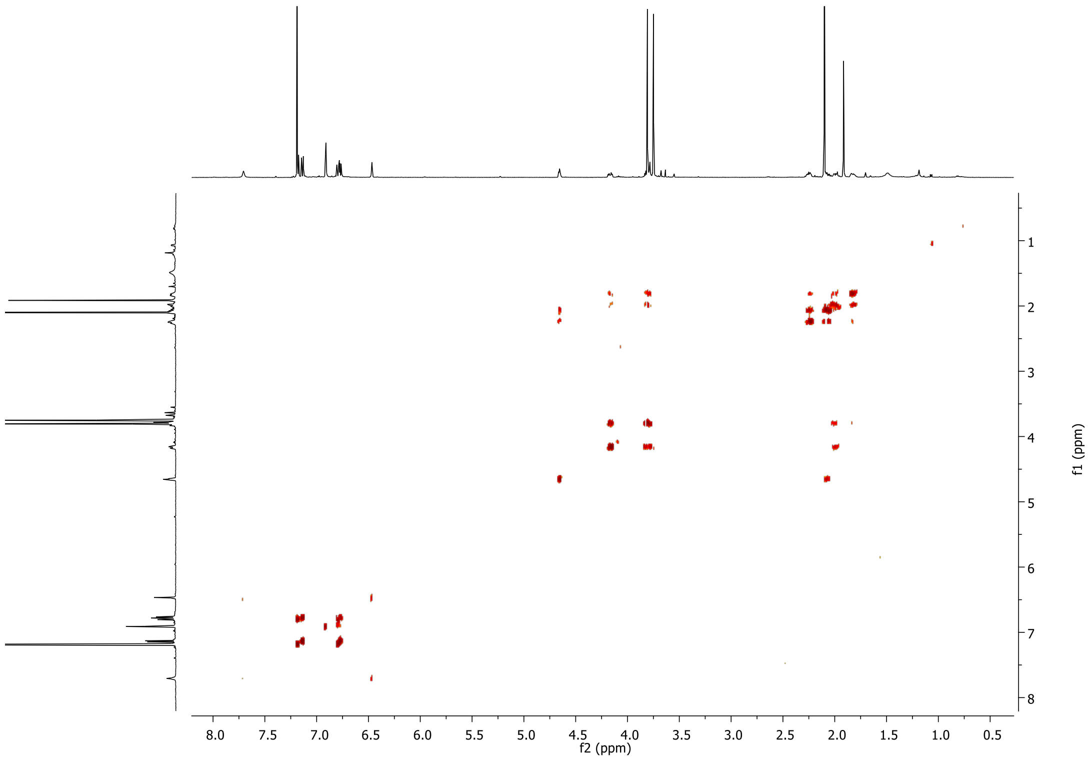

2D HSQC ($CDCl_3$)

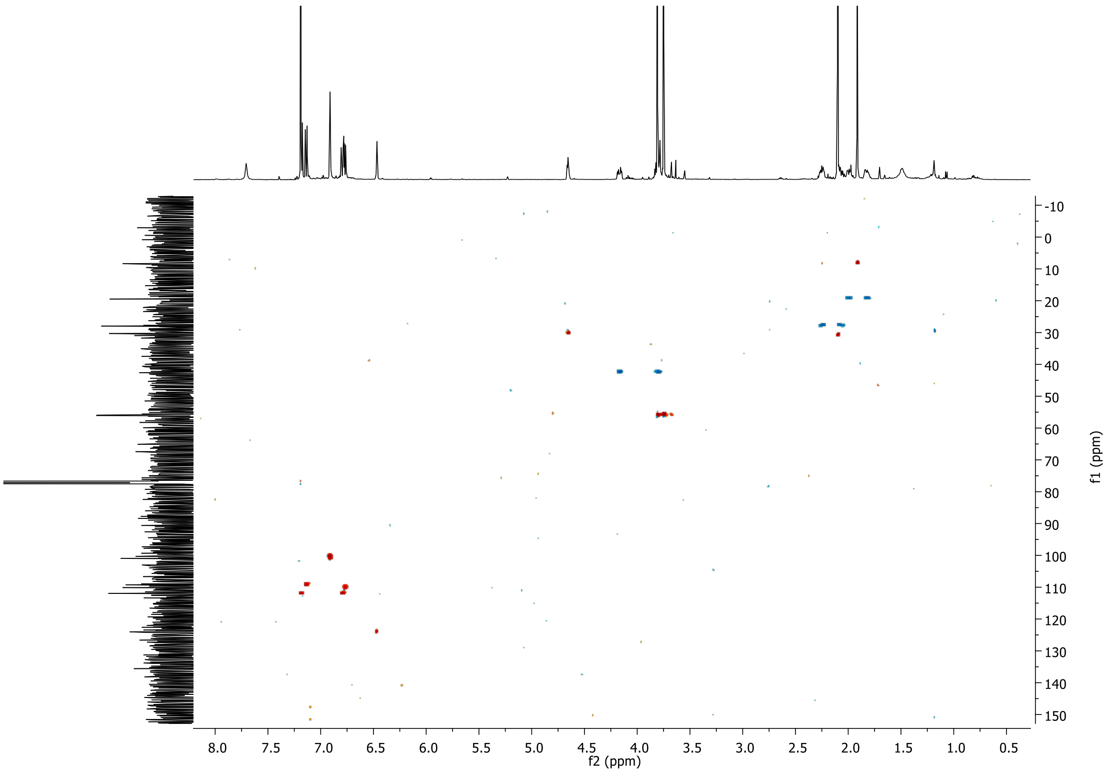

2,3'-BIM 3ga

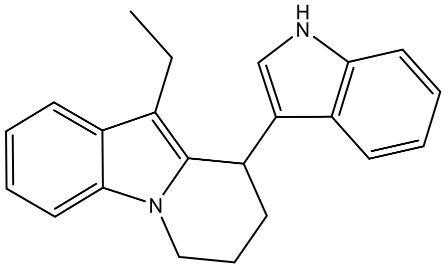

^{1}H NMR (500 MHz, $CDCl_3$, 25°C, TMS)

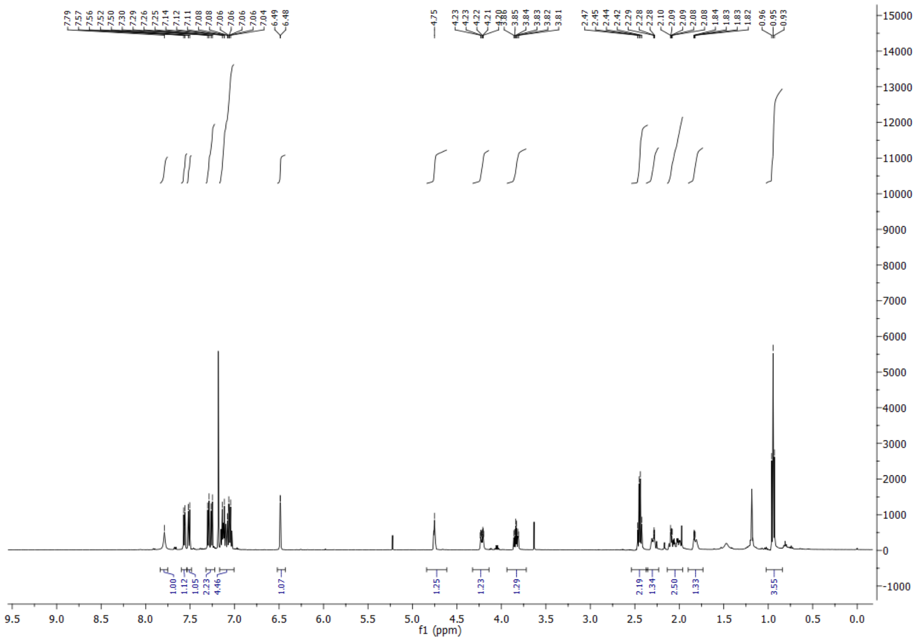

^{13}C NMR (126 MHz, $CDCl_3$, 25°C)

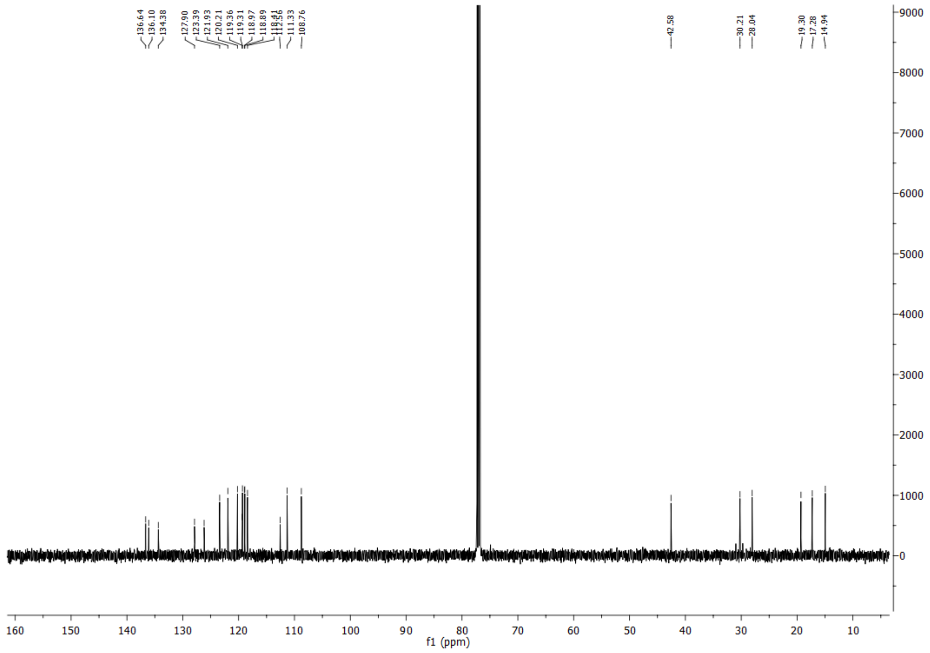

2D HSQC ($CDCl_3$)

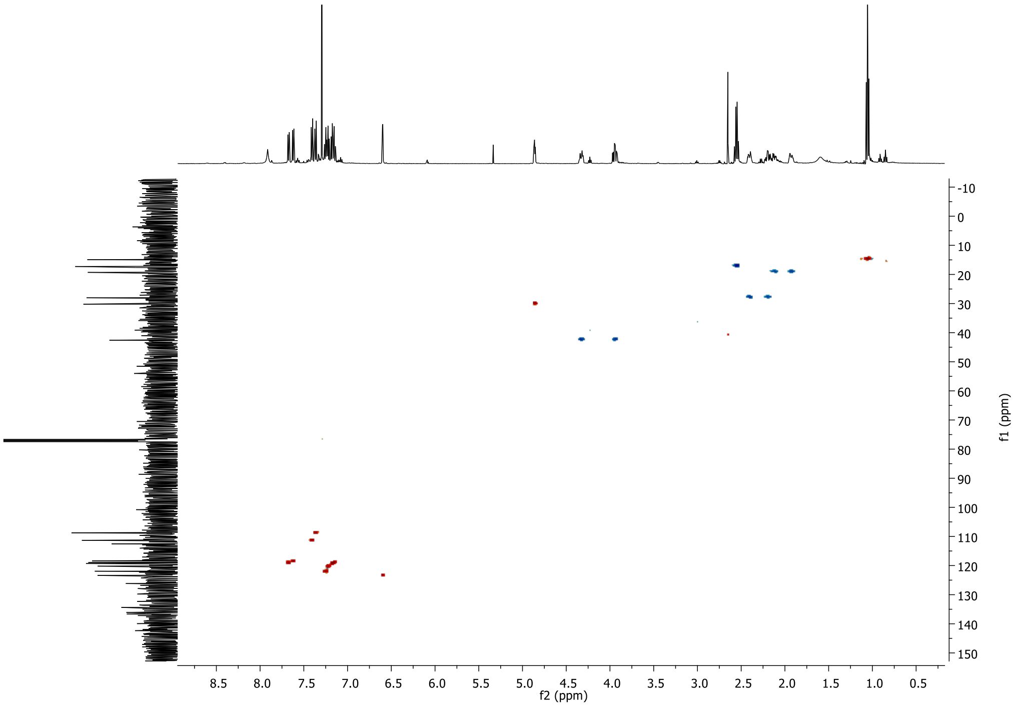

Cycle 4h’

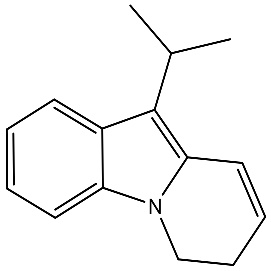

^{1}H NMR (500 MHz, $CDCl_3$, 25°C, TMS)

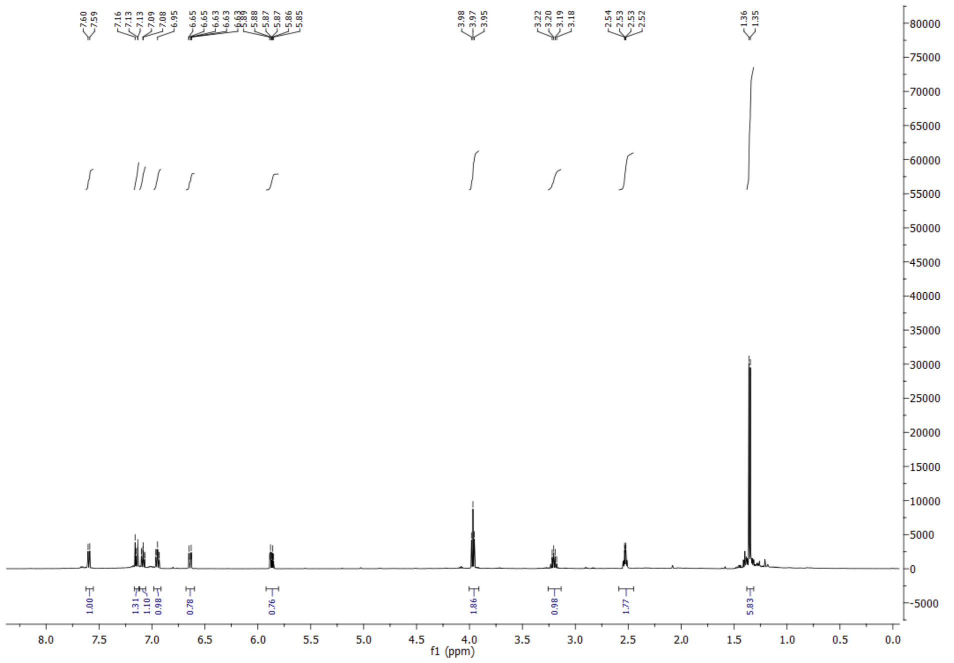

^{13}C NMR (126 MHz, $CDCl_3$, 25°C)

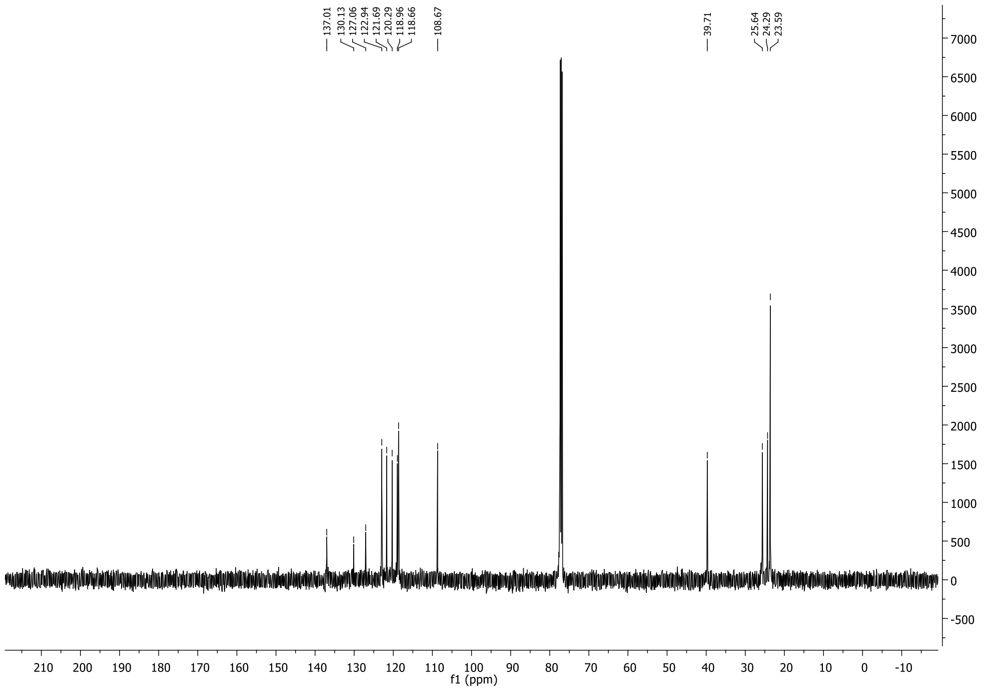

2D HSQC ($CDCl_3$)

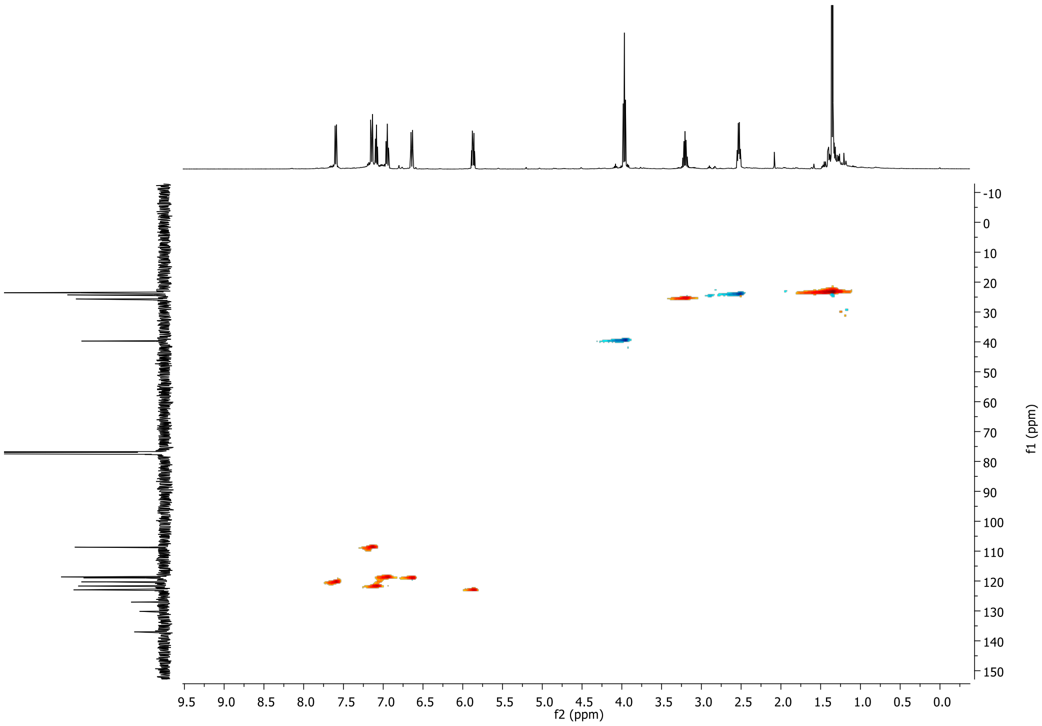

2,3'-BIM 3ha

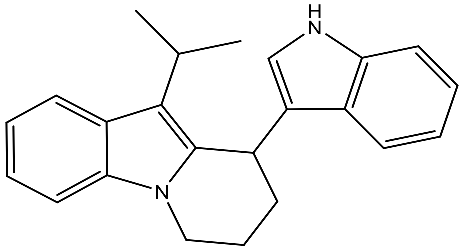

^{1}H NMR (500 MHz, $CDCl_3$, 25°C, TMS)

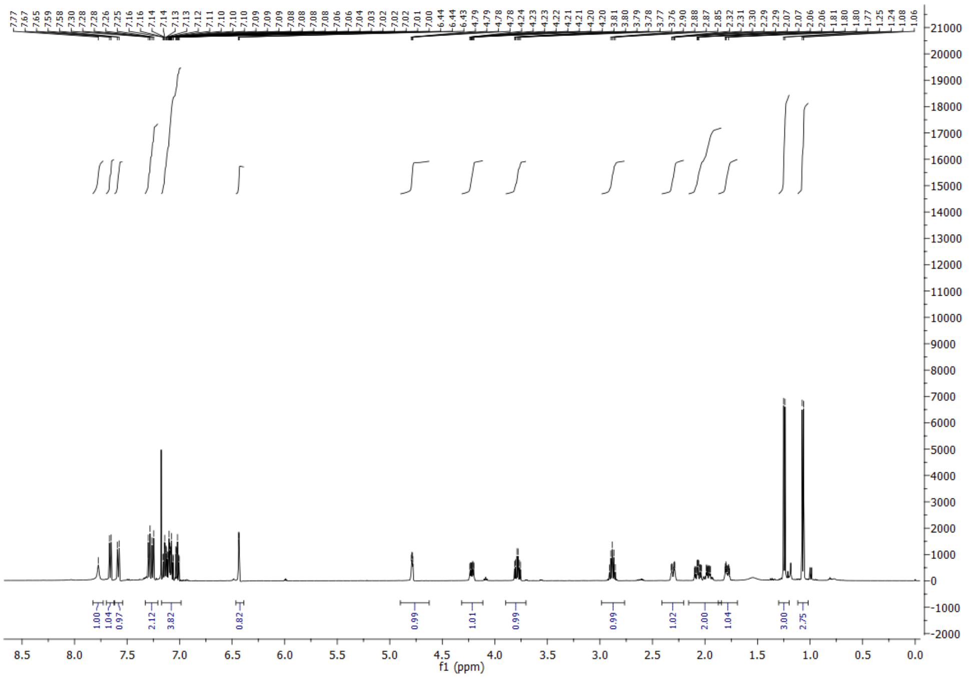

^{13}C NMR (126 MHz, $CDCl_3$, 25°C)

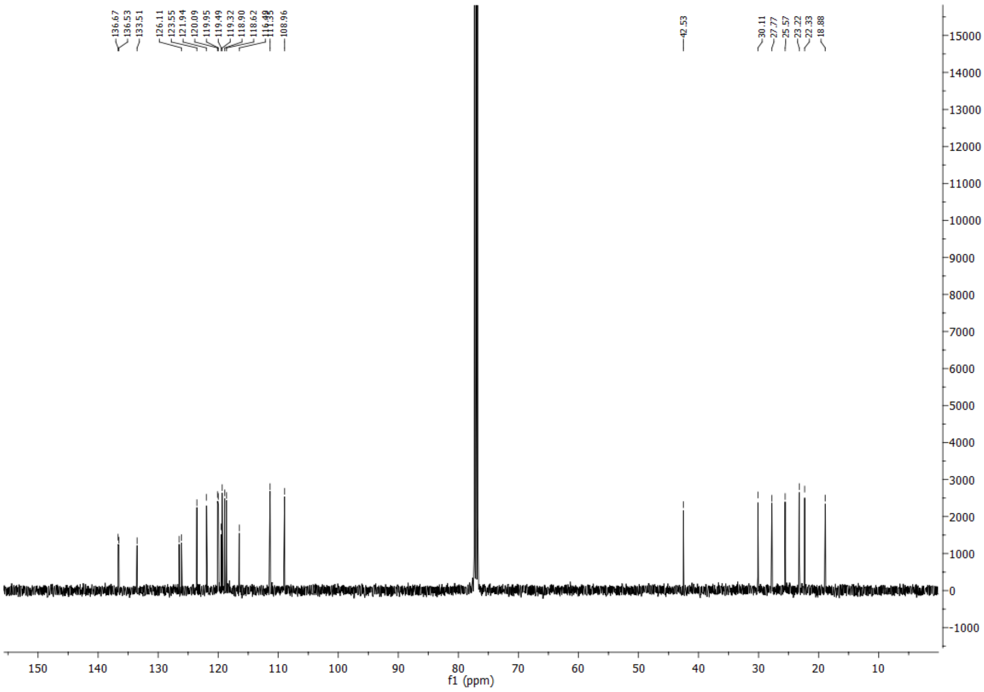

2D HSQC ($CDCl_3$)

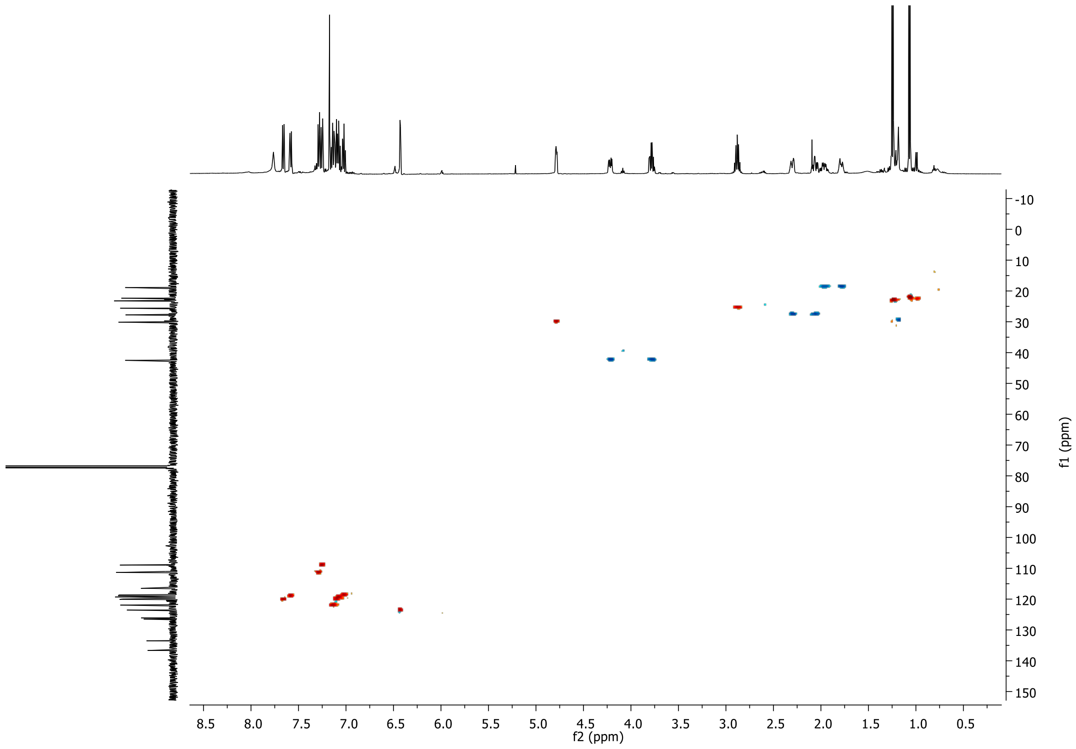

Cycle 4i’

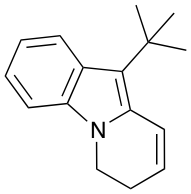

^{1}H NMR (500 MHz, $CDCl_3$, 25°C, TMS)

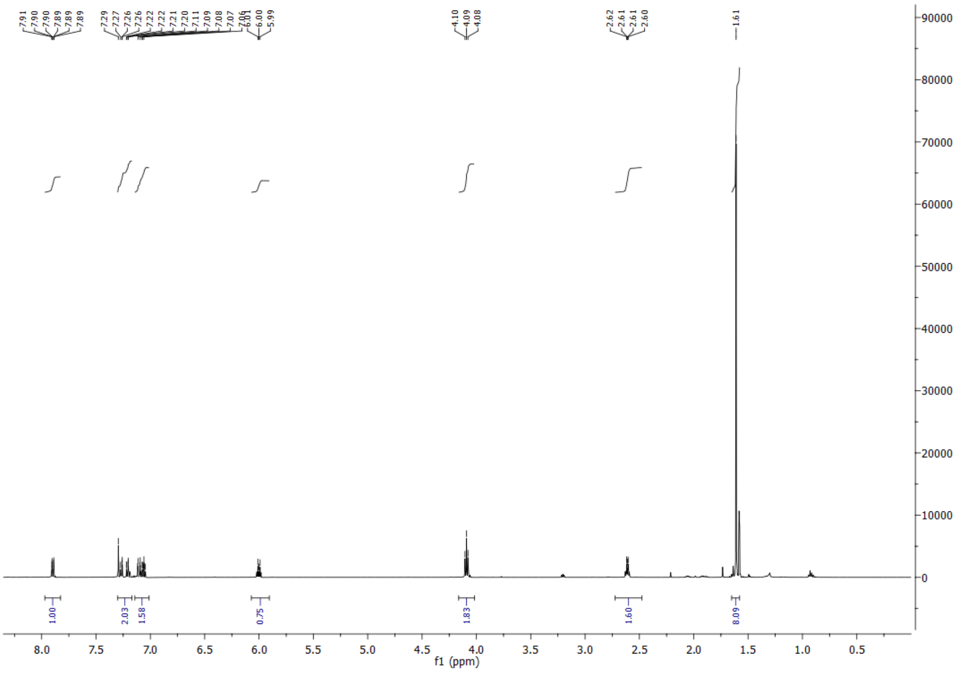

^{13}C NMR (126 MHz, $CDCl_3$, 25°C)

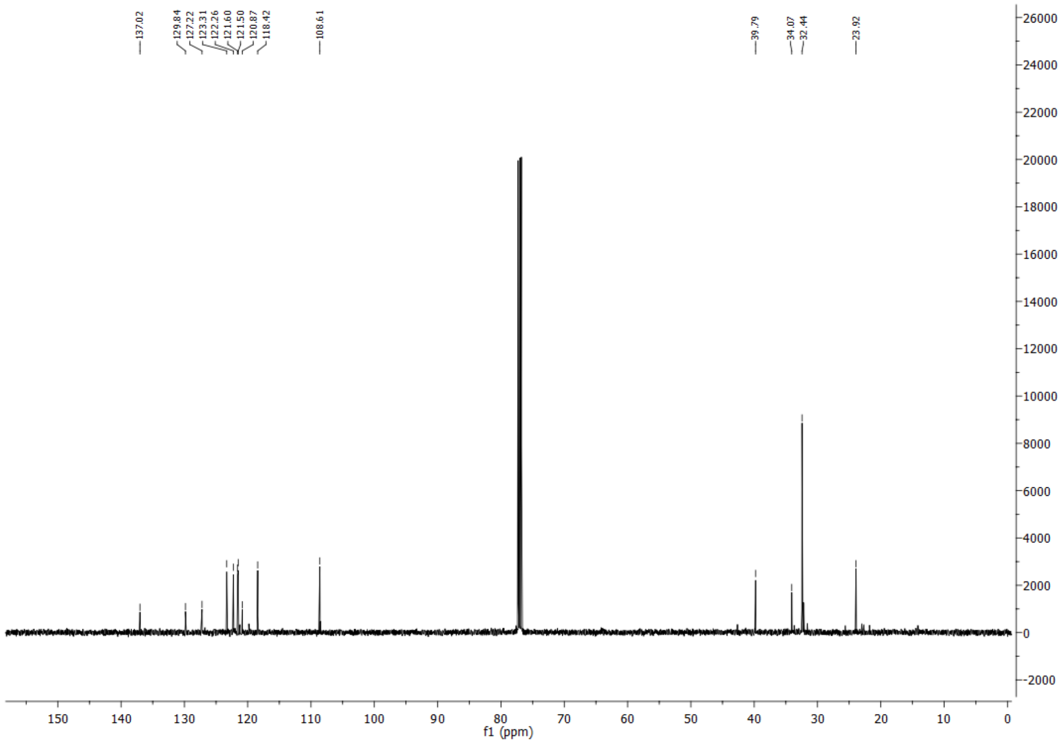

2,3’-BIM 3ia

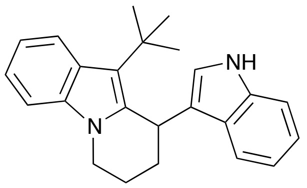

^{1}H NMR (500 MHz, $CDCl_3$, 25°C, TMS)

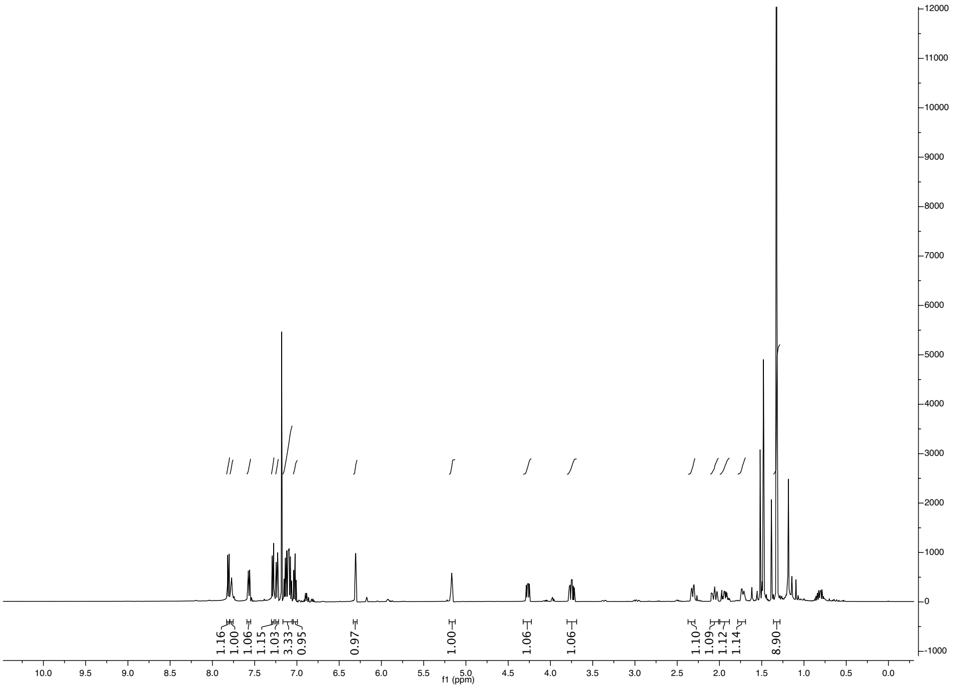

^{13}C NMR (126 MHz, $CDCl_3$, 25°C)

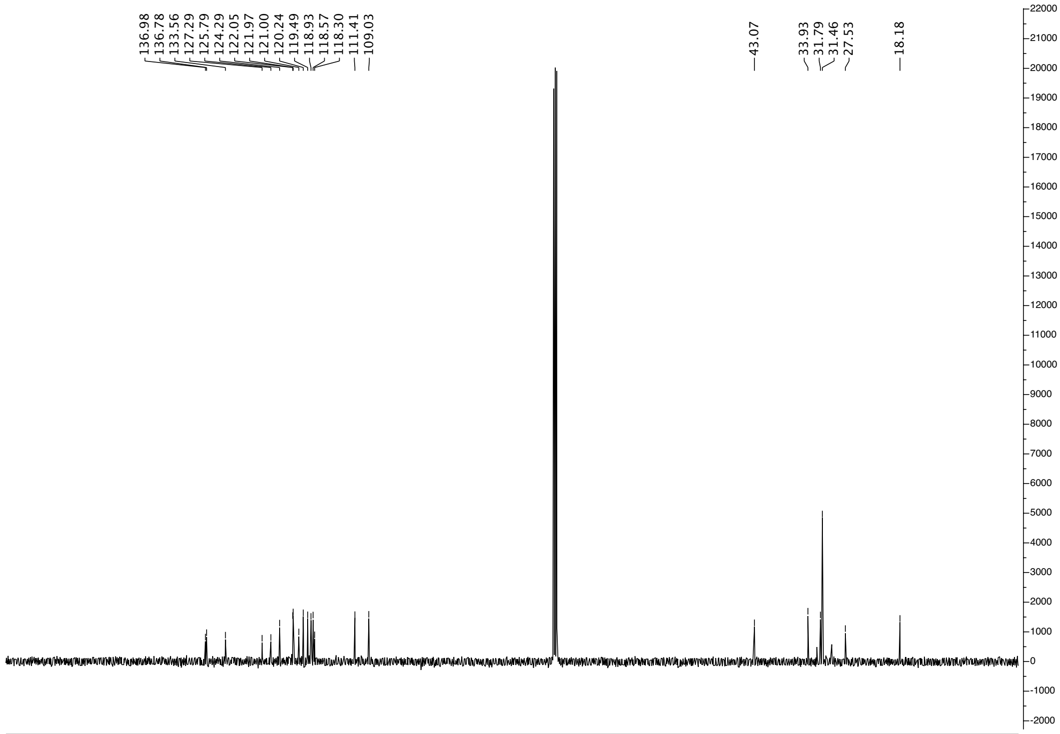

2D HSQC ($CDCl_3$)

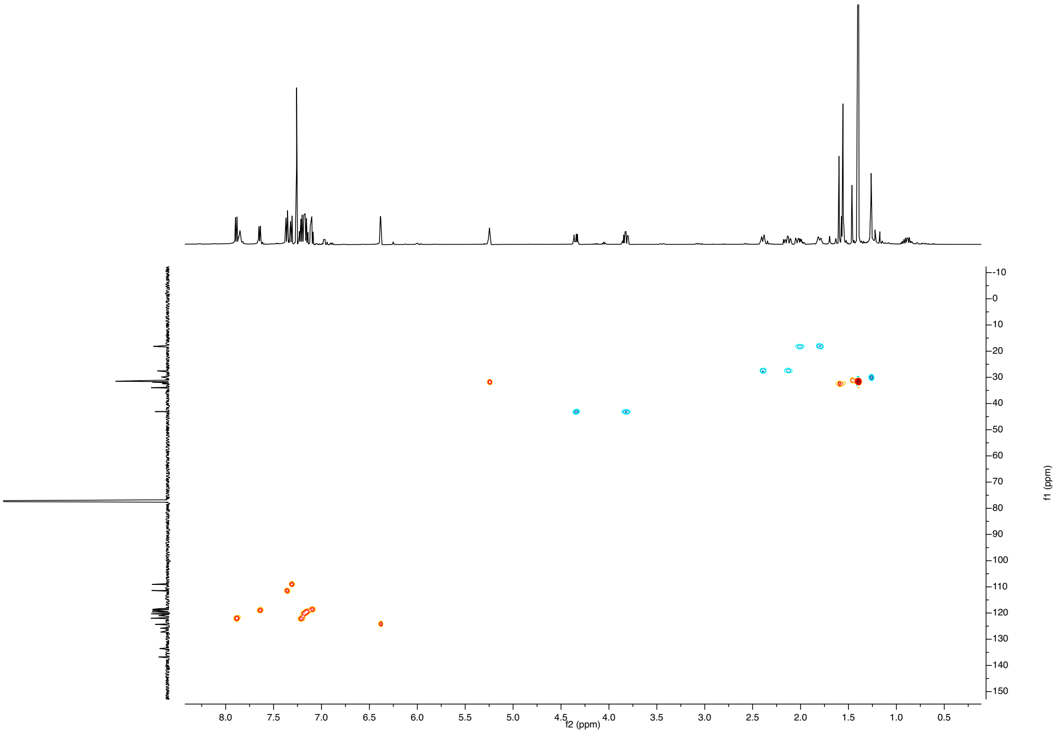

2,3'-BIM 3ja

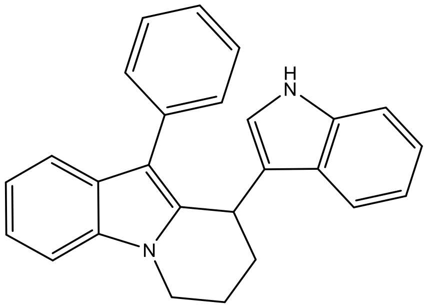

^{1}H NMR (500 MHz, CDCl$_3$, 25°C, TMS)

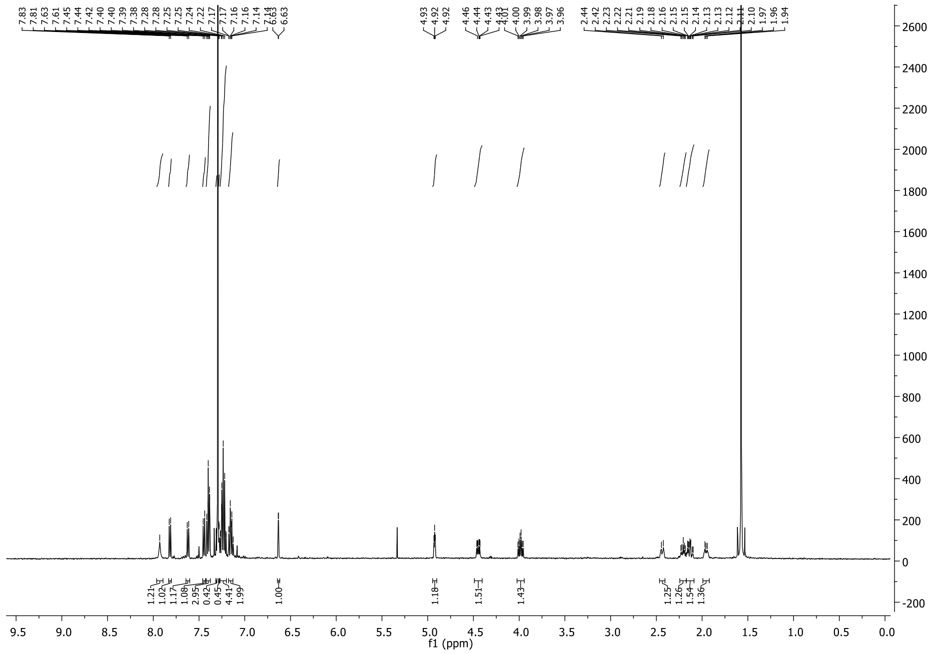

^{13}C NMR (126 MHz, CDCl$_3$, 25°C)

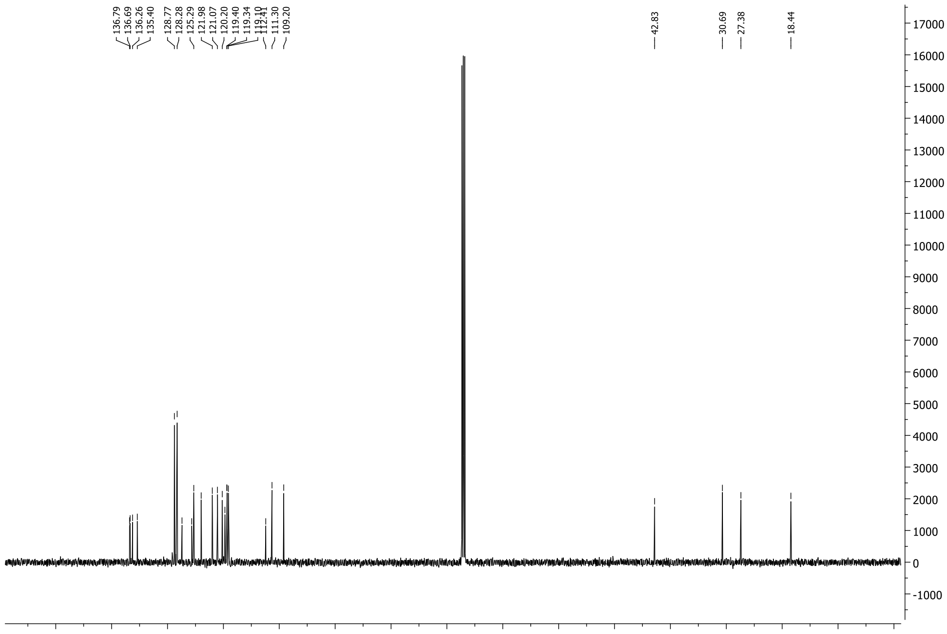

2D HSQC ($CDCl_3$)

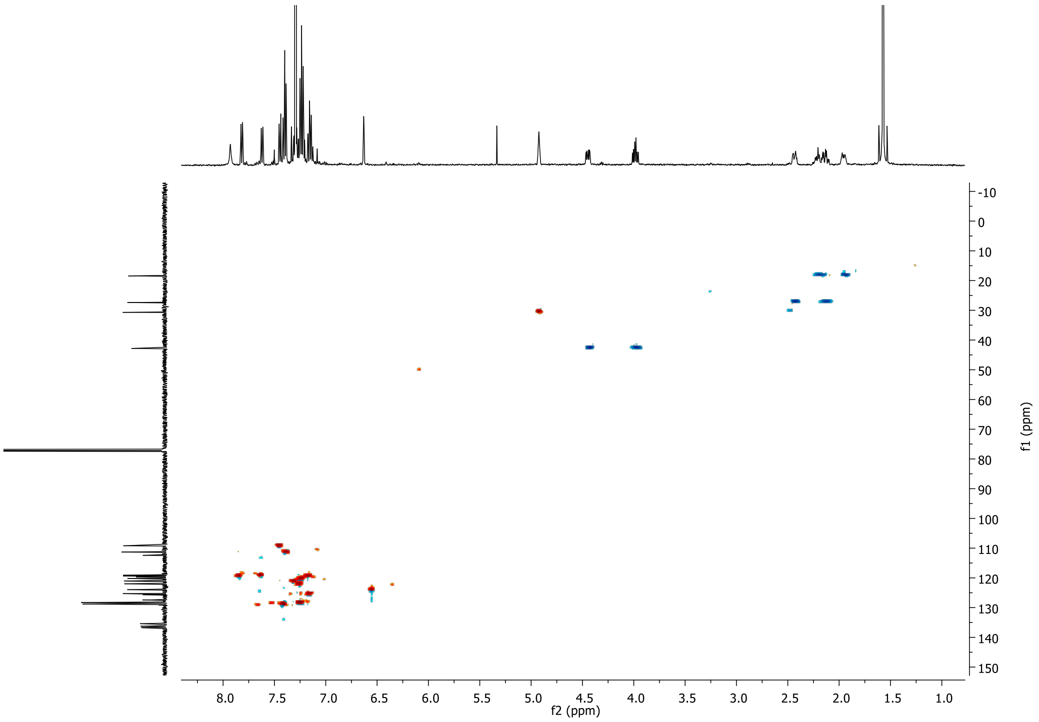

Trisindole 5ka

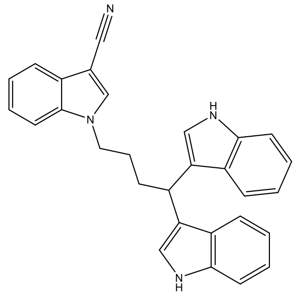

^{1}H NMR (500 MHz, $CDCl_3$, 25°C, TMS)

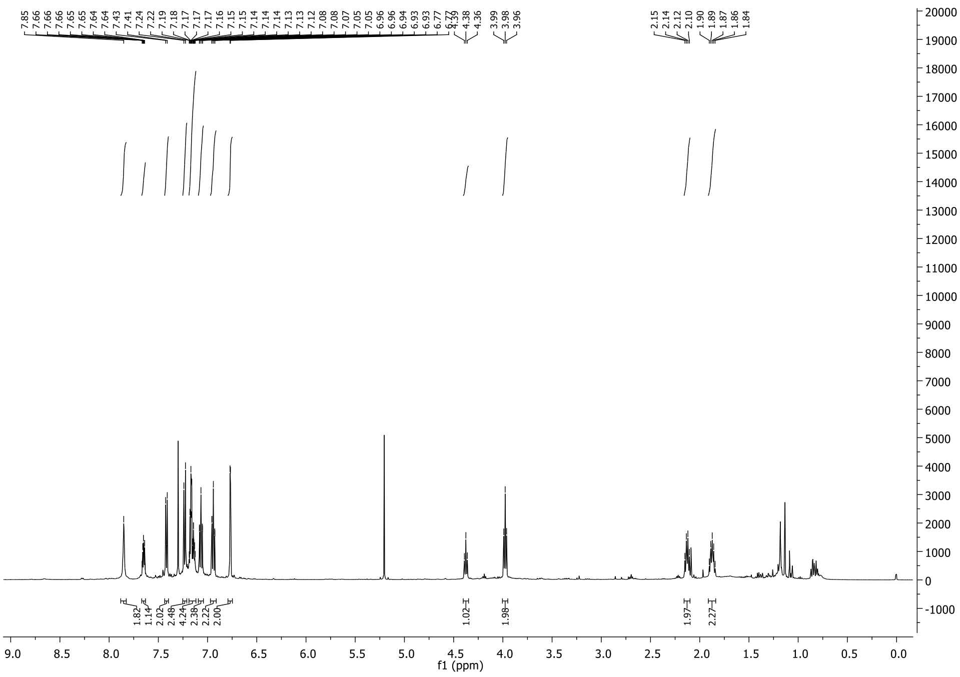

^{13}C NMR (126 MHz, $CDCl_3$, 25°C)

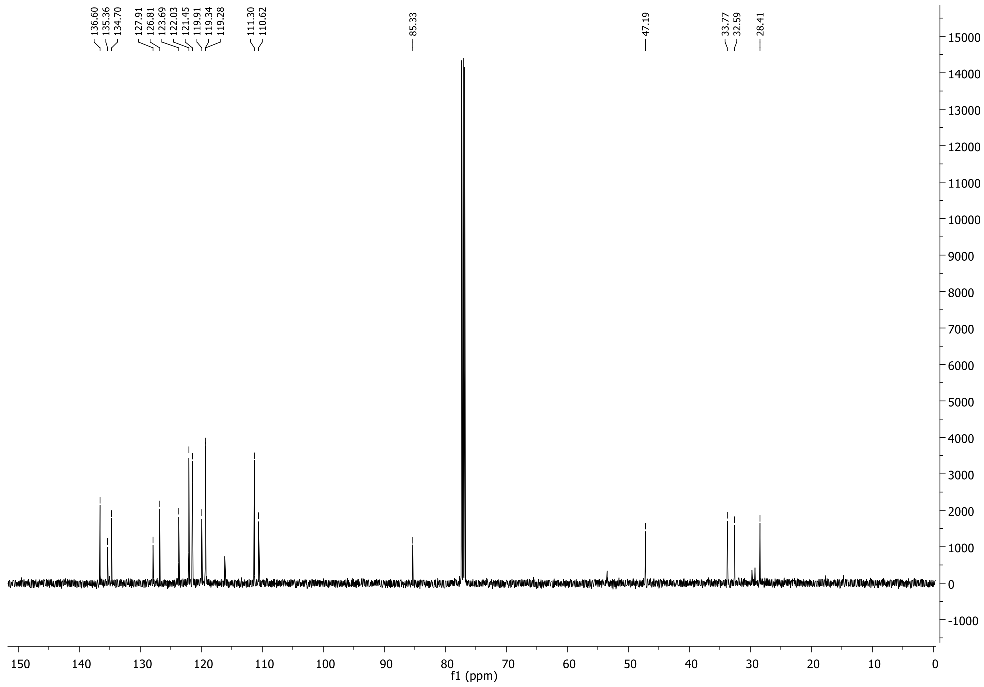

2D HSQC ($CDCl_3$)

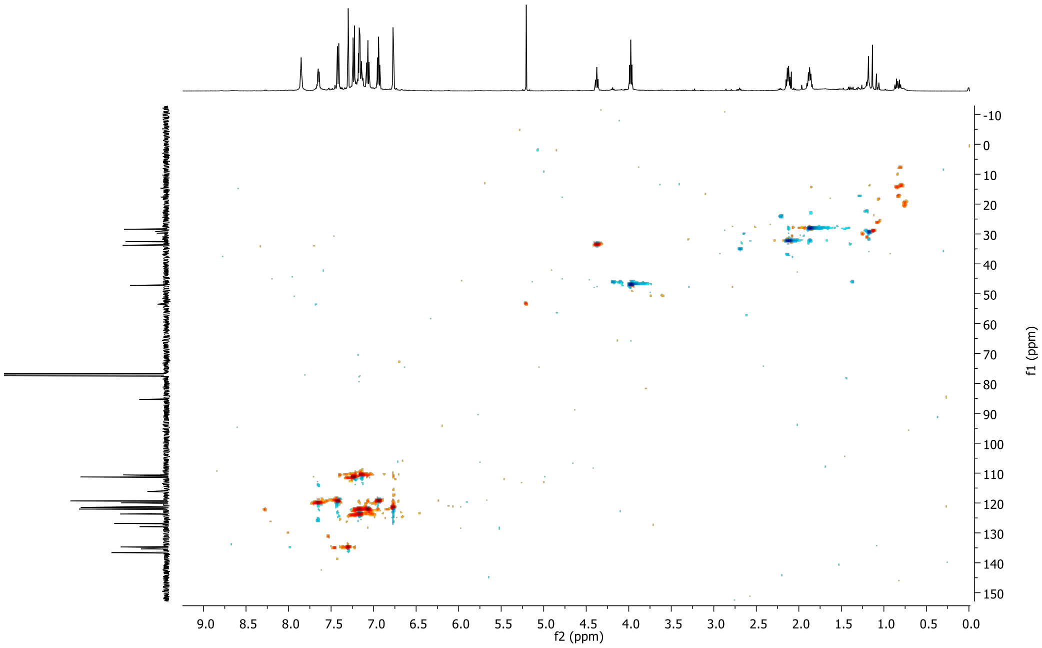

Allyl indole 6ka

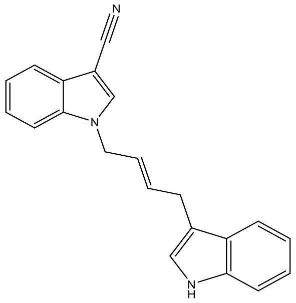

^{1}H NMR (500 MHz, $CDCl_3$, 25°C, TMS)

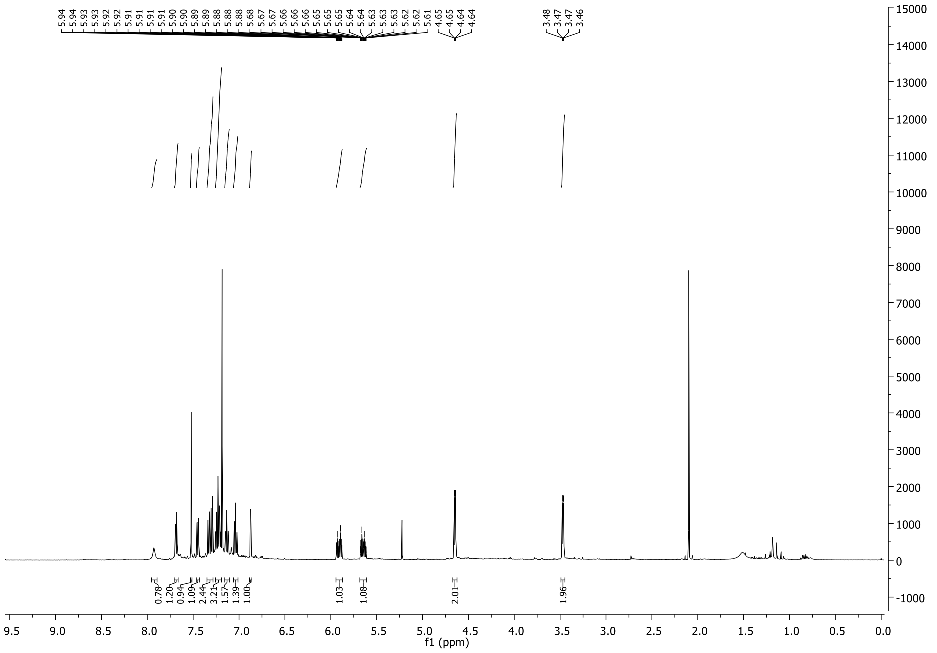

^{13}C NMR (126 MHz, $CDCl_3$, 25°C)

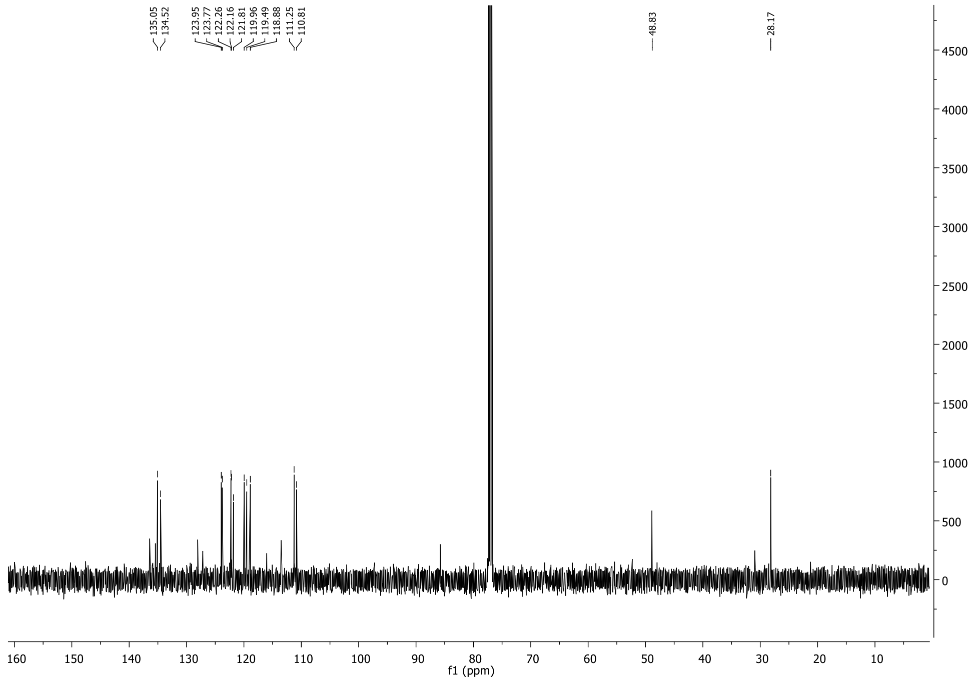

2D HSQC ($CDCl_3$)

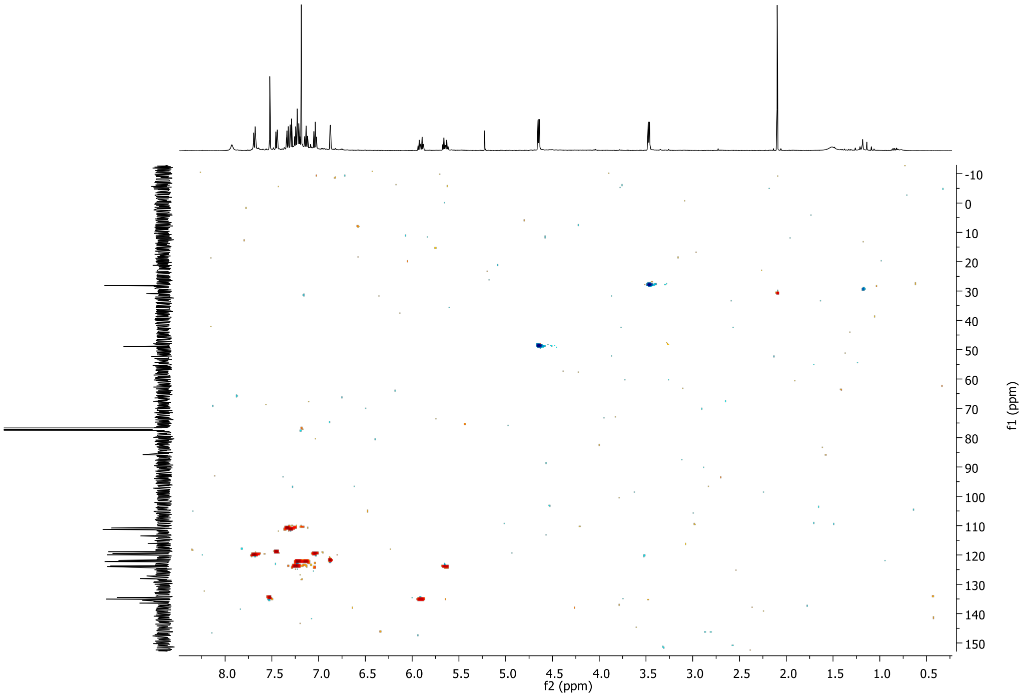

Trisindole 5la

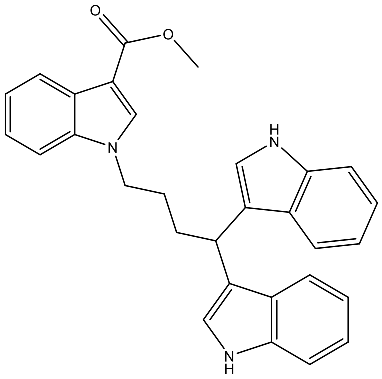

^{1}H NMR (500 MHz, $CDCl_3$, 25°C, TMS)

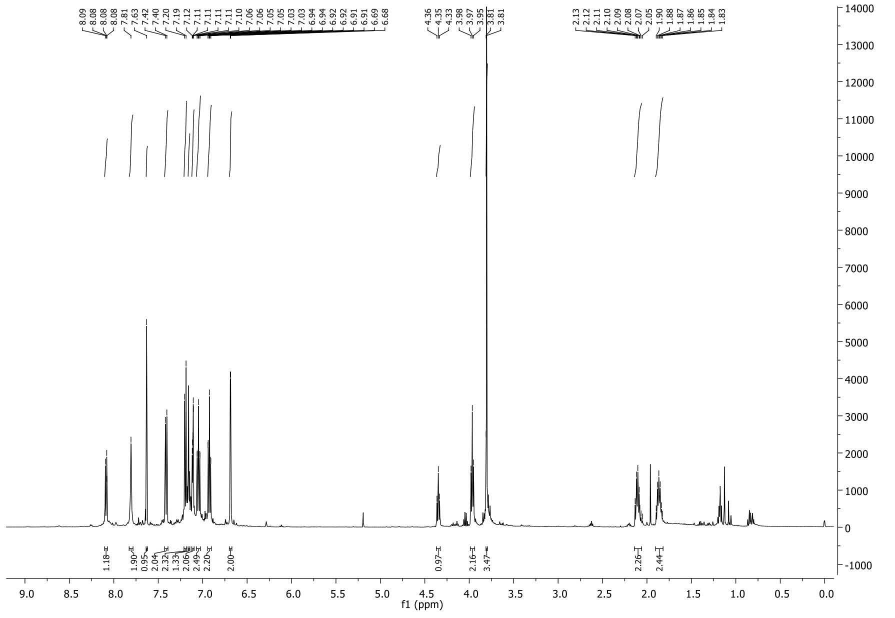

^{13}C NMR (126 MHz, $CDCl_3$, 25°C)

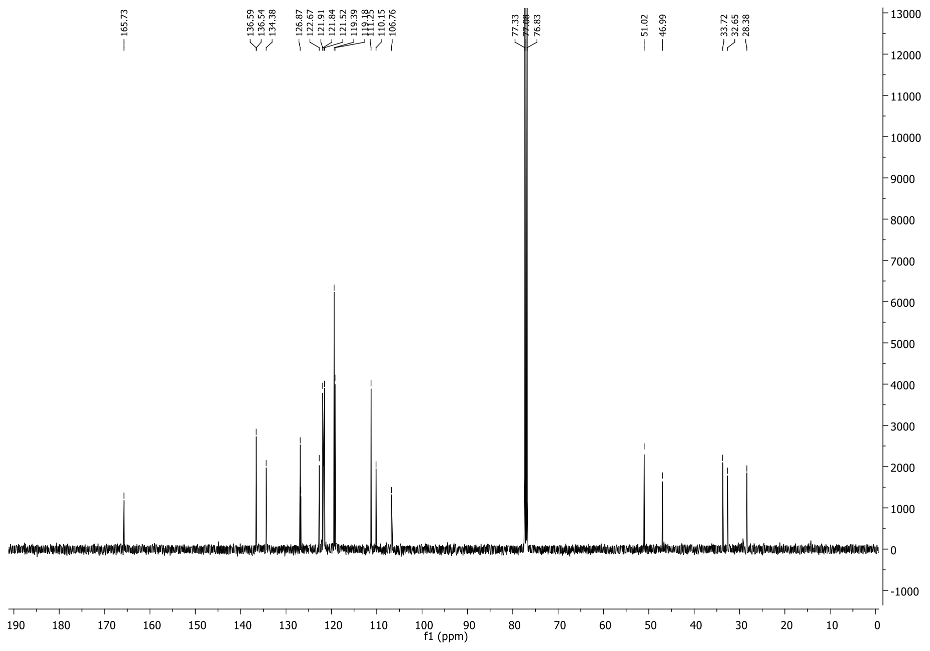

2D HSQC ($CDCl_3$)

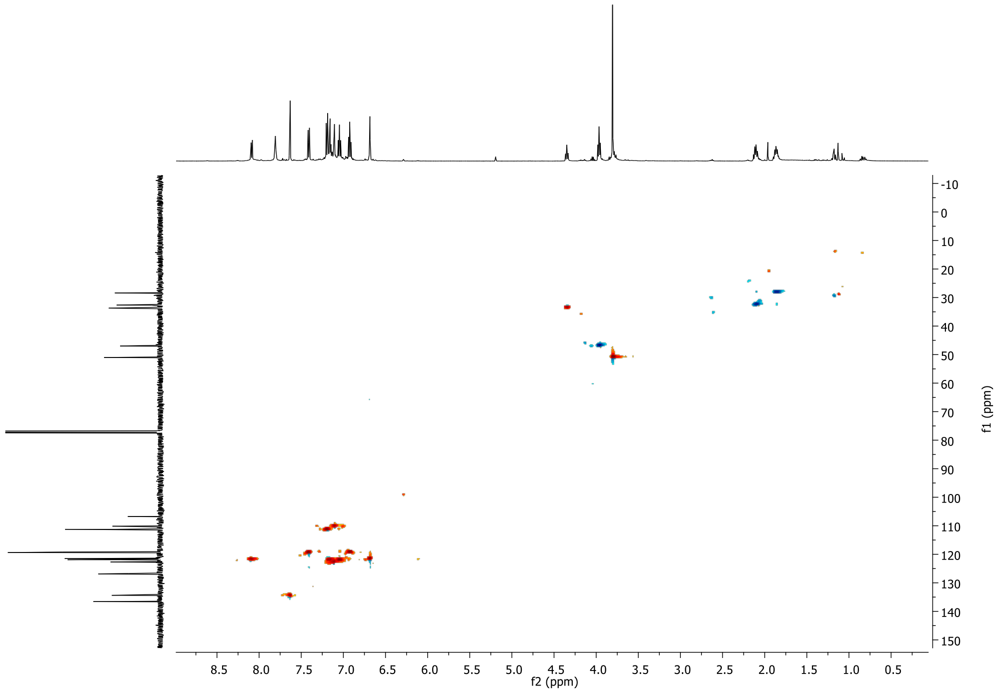

Allyl indole 6la

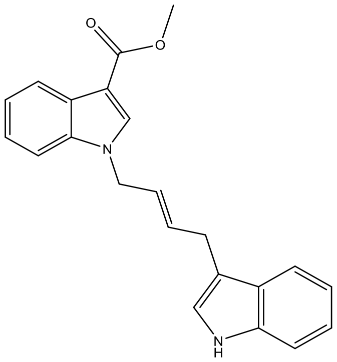

^{1}H NMR (500 MHz, $CDCl_3$, 25°C, TMS)

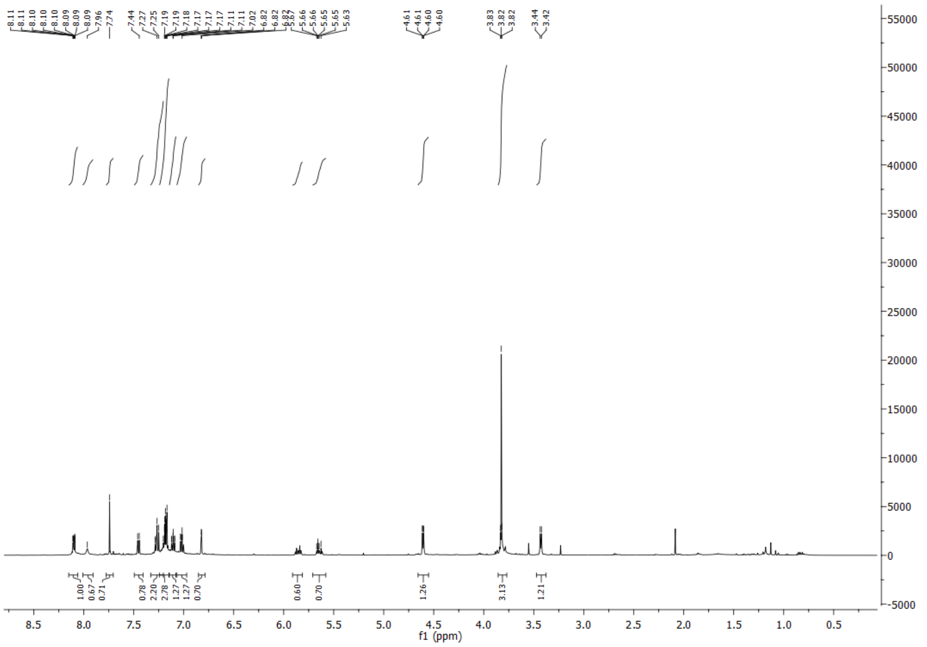

^{13}C NMR (126 MHz, $CDCl_3$, 25°C)

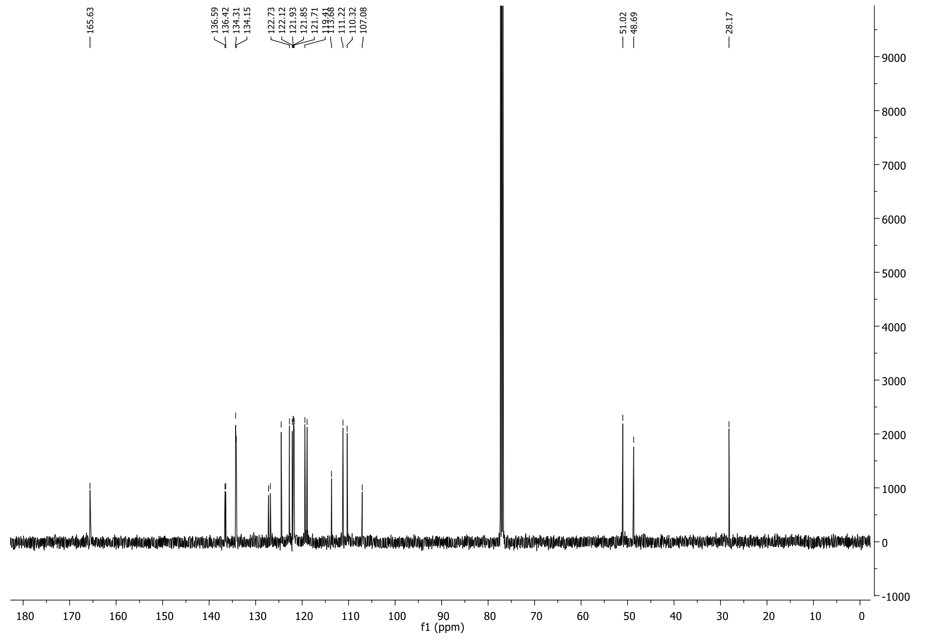

2D HSQC ($CDCl_3$)

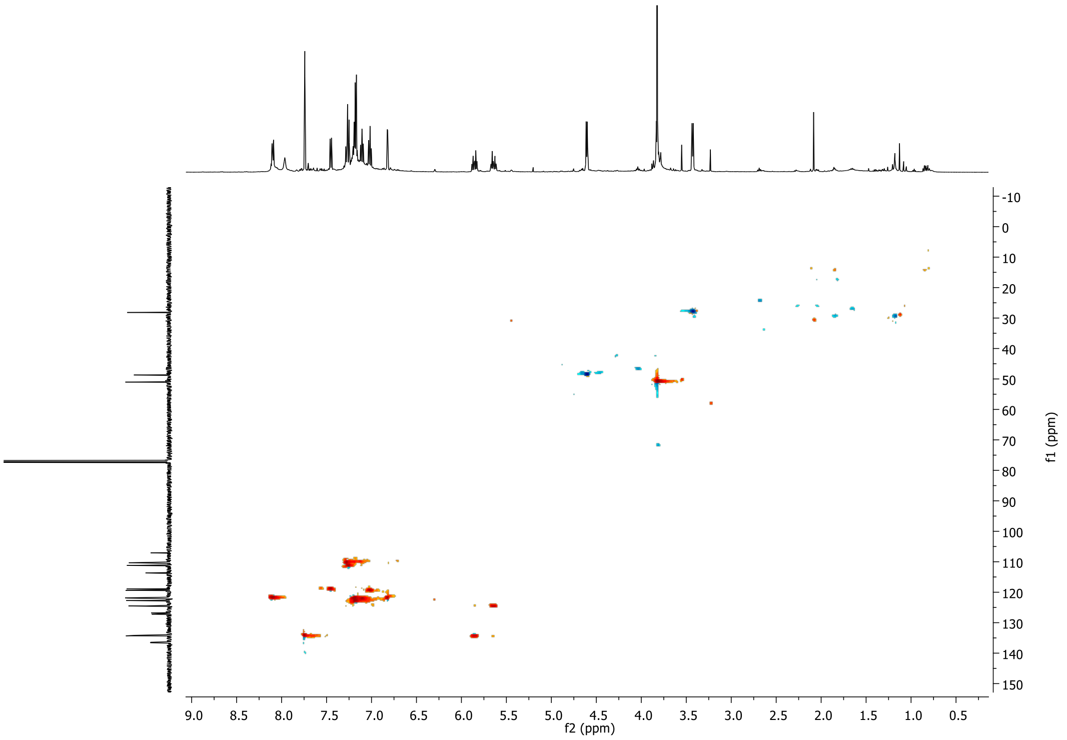

Supporting Information

Trisindole 5ma

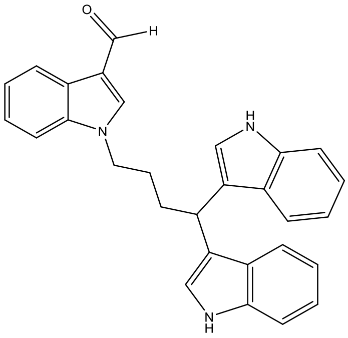

^{1}H NMR (500 MHz, $CDCl_3$, 25°C, TMS)

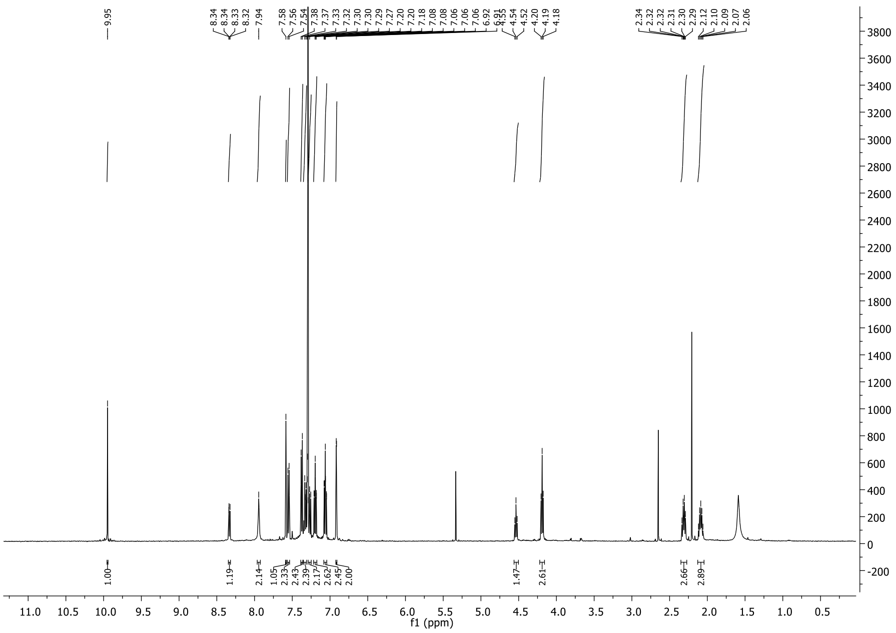

2D HSQC ($CDCl_3$)

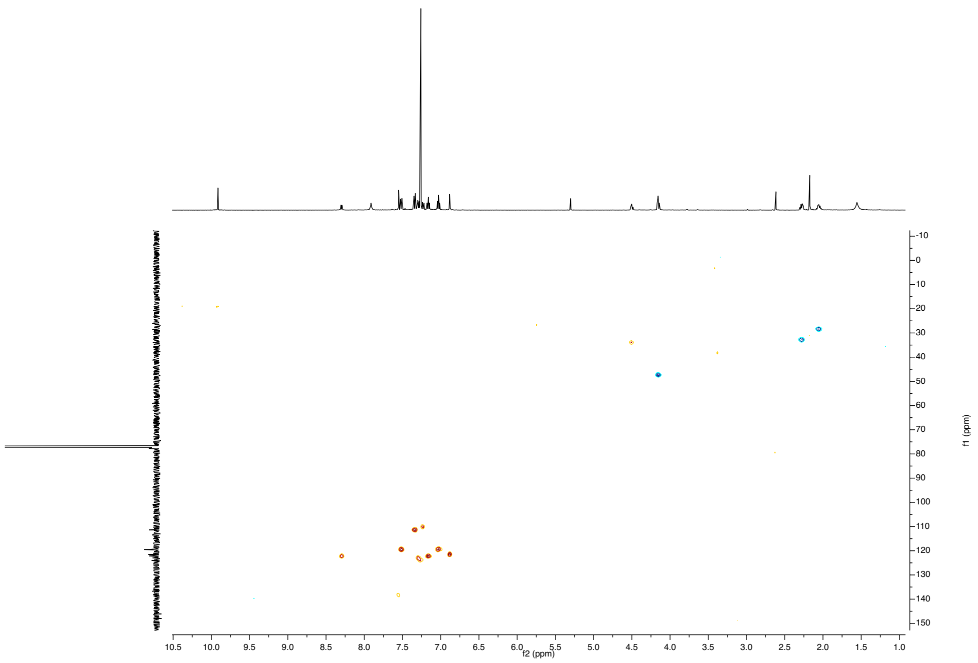

Allyl indole 6ma

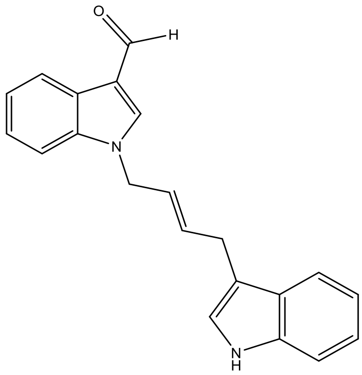

^{1}H NMR (500 MHz, $CDCl_3$, 25°C, TMS)

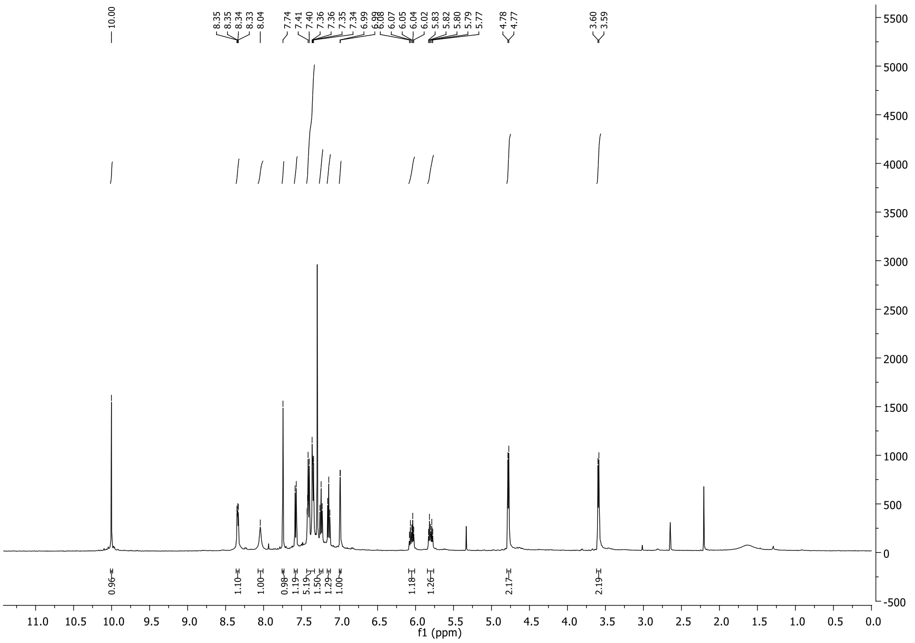

^{13}C NMR (126 MHz, $CDCl_3$, 25°C)

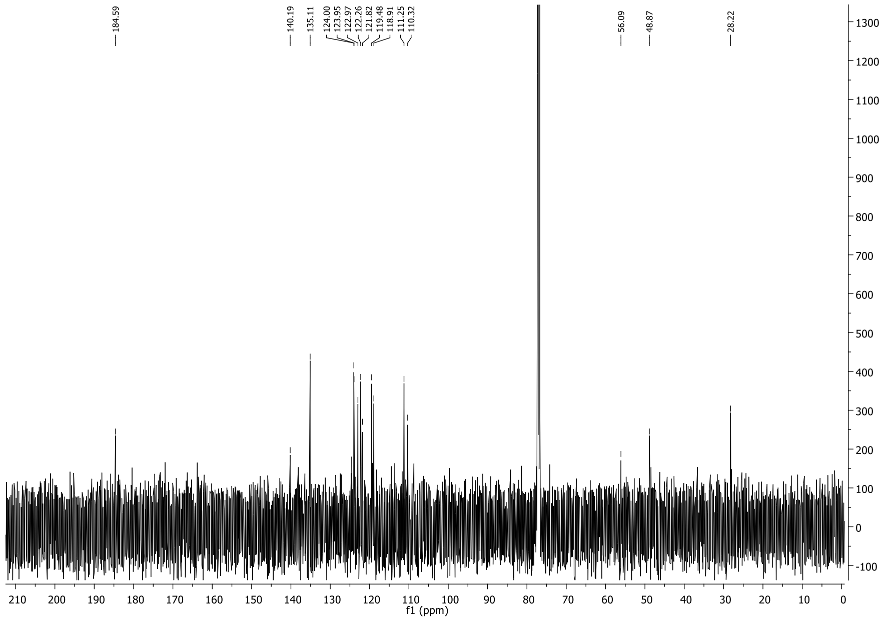

Supporting Information

2D HSQC ($CDCl_3$)

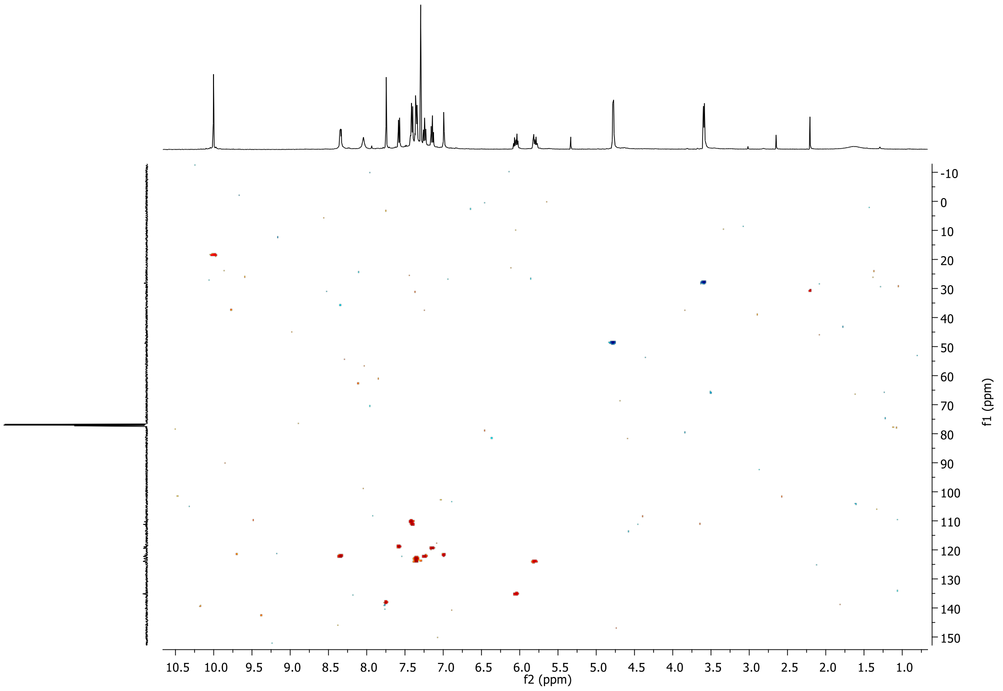

Trisindole 5na

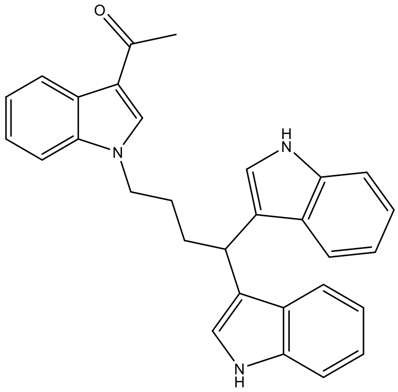

^{1}H NMR (500 MHz, $CDCl_3$, 25°C, TMS)

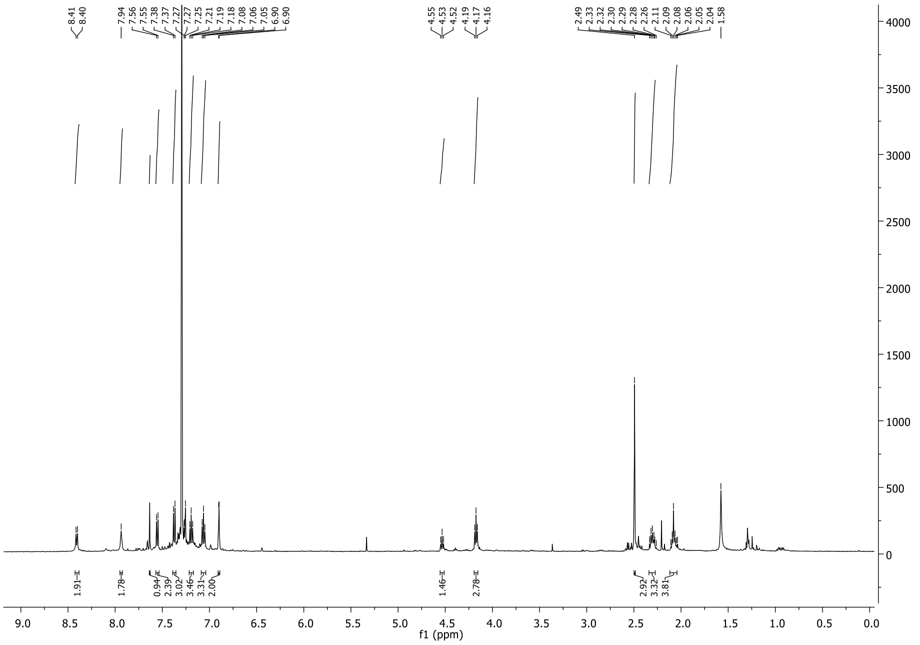

^{13}C NMR (126 MHz, $CDCl_3$, 25°C)

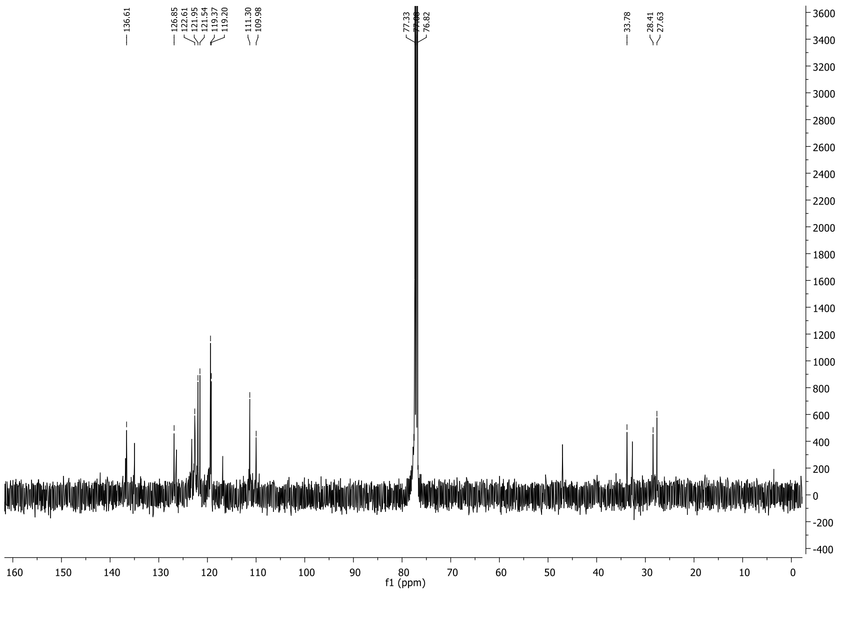

2D HSQC ($CDCl_3$)

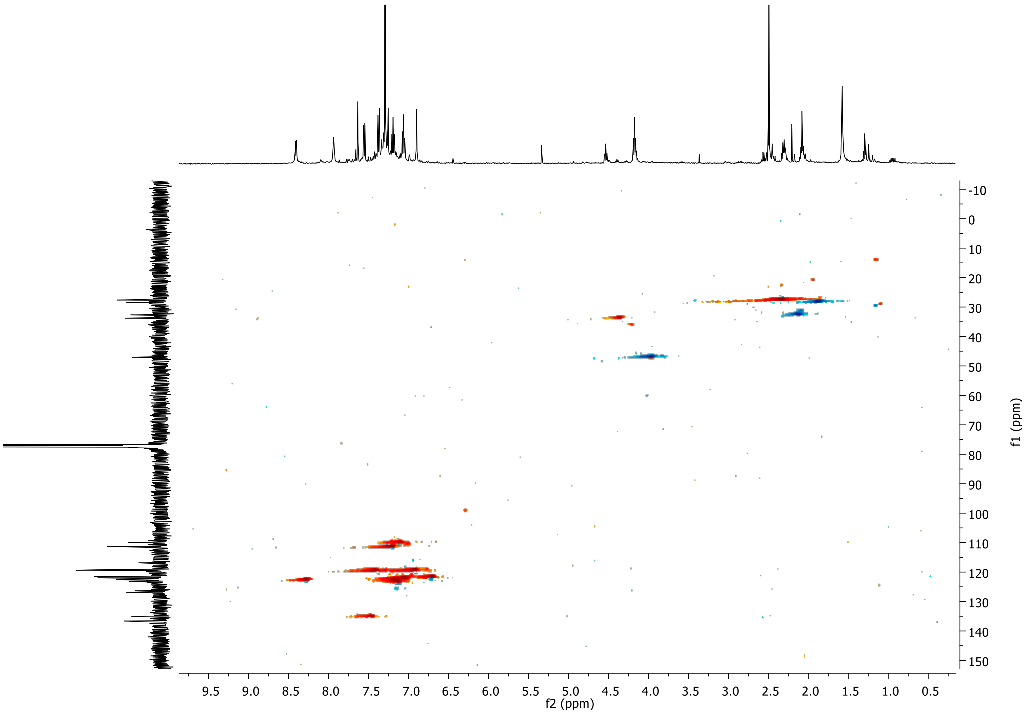

Allyl indole 6na

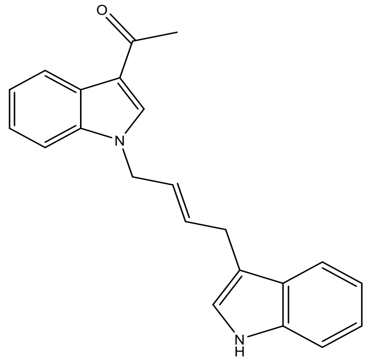

^1H NMR (500 MHz, $CDCl_3$, 25°C, TMS)

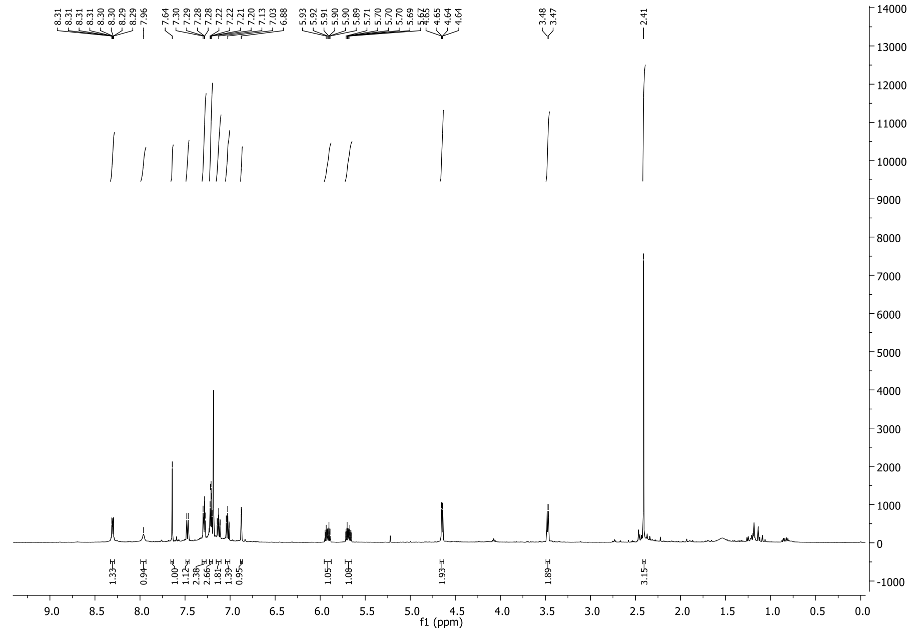

^{13}C NMR (126 MHz, $CDCl_3$, 25°C)

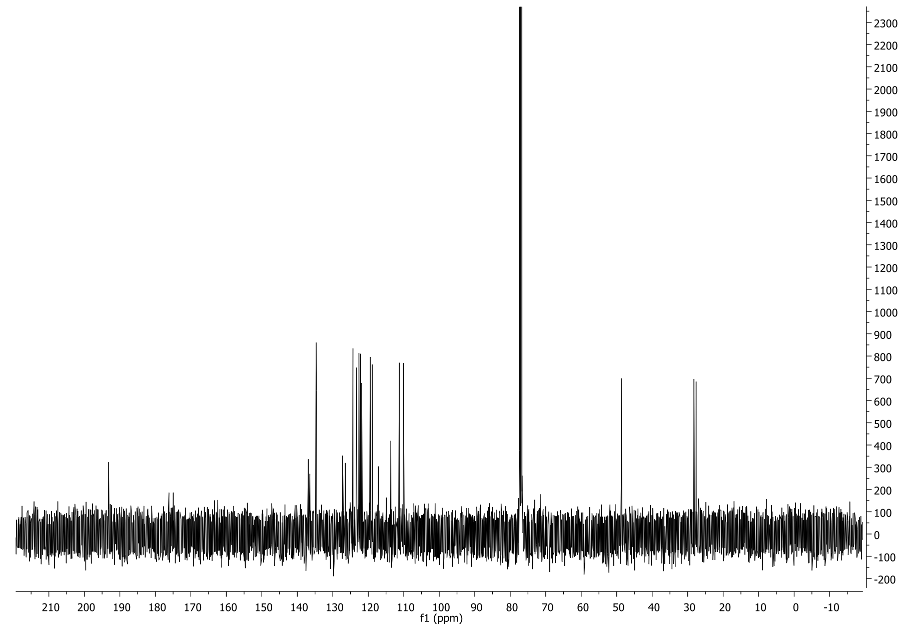

2D HSQC ($CDCl_3$)

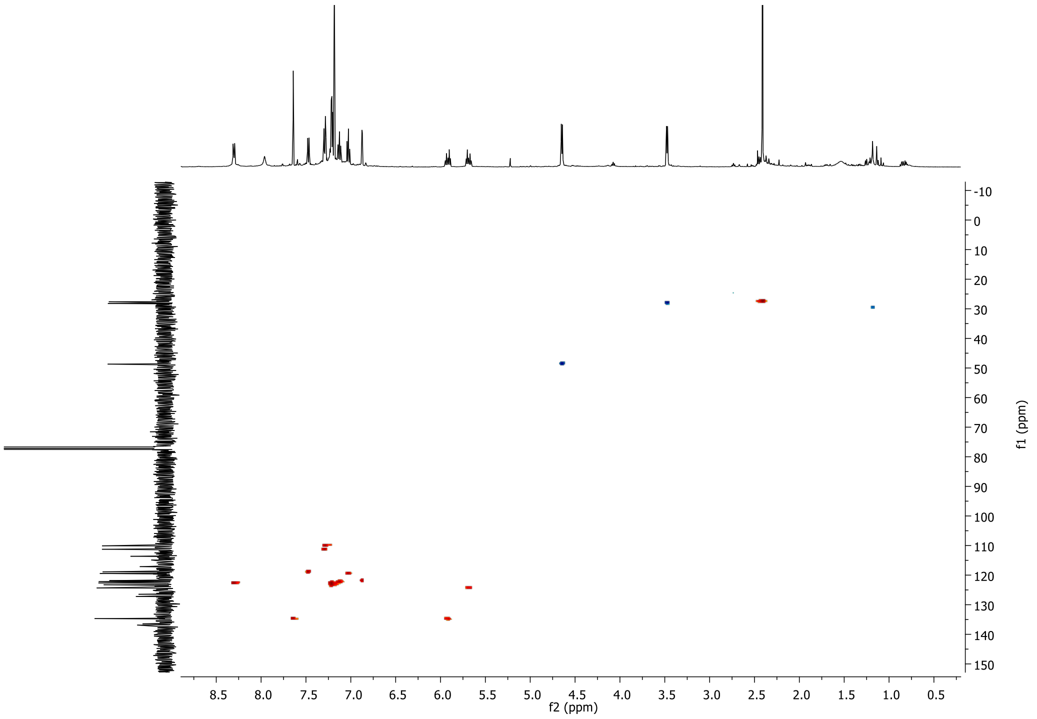

Trisindole 5oa

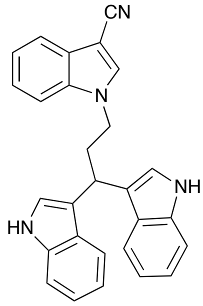

^{1}H NMR (500 MHz, acetone-d_6, 25°C, TMS)

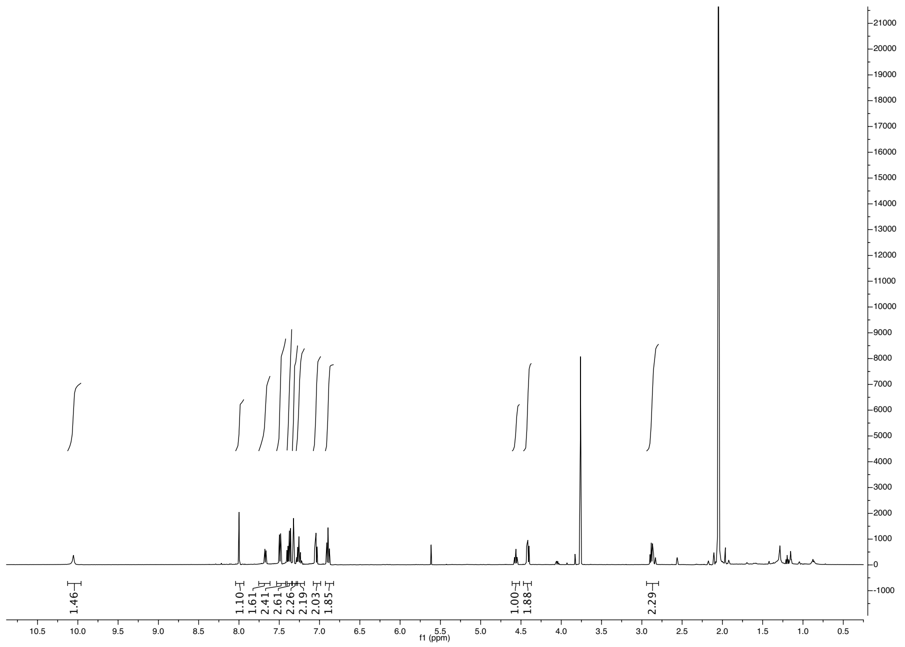

^{13}C NMR (126 MHz, $CDCl_3$, 25°C)

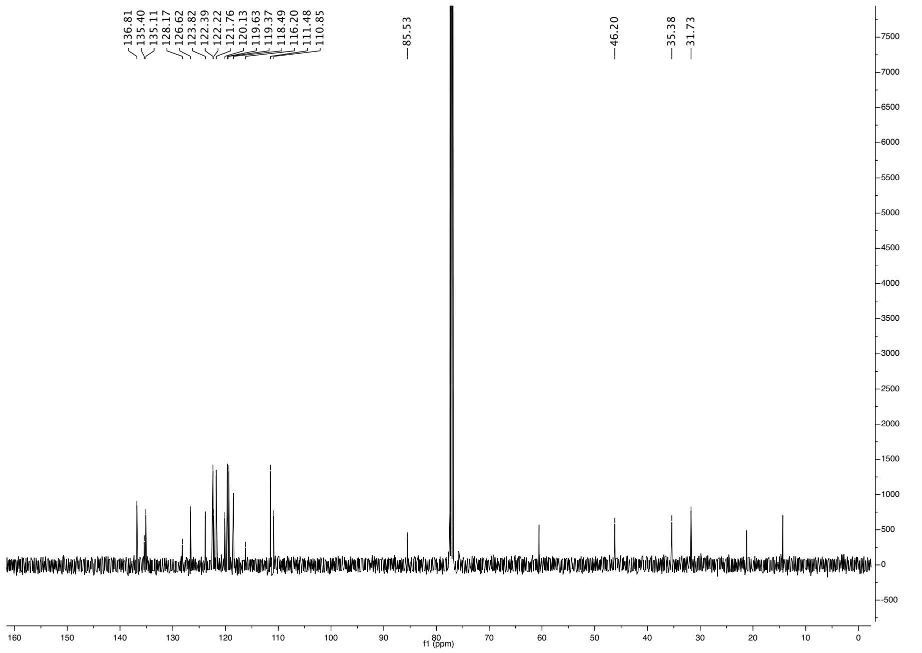

Supporting Information

2D gCOSY (acetone-d_6)

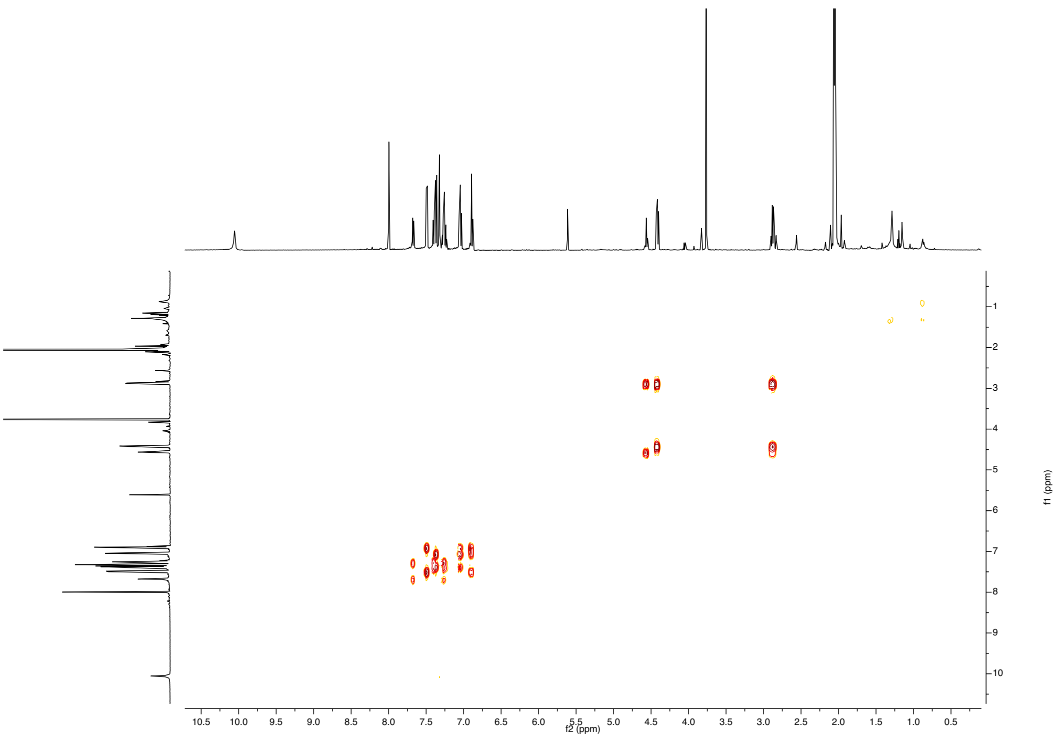

2D HSQC ($CDCl_3$)

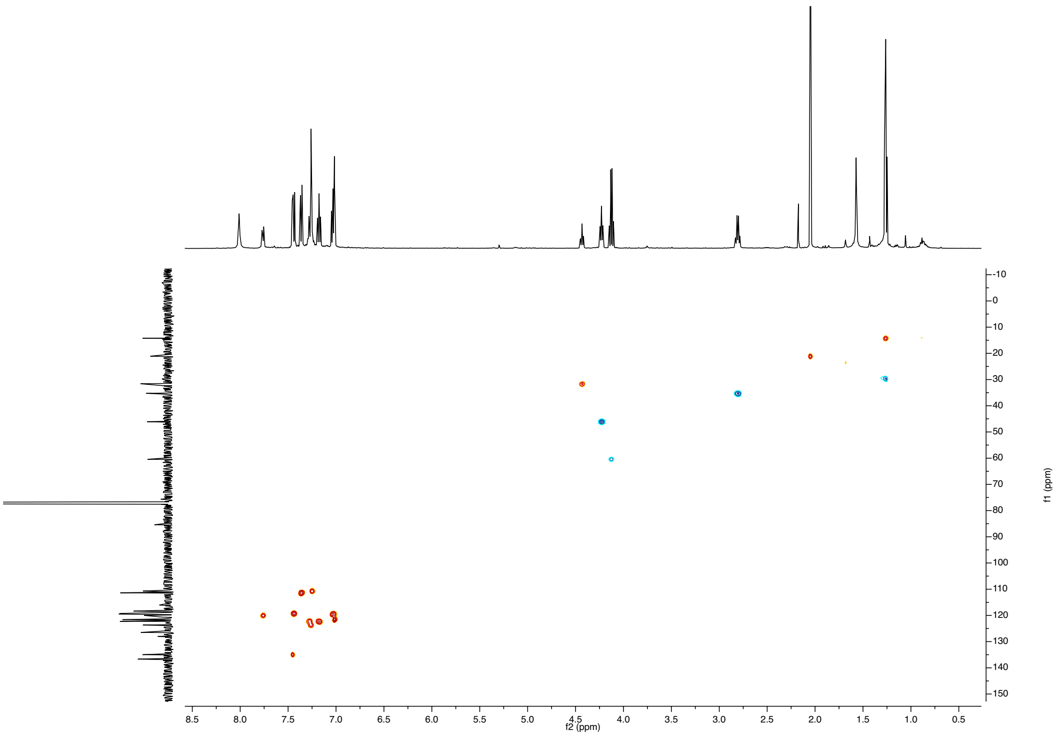

Allyl indole 6oa

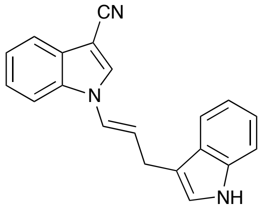

^{1}H NMR (500 MHz, $CDCl_3$, 25°C, TMS)

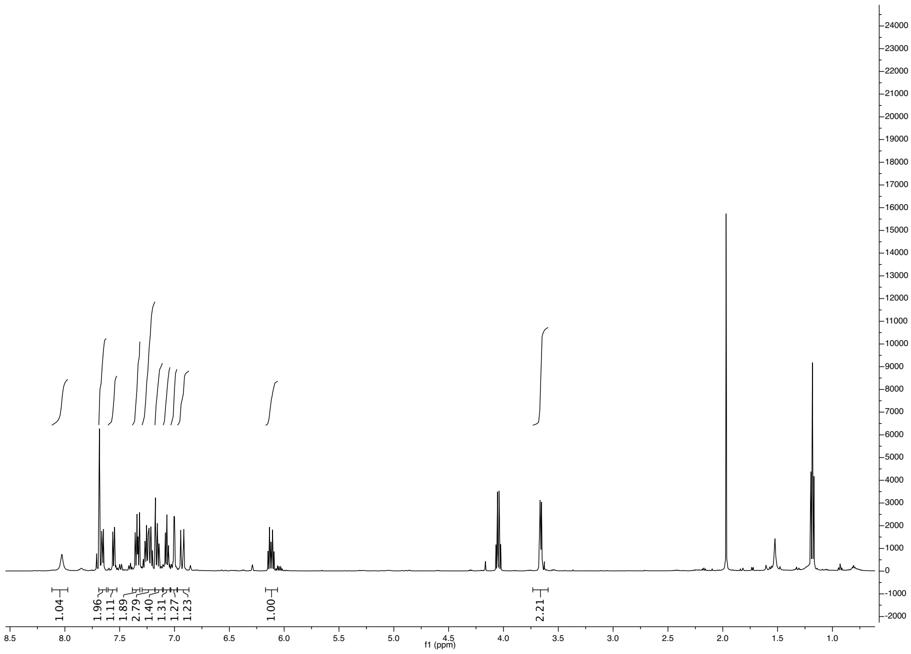

^{13}C NMR (126 MHz, $CDCl_3$, 25°C)

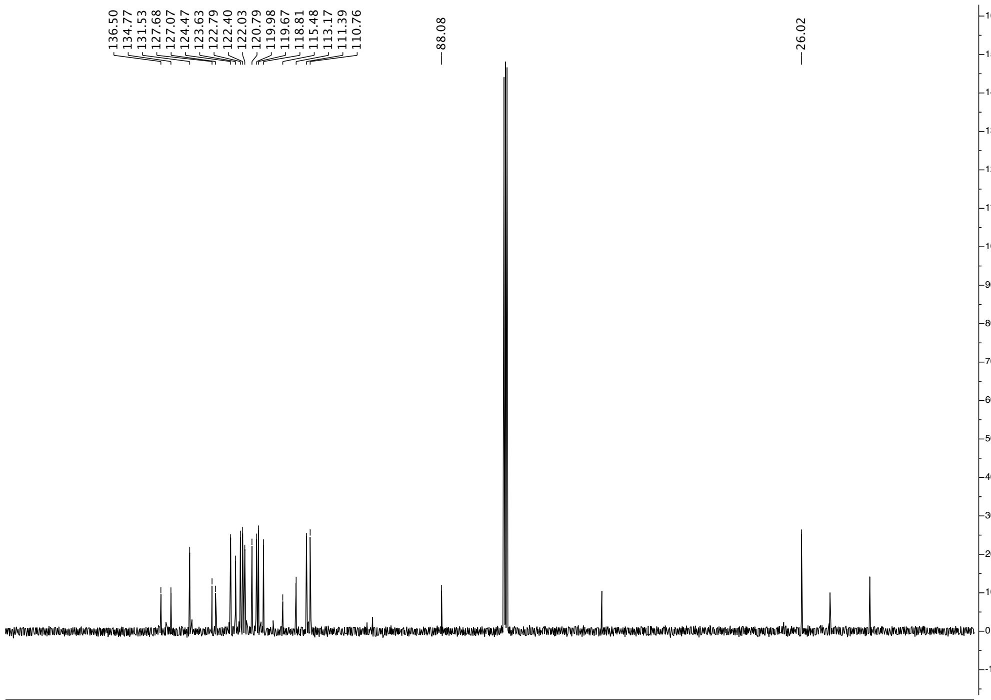

2D gCOSY ($CDCl_3$)

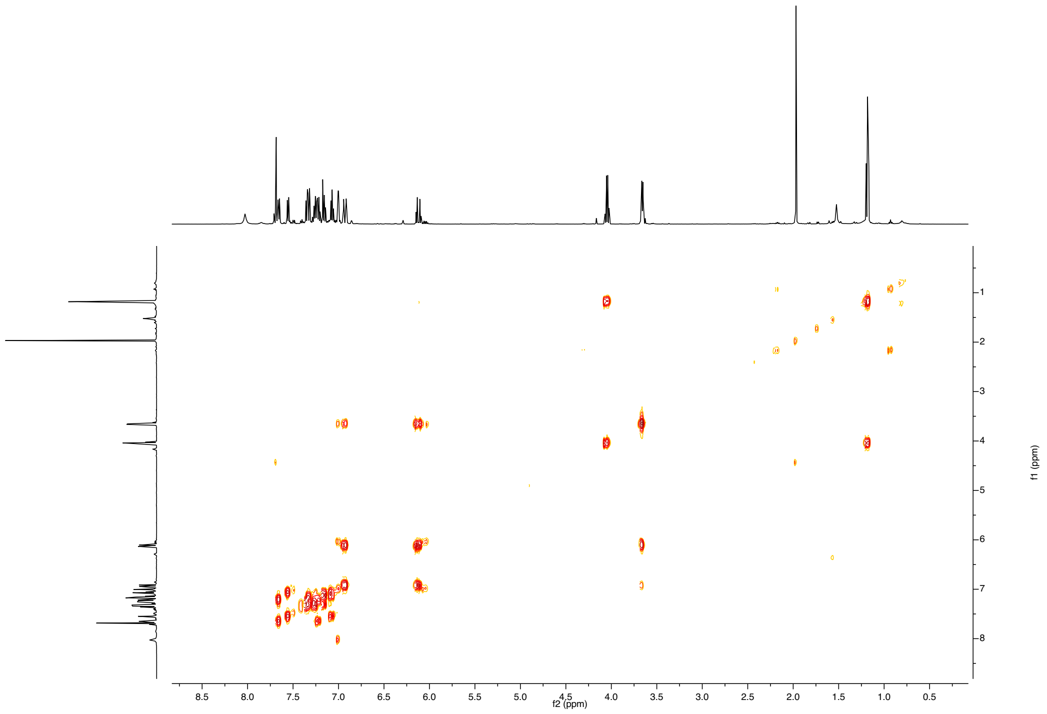

2D HSQC ($CDCl_3$)

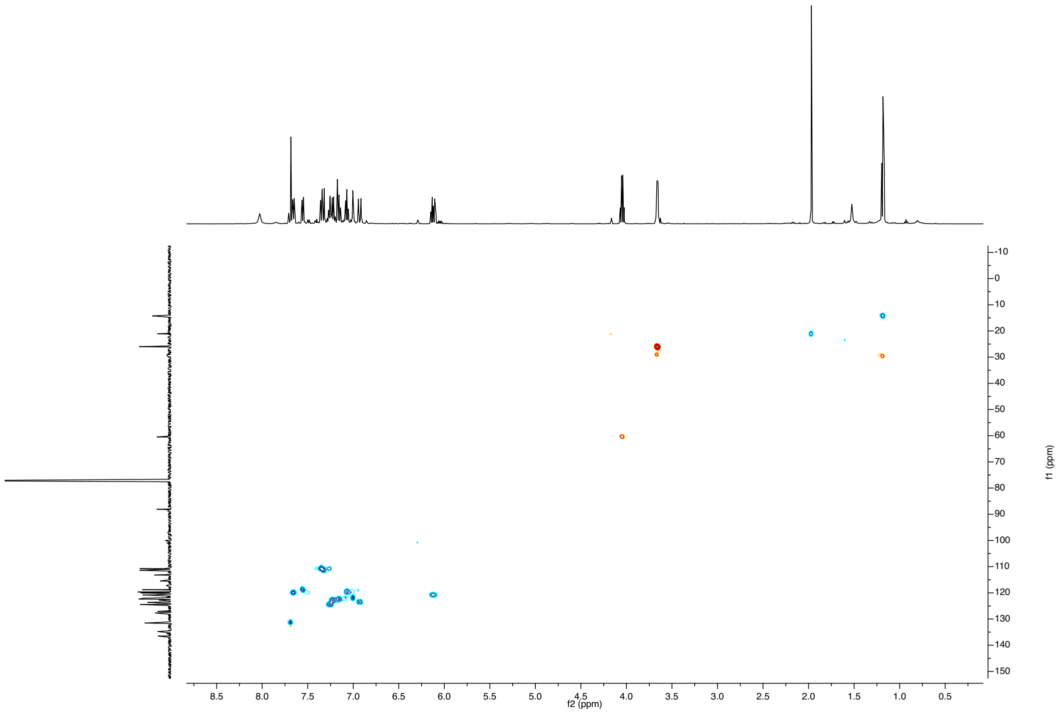

Cycles 4p’ and 4p (traces)

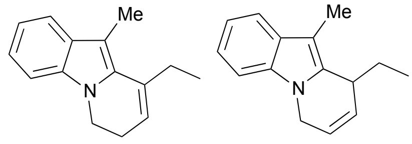

^{1}H NMR (500 MHz, $CDCl_3$, 25°C, TMS)

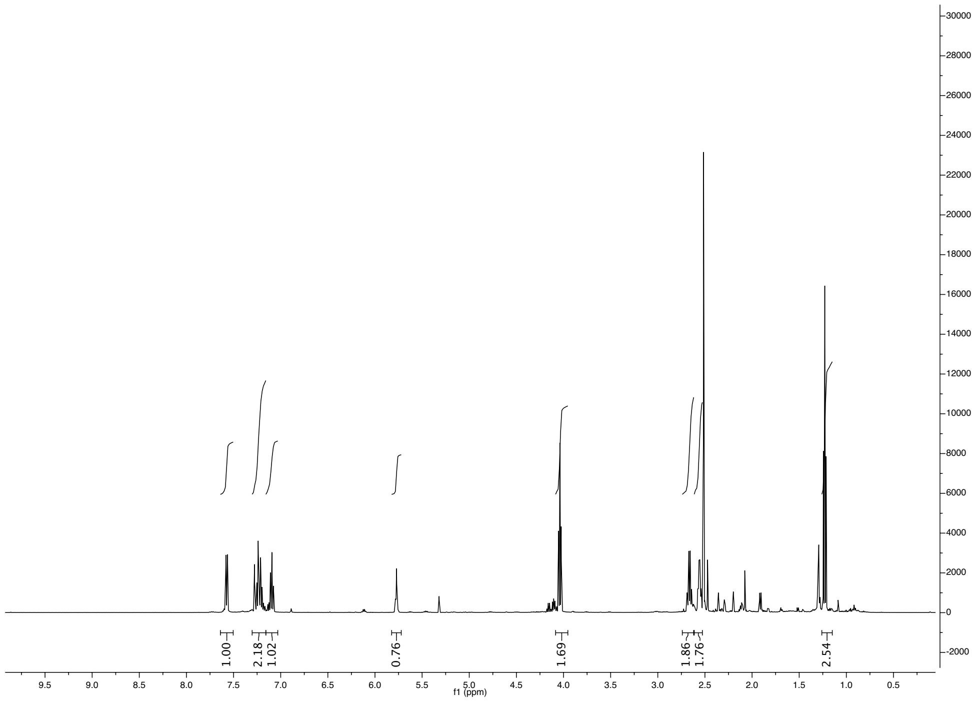

^{13}C NMR (126 MHz, $CDCl_3$, 25°C)

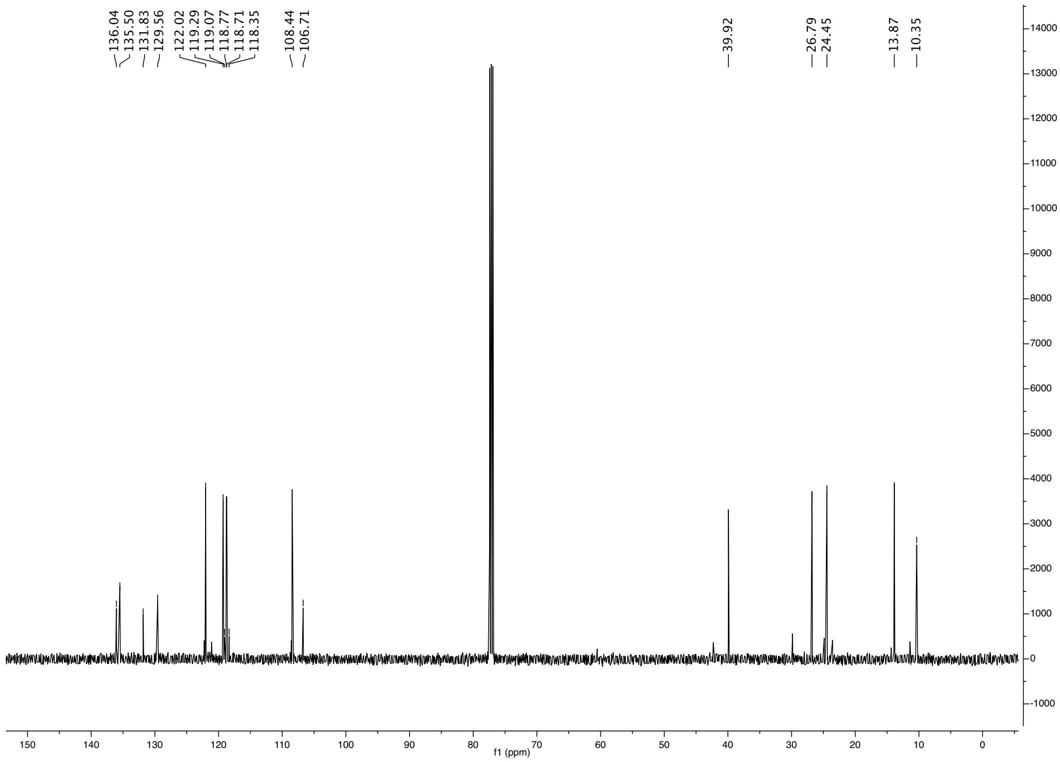

Supporting Information

2D gCOSY ($CDCl_3$)

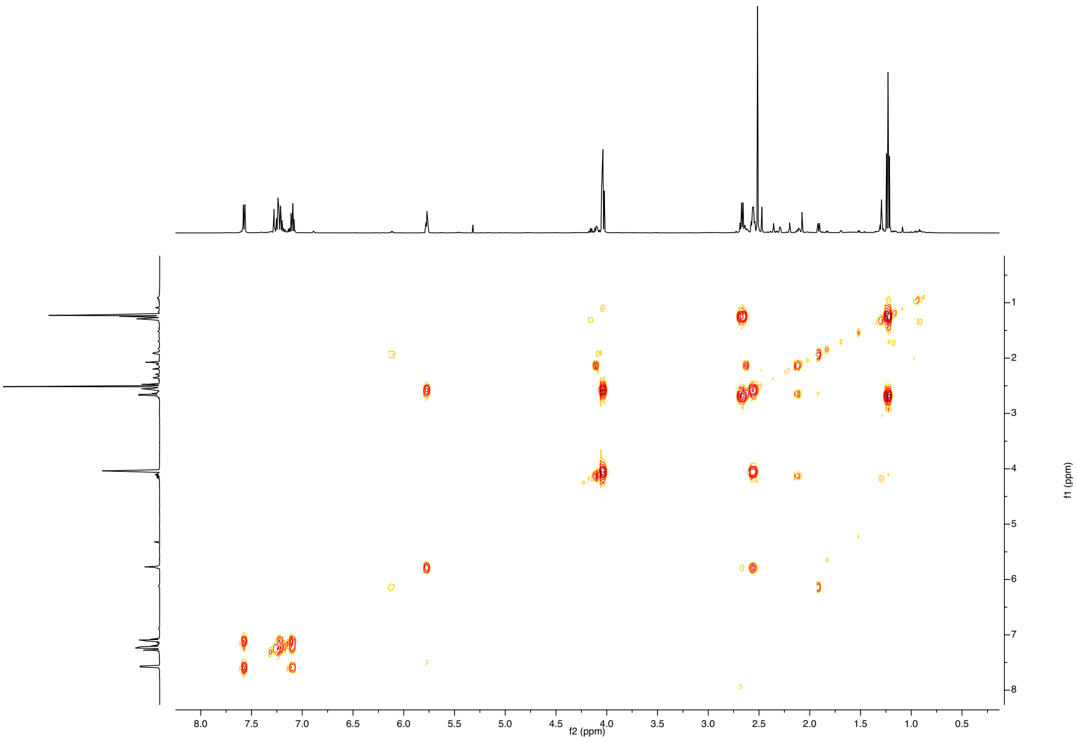

2D HSQC ($CDCl_3$)

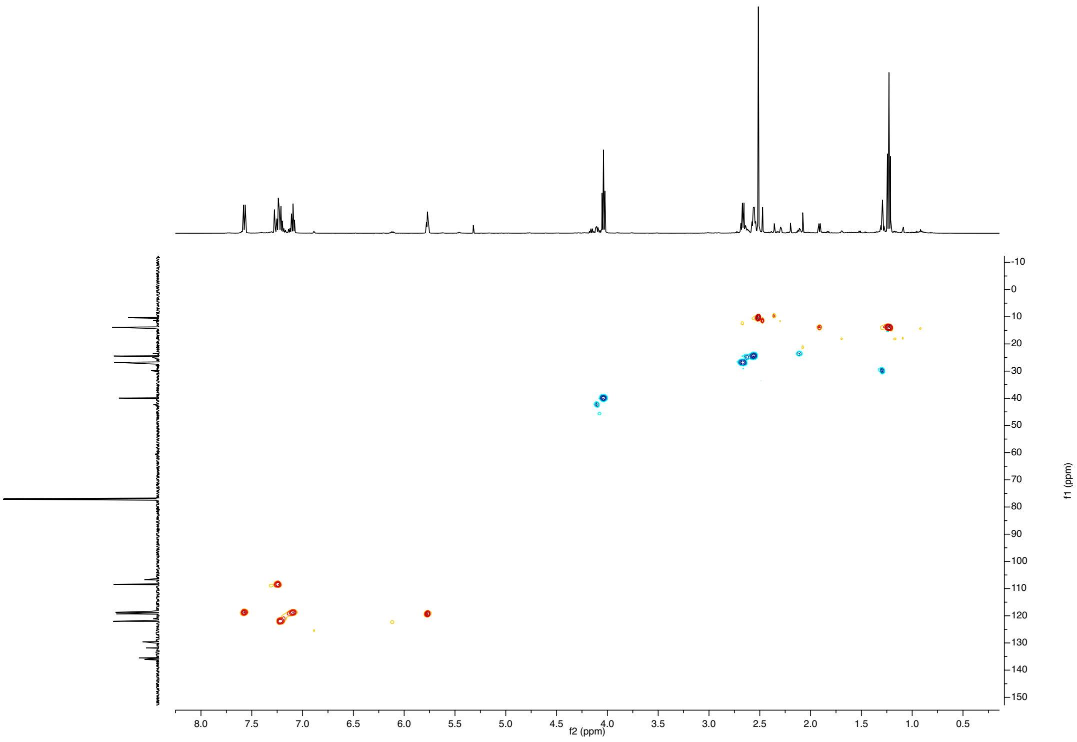

Dimer 7a

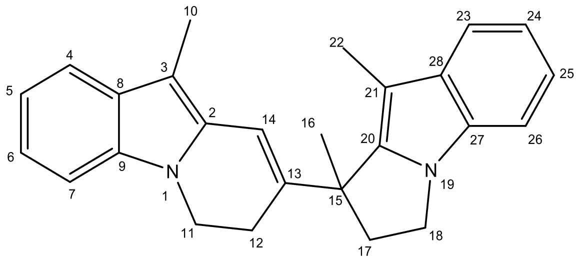

^{1}H NMR (500 MHz, $CDCl_3$, 25°C, TMS)

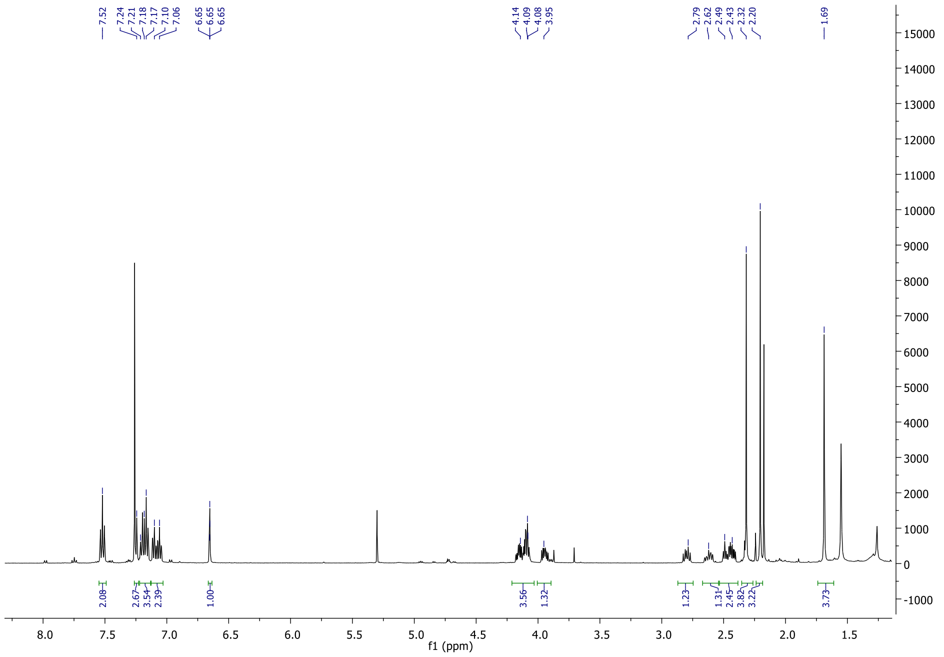

^{13}C NMR (126 MHz, $CDCl_3$, 25°C)

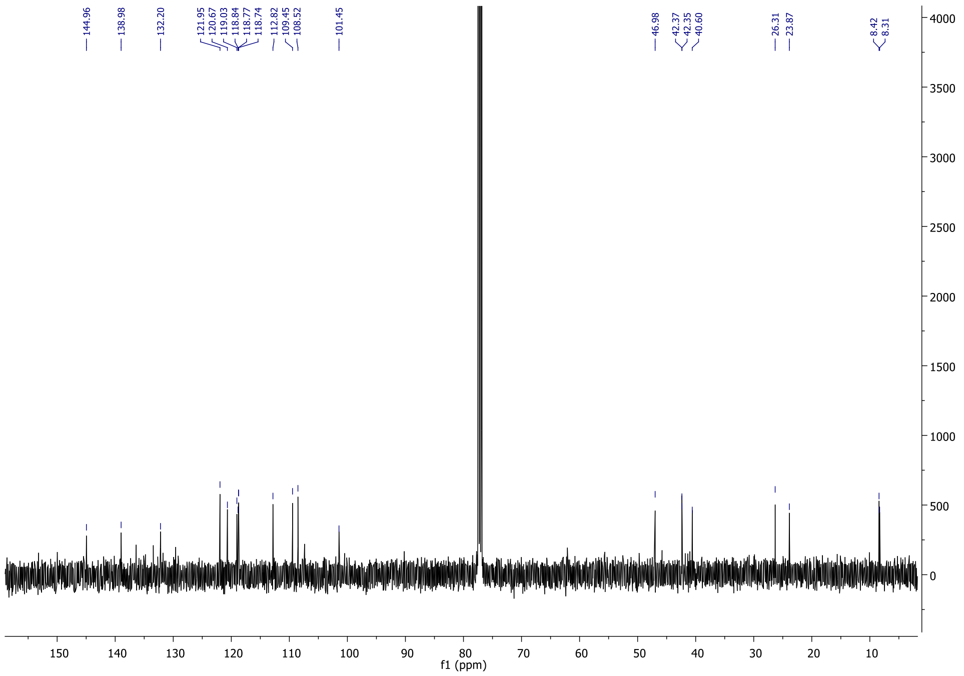

2D gCOSY ($CDCl_3$)

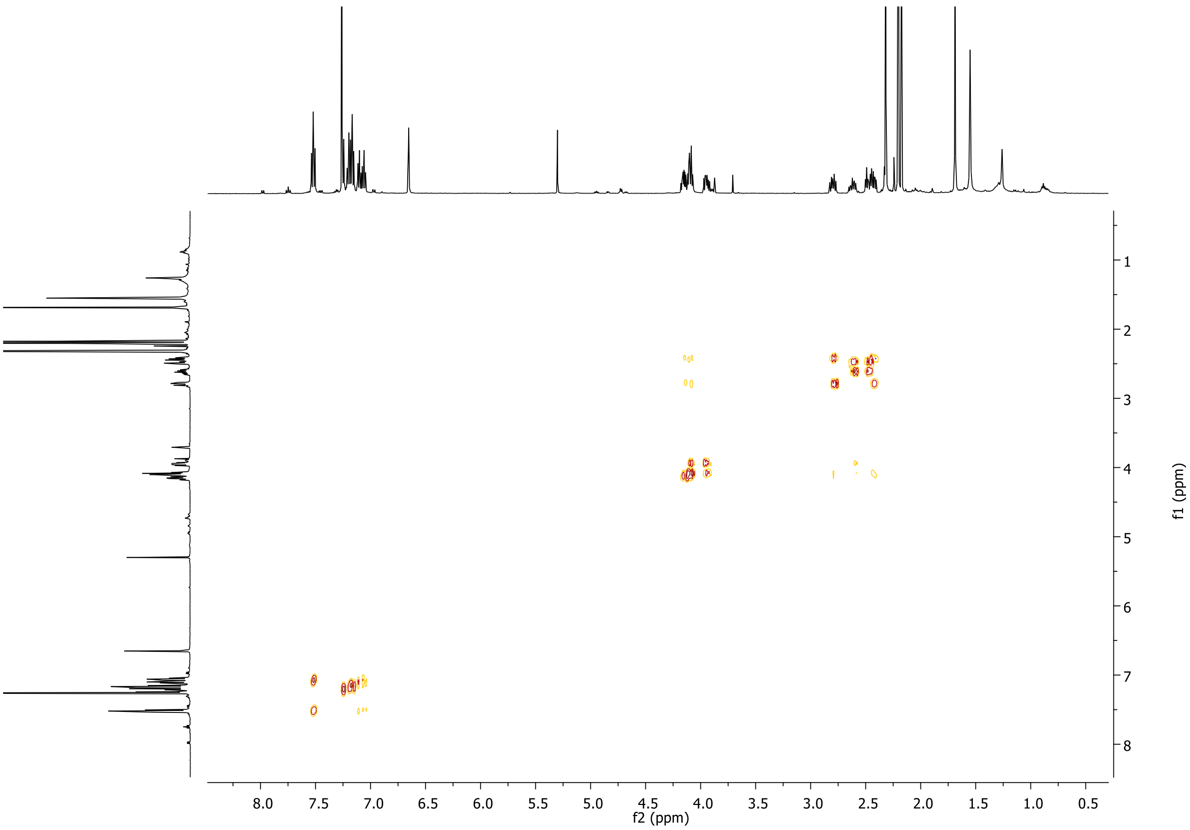

2D HSQC ($CDCl_3$)

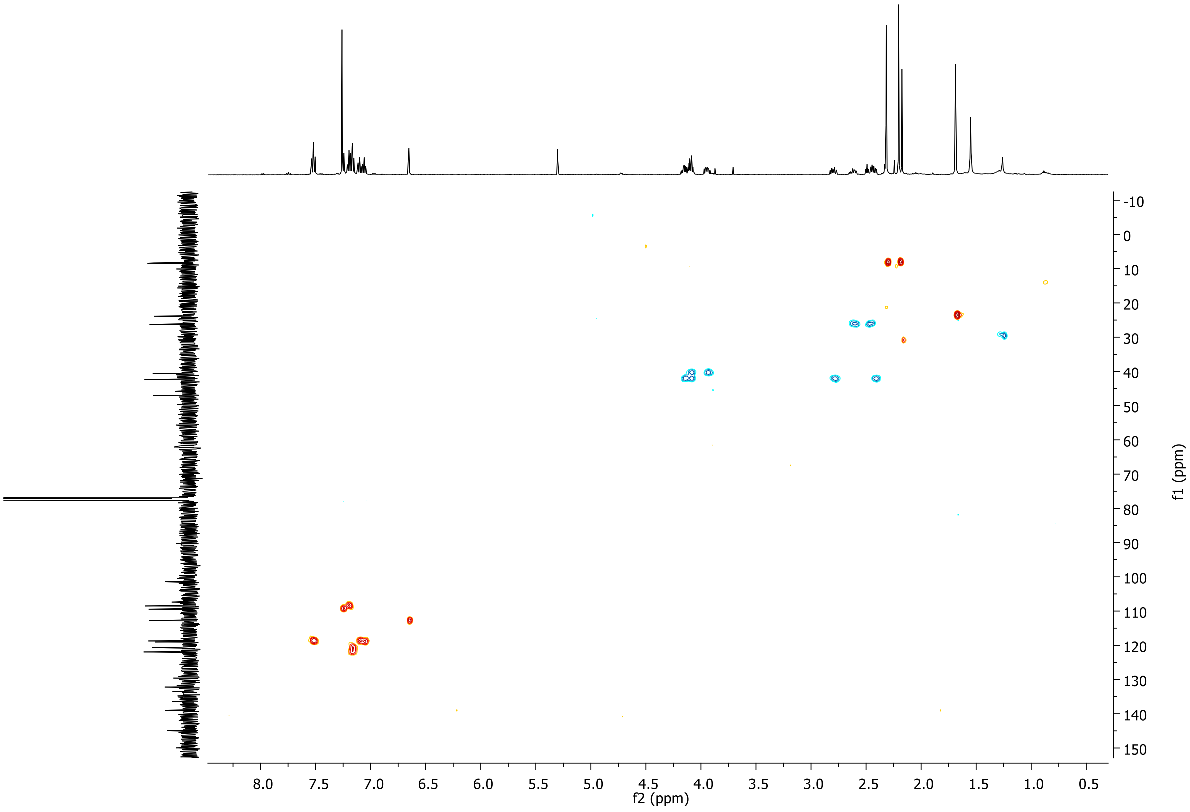

2D HMBC ($CDCl_3$)

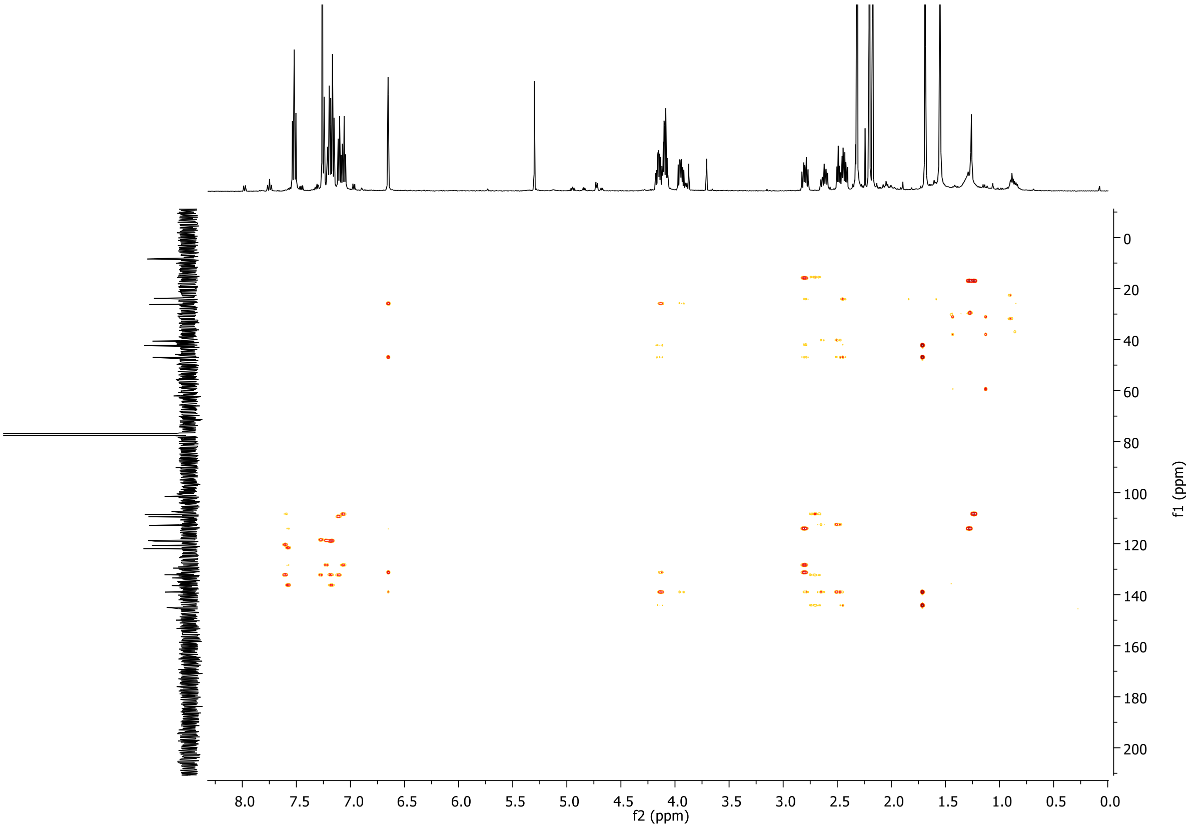

Dimer 7g

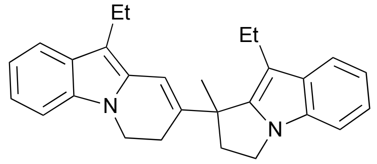

^{1}H NMR (500 MHz, $CDCl_3$, 25°C, TMS)

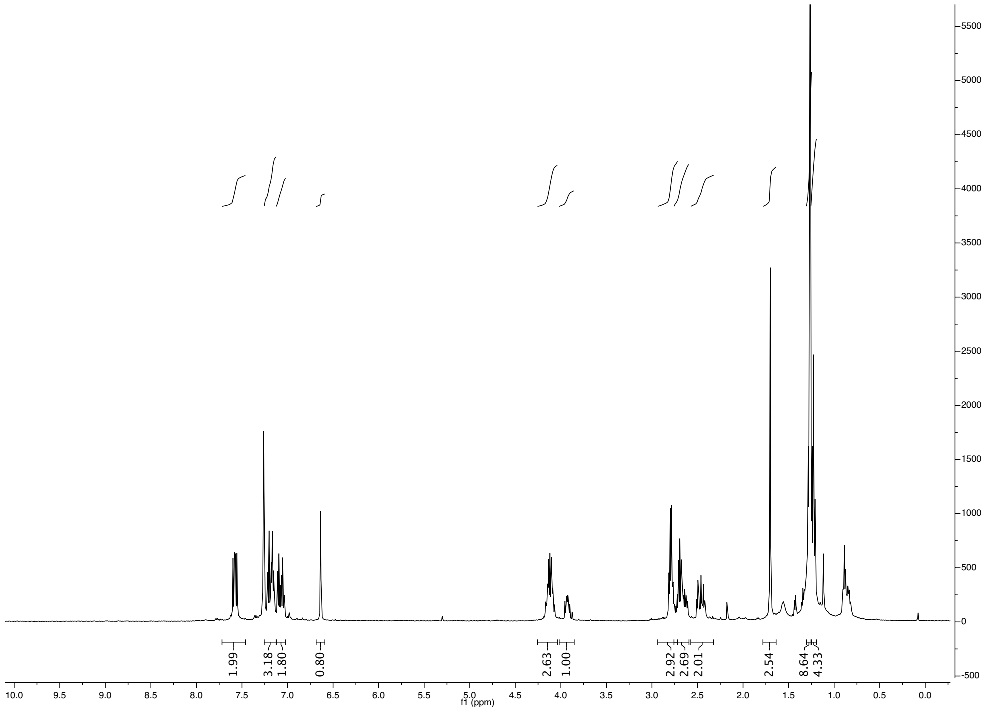

^{13}C NMR (126 MHz, $CDCl_3$, 25°C)

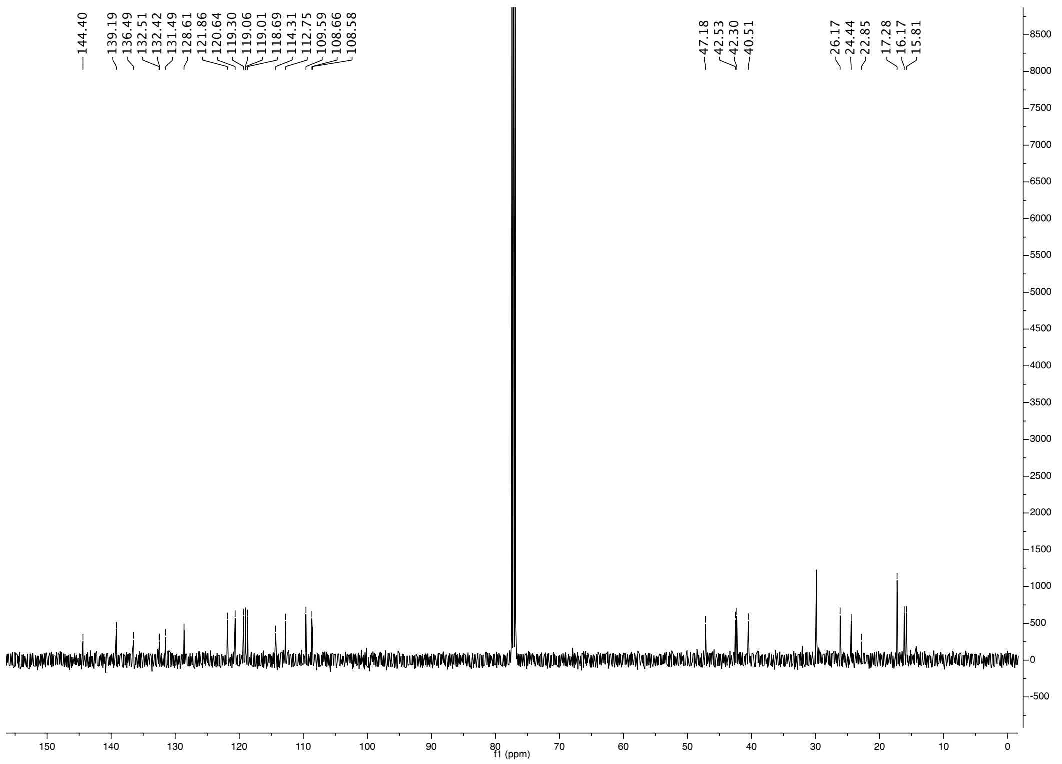

Compound 3an

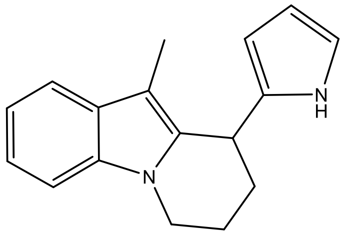

^{1}H NMR (500 MHz, $CDCl_3$, 25°C, TMS)

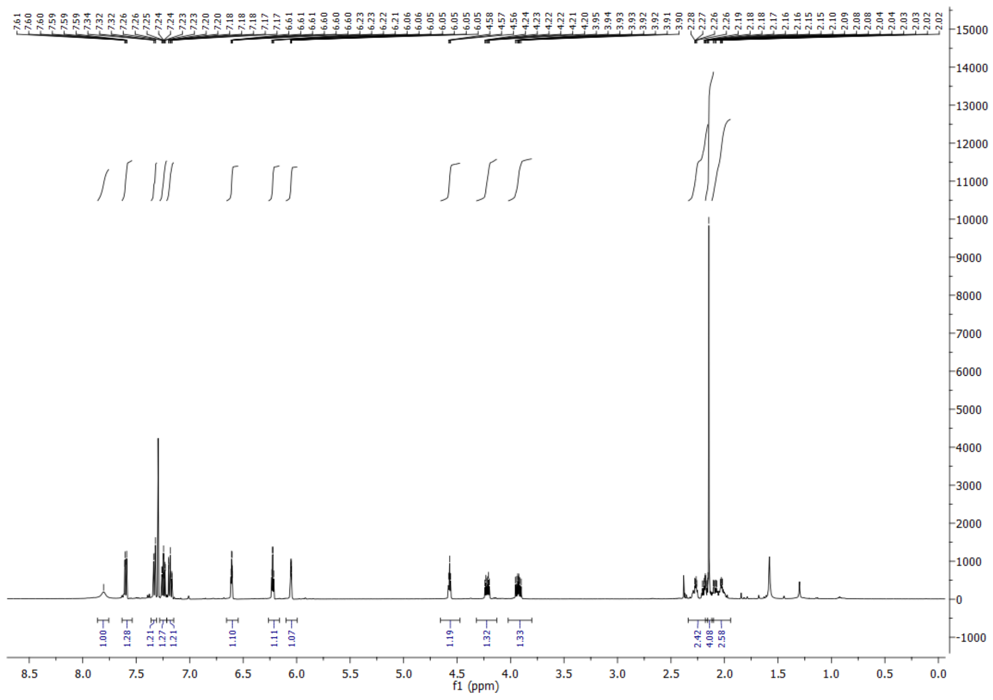

^{13}C NMR (126 MHz, $CDCl_3$, 25°C)

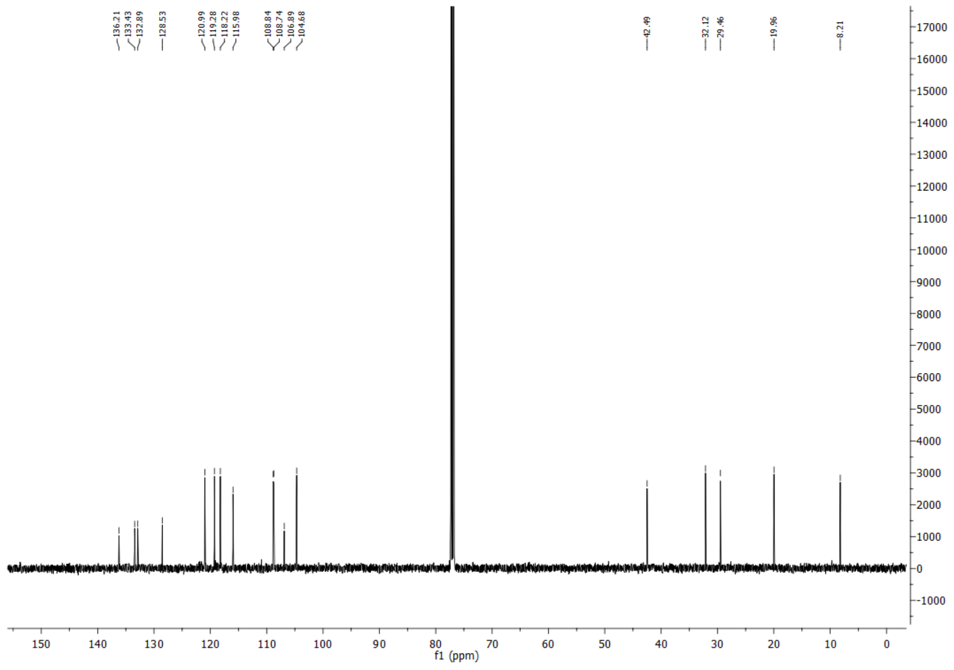

2D gCOSY ($CDCl_3$)

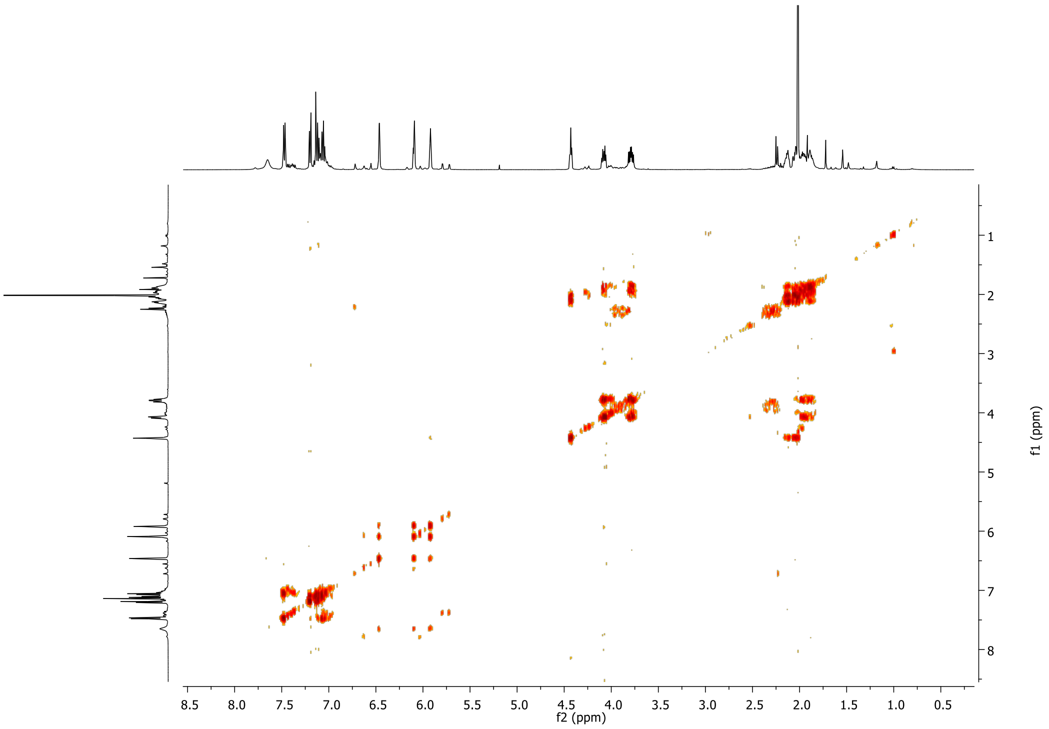

2D HSQC ($CDCl_3$)

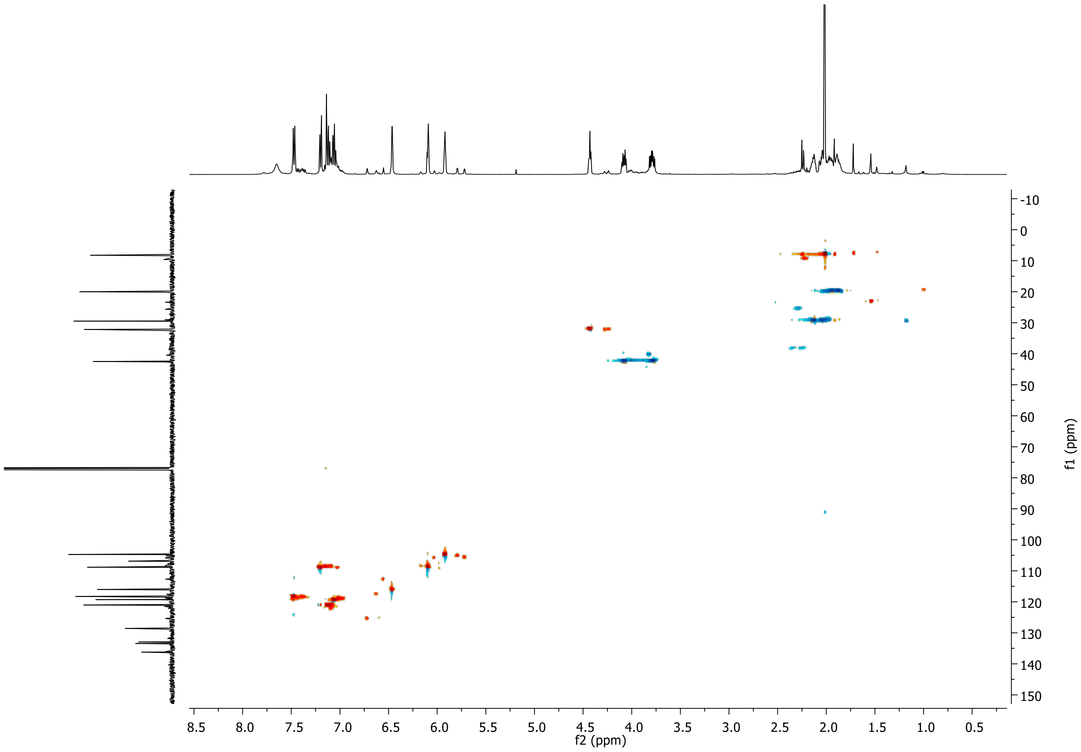

2D HMBC ($CDCl_3$)

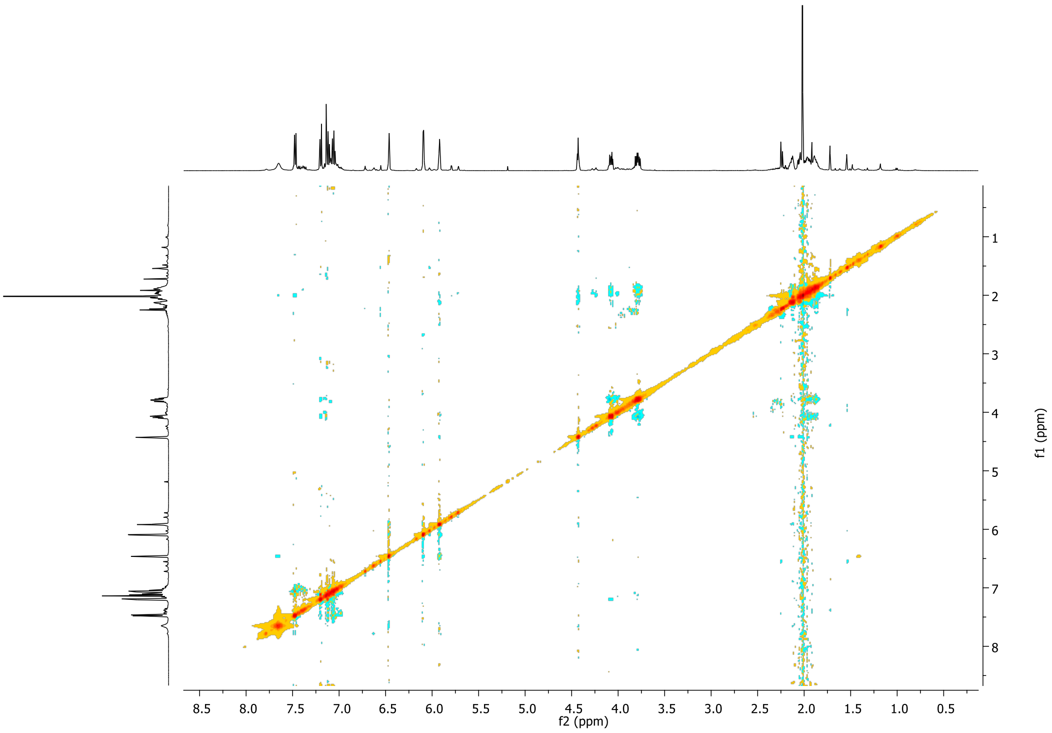

Supplement: Supplementary file 1 — Supplementary [file CHEM-24-6105-s001.pdf]
